# Yawn Contagion and Modality‐Matching in the Female‐Bonded Society of Geladas (*Theropithecus gelada*)

**DOI:** 10.1002/ajp.23709

**Published:** 2024-12-17

**Authors:** Luca Pedruzzi, Paolo Oliveri, Martina Francesconi, Alban Lemasson, Elisabetta Palagi

**Affiliations:** ^1^ EthoS (Ethologie Animale et Humaine) ‐ U.M.R 6552, Université de Rennes Université de Normandie, CNRS Rennes France; ^2^ Unit of Ethology, Department of Biology University of Pisa Pisa Paris Italy; ^3^ Institut Universitaire de France France; ^4^ Natural History Museum University of Pisa Pisa Italy

**Keywords:** behavioral contagion, contagious yawning, female sensitivity, monkeys, multimodal signal mirroring

## Abstract

Behavioral contagion is widespread in primates, with yawn contagion (YC) being a well‐known example. Often associated with ingroup dynamics and synchronization, the possible functions and evolutionary pathways of YC remain subjects of active debate. Among nonhuman animals, geladas (*Theropithecus gelada*) are the only species known to occasionally emit a distinct vocalization while yawning. Yet, the role of different sensory modalities in YC remains poorly understood. Due to their social and communicative complexity, geladas serve as an excellent model for investigating the effects of multimodality and social factors on behavioral contagion. Here we studied a large zoo‐housed colony of geladas (103 subjects, 1422 yawns) and confirm the previous evidence for visual and auditory YC. Hearing, seeing, or hearing and seeing yawns significantly triggered contagious yawning at comparable levels. Additionally, we found no evidence of laterality influencing responses based on the side of detection. While the social bond, measured via grooming, between the trigger and receiver did not correlate with YC, a consistent sex effect emerged. Females responded more frequently to female than to male yawns and were more likely to match modality (i.e., vocalized vs. nonvocalized) and mirror morphology of other females' yawns. Effective female‐female communication and affiliation are crucial for maintaining cohesion and fostering strong intra‐unit relationships among geladas. Our results underscore the importance of different sensory components in the distribution of YC, particularly for species living in complex social systems. These findings raise further questions about the functional and emotional significance of yawning and potential inter‐sexual differences, suggesting that the phenomenon is more complex than previously thought.

## Summary

- Both visual and vocal cues induce yawn contagion in geladas, with yawns of different morphology and duration being similarly contagious
- Yawn contagion comes with modality matching and morphological mirroring in geladas; notably, female‐female dyads exhibit the strongest rates of yawn contagion, matching and mirroring
- Unlike previous data, dyadic grooming exchanged do not predict yawn contagion propensity

## Graphical abstract

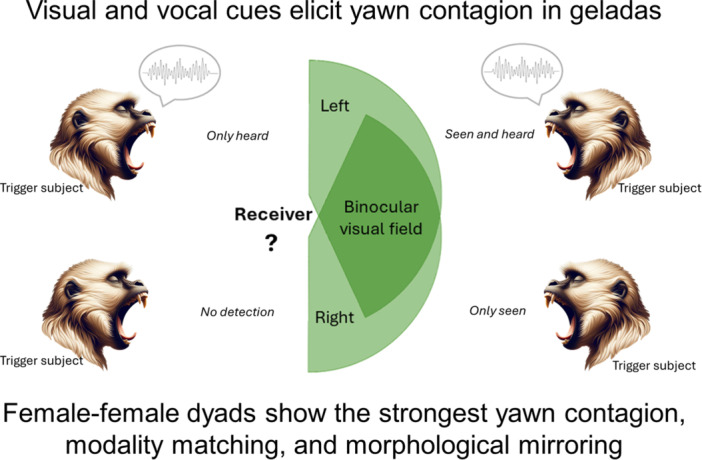

## 1 Introduction

**Figure 1:**
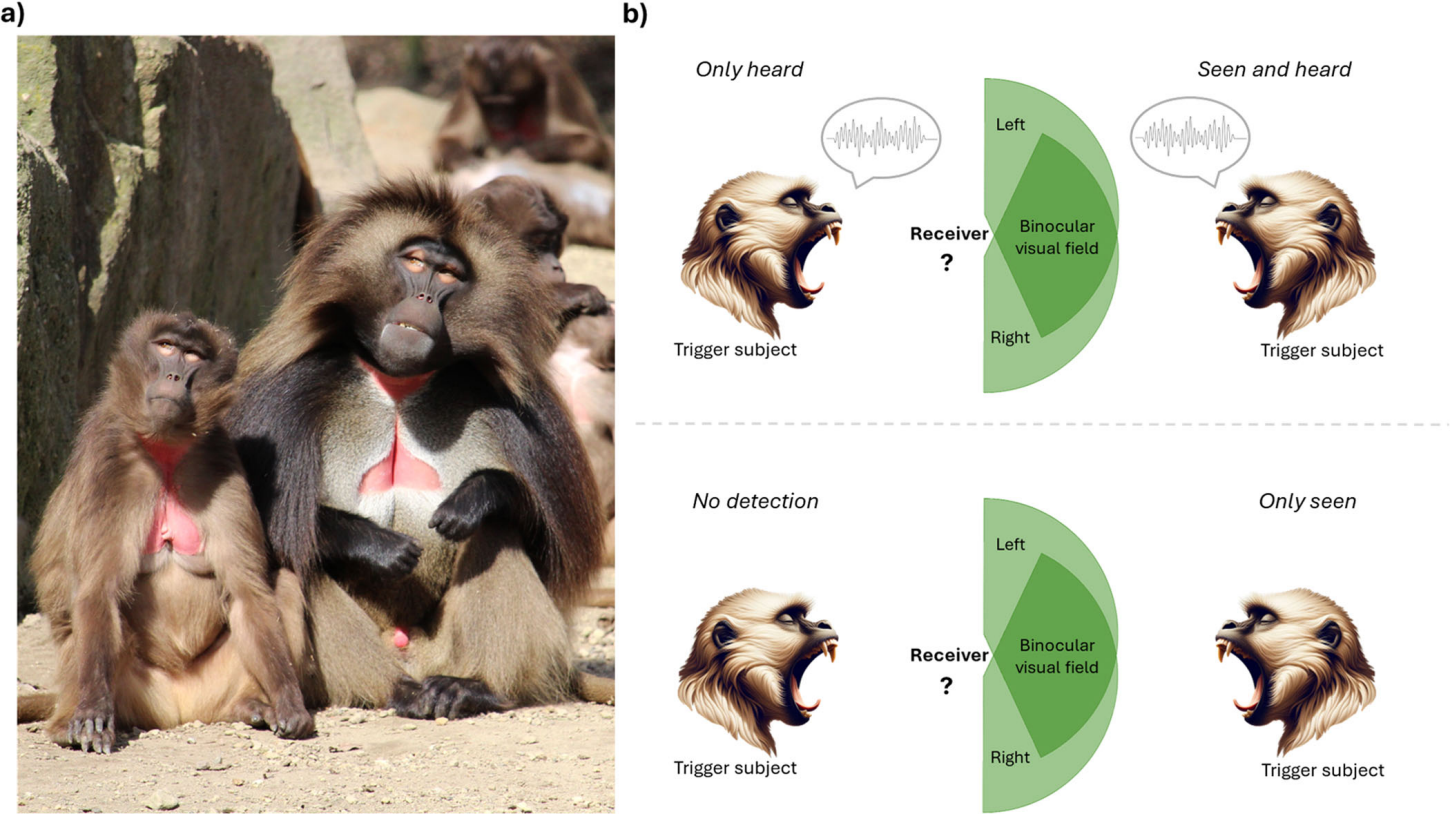
(a) Picture depicting an adult female (left) and adult male (right) geladas of the study group (pictures by MF). Geladas show strong sexual dimorphism in size and secondary sexual traits. (b) Schematic representation of the possible *Detection* conditions in geladas for vocalized and nonvocalized yawns according to the general visual field of monkeys.

Yawn contagion (YC) is a social phenomenon that has been attracting the interest of primatologists and comparative psychologists in the last two decades (Palagi et al. 2020). Compared to spontaneous yawning, YC seems a derived trait mostly found in highly social species, where selective pressures have promoted several mechanisms of behavioral pairing (Duranton and Gaunet 2016). Seeing yawns elicit yawning in many nonhuman animals such as great apes (Amici, Aureli, and Call 2014; Demuru and Palagi 2012; Massen, Vermunt, and Sterck 2012; van Berlo et al. 2020, but not gorillas, Palagi, Norscia, and Cordoni 2019), monkeys (Galotti et al. 2024; Palagi et al. 2009; Pedruzzi et al. 2022; Valdivieso‐Cortadella et al. 2023), lemurs (Valente et al. 2023, but see Reddy et al. 2016) and nonprimate species (carnivores, Ake and Kutsukake 2023; Casetta, Nolfo, and Palagi Nolfo, and Palagi 2021, 2022; Romero, Konno, and Hasegawa 2013, 2014; pigs, Norscia et al. 2021; birds, Gallup et al. 2015, but see Gallup et al. 2022). YC has been inconsistently categorized as an example of motor mimicry, emotional sharing, or behavioral contagion (Massen and Gallup 2017; Palagi et al. 2020; Yoon and Tennie 2010). The current evidence does not rule out the possibility that YC might not serve an adaptive function and that could be a byproduct of overlapping neural mechanisms related to attention and arousal (Gallup 2022; Massen and Gallup 2017; Palagi et al. 2020). However, among the context‐dependent and not‐mutually‐exclusive functions of YC, its adaptive value seems linked to its role in promoting vigilance (Gallup and Meyers 2021), body synchronization (Casetta, Nolfo, and Palagi 2021) and activity/state changes (Casetta, Nolfo, and Palagi 2022; Galotti et al. 2024), or even affiliation (Poole and Henderson 2023) among group members. Here we carried out an observational study on a large zoo‐housed colony of geladas (*Theropithecus gelada*) to investigate the role of visual and vocal cues in yawn contagion (Gallup 2022; Palagi et al. 2009) as well as the role of social/sex factors in the modulation of the phenomenon. Geladas (Figure 1a) are monkeys endemic to Ethiopia living in multi‐level social groups (Snyder‐Mackler, Beehner, and Bergman 2012) and characterized by a derived and rich communicative repertoire, in terms of both vocal (Gustison, le Roux, and Bergman 2012; Pedruzzi et al. 2024) and facial (Lazow and Bergman 2020; Leone, Ferrari, and Palagi 2014; Palagi and Mancini 2011) signals. The species is an optimal model to study YC as they show a great variability in yawning production. Indeed, they emit different morphological variants of yawns (yawns with covered teeth, Y1, with uncovered teeth, Y2, and with uncovered teeth and gums, Y3, Palagi et al. 2009) and, exceptionally, together with humans (Massen, Church, and Gallup 2015; Norscia et al. 2020) they are the only primate known to emit a specific vocalization while yawning (Gustison, le Roux, and Bergman 2012; Pedruzzi et al. 2024), although the function of such vocalization still remains unknown. In geladas, seeing others' yawns is contagious (Gallo et al. 2021; Palagi et al. 2009), as well as just hearing a yawn sound (playback experiment, Pedruzzi et al. 2024). Thus, we predict to confirm the role of both visual and auditory components in YC during spontaneous interactions (*Prediction 1*).

In some multimodal signals, the different components (e.g., visual and acoustic) convey identical information and their combination can enhance the precision of receiver responses and acts as a safeguard against imperfect sender coding (Fröhlich and van Schaik 2018; Hobaiter, Byrne, and Zuberbühler 2017; Moller and Pomiankowski 1993), specifically under various types of environmental and social noise (e.g., other vocalizations by conspecifics) (Hebets and Papaj 2005). For other signals, the different modalities convey distinct information, leading to a cumulative increase in overall information content (Hobaiter, Byrne, and Zuberbühler 2017; Johnstone 1996). The picture for yawns is not clear in humans, where experimental data seems to suggest that bimodal yawns (heard and seen yawns) are indeed more contagious than unimodal ones (only heard or only seen) (De Weck et al. 2022), but naturalistic data do not support such difference (Norscia and Palagi 2011). If multimodality has a cumulative effect in making gelada yawns more conspicuous, we expect heard and seen yawns to be more contagious compared to only seen or only heard ones (*Prediction 2*).

Drawing from previous findings in geladas (Palagi et al. 2009) and from the link posited between the phenomenon and empathic propensities (Campbell and de Waal 2014; Clay, Palagi, and de Waal 2018; Franzen, Mader, and Winter 2018), we predict the strength of the social bond (measured via grooming) to positively correlate with the tendency for yawn contagion susceptibility (*Prediction 3a*). Considering gelada yawn complexity in terms of modality (vocalized and not‐vocalized yawns) and morphology (Y1, Y2, Y3) (Palagi et al. 2009), the social modulation of YC might extend beyond the likelihood of responding to others' yawns. Indeed, when YC occurs, we expect a higher degree of modality matching (i.e., both receiver and trigger emitting a vocalized or not vocalized yawn) (*Prediction 3b*) and morphological mirroring (i.e., both receiver and trigger producing the same yawn morphological type) (*Prediction 3c*) between subjects with stronger social bonds. The socio‐communicative functions of spontaneous and contagious yawning (Guggisberg et al. 2011) as well as its possible role in motor alignment (Casetta, Nolfo, and Palagi 2021) would predict groupmates with a high degree of motor synchronization or showing cooperative interactions to be affected by YC at high rates (Ake and Kutsukake 2023; Casetta, Nolfo, and Palagi 2021). For instance, yawns produced by subjects having a central role in the group dynamics have been reported to be especially contagious (e.g., females in bonobos, Demuru and Palagi 2012; males in chimpanzees, Massen, Vermunt, and Sterck 2012) possibly for the higher adaptive value of aligning with their behaviors (Ostner, Wilken, and Schülke 2021). The basal core unit of gelada societies is either the one‐male (OMU) or the all‐male unit (AMU) (Snyder‐Mackler, Beehner, and Bergman 2012), and intra‐unit relationships are characterized by notable levels of cohesion and affiliation (Matsuda et al. 2012). In particular, gelada groups have been described as female‐bonded societies with females showing high rates of affiliation (Pallante et al. 2019; Tinsley Johnson et al. 2014), social play (Mancini and Palagi 2009), and agonistic support (Pallante, Stanyon, and Palagi 2016). Moreover, the group cohesion seems linked to female ingroup bonding, as, despite the centrality of gelada males in the group dynamics (Matsuda et al. 2012), there are reports of groups persisting even in the absence of the leader male (Snyder‐Mackler, Beehner, and Bergman 2012). Here, we predict yawns emitted by females to be more contagious than those emitted by males, especially for other females (*Prediction 4a*), possibly to foster affiliation and synchronization. Moreover, we also predict that female receivers show a higher degree of modality matching (*Prediction 4b*) and morphological mirroring (*Prediction 4c*) in response to other female compared to male trigger yawners.

Finally, the possible emotional valence conveyed by yawns is still under debate (Massen and Gallup 2017). Stimuli processing is lateralized across primate and nonprimate species according to the familiarity or the emotional valence of the stimulus itself (Gainotti 2022; Rogers and Vallortigara 2021). As emotional stimuli are known to be differently processed according to the perception side (Gainotti 2022), if yawning per se conveys a non‐neutral emotional valence, we would expect that the face side (i.e., left vs. right) with which the receiver detects yawn visual component could influence its contagiousness. Thus, we would expect that according to the side of detection, yawns may be differently contagious (*Prediction 5*), an aspect, to our knowledge, never previously investigated.

## 2 Methods

### 2.1 Ethics Statement

The study involved recording geladas from a distance, with no direct contact or manipulation of the animals. This recording process adhered to American Society of Primatologists Principles for the Ethical Treatment of Nonhuman Primates (e.g., maintaining an appropriate recording distance, avoiding any kind of distress for the animals). Consequently, the ethics committee of the University of Pisa waived the requirement for a permit.

### 2.2 Study Group, Data Collection, Video Analyses

Data collection took place in April and May 2023 (7 days a week over 10‐h periods, 08:30 a.m.–1 p.m. and 3 p.m.–8 p.m.) at NaturZoo Rheine (Germany), where the world largest gelada (*Theropithecus gelada*) colony is housed (Palagi and Bergman 2021) comprising 103 individuals (social housing condition: Continuous Full Contact, group). During the data collection, the colony was divided into two enclosures. The first enclosure consisted of two OMUs, while the second enclosure comprised two OMUs and one AMU (Table S1). The two enclosures were physically separated by a water pond, allowing animals in both enclosures to see and hear each other but preventing any physical contact. Following EEP gelada program guidelines to prevent inbreeding in the population, the adult males and some of the subadults of one enclosure (G2, Table S1) were moved to other zoos, leading to two periods of data collection (*Period*: pre vs. postremoval) in the colony. The enclosures were characterized by both indoor spaces (36 m^2^) and large outdoor areas (2700 m^2^) (Pedruzzi et al. 2024), where the animals could freely move. The animals were provided with grass, vegetables, and pellets twice a day (9:30 a.m., 2:30 p.m.). Water was always available. All study subjects (all adults as well as most subadults of the group) were individually identified through distinctive features. Three observers (LP, PO, MF) recorded the colony (SONY Handycam Full HD, FDR‐AX43A with directional microphones Sennheiser MKE600) spread in different sections of the enclosure to concurrently monitor the entire group. Even though we cannot exclude slight changes in the video‐frame size due to differences in the zoom used while recording, we generally kept our zoom setting so that we could record all geladas around the trigger yawner at a maximal range of about 10 meters. Via all‐occurrences sampling (Altmann 1974), we recorded social interactions (e.g., affiliation, proximity, yawning events) by randomly following subgroups of subjects visible to the observers. We obtained 230 h of recordings and calculated individual recording time through 3‐min scan sampling during video analyses. For each animal, we summed all the scans in which each subject was present and obtained the proxy data of its recording time by multiplying this number by three (e.g., 240 scans in which a subject is present = 720 min of observation) (Facondini Pedruzzi et al. 2024) (mean individual recording time ± SD: 5.8 ± 3.1 h). We analyzed videos using PotPlayer to record the exact occurrence and duration of the behavioral patterns of interest (accuracy: 0.02 s). More in detail, for each yawn, we coded its exact time of occurrence, the identity of the yawner, the morphological variant of the yawn (yawn types: Y1, Y2, Y3, Palagi et al. 2009), if it was vocalized (Pedruzzi et al. 2024), its duration, the identity and the number of subjects around the trigger subject, and if the trigger yawn was detected by any of the receivers and the type of detection (not detected, only heard, only seen, seen and heard), and, if visually perceived, the side of detection (left or right) for each observer.

### 2.3 Operational Definitions

*Detection*. We coded if yawns were detected for each receiver subject. As geladas can produce both vocalized and nonvocalized yawns, we have four distinct conditions for the detection of yaws (Figure 1b). For yawns emitted *without vocalization*, we can have the *No detection* condition, occurring when the face of the potential responder turned 180° away from the trigger or when an obstacle prevented the potential receiver from seeing the trigger yawn (Norscia et al. 2020). Geladas, like most primates, are characterized by high orbit convergence and large binocular visual field (Heesy 2004). Here, we define a not‐vocalized yawn as *only seen* if the trigger yawn was in the visual field of the observer (Figure 1b). All doubtful cases (*n* = 16) were excluded. For yawns emitted *with vocalization*, we can have the *Seen and heard* condition if the yawn is both visually and audibly detectable. As gelada yawn vocalizations are relatively loud and generally audible at 40 meters at least (Gallo et al. 2021; Pedruzzi et al. 2024), we are confident that all the awake subjects present in the videoframe could hear vocalized yawns. Vocalized yawns were *Only heard* when they could be heard but not seen. We considered all yawn occurrences and trigger‐receiver dyads within the same group unit to avoid biases in auditory vs visual yawn contagion, as vocalized yawns can be heard also at the intergroup level due to their loudness; all coded yawns and trigger‐receiver dyads were thus within a 10‐meter range and video‐recorded.

*Response*. We coded as yawn responses all yawns emitted by receiver subjects in the 3 min following the trigger yawn. We opted for a 3‐min time window in line with the literature on YC in geladas (Gallo et al. 2021; Leone, Ferrari, and Palagi 2014; Palagi et al. 2009; Pedruzzi et al. 2024). In the event of a response, we coded the identity of the responder, the morphological type of yawn, whether the yawn type of the receiver matched the trigger yawn type (mirror response) as well as if it matched the modality of the trigger yawn (e.g., if both trigger and response yawn are vocalized/not vocalized). All the subjects that could not be visible and recorded throughout the 3‐min time window following the trigger yawn (e.g., getting far from the observers) were excluded from the analyses. Moreover, all receivers who perceived more trigger yawns produced by different subjects before responding (*n* = 47 yawns) were excluded from the analyses due to the uncertainty in assigning the response yawn to one of the trigger yawns perceived.

*Number of subjects in the audience*. For each trigger yawn, we coded the total number of subjects present in the audience (*Audience size*), thus within a maximal range of approximately 10 meters.

*Side of detection*. For each receiver who visually detected trigger yawns, we coded the side of detection. We classified a yawn as detected from the left if it was exclusively perceived by the visual field of the left eye, and detected from the right if it was exclusively perceived by the visual field of the right eye (Figure 1b). All doubtful cases were excluded for parsimony. For this analysis, yawns associated with clearly negative events (i.e., aggression, self‐scratching) were excluded to avoid possible differences in contagiousness due to external negative contextual factors and not to the yawn itself.

*Grooming index* and *Proximity index*. We calculated grooming and proximity index to evaluate the strength of the relationship between two individuals. To calculate these two indices, we used the scan‐sampling method (Lehner 1992): every 3 min, we identified all the subjects present in the videoframe, and documented their grooming and proximity activities. For *Grooming index*, we divided the number of scans in which two subjects groomed each other (independently from grooming direction) by the number of scans in which both the individuals were present. For the *Proximity index*, we divided the number of scans in which an individual was in proximity (two individuals sitting at a distance that does not exceed that of an outstretched limb) with another specific individual by the number of scans in which both the individuals were present. We did this for all possible dyads of the colony. We included both indices as spatial proximity among females seems less indicative of social bonding, as geladas frequently show high levels of spatial overlap with ingroup individuals (Tinsley Johnson et al. 2014). Moreover, by controlling for the proximity levels between subjects, we can control whether YC is biased by different levels of social bonding or of detection probability (see Gallup 2021; Massen and Gallup 2017) (e.g., the more often two subjects are in proximity, the likelier for them to be in close visual contact), as in Palagi et al. (2009).

*Receiver frequency of spontaneous yawning*. To control for differences in yawn contagion being merely due to individual differences in yawning rates, we calculated the individual spontaneous yawn frequency (yawns/hour) as follows: number of spontaneous yawns (derived from the total yawns performed by an individual minus those emitted following the detection of others' yawns in a time‐window of 3 min) divided by the recording time for the individual.

*Inter‐coder agreement*. Inter‐coder reliability between LP and PO was assessed independently on approximately 15% of the videos using Cohen's Kappa coefficient (Cohen 1968), consistently achieving a value greater than 0.80 (subject identification, *K* = 0.86; yawn presence, yawn type, modality, *K*_average_ = 0.90; detection *K*_average_ = 0.87; grooming and proximity, *K*_average_ = 0.97).

### 2.4 Statistical Analyses

Model 1–*Yawn contagion*. We ran the first GLMM with *Yawn response* as response variable (presence/absence, binomial error distribution), with each model observation being a trigger‐receiver pair for each yawning event. The interaction between the identity of *Trigger* and *Receiver* subjects and the *Trigger yawn ID*s were included as random factor, whereas the *Receiver spontaneous yawn frequency* and the *Period* were included as control factor. The fixed factors considered were: (i) *Detection* (No detection/Only heard/Only seen/Heard and seen), (ii) Trigger sex*Receiver sex, (iii) *Trigger sex*, (iv) *Receiver sex*, (v) *Grooming index*, (vi) *Proximity index*, (vii) *Type of trigger yawn* (Y1/Y2/Y3), (viii) *Duration of trigger yawn* (seconds), (ix) *Audience size*.

Model 2–*Yawn modality matching*. To understand which factors predict the likelihood of matching the modality of the trigger yawn (e.g., producing a vocalized/not‐vocalized yawn in response to a vocalized/not‐vocalized trigger yawn), we ran a GLMM with *Yawn modality matching* as response variable (presence/absence, binomial error distribution). The interaction Trigger*Receiver subjects was included as random factor, and the *Period* was included as control factor. The fixed factors considered were: (i) Trigger sex*Receiver sex, (ii) *Trigger sex*, (iii) *Receiver sex*, (iv) *Grooming index*, (v) *Proximity index*.

Model 3–*Yawn type mirroring*. To understand which factors predict the likelihood of mirroring the trigger yawn type (e.g., producing a type 3 yawn in response to a type 3 trigger yawn), we ran a GLMM with *Yawn type mirroring* as response variable (presence/absence, binomial error distribution). The interaction Trigger*Receiver subjects was included as random factor, and the *Period* was included as control factor. The fixed factors considered were: (i) Trigger sex*Receiver sex, (ii) *Trigger sex*, (iii) *Receiver sex*, (iv) *Grooming index*, (v) *Proximity index*.

Model 4–*Laterality in YC*. To understand if the side of detection (left/right) predicts the likelihood of responding to a trigger yawn, we ran a GLMM with *Yawn response* as response variable (presence/absence, binomial error distribution). The interaction Trigger*Receiver was included as random factor. The control factors considered were: (i) Trigger sex*Receiver sex, (ii) *Trigger sex*, (iii) *Receiver sex*, (iv) *Grooming index*, (v) *Proximity index*, (vi) *Type of trigger yawn*, (vii) *Duration of trigger yawn*, (viii) *Number of subjects in the audience*. The fixed factor considered was the *Detection side* (Left/Right). Due to the small sample size, we could not test this at the individual level.

All the analyses were carried out using R_Studio_ (http://www.r-project.org). Multicollinearity in the GLMMs was assessed using the *check_collinearity* function (package *performance* 0.4.4) through Variance Inflation Factors (VIFs). *Low correlation* was found for all the fixed factors included in the models (VIF: 1.04–2.02). The significance of the models was evaluated by comparing the full model against a null or control model consisting only of random effects (and control factors) using the Likelihood Ratio Test (LRT) with the *Chisq* test argument (Dobson and Barnett 2018). To determine the predictors p‐value, LRTs were conducted between the full model and a model lacking that specific predictor, using *ANOVA* function (Barr et al. 2013). To calculate marginal and residual R^2^ values, we used the *MuMIn* package version 1.43.17 (Barton 2020). Relative odds ratios were used to illustrate the impact of estimated effects, using the *confint()* function, where odds ratios (OR) represent the expected change in odds when all variables are held at reference values, and the fixed factor increases by one unit or change categorical level. For pairwise comparisons with factors with more than two levels (and for interaction factors), we used the package *emmeans* to perform the Tukey test (Bretz, Hothorn, and Westfall 2016; Lenth et al. 2021). To assess model fit and potential overdispersion, we used the DHARMa package (version 0.3.3.0, Hartig 2020). The GLMMs showed no overdispersion (dispersion range: 0.828–1, *p*‐value range: 0.491‐1), no outliers were detected (*p*‐value range = 0.768–1), and normality of the residuals was confirmed through visual inspection of Q‐Q plots (Kolmogorov‐Smirnov test, *p*‐value range: 0.303–0.623).

## 3 Results

*Descriptive results on spontaneous yawning* ‐ We recorded a total of 1422 yawns (both vocalized and nonvocalized yawns) produced by 67 individually recognized subjects of our group. The average frequency of spontaneous yawning (excluding yawns produced in response to others' yawns) was 2.92 yawns/hour (SD: 3.13 yawns/hour) and differed according to sex (Wilcoxon signed‐rank test, unpaired data, *W* = 183.5, *p* = 0.007) with males yawning more frequently than females. While yawning, males vocalized in the 69.6% of events (388/557), whereas females in the 18% of cases (175/969). Figure S1 depicts the number of vocalized vs nonvocalized yawns (i.e., modality of yawn responses) produced in response to trigger yawns in the three types of detection conditions (only heard, only seen, seen and heard). Yawns had an average duration of 2.74 s (SD: 1.64 s, range: 0.5–9.8 s); females produced slightly longer yawns compared to males (mean ± SD: 1.94 ± 0.88 vs. 2.23 ± 1.03 s, Wilcoxon signed‐rank test, unpaired data, *W* = 202397, *p* < 0.0001) and vocalized yawns were slightly shorter than nonvocalized ones (mean ± SD, silent yawns: 2.24 ± 1.20 s, vocalized yawns: 2.01 ± 0.91 s, Wilcoxon signed‐rank test, unpaired data, *W* = 1472090, *p* < 0.0001). The three morphological variants of yawns (Y1, Y2, Y3) were differentially emitted by males (25.6% Y1, 16% Y2, 58.3% Y3) and females (69% Y1, 4% Y2, 27% Y3), with males producing more often yawns with exposed gums and teeth while females producing more often yawns with covered teeth.

**Figure 2:**
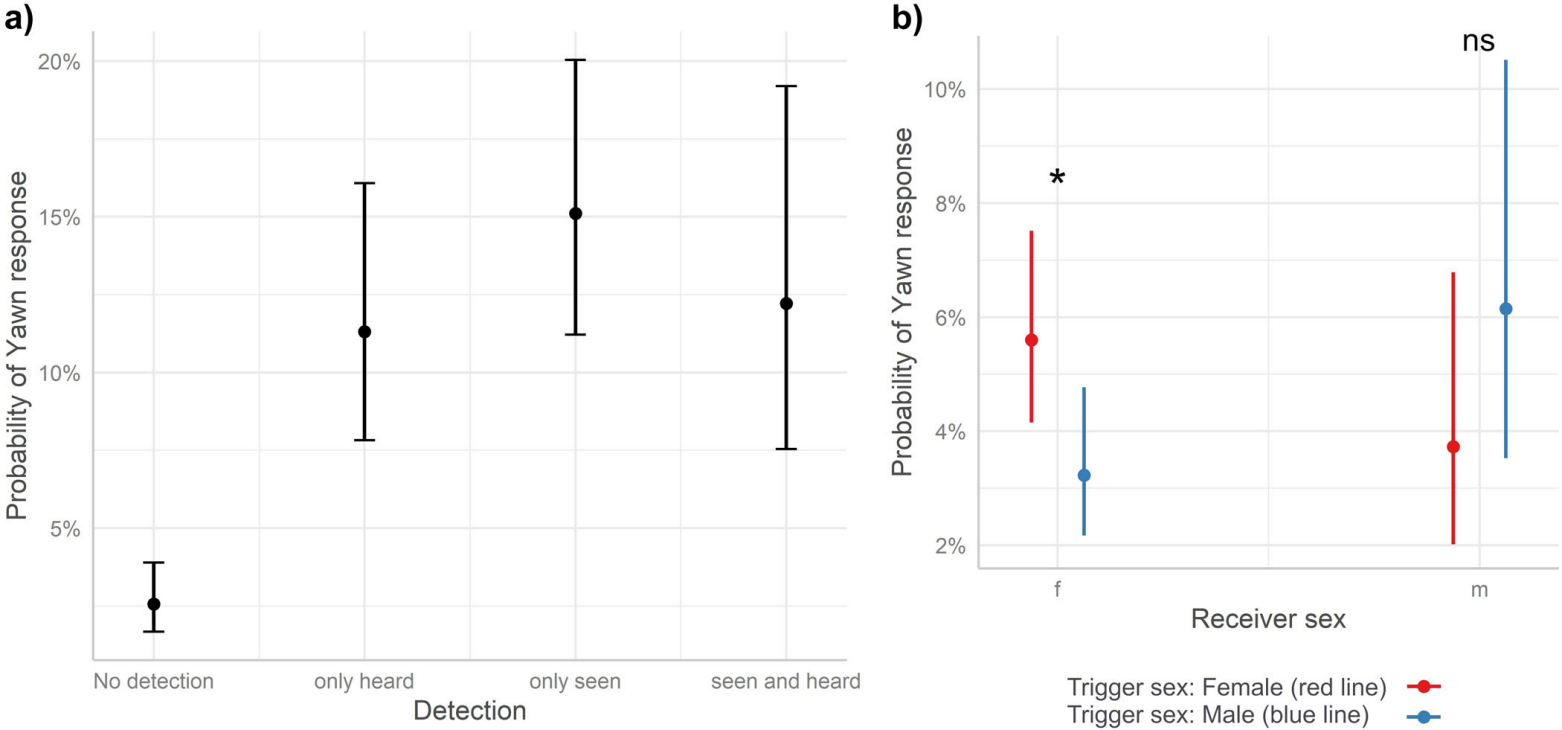
(a) Effect plot showing the significant effect of the *Detection* on the likelihood of yawn response (*X* ^2^ = 93.23, *p* < 0.0001, Tukey test results: df = 3243, No detection vs. Only heard: *t*‐ratio = −7.018, *p* < 0.0001; No detection vs. Only seen: *t*‐ratio = −9.362, *p* < 0.0001; No detection vs. Seen and heard: *t*‐ratio = −5.927, *p* < 0.0001). (b) Effect plot showing the significant interaction between the *Receiver sex* and the *Trigger sex* on the likelihood of yawn response (for female receivers, Male trigger vs. Female trigger: df = 3243, *t*‐ratio = 2.585, *p* = 0.048; for male receivers, Male trigger vs Female trigger: df = 3243, *t*‐ratio = −2.225, *p* = 0.11, 0.117).

Model 1–*Yawn contagion*. Number of observations for the model = 3261. Random factor: *Trigger* (*n* = 62)*Receiver (*n* = 61). The full model investigating the factors affecting the likelihood of responding to others' yawns significantly differed from the control one (*X* ^2^_12_ = 145.4, *p* < 0.0001). The variables *Detection* and Trigger sex*Receiver sex significantly affected the likelihood of yawning. Specifically, the likelihood of yawning was higher after seeing, hearing or seeing and hearing yawns compared to when the yawn was not detected (*X* ^2^ = 93.23, *p* < 0.0001, Tukey test results: df = 3243, No detection vs. Only heard: *t*‐ratio = −7.018, *p* < 0.0001; No detection vs. Only seen: *t*‐ratio = −9.362, *p* < .0001; No detection vs. Seen and heard: *t*‐ratio = −5.927, *p* < 0.0001, Figure 2a, Table 1a), whereas, overall, no difference was detected between the levels Only seen vs Only heard, Only seen versus Seen and heard, Only heard versus Seen and heard. Females responded more to female compared to male trigger yawns (for female receivers, Male trigger versus Female trigger: df = 3243, *t*‐ratio = 2.585, *p* = 0.048, Figure 2b, Table 1a). A similar but not significant tendency seem to emerge also for male‐male trigger‐receiver dyads (Figure 2b). *Receiver sex, Trigger sex, Type of trigger yawn, Duration of trigger yawn, Grooming index*, and *Proximity index* did not affect the likelihood of yawning in response to a trigger yawn (Table 1a).

**Table 1:** Estimated parameters (Coeff), Standard Error (SE), and results of the Likelihood Ratio Tests (*χ* ^2^) of the GLMMs.

Fixed effects	Coeff.	SE	*χ* ^2^	df	*p*
a ***Model 1** ‐ Yawn contagion*					
Intercept	−3.274	0.361	—	—	—
**Tested variables**					
Detection	**—**	**—**	93.226	3	**0.000**
Only heard	1.579	0.225	—	—	**—**
Only seen	1.912	0.204	—	—	**—**
Seen and heard	1.667	0.281	—	—	**—**
Trigger sex (m)	−0.576	0.223	2.587	1	0.108
Receiver sex (m)	‐0.427	0.328	0.525	1	0.469
Trigger sex*Receiver sex	1.102	0.376	8.604	1	**0.003**
Grooming index	−0.217	0.656	0.110	1	0.740
Proximity index	−0.693	1.057	0.430	1	0.512
Type of trigger yawn	**—**	**—**	0.525	2	0.769
Type T2	−0.221	0.310	—	—	—
Type T3	−0.065	0.182	—	—	—
Duration of trigger yawn	−0.127	0.084	2.290	1	0.130
**Control variables**					
Receiver spontaneous yawn frequency	10.635	1.958	29.487	1	**0.000**
Period	−0.011	0.164	0.0049	1	0.944
Audience size	−0.065	0.024	7.414	1	**0.006**
*N*_observations_ = 3261, *N*_Trigger_ = 47, *N*_Receiver_ = 63. Random factors: Trigger, Variance = 4.765*10^−^^10^, SD = 2.183*10^−^^5^; Receiver, Variance = 1.128*10^−^^8^, SD = 1.062*10^−^^4^; Yawn ID, Variance = 0.292, SD = 0.540					
b ***Model 2 ‐** Yawn modality matching*					
Intercept	1.121	0.414	—	—	—
**Tested variable**					
Trigger sex (m)	−1.800	0.448	12.053	1	**0.000**
Receiver sex (m)	−0.649	0.516	0.026	1	0.873
Trigger sex*Receiver sex	1.495	0.739	4.094	1	**0.043**
Grooming index	−0.064	1.426	0.002	1	0.964
Proximity index	−0.546	2.511	0.047	1	0.828
**Control variables**					
Period	0.123	0.509	0.059	1	0.808
*N*_observations_ = 229, *N*_Trigger_ = 47, *N*_Receiver_ = 63. Random factors: Trigger, Variance = 0.306, SD = 0.553; Receiver, Variance = 5.375*10^−^^9^, SD = 7.331*33110^−^^5^					
c ***Model 3 ‐** Yawn type mirroring*					
Intercept	0.379	0.333	**—**	**—**	**—**
**Tested variables**					
Trigger sex (m)	−1.415	0.334	10.440	1	**0.001**
Receiver sex (m)	−1.406	0.484	1.356	1	0.244
Trigger sex*Receiver sex	2.040	0.691	8.708	1	**0.003**
Grooming index	1.166	1.304	0.800	1	0.371
Proximity index	1.368	2.252	0.369	1	0.543
**Control variables**					
Period	0.106	0.467	0.051	1	0.821
*N*_observations_ = 229, *N*_Trigger_ = 47, *N*_Receiver_ = 63. Random factors: Trigger, Variance = 1.604*10^−^^9^, SD = 4.005*10^−^^5^; Receiver, Variance = 1.057*10^−^^9^, SD = 3.251*10^−^^5^					

*Note:* Significant *p* values are in bold. Estimate ± SE refers to the difference of the response between the reported level of this categorical predictor and the reference category of the same predictor.

Abbreviations: df, degree(s) of freedom; ‐, not applicable.

**Figure 3:**
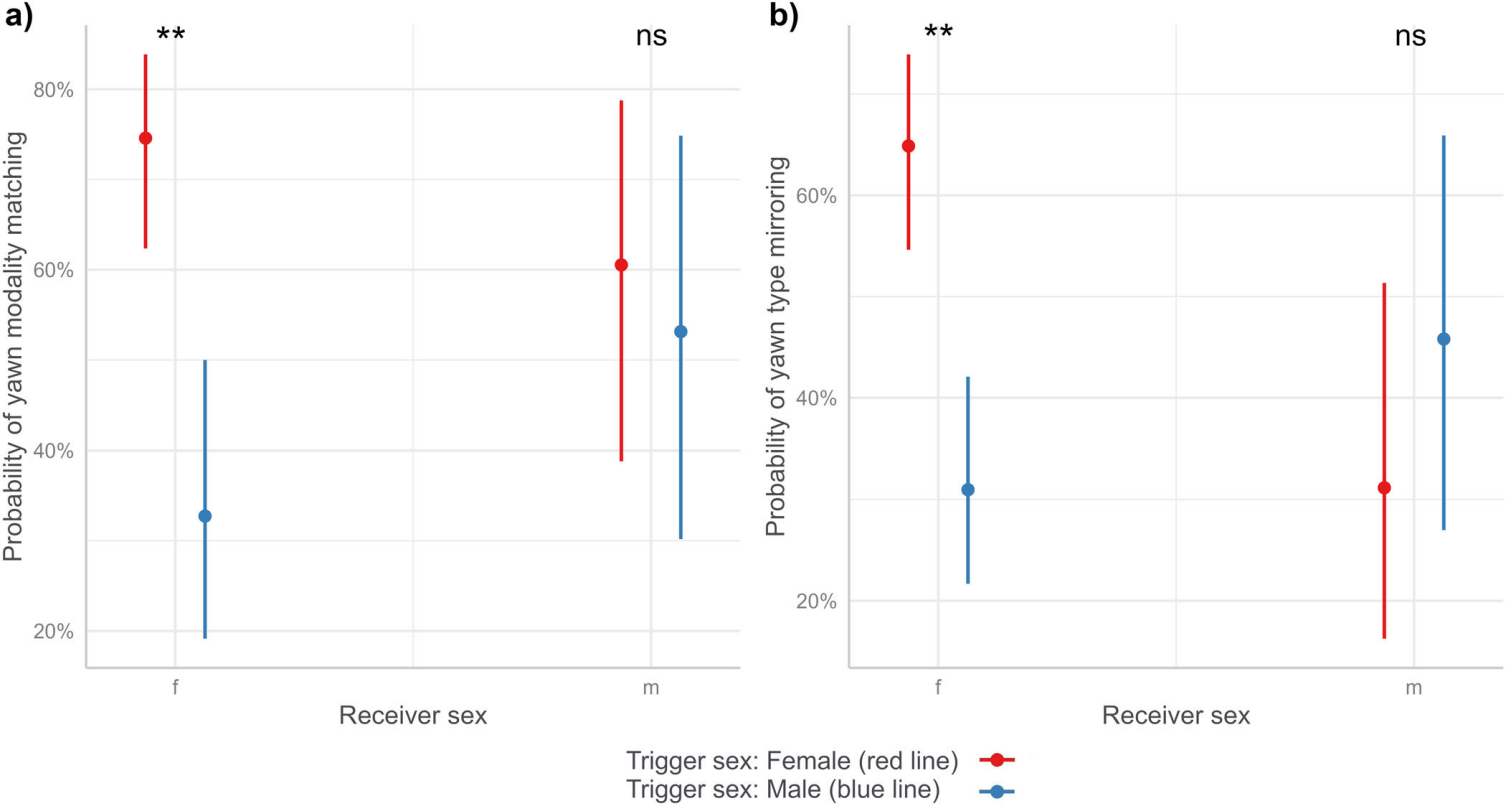
Effect plot showing the significant interaction between the *Receiver sex* and the *Trigger sex* on the likelihood of (a) yawn modality matching (for female receivers, Male trigger vs. Female trigger: df = 220, *t*‐ratio = 4.010, *p* = 0.0005; for male receivers, Male trigger vs. Female trigger: df = 220, *t*‐ratio = 0.450, *p* = 0.970), and (b) yawn type mirroring (for female receivers, Male trigger vs. Female trigger: df = 220, *t*‐ratio=4.238, *p* = 0.0002; for male receivers, Male trigger vs. Female trigger: df = 220, *t*‐ratio = −1.023, *p* = 0.736) between the trigger and the receiver yawns.

Model 2–*Yawn modality matching*. Number of observations for the model = 229. Random factor: *Trigger* (*n* = 47)*Receiver (*n* = 63). The full model significantly differed from the null one (*X* ^2^_5_ = 11.96, *p* = 0.035). The variables *Trigger sex* and Trigger sex*Receiver sex significantly affected the likelihood of yawning. Specifically, females mirrored the trigger yawn modality towards female yawns compared to male yawns (for female receivers, Male trigger vs. Female trigger: df = 220, *t*‐ratio = 4.010, *p* = 0.0005, Figure 3a, Table 1b). *Receiver sex, Grooming index* and *Proximity index* did not affect the likelihood of modality matching (Table 1b).

Model 3–*Yawn type mirroring*. Number of observations for the model (excluding only heard trigger yawns) = 229. Random factor: *Trigger* (*n* = 47)*Receiver (*n* = 63). The full model investigating the factors affecting the likelihood of mirroring the morphological variant of the trigger yawn significantly differed from the null one (*X* ^2^_5_ = 24.18, *p* = 0.0002). The variables *Trigger sex* and Trigger sex*Receiver sex significantly affected the likelihood of yawn type matching. Female receivers produced the same morphological variant of the trigger yawn more often in response to female triggers compared to males (for female receivers, Male trigger vs Female trigger: df = 220, *t*‐ratio = 4.238, *p* = 0.0002, Figure 3b, Table 1c). *Receiver sex, Grooming index* and *Proximity index* did not affect the likelihood of yawn type mirroring (Table 1c).

Model 4–*Laterality in YC*. Number of observations for the model = 283. Random factor: Trigger subject (*n* = 50) * Receiver subject (*n* = 54). The full model investigating whether the side of detection of yawns could affect the likelihood of yawn contagion did not differ from the control model (*X* ^2^ = 0.37, *p* = 0.54).

## 4 Discussion

In the present study we investigated the effect of multimodality and social factors on yawn contagion (YC) in a large zoo‐housed colony of geladas (*Theropithecus gelada*). Geladas are the only nonhuman species known to produce a distinct vocalization associated with yawning; thus, they represent a good model to study the evolution of human yawning complexity and, more generally, of bimodal signals. First, our data confirm previous evidence for visual YC in geladas in captivity (Palagi et al. 2009), with a larger sample size and new analytical techniques (*Prediction 1* supported). Yawn vocal cues were here capable to elicit a corresponding motor action through a different sensory channel, as only hearing yawn sound was enough to elicit a yawning response (*Prediction 1* supported), consistently with recent experimental data on the same group (Pedruzzi et al. 2024). Behavioral contagion can indeed occur through sensory channels beyond the visual one (Ferrari et al. 2005), even though relatively limited research effort has been dedicated to vocal cues in motor resonance phenomena. Hearing, seeing, or hearing and seeing yawns triggered contagion at comparable levels (*Prediction 2* not supported), similarly to naturalistic data on humans (Norscia et al. 2020), and in contrast with experimental evidence (De Weck et al. 2022). This suggests that one sensory modality is enough to automatically trigger a response in the receiver. Even though geladas, like most cercopithecines, are visually‐oriented (Waller et al. 2024), they exhibit highly developed acoustic communication (Gustison, le Roux, and Bergman 2012), and possibly a greater reliance on acoustic cues linked to the need for maintaining bonds within the reproductive unit in complex social environments. Geladas live in multilevel social groups where several group units often co‐feed and move together, creating high environmental and social noise (e.g., frequent vocalizations as well as intra‐ and intergroup interactions), which means groupmates are frequently only in vocal or visual contact (Snyder‐Mackler, Beehner, and Bergman 2012). These communicative and social features of the species could explain why the sound of a yawn can be as contagious as the visual component of yawn in geladas, as well as why unimodal yawns are processed with equal efficacy as multimodal yawns. The evolution of multimodality in a signal can indeed improve the ease and the frequency with which an individual can share information with other conspecifics and ensure detection (Fröhlich and van Schaik 2018; Hebets and Papaj 2005). To better understand the evolutionary meaning of yawn vocalizations, future studies will first need to address the contextual or affective factors inducing, for some yawns, a vocalization.

Unlike previous evidence in the species (Palagi et al. 2009), we found no association between YC and the social bond between trigger and responder subjects (measured via grooming) (*Prediction 3a, 3b, 3c* not supported). This result calls for the need of intergroup comparisons and complicates the already intricate picture of the link betweenF relationship quality and yawn contagion (Massen and Gallup 2017; Palagi et al. 2020), an aspect which received inconsistent evidence across studies also within the same species (bonobos: Demuru and Palagi 2012; Norscia et al. 2022; domestic dogs: Joly‐Mascheroni, Senju, and Shepherd 2008; Kis et al. 2020; Neilands et al. 2020; O'Hara and Reeve 2011). In our case, it might either be that other factors are at play and modulate the phenomenon and should be integrated when measuring relationship quality in geladas (e.g., kin relationships, agonistic support, grooming reciprocity, embracing behavior, help in offspring care). Moreover, when dealing with large samples, a more intensive sampling of grooming interactions might be useful to have finer measures of relationship quality for all possible dyads of subjects. On the other hand, a consistent sex effect was found here, as females exhibited a heightened response when triggered by other females (*Prediction 4a* supported). This is in line with evidence indicating that yawns produced by socially relevant subjects (e.g., dominant, relevant sex) might be especially contagious (Demuru and Palagi 2012; Massen, Vermunt, and Sterck 2012). However, nonsignificant sex‐effects in yawn contagiousness are also often found, indicating high inter‐study and intergroup variability in the study of yawn contagion (e.g., geladas, Gallo et al. 2021; spider monkeys, Valdivieso‐Cortadella et al. 2023; chimpanzees, Campbell and Cox 2019; humans, Gallup and Massen 2016). In addition, here female‐female trigger‐responder dyads also showed the highest modality matching (*Prediction 4b s*upported) and morphological mirroring (*Prediction 4c* supported). The matching of specific behavioral types has also been observed in stretching type matching in budgerigars (Miller et al. 2012). Female‐female communication and affiliation is indeed crucial for the cohesion and affiliation characterizing gelada intra‐unit relationships (Matsuda et al. 2012; Pallante et al. 2019; Tinsley Johnson et al. 2014), and adult females play a central role in maintaining within‐OMU stability (Leone and Palagi 2010; Snyder‐Mackler, Beehner, and Bergman 2012). The stronger mirroring shown by female trigger‐receiver dyads supports the role of YC in improving synchronization and intra‐sex affiliation (Ake and Kutsukake 2023; Casetta, Nolfo, and Palagi 2021; Palagi et al. 2009; Poole and Henderson 2023). The prominent position of females might necessitate a heightened receptiveness to social cues, like yawning, which could promote synchronization among individuals and enhance social unity (Palagi et al. 2009). Although here we did not directly investigate the potential effect of the trigger hierarchical rank on yawn contagion, dominance ranks may also enhance behavioral contagion, as the increased emotional attachment and the heightened salience of signals produced by high‐status individuals have been proposed as explanations for this bias in several phenomena of motor resonance (Demuru and Palagi 2012; Facondini, Pedruzzi et al. 2024; Iki and Kutsukake 2021; Massen, Vermunt, and Sterck 2012; Ostner, Wilken, and Schülke 2021). Finally, we did not find laterality of YC at group level (*Prediction 4* not supported), possibly implying that either yawns might not be per se be processed as emotionally valent stimuli (Gainotti 2022), or either that yawn contagion goes beyond simple emotional mechanisms, being a more polyvalent and context‐dependent behavior. Naturally, the absence of the effect from our results does not indicate the absence of the phenomenon, as it might be due to some limits of our work, such as the lowly controlled conditions in such a naturalistic environment.

Overall, our results (Table 2) suggest that even though males seem to be central in the phenomenon as they produce more frequently multimodal yawn signals, when we focus on yawn contagion females seem to emerge as central characters in the species, possibly reflecting their importance in ingroup social dynamics. This underscores the need for further investigation into the role of females in the social dynamics of geladas and other primate species typically characterized by male dominance, where the role of females is often underestimated (Hrdy 2009). In conclusion, our study highlights the importance of analyzing different sensory components to elucidate the modulation and distribution of YC, especially in human and nonhuman species living in multi‐layered social systems. Our data raise further questions about the functional and emotional significance of yawns and their associated vocalizations in geladas compared to humans. Additionally, we highlight that not only contextual but also potential inter‐sexual differences might occur in the functions of yawning among group members, suggesting the phenomenon to be more complex than previously thought.

**Table 2:** Summary of hypotheses, predictions, and outcomes of the study.

Hypothesis		Prediction	Outcome
***Hypothesis 1***	Seeing (Palagi et al. 2009) and hearing (Pedruzzi et al. 2024) others' yawns is reported to be contagious in geladas	Both visual and auditory YC are present in our study group (***Prediction 1***)	***Supported***
***Hypothesis 2***	The different modalities of a multimodal signal have a cumulative increase in overall information content and response probability (Fröhlich and van Schaik 2018; Hobaiter, Byrne, and Zuberbühler 2017)	Bimodal yawns are more conspicuous and thus contagious than only seen or only heard yawns (***Prediction 2***)	***Not supported***
***Hypothesis 3***	YC has been reported to be a socially modulated phenomenon (Palagi et al. 2020)	The stronger the dyadic social bond between the trigger and receiver, the stronger YC, modality matching, and morphological mirroring (***Predictions 3a, 3b, 3c***)	***Not supported***
***Hypothesis 4***	Yawns emitted by socially relevant subjects are more contagious (Massen, Vermunt, and Sterck 2012) possibly to increase ingroup synchronization (Casetta, Nolfo, and Palagi 2021)	Yawns produced by females are more contagious, especially for other females, and elicit more modality matching and morphological mirroring (***Predictions 4a, 4b, 4c***)	***Supported***
***Hypothesis 5***	The processing of stimuli with non‐neutral emotional valence is lateralized across mammal species (Gainotti 2022)	Contagiousness differs according to the side (left/right) of yawn detection (***Prediction 5***)	***Not supported***

Abbreviation: YC, yawn contagion.

## Author Contributions

Luca Pedruzzi, Alban Lemasson, Elisabetta Palagi conceived the study. Luca Pedruzzi, Paolo Oliveri, Martina Francesconi collected the data. Luca Pedruzzi and Paolo Oliveri coded videos. Luca Pedruzzi carried out statistical analyses. Luca Pedruzzi, Paolo Oliveri, Martina Francesconi, Alban Lemasson, Elisabetta Palagi wrote the first draft of the manuscript.

## Conflicts of Interest

The authors declare no conflicts of interest.

## Supporting information

Supporting information.

Supporting information.

Supporting information.

Supporting information.

## Data Availability

The full datasets used for the present work have been uploaded as supplementary files.
