# Supplementary material for: Yawn Contagion and Modality‐Matching in the Female‐Bonded Society of Geladas (Theropithecus gelada)
Source: Am J Primatol. 2024 Dec 17;87(1):e23709. doi: 10.1002/ajp.23709 (PMC11652820; doi:10.1002/ajp.23709)

**Figure S1.** Graph depicting the modality of yawn responses (count numbers of vocalised vs non-vocalised yawn responses) according to the type of detection of the trigger yawn (only heard, only seen, seen and heard trigger yawns). YR = yawn response.


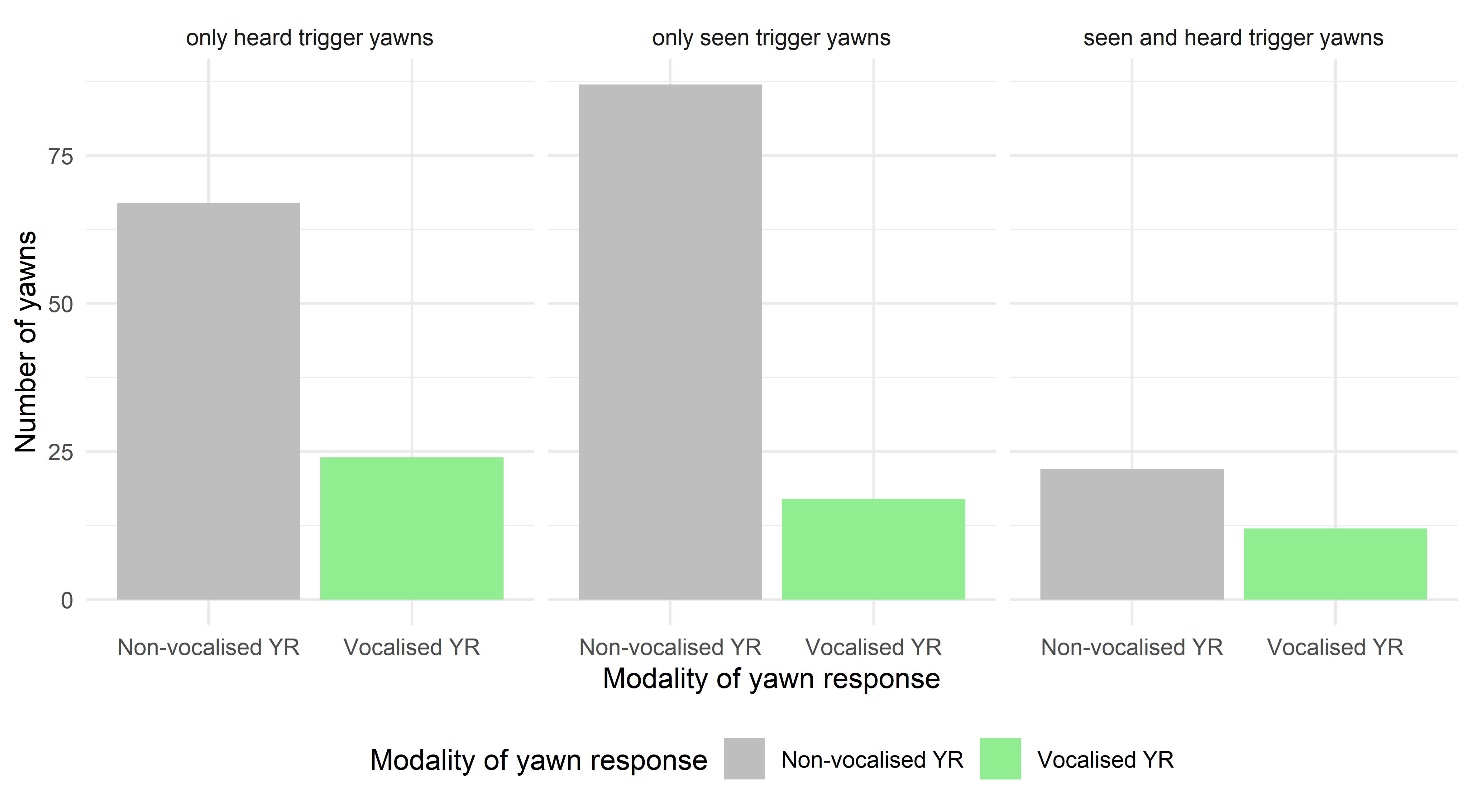

Supplement: Supplementary file 1 — Supporting information. [file AJP-87-e23709-s003.docx]
