# Supplementary material for: Yawn Contagion and Modality‐Matching in the Female‐Bonded Society of Geladas (Theropithecus gelada)
Source: Am J Primatol. 2024 Dec 17;87(1):e23709. doi: 10.1002/ajp.23709 (PMC11652820; doi:10.1002/ajp.23709)
Supplement: Supplementary file 2 — Supporting information. [file AJP-87-e23709-s004.docx]

**Table S1. a)** Compositions of the different group units at the time of data collection. We defined adults as individuals older than six years old, subadults as those between three and six years old, and infants as individuals less than three years old, following Palagi & Mancini (2011). **b)** Individually recognized geladas hosted at NaturZoo Rheine (April-May 2023).

**a)**

| ENCLOSURE | LEADER MALE OF THE UNIT | ADULT FEMALES | ADULT MALES | SUBADULT FEMALES | SUBADULT MALES | INFANTS |
| --- | --- | --- | --- | --- | --- | --- |
| G2 | Sumo | 20 | 1 | 4 | 3 | 2 |
| G2 | Enzo | 5 | 1 | 4 | 2 | 6 |
| G2 | Rocco | 0 | 2 | 0 | 7 | 0 |
| G1 | Biondo | 12 | 1 | 7 | 6 | 4 |
| G1 | Giangi | 6 | 1 | 5 | 2 | 1 |

**b)**

| **SUBJECTS** | **ENCLOSURE** | **OMU** | **SEX** | **AGE** | **TOTAL YAWNS PRODUCED** |
| --- | --- | --- | --- | --- | --- |
| Biondo | G1 | B | M | Adult | 122 |
| Barbie | G1 | B | F | Adult | 8 |
| Basic | G1 | B | F | Adult | 20 |
| Belly | G1 | B | F | Subadult | 6 |
| Bernoccolo | G1 | B | M | Subadult | 7 |
| Betta | G1 | B | F | Adult | 6 |
| Biba | G1 | B | F | Subadult | 1 |
| Bifida | G1 | B | F | Adult | 27 |
| Bigne | G1 | B | F | Adult | 21 |
| Bijoux | G1 | B | F | Subadult | 9 |
| Bisnonna | G1 | B | F | Adult | 23 |
| Black | G1 | B | M | Adult | 6 |
| Blanca | G1 | B | F | Adult | 14 |
| Blind | G1 | B | F | Adult | 26 |
| Borsa | G1 | B | F | Adult | 27 |
| Bortolo | G1 | B | M | Subadult | 9 |
| Braccio | G1 | B | F | Adult | 16 |
| Bratz | G1 | B | F | Adult | 35 |
| Giangi | G1 | G | M | Adult | 153 |
| Gatta | G1 | G | F | Adult | 12 |
| Gelly | G1 | G | F | Subadult | 0 |
| Gessica | G1 | G | F | Adult | 28 |
| Ghiozza | G1 | G | F | Adult | 14 |
| Ghirlanda | G1 | G | F | Adult | 15 |
| Gianna | G1 | G | F | Adult | 23 |
| Gigetto | G1 | G | M | Subadult | 12 |
| Gigio | G1 | G | M | Subadult | 1 |
| Grappolo | G1 | G | F | Adult | 30 |
| Enzo | G2 | E | M | Adult | 78 |
| E1m6 | G2 | E | F | Adult | 4 |
| E2m6 | G2 | E | F | Adult | 4 |
| Edera | G2 | E | F | Adult | 1 |
| Elly | G2 | E | F | Subadult | 1 |
| Evasa | G2 | E | F | Adult | 14 |
| Strip | G2 | E | F | Adult | 8 |
| Striscia | G2 | E | F | Adult | 5 |
| Sumo | G2 | S | M | Adult | 46 |
| Neomamma | G2 | S | F | Adult | 22 |
| Osso | G2 | S | F | Adult | 18 |
| Roll | G2 | S | F | Adult | 40 |
| Rosa | G2 | S | F | Adult | 9 |
| Ruga | G2 | S | F | Adult | 5 |
| Sally | G2 | S | F | Subadult | 2 |
| Scapola | G2 | S | F | Adult | 7 |
| Schiarita | G2 | S | F | Adult | 29 |
| Sciura | G2 | S | F | Adult | 59 |
| Secco | G2 | S | M | Subadult | 29 |
| Severa | G2 | S | F | Adult | 37 |
| Sfasciato | G2 | S | M | Subadult | 6 |
| Sguercia | G2 | S | F | Adult | 3 |
| Sine | G2 | S | F | Adult | 31 |
| Small | G2 | S | F | Subadult | 10 |
| Sonia | G2 | S | F | Adult | 24 |
| Sorcia | G2 | S | F | Adult | 11 |
| Spiga | G2 | S | F | Subadult | 10 |
| Spilla | G2 | S | F | Adult | 33 |
| Spot | G2 | S | F | Adult | 17 |
| Stacy | G2 | S | F | Adult | 20 |
| Stella | G2 | S | F | Subadult | 8 |
| Strega | G2 | S | F | Adult | 7 |
| Susy | G2 | S | F | Adult | 13 |
| Tris | G2 | S | F | Adult | 15 |
| Rocco | G2 | AMU | M | Adult | 56 |
| Sandro | G2 | AMU | M | Adult | 18 |
| Tino | G2 | AMU | M | Subadult | 19 |
| Tinino 1 | G2 | AMU | M | Subadult | 5 |
| Tinino 2 | G2 | AMU | M | Subadult | 4 |
