# Supplementary material for: Yawn Contagion and Modality‐Matching in the Female‐Bonded Society of Geladas (Theropithecus gelada)
Source: Am J Primatol. 2024 Dec 17;87(1):e23709. doi: 10.1002/ajp.23709 (PMC11652820; doi:10.1002/ajp.23709)
Supplement: Supplementary file 3 — Supporting information. [file AJP-87-e23709-s002.docx]

YAWNING FREQUENCIES

| subject | sex | recording time (min) | total yawns | spontaneous yawns | yawns/minute |
| --- | --- | --- | --- | --- | --- |
| biondo | m | 756 | 122 | 106 | 0.14021164 |
| bratz | f | 354 | 35 | 21 | 0.059322034 |
| spilla | f | 435 | 33 | 19 | 0.043678161 |
| giangi | m | 663 | 153 | 143 | 0.215686275 |
| gianna | f | 411 | 23 | 13 | 0.03163017 |
| gessica | f | 453 | 28 | 18 | 0.039735099 |
| severa | f | 408 | 37 | 27 | 0.066176471 |
| enzo | m | 381 | 78 | 69 | 0.181102362 |
| stacy | f | 516 | 20 | 11 | 0.021317829 |
| bigne | f | 342 | 21 | 13 | 0.038011696 |
| ghiozza | f | 351 | 14 | 6 | 0.017094017 |
| roll | f | 870 | 40 | 32 | 0.036781609 |
| schiarita | f | 507 | 29 | 21 | 0.041420118 |
| braccio | f | 450 | 16 | 9 | 0.02 |
| grappolo | f | 399 | 30 | 23 | 0.05764411 |
| sumo | m | 192 | 46 | 39 | 0.203125 |
| sonia | f | 408 | 24 | 17 | 0.041666667 |
| spot | f | 486 | 17 | 10 | 0.020576132 |
| sandro | m | 123 | 18 | 11 | 0.089430894 |
| basic | f | 264 | 20 | 14 | 0.053030303 |
| blanca | f | 243 | 14 | 8 | 0.032921811 |
| blind | f | 372 | 26 | 20 | 0.053763441 |
| gatta | f | 363 | 12 | 6 | 0.016528926 |
| evasa | f | 333 | 14 | 8 | 0.024024024 |
| sciura | f | 831 | 59 | 53 | 0.06377858 |
| bernoccolo | m | 114 | 7 | 2 | 0.01754386 |
| bifida | f | 282 | 27 | 22 | 0.078014184 |
| borsa | f | 324 | 27 | 22 | 0.067901235 |
| gigetto | m | 177 | 12 | 7 | 0.039548023 |
| ghirlanda | f | 432 | 15 | 10 | 0.023148148 |
| osso | f | 429 | 18 | 13 | 0.03030303 |
| scapola | f | 528 | 7 | 2 | 0.003787879 |
| secco | m | 195 | 29 | 24 | 0.123076923 |
| sine | f | 513 | 31 | 26 | 0.050682261 |
| sorcia | f | 264 | 11 | 6 | 0.022727273 |
| tris | f | 957 | 15 | 10 | 0.010449321 |
| barbie | f | 150 | 8 | 4 | 0.026666667 |
| betta | f | 489 | 6 | 2 | 0.00408998 |
| black | m | 246 | 6 | 2 | 0.008130081 |
| bortolo | m | 156 | 9 | 5 | 0.032051282 |
| e2m6 | f | 234 | 4 | 0 | 0 |
| tino | m | 135 | 19 | 15 | 0.111111111 |
| belly | f | 123 | 6 | 3 | 0.024390244 |
| bisnonna | f | 408 | 23 | 20 | 0.049019608 |
| sguercia | f | 213 | 3 | 0 | 0 |
| spiga | f | 381 | 10 | 7 | 0.018372703 |
| stella | f | 249 | 8 | 5 | 0.020080321 |
| susy | f | 360 | 13 | 10 | 0.027777778 |
| tinino1 | m | 93 | 5 | 2 | 0.021505376 |
| bijoux | f | 114 | 9 | 7 | 0.061403509 |
| striscia | f | 348 | 5 | 3 | 0.00862069 |
| e1m6 | f | 282 | 4 | 2 | 0.007092199 |
| neomamma | f | 609 | 22 | 20 | 0.032840722 |
| rosa | f | 258 | 9 | 7 | 0.027131783 |
| ruga | f | 651 | 5 | 3 | 0.004608295 |
| strega | f | 312 | 7 | 5 | 0.016025641 |
| rocco | m | 216 | 56 | 54 | 0.25 |
| gigio | m | 117 | 1 | 0 | 0 |
| edera | f | 246 | 1 | 0 | 0 |
| strip | f | 348 | 8 | 7 | 0.020114943 |
| elly | f | 321 | 1 | 0 | 0 |
| sally | f | 339 | 2 | 1 | 0.002949853 |
| small | f | 204 | 10 | 9 | 0.044117647 |
| tinino2 | m | 81 | 4 | 3 | 0.037037037 |
| biba | f | 90 | 1 | 1 | 0.011111111 |
| gelly | f | 96 | 0 | 0 | 0 |
| sfasciato | m | 246 | 6 | 6 | 0.024390244 |

YAWN TYPES AND MORPHOLOGY

| subject | type | length | sex |
| --- | --- | --- | --- |
| Bratz | 3 | 0.05 | f |
| Schiarita | 1 | 0.12 | f |
| Gatta | 3 | 0.55 | f |
| Biondo | 3 | 0.62 | m |
| Sine | 1 | 0.66 | f |
| Giangi | 3 | 0.69 | m |
| Basic | 3 | 0.75 | f |
| Blind | 1 | 0.8 | f |
| Rocco | 1 | 0.83 | m |
| Sguercia | 1 | 0.83 | f |
| Bigne | 3 | 0.83 | f |
| Borsa | 1 | 0.84 | f |
| Secco | 2 | 0.84 | m |
| Grappolo | 1 | 0.88 | f |
| Spot | 1 | 0.88 | f |
| Bifida | 1 | 0.9 | f |
| Biondo | 1 | 0.91 | m |
| Gianna | 1 | 0.93 | f |
| Biondo | 1 | 0.94 | m |
| Giangi | 1 | 0.97 | m |
| Rocco | 3 | 0.99 | m |
| Bratz | 3 | 1 | f |
| Giangi | 3 | 1 | m |
| Bifida | 1 | 1.01 | f |
| Rocco | 1 | 1.01 | m |
| Tino | 1 | 1.01 | m |
| Sumo | 3 | 1.01 | m |
| Biondo | 1 | 1.02 | m |
| Roll | 1 | 1.02 | f |
| Schiarita | 1 | 1.02 | f |
| Tino | 1 | 1.03 | m |
| Biondo | 2 | 1.04 | m |
| Biondo | 2 | 1.05 | m |
| Sonia | 3 | 1.06 | f |
| Sonia | 1 | 1.07 | f |
| Tris | 1 | 1.07 | f |
| Enzo | 2 | 1.07 | m |
| Enzo | 3 | 1.07 | m |
| Sumo | 3 | 1.07 | m |
| Blanca | 1 | 1.08 | f |
| Giangi | 1 | 1.08 | m |
| Sciura | 1 | 1.08 | f |
| Tino | 1 | 1.08 | m |
| Enzo | 3 | 1.08 | m |
| Spot | 3 | 1.08 | f |
| Bigne | 1 | 1.09 | f |
| Biondo | 1 | 1.09 | m |
| Giangi | 3 | 1.09 | m |
| Biondo | 1 | 1.11 | m |
| Giangi | 2 | 1.11 | m |
| Giangi | 3 | 1.11 | m |
| Biondo | 1 | 1.12 | m |
| sumo | 3 | 1.12 | m |
| Blind | 1 | 1.13 | f |
| Sciura | 1 | 1.13 | f |
| spot | 1 | 1.13 | f |
| Biondo | 3 | 1.13 | m |
| Biondo | 1 | 1.14 | m |
| Grappolo | 1 | 1.14 | f |
| Sumo | 1 | 1.14 | m |
| Enzo | 3 | 1.14 | m |
| Giangi | 3 | 1.14 | m |
| Giangi | 3 | 1.14 | m |
| Sumo | 3 | 1.14 | m |
| Bratz | 1 | 1.15 | f |
| Ruga | 1 | 1.15 | f |
| Susy | 1 | 1.15 | f |
| Gianna | 1 | 1.16 | f |
| Sumo | 3 | 1.16 | m |
| Blind | 1 | 1.17 | f |
| Spilla | 1 | 1.17 | f |
| Strip | 1 | 1.17 | f |
| Sumo | 1 | 1.17 | m |
| Borsa | 2 | 1.17 | f |
| Roll | 1 | 1.18 | f |
| Sine | 1 | 1.18 | f |
| Osso | 1 | 1.19 | f |
| sorcia | 1 | 1.19 | f |
| sorcia | 1 | 1.19 | f |
| Sumo | 1 | 1.19 | m |
| Susy | 1 | 1.19 | f |
| Secco | 2 | 1.19 | m |
| Sumo | 3 | 1.19 | m |
| Braccio | 1 | 1.2 | f |
| Braccio | 1 | 1.2 | f |
| Evasa | 1 | 1.2 | f |
| Secco | 1 | 1.2 | m |
| Biondo | 3 | 1.2 | m |
| Giangi | 3 | 1.2 | m |
| Elly | 1 | 1.21 | f |
| Sciura | 1 | 1.21 | f |
| Blind | 3 | 1.21 | f |
| Enzo | 3 | 1.21 | m |
| Blind | 1 | 1.22 | f |
| Gianna | 1 | 1.22 | f |
| Osso | 1 | 1.22 | f |
| Roll | 1 | 1.22 | f |
| Enzo | 3 | 1.22 | m |
| Roll | 3 | 1.22 | f |
| Rosa | 3 | 1.22 | f |
| Blanca | 1 | 1.23 | f |
| Bratz | 1 | 1.23 | f |
| Evasa | 1 | 1.23 | f |
| Spilla | 1 | 1.23 | f |
| Tinino2 | 1 | 1.23 | m |
| Giangi | 2 | 1.23 | m |
| Biondo | 3 | 1.23 | m |
| Rocco | 3 | 1.23 | m |
| Secco | 3 | 1.23 | m |
| Gessica | 1 | 1.24 | f |
| Severa | 1 | 1.24 | f |
| Small | 1 | 1.24 | f |
| Biondo | 2 | 1.24 | m |
| Biondo | 3 | 1.24 | m |
| Enzo | 3 | 1.24 | m |
| Sumo | 3 | 1.24 | m |
| Enzo | 1 | 1.25 | m |
| Rocco | 1 | 1.25 | m |
| Ruga | 1 | 1.25 | f |
| Enzo | 2 | 1.25 | m |
| Enzo | 3 | 1.25 | m |
| Gianna | 3 | 1.25 | f |
| Bernoccolo | 1 | 1.26 | m |
| Biondo | 1 | 1.26 | m |
| Sine | 1 | 1.26 | f |
| Biondo | 2 | 1.26 | m |
| Giangi | 3 | 1.26 | m |
| Schiarita | 3 | 1.26 | f |
| Sumo | 3 | 1.26 | m |
| Neomamma | 1 | 1.27 | f |
| Roll | 1 | 1.27 | f |
| Sine | 1 | 1.27 | f |
| Bifida | 1 | 1.28 | f |
| Biondo | 1 | 1.28 | m |
| Gigio | 1 | 1.28 | m |
| Severa | 1 | 1.28 | f |
| Giangi | 2 | 1.28 | m |
| Biondo | 3 | 1.28 | m |
| Giangi | 3 | 1.28 | m |
| Sfasciato | 3 | 1.28 | m |
| Spiga | 3 | 1.28 | f |
| Bortolo | 1 | 1.29 | m |
| Gianna | 1 | 1.29 | f |
| Giangi | 3 | 1.29 | m |
| Schiarita | 3 | 1.29 | f |
| Tino | 3 | 1.29 | m |
| Bigne | 1 | 1.3 | f |
| Evasa | 1 | 1.3 | f |
| Biondo | 3 | 1.3 | m |
| Sumo | 3 | 1.3 | m |
| Enzo | 1 | 1.31 | m |
| Giangi | 1 | 1.31 | m |
| Gianna | 1 | 1.31 | f |
| Roll | 1 | 1.31 | f |
| Schiarita | 1 | 1.31 | f |
| Sciura | 1 | 1.31 | f |
| Severa | 1 | 1.31 | f |
| Tris | 1 | 1.31 | f |
| Giangi | 2 | 1.31 | m |
| Braccio | 3 | 1.31 | f |
| Enzo | 3 | 1.31 | m |
| Rocco | 3 | 1.31 | m |
| Secco | 3 | 1.31 | m |
| Blind | 1 | 1.32 | f |
| Gatta | 1 | 1.32 | f |
| Stella | 1 | 1.32 | f |
| Enzo | 2 | 1.32 | m |
| Giangi | 2 | 1.32 | m |
| Blind | 3 | 1.32 | f |
| Grappolo | 3 | 1.32 | f |
| Sciura | 3 | 1.32 | f |
| Roll | 1 | 1.33 | f |
| Rosa | 1 | 1.33 | f |
| Sine | 1 | 1.33 | f |
| Blind | 3 | 1.33 | f |
| Enzo | 3 | 1.33 | m |
| Gessica | 3 | 1.33 | f |
| Enzo | 1 | 1.34 | m |
| Gianna | 1 | 1.34 | f |
| Scapola | 1 | 1.34 | f |
| Sumo | 2 | 1.34 | m |
| Enzo | 3 | 1.34 | m |
| Grappolo | 3 | 1.34 | f |
| Blanca | 1 | 1.35 | f |
| Sciura | 1 | 1.35 | f |
| Sonia | 1 | 1.35 | f |
| Strip | 1 | 1.35 | f |
| Tris | 1 | 1.35 | f |
| Enzo | 3 | 1.35 | m |
| Osso | 1 | 1.36 | f |
| Giangi | 2 | 1.36 | m |
| Severa | 2 | 1.36 | f |
| Giangi | 1 | 1.37 | m |
| Roll | 1 | 1.37 | f |
| Sorcia | 1 | 1.37 | f |
| Strip | 1 | 1.37 | f |
| Blind | 1 | 1.38 | f |
| Grappolo | 1 | 1.38 | f |
| Roll | 1 | 1.38 | f |
| Sonia | 1 | 1.38 | f |
| Spilla | 1 | 1.38 | f |
| Tinino1 | 1 | 1.38 | m |
| Enzo | 2 | 1.38 | m |
| Basic | 3 | 1.38 | f |
| Enzo | 3 | 1.38 | m |
| Neomamma | 3 | 1.38 | f |
| Tris | 1 | 1.39 | f |
| Enzo | 1 | 1.4 | m |
| Gigetto | 1 | 1.4 | m |
| Sorcia | 1 | 1.4 | f |
| Sumo | 2 | 1.4 | m |
| Tino | 3 | 1.4 | m |
| Blanca | 1 | 1.41 | f |
| Giangi | 1 | 1.41 | m |
| Osso | 1 | 1.41 | f |
| Bigne | 3 | 1.41 | f |
| Enzo | 3 | 1.41 | m |
| Giangi | 3 | 1.41 | m |
| Sciura | 3 | 1.41 | f |
| Bigne | 1 | 1.42 | f |
| Braccio | 1 | 1.42 | f |
| Rocco | 1 | 1.42 | m |
| Sorcia | 1 | 1.42 | f |
| Spot | 1 | 1.42 | f |
| Secco | 3 | 1.42 | m |
| Susy | 1 | 1.43 | f |
| Borsa | 2 | 1.43 | f |
| Sumo | 2 | 1.43 | m |
| Enzo | 3 | 1.43 | m |
| Secco | 3 | 1.43 | m |
| Secco | 3 | 1.43 | m |
| Biba | 1 | 1.44 | f |
| Sciura | 1 | 1.44 | f |
| Severa | 1 | 1.44 | f |
| Bigne | 3 | 1.44 | f |
| Biondo | 3 | 1.44 | m |
| Giangi | 3 | 1.44 | m |
| Giangi | 3 | 1.44 | m |
| Sciura | 3 | 1.44 | f |
| Black | 1 | 1.45 | m |
| Blind | 1 | 1.45 | f |
| Biondo | 2 | 1.45 | m |
| Biondo | 2 | 1.45 | m |
| Sumo | 3 | 1.45 | m |
| Tino | 3 | 1.45 | m |
| E2M6 | 1 | 1.46 | f |
| Sciura | 1 | 1.46 | f |
| Secco | 1 | 1.46 | m |
| Severa | 1 | 1.46 | f |
| Striscia | 1 | 1.46 | f |
| Biondo | 3 | 1.46 | m |
| Enzo | 3 | 1.46 | m |
| Giangi | 3 | 1.46 | m |
| Giangi | 3 | 1.46 | m |
| Neomamma | 1 | 1.47 | f |
| Giangi | 2 | 1.47 | m |
| Giangi | 1 | 1.48 | m |
| Gigetto | 1 | 1.48 | m |
| Biondo | 2 | 1.48 | m |
| Secco | 3 | 1.48 | m |
| Rocco | 1 | 1.49 | m |
| Sine | 1 | 1.49 | f |
| Osso | 2 | 1.49 | f |
| Bigne | 3 | 1.49 | f |
| Roll | 3 | 1.49 | f |
| Spiga | 3 | 1.49 | f |
| Sumo | 3 | 1.49 | m |
| Bifida | 1 | 1.5 | f |
| Borsa | 1 | 1.5 | f |
| braccio | 1 | 1.5 | f |
| Evasa | 1 | 1.5 | f |
| Roll | 1 | 1.5 | f |
| Severa | 1 | 1.5 | f |
| Secco | 2 | 1.5 | m |
| Enzo | 3 | 1.5 | m |
| Rocco | 3 | 1.5 | m |
| Sumo | 3 | 1.5 | m |
| Basic | 1 | 1.51 | f |
| Borsa | 1 | 1.51 | f |
| Rocco | 1 | 1.51 | m |
| Sciura | 1 | 1.51 | f |
| Sonia | 1 | 1.51 | f |
| Enzo | 2 | 1.51 | m |
| Sciura | 2 | 1.51 | f |
| Biondo | 3 | 1.51 | m |
| Sine | 3 | 1.51 | f |
| Sumo | 3 | 1.51 | m |
| Biondo | 1 | 1.52 | m |
| Roll | 1 | 1.52 | f |
| Stella | 1 | 1.52 | f |
| Tino | 1 | 1.52 | m |
| Betta | 3 | 1.52 | f |
| Giangi | 3 | 1.52 | m |
| Secco | 3 | 1.52 | m |
| Osso | 1 | 1.53 | f |
| Roll | 1 | 1.53 | f |
| Scapola | 1 | 1.53 | f |
| Sciura | 1 | 1.53 | f |
| Severa | 1 | 1.53 | f |
| Severa | 1 | 1.53 | f |
| Giangi | 2 | 1.53 | m |
| Rocco | 2 | 1.53 | m |
| Sumo | 2 | 1.53 | m |
| Bernoccolo | 3 | 1.53 | m |
| Ghirlanda | 3 | 1.53 | f |
| Giangi | 3 | 1.53 | m |
| Bratz | 1 | 1.54 | f |
| Neomamma | 1 | 1.54 | f |
| Susy | 1 | 1.54 | f |
| Tinino2 | 1 | 1.54 | m |
| Enzo | 2 | 1.54 | m |
| Scapola | 2 | 1.54 | f |
| Biondo | 3 | 1.54 | m |
| Biondo | 1 | 1.55 | m |
| Bratz | 1 | 1.55 | f |
| Gianna | 1 | 1.55 | f |
| Stacy | 1 | 1.55 | f |
| Biondo | 2 | 1.55 | m |
| Biondo | 2 | 1.55 | m |
| Biondo | 3 | 1.55 | m |
| Enzo | 3 | 1.55 | m |
| Blind | 1 | 1.56 | f |
| Bortolo | 1 | 1.56 | m |
| Gessica | 1 | 1.56 | f |
| Rocco | 1 | 1.56 | m |
| Sandro | 1 | 1.56 | m |
| Sandro | 1 | 1.56 | m |
| Susy | 1 | 1.56 | f |
| Biondo | 3 | 1.56 | m |
| Giangi | 3 | 1.56 | m |
| Schiarita | 3 | 1.56 | f |
| Secco | 3 | 1.56 | m |
| Sumo | 3 | 1.56 | m |
| Tino | 3 | 1.56 | m |
| Bigne | 1 | 1.57 | f |
| Gessica | 1 | 1.57 | f |
| Sumo | 1 | 1.57 | m |
| Biondo | 2 | 1.57 | m |
| Enzo | 2 | 1.57 | m |
| Blind | 3 | 1.57 | f |
| Sumo | 3 | 1.57 | m |
| Bratz | 1 | 1.58 | f |
| Giangi | 1 | 1.58 | m |
| Giangi | 2 | 1.58 | m |
| Biondo | 3 | 1.58 | m |
| Black | 1 | 1.59 | m |
| Neomamma | 1 | 1.59 | f |
| Sonia | 1 | 1.59 | f |
| Strega | 1 | 1.59 | f |
| Susy | 1 | 1.59 | f |
| Bijoux | 3 | 1.59 | f |
| Biondo | 3 | 1.59 | m |
| Biondo | 3 | 1.59 | m |
| Borsa | 1 | 1.6 | f |
| Braccio | 1 | 1.6 | f |
| Ghirlanda | 1 | 1.6 | f |
| Neomamma | 1 | 1.6 | f |
| Rocco | 1 | 1.6 | m |
| Scapola | 1 | 1.6 | f |
| Severa | 1 | 1.6 | f |
| Enzo | 3 | 1.6 | m |
| Giangi | 3 | 1.6 | m |
| Giangi | 3 | 1.6 | m |
| Sandro | 3 | 1.6 | m |
| Sumo | 3 | 1.6 | m |
| Gianna | 1 | 1.61 | f |
| Osso | 1 | 1.61 | f |
| Giangi | 2 | 1.61 | m |
| Belly | 3 | 1.61 | f |
| Biondo | 3 | 1.61 | m |
| Biondo | 3 | 1.61 | m |
| Tino | 3 | 1.61 | m |
| Basic | 1 | 1.62 | f |
| Blind | 1 | 1.62 | f |
| Blind | 1 | 1.62 | f |
| Bortolo | 1 | 1.62 | m |
| Braccio | 1 | 1.62 | f |
| Braccio | 1 | 1.62 | f |
| Giangi | 1 | 1.62 | m |
| Giangi | 1 | 1.62 | m |
| Gianna | 1 | 1.62 | f |
| Neomamma | 1 | 1.62 | f |
| Biondo | 3 | 1.62 | m |
| Enzo | 3 | 1.62 | m |
| Tino | 3 | 1.62 | m |
| Bratz | 1 | 1.63 | f |
| Rocco | 1 | 1.63 | m |
| Sonia | 1 | 1.63 | f |
| Giangi | 2 | 1.63 | m |
| Biondo | 3 | 1.63 | m |
| Gessica | 3 | 1.63 | f |
| Osso | 3 | 1.63 | f |
| Sandro | 3 | 1.63 | m |
| Bijoux | 1 | 1.64 | f |
| Bratz | 1 | 1.64 | f |
| Enzo | 1 | 1.64 | m |
| Roll | 1 | 1.64 | f |
| Spilla | 1 | 1.64 | f |
| Stacy | 1 | 1.64 | f |
| Stacy | 1 | 1.64 | f |
| Enzo | 3 | 1.64 | m |
| Giangi | 3 | 1.64 | m |
| Sciura | 3 | 1.64 | f |
| Basic | 1 | 1.65 | f |
| Enzo | 1 | 1.65 | m |
| Severa | 1 | 1.65 | f |
| Severa | 1 | 1.65 | f |
| Sine | 1 | 1.65 | f |
| Biondo | 2 | 1.65 | m |
| Giangi | 2 | 1.65 | m |
| Sciura | 3 | 1.65 | f |
| Biondo | 1 | 1.66 | m |
| Sorcia | 1 | 1.66 | f |
| Stacy | 1 | 1.66 | f |
| Enzo | 2 | 1.66 | m |
| Rocco | 2 | 1.66 | m |
| Biondo | 3 | 1.66 | m |
| Osso | 3 | 1.66 | f |
| Braccio | 1 | 1.67 | f |
| Evasa | 1 | 1.67 | f |
| Sine | 1 | 1.67 | f |
| Spot | 1 | 1.67 | f |
| Strega | 1 | 1.67 | f |
| Bifida | 3 | 1.67 | f |
| Bifida | 3 | 1.67 | f |
| Braccio | 3 | 1.67 | f |
| enzo | 3 | 1.67 | m |
| Giangi | 3 | 1.67 | m |
| Giangi | 3 | 1.67 | m |
| Strega | 3 | 1.67 | f |
| Sumo | 3 | 1.67 | m |
| Belly | 1 | 1.68 | f |
| Borsa | 1 | 1.68 | f |
| Braccio | 1 | 1.68 | f |
| Roll | 1 | 1.68 | f |
| Severa | 1 | 1.68 | f |
| Enzo | 3 | 1.68 | m |
| Giangi | 3 | 1.68 | m |
| Giangi | 3 | 1.68 | m |
| Tino | 3 | 1.68 | m |
| Blind | 1 | 1.69 | f |
| Evasa | 1 | 1.69 | f |
| Grappolo | 1 | 1.69 | f |
| Tinino1 | 1 | 1.69 | m |
| Blanca | 3 | 1.69 | f |
| Bigne | 1 | 1.7 | f |
| Sine | 1 | 1.7 | f |
| Sine | 1 | 1.7 | f |
| Strip | 1 | 1.7 | f |
| Enzo | 3 | 1.7 | m |
| Gessica | 3 | 1.7 | f |
| Bifida | 1 | 1.71 | f |
| Bisnonna | 1 | 1.71 | f |
| Gessica | 1 | 1.71 | f |
| Giangi | 1 | 1.71 | m |
| Secco | 1 | 1.71 | m |
| Sine | 1 | 1.71 | f |
| sorcia | 1 | 1.71 | f |
| Spilla | 1 | 1.71 | f |
| Stella | 1 | 1.71 | f |
| Borsa | 2 | 1.71 | f |
| Tris | 3 | 1.71 | f |
| Braccio | 1 | 1.72 | f |
| Neomamma | 1 | 1.72 | f |
| Rocco | 1 | 1.72 | m |
| Rosa | 1 | 1.72 | f |
| Schiarita | 1 | 1.72 | f |
| Spilla | 1 | 1.72 | f |
| Severa | 2 | 1.72 | f |
| Biondo | 3 | 1.72 | m |
| Biondo | 3 | 1.72 | m |
| Bratz | 3 | 1.72 | f |
| Enzo | 3 | 1.72 | m |
| Giangi | 3 | 1.72 | m |
| Neomamma | 1 | 1.73 | f |
| Rocco | 1 | 1.73 | m |
| Roll | 1 | 1.73 | f |
| Stacy | 1 | 1.73 | f |
| Biondo | 3 | 1.73 | m |
| Bratz | 3 | 1.73 | f |
| Giangi | 3 | 1.73 | m |
| Sumo | 3 | 1.73 | m |
| Barbie | 1 | 1.74 | f |
| Barbie | 1 | 1.74 | f |
| Basic | 1 | 1.74 | f |
| Ghiozza | 1 | 1.74 | f |
| Ghirlanda | 1 | 1.74 | f |
| Sine | 1 | 1.74 | f |
| Stella | 1 | 1.74 | f |
| Strega | 1 | 1.74 | f |
| Rocco | 2 | 1.74 | m |
| Giangi | 3 | 1.74 | m |
| Sumo | 3 | 1.74 | m |
| Roll | 1 | 1.75 | f |
| Braccio | 2 | 1.75 | f |
| Giangi | 2 | 1.75 | m |
| Basic | 1 | 1.76 | f |
| Gessica | 1 | 1.76 | f |
| Biondo | 3 | 1.76 | m |
| Bratz | 3 | 1.76 | f |
| Gigetto | 3 | 1.76 | m |
| Rocco | 3 | 1.76 | m |
| Sumo | 3 | 1.76 | m |
| Bisnonna | 1 | 1.77 | f |
| Black | 1 | 1.77 | m |
| Gessica | 1 | 1.77 | f |
| Gigetto | 1 | 1.77 | m |
| Roll | 1 | 1.77 | f |
| Sandro | 1 | 1.77 | m |
| Spot | 1 | 1.77 | f |
| Strega | 1 | 1.77 | f |
| Biondo | 3 | 1.77 | m |
| Grappolo | 3 | 1.77 | f |
| Tino | 3 | 1.77 | m |
| Bifida | 1 | 1.78 | f |
| Ghiozza | 1 | 1.78 | f |
| neomamma | 1 | 1.78 | f |
| Sciura | 1 | 1.78 | f |
| Blind | 3 | 1.78 | f |
| Blind | 3 | 1.78 | f |
| Spilla | 3 | 1.78 | f |
| Sumo | 3 | 1.78 | m |
| Bernoccolo | 1 | 1.79 | m |
| Bigne | 1 | 1.79 | f |
| Biondo | 1 | 1.79 | m |
| Blanca | 1 | 1.79 | f |
| Sine | 1 | 1.79 | f |
| Biondo | 3 | 1.79 | m |
| Sumo | 3 | 1.79 | m |
| Biondo | 1 | 1.8 | m |
| Bratz | 1 | 1.8 | f |
| Tris | 1 | 1.8 | f |
| Enzo | 2 | 1.8 | m |
| Tino | 2 | 1.8 | m |
| Enzo | 3 | 1.8 | m |
| Rocco | 3 | 1.8 | m |
| Edera | 1 | 1.81 | f |
| Gigetto | 1 | 1.81 | m |
| Biondo | 2 | 1.81 | m |
| Enzo | 2 | 1.81 | m |
| Evasa | 2 | 1.81 | f |
| Belly | 3 | 1.81 | f |
| Giangi | 3 | 1.81 | m |
| Sandro | 3 | 1.81 | m |
| E2m6 | 1 | 1.82 | f |
| Enzo | 1 | 1.82 | m |
| Sciura | 1 | 1.82 | f |
| Bratz | 3 | 1.82 | f |
| Giangi | 3 | 1.82 | m |
| Rocco | 3 | 1.82 | m |
| Rocco | 3 | 1.82 | m |
| Giangi | 1 | 1.83 | m |
| Roll | 1 | 1.83 | f |
| Schiarita | 1 | 1.83 | f |
| Sine | 1 | 1.83 | f |
| Stacy | 1 | 1.83 | f |
| Sciura | 2 | 1.83 | f |
| Biondo | 3 | 1.83 | m |
| Belly | 1 | 1.84 | f |
| Bisnonna | 1 | 1.84 | f |
| Severa | 1 | 1.84 | f |
| Sine | 1 | 1.84 | f |
| Stacy | 1 | 1.84 | f |
| Stella | 1 | 1.84 | f |
| Striscia | 1 | 1.84 | f |
| Enzo | 2 | 1.84 | m |
| Giangi | 2 | 1.84 | m |
| Giangi | 3 | 1.84 | m |
| Gessica | 1 | 1.85 | f |
| Schiarita | 1 | 1.85 | f |
| Severa | 1 | 1.85 | f |
| Sonia | 1 | 1.85 | f |
| Biondo | 2 | 1.85 | m |
| Gessica | 3 | 1.85 | f |
| Grappolo | 3 | 1.85 | f |
| Rocco | 3 | 1.85 | m |
| Basic | 1 | 1.86 | f |
| Basic | 1 | 1.86 | f |
| Bisnonna | 1 | 1.86 | f |
| Black | 1 | 1.86 | m |
| Rocco | 1 | 1.86 | m |
| Roll | 1 | 1.86 | f |
| Susy | 1 | 1.86 | f |
| Biondo | 3 | 1.86 | m |
| Osso | 1 | 1.87 | f |
| Sally | 1 | 1.87 | f |
| Severa | 1 | 1.87 | f |
| Biondo | 3 | 1.87 | m |
| Secco | 3 | 1.87 | m |
| Secco | 3 | 1.87 | m |
| Sumo | 3 | 1.87 | m |
| sumo | 3 | 1.87 | m |
| Blind | 1 | 1.88 | f |
| Biondo | 1 | 1.89 | m |
| Borsa | 1 | 1.89 | f |
| Evasa | 1 | 1.89 | f |
| Sonia | 1 | 1.89 | f |
| Spot | 1 | 1.89 | f |
| Striscia | 1 | 1.89 | f |
| Secco | 2 | 1.89 | m |
| Biondo | 3 | 1.89 | m |
| Giangi | 3 | 1.89 | m |
| Grappolo | 3 | 1.89 | f |
| Sciura | 1 | 1.9 | f |
| Sonia | 1 | 1.9 | f |
| Giangi | 2 | 1.9 | m |
| Biondo | 3 | 1.9 | m |
| Enzo | 3 | 1.9 | m |
| Rosa | 3 | 1.9 | f |
| Sumo | 3 | 1.9 | m |
| Blanca | 1 | 1.91 | f |
| Bratz | 1 | 1.91 | f |
| Gessica | 1 | 1.91 | f |
| Severa | 1 | 1.91 | f |
| Tris | 1 | 1.91 | f |
| Giangi | 2 | 1.91 | m |
| Neomamma | 2 | 1.91 | f |
| Blind | 3 | 1.91 | f |
| Rocco | 3 | 1.91 | m |
| Sfasciato | 3 | 1.91 | m |
| Grappolo | 1 | 1.92 | f |
| Sciura | 1 | 1.92 | f |
| Enzo | 2 | 1.92 | m |
| Enzo | 3 | 1.92 | m |
| Rocco | 3 | 1.92 | m |
| Striscia | 3 | 1.92 | f |
| sciura | 1 | 1.93 | f |
| Stacy | 1 | 1.93 | f |
| Biondo | 3 | 1.93 | m |
| Gianna | 3 | 1.93 | f |
| Schiarita | 3 | 1.93 | f |
| Sine | 3 | 1.93 | f |
| Stacy | 3 | 1.93 | f |
| Bratz | 1 | 1.94 | f |
| Gessica | 1 | 1.94 | f |
| Neomamma | 1 | 1.94 | f |
| Schiarita | 1 | 1.94 | f |
| Sciura | 1 | 1.94 | f |
| Sonia | 1 | 1.94 | f |
| Biondo | 2 | 1.94 | m |
| Sciura | 2 | 1.94 | f |
| Bigne | 3 | 1.94 | f |
| Biondo | 3 | 1.94 | m |
| Enzo | 3 | 1.94 | m |
| Giangi | 3 | 1.94 | m |
| Blind | 1 | 1.95 | f |
| Secco | 1 | 1.95 | m |
| Spiga | 1 | 1.95 | f |
| Bigne | 3 | 1.95 | f |
| Rocco | 3 | 1.95 | m |
| Sandro | 3 | 1.95 | m |
| Blanca | 1 | 1.96 | f |
| E1M6 | 1 | 1.96 | f |
| Neomamma | 1 | 1.96 | f |
| Roll | 1 | 1.96 | f |
| Sine | 1 | 1.96 | f |
| Tinino2 | 1 | 1.96 | m |
| Bortolo | 2 | 1.96 | m |
| Giangi | 2 | 1.96 | m |
| Gatta | 1 | 1.97 | f |
| Giangi | 1 | 1.97 | m |
| Severa | 1 | 1.97 | f |
| Sonia | 1 | 1.97 | f |
| Sonia | 1 | 1.97 | f |
| Sorcia | 1 | 1.97 | f |
| Secco | 3 | 1.97 | m |
| Sumo | 3 | 1.97 | m |
| Evasa | 1 | 1.98 | f |
| Biondo | 2 | 1.98 | m |
| Bifida | 3 | 1.98 | f |
| Biondo | 3 | 1.98 | m |
| Bratz | 3 | 1.98 | f |
| Enzo | 3 | 1.98 | m |
| Giangi | 1 | 1.99 | m |
| Schiarita | 1 | 1.99 | f |
| Sonia | 1 | 1.99 | f |
| Bijoux | 2 | 1.99 | f |
| Biondo | 3 | 1.99 | m |
| Bisnonna | 3 | 1.99 | f |
| Bigne | 1 | 2 | f |
| Small | 1 | 2 | f |
| Spot | 1 | 2 | f |
| sumo | 1 | 2 | m |
| Tino | 1 | 2 | m |
| Biondo | 3 | 2 | m |
| Blind | 3 | 2 | f |
| Ghirlanda | 3 | 2 | f |
| Giangi | 3 | 2 | m |
| Gessica | 1 | 2.01 | f |
| Sally | 1 | 2.01 | f |
| Severa | 1 | 2.01 | f |
| Bigne | 3 | 2.01 | f |
| Ghirlanda | 3 | 2.01 | f |
| Gessica | 1 | 2.02 | f |
| Gigetto | 1 | 2.02 | m |
| Roll | 1 | 2.02 | f |
| Biondo | 3 | 2.02 | m |
| Bratz | 3 | 2.02 | f |
| Bortolo | 1 | 2.03 | m |
| Neomamma | 1 | 2.03 | f |
| Sine | 1 | 2.03 | f |
| Biondo | 1 | 2.04 | m |
| E2M6 | 1 | 2.04 | f |
| Ghirlanda | 1 | 2.04 | f |
| Enzo | 2 | 2.04 | m |
| Grappolo | 3 | 2.04 | f |
| Rocco | 3 | 2.04 | m |
| Gessica | 1 | 2.05 | f |
| Gianna | 1 | 2.05 | f |
| Roll | 1 | 2.05 | f |
| Giangi | 2 | 2.05 | m |
| Blind | 1 | 2.06 | f |
| Gessica | 1 | 2.06 | f |
| Spilla | 1 | 2.06 | f |
| Giangi | 2 | 2.06 | m |
| Stella | 2 | 2.06 | f |
| Blanca | 1 | 2.07 | f |
| Blanca | 1 | 2.07 | f |
| Tinino1 | 1 | 2.07 | m |
| Biondo | 3 | 2.07 | m |
| Giangi | 3 | 2.07 | m |
| Secco | 3 | 2.07 | m |
| Borsa | 1 | 2.08 | f |
| Evasa | 1 | 2.08 | f |
| Schiarita | 1 | 2.08 | f |
| Sciura | 1 | 2.08 | f |
| Bratz | 3 | 2.08 | f |
| Grappolo | 3 | 2.08 | f |
| Bigne | 1 | 2.09 | f |
| Ghirlanda | 1 | 2.09 | f |
| Black | 1 | 2.1 | m |
| Sine | 1 | 2.1 | f |
| Sorcia | 1 | 2.1 | f |
| Enzo | 3 | 2.1 | m |
| Rocco | 3 | 2.1 | m |
| Tino | 3 | 2.1 | m |
| Sandro | 1 | 2.11 | m |
| Sonia | 1 | 2.11 | f |
| Stacy | 1 | 2.11 | f |
| Biondo | 2 | 2.11 | m |
| Gigetto | 1 | 2.12 | m |
| Sciura | 1 | 2.12 | f |
| Enzo | 3 | 2.12 | m |
| Ghirlanda | 3 | 2.12 | f |
| Betta | 1 | 2.13 | f |
| Biondo | 1 | 2.13 | m |
| Sonia | 1 | 2.13 | f |
| Sorcia | 1 | 2.13 | f |
| Braccio | 2 | 2.13 | f |
| Blind | 1 | 2.14 | f |
| Bortolo | 1 | 2.14 | m |
| Schiarita | 1 | 2.14 | f |
| Sine | 2 | 2.14 | f |
| Enzo | 3 | 2.14 | m |
| Basic | 1 | 2.15 | f |
| Sine | 1 | 2.15 | f |
| Tris | 1 | 2.15 | f |
| Bernoccolo | 3 | 2.15 | m |
| Rocco | 3 | 2.15 | m |
| Sine | 1 | 2.16 | f |
| Enzo | 3 | 2.16 | m |
| Rocco | 3 | 2.16 | m |
| Gianna | 1 | 2.17 | f |
| Neomamma | 1 | 2.17 | f |
| Small | 1 | 2.17 | f |
| Spilla | 1 | 2.17 | f |
| Bifida | 3 | 2.17 | f |
| Giangi | 3 | 2.17 | m |
| Sciura | 3 | 2.17 | f |
| Gatta | 1 | 2.18 | f |
| Gessica | 1 | 2.18 | f |
| Enzo | 3 | 2.18 | m |
| Ghirlanda | 3 | 2.18 | f |
| Neomamma | 1 | 2.19 | f |
| Rocco | 1 | 2.19 | m |
| Severa | 1 | 2.19 | f |
| Strega | 1 | 2.19 | f |
| Bernoccolo | 3 | 2.19 | m |
| Biondo | 3 | 2.19 | m |
| Rocco | 3 | 2.19 | m |
| Secco | 3 | 2.19 | m |
| Giangi | 1 | 2.2 | m |
| Spot | 1 | 2.2 | f |
| Tinino2 | 1 | 2.2 | m |
| Giangi | 2 | 2.2 | m |
| Bifida | 3 | 2.2 | f |
| Biondo | 3 | 2.2 | m |
| Biondo | 1 | 2.21 | m |
| Rocco | 3 | 2.21 | m |
| Bisnonna | 1 | 2.22 | f |
| Gianna | 1 | 2.22 | f |
| Roll | 1 | 2.22 | f |
| Sine | 1 | 2.22 | f |
| Stella | 1 | 2.22 | f |
| Ghiozza | 3 | 2.22 | f |
| Bratz | 1 | 2.23 | f |
| Biondo | 3 | 2.23 | m |
| Bratz | 3 | 2.23 | f |
| Borsa | 1 | 2.24 | f |
| E1M6 | 1 | 2.24 | f |
| Roll | 1 | 2.24 | f |
| Biondo | 3 | 2.24 | m |
| Rocco | 3 | 2.24 | m |
| Schiarita | 3 | 2.24 | f |
| Sfasciato | 3 | 2.24 | m |
| Sine | 3 | 2.24 | f |
| Sine | 3 | 2.24 | f |
| Bigne | 1 | 2.25 | f |
| Bratz | 1 | 2.25 | f |
| E2m6 | 1 | 2.25 | f |
| Ruga | 1 | 2.25 | f |
| Enzo | 3 | 2.25 | m |
| Spilla | 1 | 2.26 | f |
| Rocco | 3 | 2.26 | m |
| Borsa | 2 | 2.27 | f |
| Enzo | 3 | 2.27 | m |
| Ghirlanda | 3 | 2.27 | f |
| Basic | 1 | 2.28 | f |
| Gianna | 1 | 2.28 | f |
| Sine | 1 | 2.28 | f |
| Spiga | 1 | 2.28 | f |
| Biondo | 2 | 2.28 | m |
| Bifida | 3 | 2.28 | f |
| Biondo | 3 | 2.28 | m |
| Barbie | 1 | 2.29 | f |
| Bortolo | 1 | 2.29 | m |
| Bratz | 1 | 2.29 | f |
| Gigetto | 1 | 2.29 | m |
| Small | 1 | 2.29 | f |
| Small | 1 | 2.29 | f |
| Tris | 1 | 2.29 | f |
| Tris | 1 | 2.29 | f |
| Giangi | 2 | 2.29 | m |
| Sciura | 1 | 2.3 | f |
| Stacy | 1 | 2.3 | f |
| Bifida | 2 | 2.3 | f |
| Giangi | 2 | 2.3 | m |
| Barbie | 3 | 2.3 | f |
| Barbie | 3 | 2.3 | f |
| Enzo | 3 | 2.3 | m |
| Sandro | 3 | 2.3 | m |
| Severa | 1 | 2.31 | f |
| Bisnonna | 3 | 2.31 | f |
| Ghiozza | 1 | 2.32 | f |
| Enzo | 3 | 2.32 | m |
| Scapola | 3 | 2.32 | f |
| Ghirlanda | 1 | 2.33 | f |
| Rocco | 1 | 2.33 | m |
| Sciura | 1 | 2.33 | f |
| Braccio | 3 | 2.33 | f |
| Enzo | 3 | 2.33 | m |
| Sandro | 3 | 2.33 | m |
| Belly | 1 | 2.34 | f |
| Spiga | 1 | 2.34 | f |
| Stacy | 1 | 2.35 | f |
| Enzo | 3 | 2.35 | m |
| Roll | 1 | 2.36 | f |
| Bisnonna | 3 | 2.36 | f |
| Enzo | 3 | 2.36 | m |
| Gianna | 3 | 2.36 | f |
| Stacy | 1 | 2.37 | f |
| Rocco | 3 | 2.37 | m |
| Ghiozza | 1 | 2.38 | f |
| Ghiozza | 1 | 2.38 | f |
| Striscia | 1 | 2.38 | f |
| Bifida | 3 | 2.38 | f |
| Gessica | 3 | 2.38 | f |
| Giangi | 3 | 2.38 | m |
| Grappolo | 3 | 2.38 | f |
| Blind | 1 | 2.39 | f |
| Neomamma | 1 | 2.39 | f |
| Spilla | 1 | 2.39 | f |
| Tris | 1 | 2.39 | f |
| Biondo | 3 | 2.39 | m |
| Grappolo | 3 | 2.39 | f |
| Blanca | 1 | 2.4 | f |
| Biondo | 3 | 2.4 | m |
| Susy | 3 | 2.4 | f |
| Bisnonna | 1 | 2.41 | f |
| Sciura | 1 | 2.41 | f |
| Spilla | 1 | 2.41 | f |
| Basic | 3 | 2.41 | f |
| Gianna | 3 | 2.41 | f |
| Spilla | 3 | 2.41 | f |
| Bisnonna | 1 | 2.42 | f |
| Spiga | 1 | 2.42 | f |
| Bijoux | 3 | 2.42 | f |
| Ghirlanda | 1 | 2.43 | f |
| Severa | 1 | 2.43 | f |
| Rocco | 3 | 2.43 | m |
| Secco | 3 | 2.43 | m |
| Biondo | 1 | 2.44 | m |
| Sguercia | 1 | 2.44 | f |
| Enzo | 3 | 2.44 | m |
| Rosa | 3 | 2.44 | f |
| Sandro | 3 | 2.44 | m |
| Betta | 1 | 2.45 | f |
| Blanca | 1 | 2.45 | f |
| Spot | 1 | 2.45 | f |
| Bijoux | 3 | 2.45 | f |
| Rocco | 3 | 2.45 | m |
| Betta | 1 | 2.46 | f |
| Biondo | 3 | 2.46 | m |
| Sumo | 3 | 2.46 | m |
| Ghiozza | 1 | 2.47 | f |
| Small | 1 | 2.47 | f |
| Stacy | 1 | 2.47 | f |
| Severa | 2 | 2.47 | f |
| Sfasciato | 3 | 2.47 | m |
| Sonia | 3 | 2.47 | f |
| Biondo | 1 | 2.48 | m |
| Grappolo | 1 | 2.48 | f |
| Sciura | 1 | 2.48 | f |
| Bisnonna | 3 | 2.48 | f |
| Belly | 1 | 2.49 | f |
| E1M6 | 1 | 2.49 | f |
| Neomamma | 1 | 2.49 | f |
| Sciura | 1 | 2.49 | f |
| Susy | 1 | 2.49 | f |
| Osso | 3 | 2.49 | f |
| sumo | 3 | 2.49 | m |
| Ghiozza | 1 | 2.5 | f |
| Osso | 1 | 2.5 | f |
| Rocco | 3 | 2.5 | m |
| Bijoux | 1 | 2.51 | f |
| Biondo | 1 | 2.51 | m |
| Sumo | 1 | 2.51 | m |
| Borsa | 2 | 2.51 | f |
| Sonia | 2 | 2.51 | f |
| Bernoccolo | 1 | 2.52 | m |
| Spilla | 1 | 2.52 | f |
| Bisnonna | 3 | 2.52 | f |
| Giangi | 3 | 2.52 | m |
| Bortolo | 1 | 2.53 | m |
| Sciura | 1 | 2.53 | f |
| Rosa | 3 | 2.53 | f |
| Secco | 3 | 2.53 | m |
| Spilla | 3 | 2.53 | f |
| Bifida | 3 | 2.54 | f |
| Bratz | 3 | 2.54 | f |
| Evasa | 1 | 2.55 | f |
| Gessica | 1 | 2.55 | f |
| Blind | 3 | 2.55 | f |
| Giangi | 3 | 2.55 | m |
| Roll | 1 | 2.56 | f |
| Stella | 1 | 2.56 | f |
| Tris | 1 | 2.56 | f |
| Enzo | 3 | 2.56 | m |
| Secco | 3 | 2.56 | m |
| Schiarita | 3 | 2.57 | f |
| Tris | 3 | 2.57 | f |
| Neomamma | 1 | 2.59 | f |
| Sfasciato | 2 | 2.59 | m |
| Bratz | 3 | 2.59 | f |
| Borsa | 1 | 2.6 | f |
| Giangi | 2 | 2.6 | m |
| Bifida | 1 | 2.61 | f |
| Spot | 2 | 2.61 | f |
| Gatta | 1 | 2.62 | f |
| Spot | 1 | 2.62 | f |
| Biondo | 3 | 2.62 | m |
| Biondo | 1 | 2.63 | m |
| Bisnonna | 1 | 2.63 | f |
| Bifida | 3 | 2.63 | f |
| Gatta | 1 | 2.64 | f |
| Spot | 1 | 2.64 | f |
| Gessica | 1 | 2.65 | f |
| Sandro | 1 | 2.65 | m |
| Sine | 1 | 2.65 | f |
| Enzo | 3 | 2.65 | m |
| Evasa | 3 | 2.65 | f |
| Spilla | 3 | 2.65 | f |
| Sciura | 1 | 2.66 | f |
| Rocco | 3 | 2.66 | m |
| Sciura | 1 | 2.67 | f |
| Giangi | 3 | 2.67 | m |
| Schiarita | 1 | 2.68 | f |
| Schiarita | 1 | 2.68 | f |
| Sine | 1 | 2.68 | f |
| Grappolo | 3 | 2.68 | f |
| Gatta | 1 | 2.69 | f |
| Bisnonna | 1 | 2.7 | f |
| Grappolo | 1 | 2.7 | f |
| Sonia | 1 | 2.7 | f |
| Giangi | 2 | 2.7 | m |
| Sciura | 1 | 2.71 | f |
| Enzo | 3 | 2.71 | m |
| Enzo | 3 | 2.71 | m |
| Grappolo | 3 | 2.71 | f |
| Strip | 3 | 2.71 | f |
| Ghirlanda | 1 | 2.72 | f |
| Susy | 1 | 2.72 | f |
| Sciura | 3 | 2.72 | f |
| Enzo | 3 | 2.74 | m |
| Bratz | 1 | 2.75 | f |
| Sciura | 1 | 2.75 | f |
| Grappolo | 3 | 2.75 | f |
| Basic | 1 | 2.76 | f |
| Gessica | 1 | 2.76 | f |
| Sciura | 1 | 2.76 | f |
| Bifida | 3 | 2.76 | f |
| Borsa | 1 | 2.77 | f |
| Rocco | 3 | 2.78 | m |
| Spilla | 3 | 2.78 | f |
| tino | 3 | 2.78 | m |
| Gessica | 3 | 2.79 | f |
| Schiarita | 3 | 2.79 | f |
| Bratz | 1 | 2.8 | f |
| Enzo | 3 | 2.8 | m |
| Rocco | 3 | 2.8 | m |
| Bifida | 3 | 2.81 | f |
| Stacy | 3 | 2.81 | f |
| Borsa | 1 | 2.82 | f |
| Strip | 1 | 2.82 | f |
| Sine | 3 | 2.82 | f |
| Spilla | 3 | 2.82 | f |
| Gatta | 3 | 2.83 | f |
| Spot | 1 | 2.84 | f |
| Biondo | 2 | 2.85 | m |
| Enzo | 3 | 2.86 | m |
| Scapola | 1 | 2.87 | f |
| Gatta | 3 | 2.87 | f |
| Gianna | 3 | 2.87 | f |
| Sine | 3 | 2.88 | f |
| Gessica | 3 | 2.89 | f |
| Severa | 1 | 2.9 | f |
| Susy | 1 | 2.9 | f |
| Ghiozza | 3 | 2.91 | f |
| Osso | 3 | 2.91 | f |
| Bijoux | 1 | 2.92 | f |
| Bisnonna | 3 | 2.92 | f |
| Bratz | 1 | 2.93 | f |
| Roll | 1 | 2.93 | f |
| Biondo | 3 | 2.93 | m |
| Borsa | 1 | 2.96 | f |
| Giangi | 3 | 2.96 | m |
| Borsa | 1 | 2.97 | f |
| Basic | 3 | 2.97 | f |
| Black | 1 | 2.98 | m |
| Sandro | 3 | 2.98 | m |
| Schiarita | 3 | 3 | f |
| Borsa | 1 | 3.01 | f |
| Sciura | 1 | 3.01 | f |
| Gianna | 1 | 3.02 | f |
| Schiarita | 1 | 3.02 | f |
| Sciura | 2 | 3.02 | f |
| Secco | 3 | 3.02 | m |
| Biondo | 3 | 3.04 | m |
| Enzo | 3 | 3.04 | m |
| Ghirlanda | 1 | 3.05 | f |
| Biondo | 3 | 3.05 | m |
| Bisnonna | 1 | 3.06 | f |
| Giangi | 1 | 3.06 | m |
| Severa | 1 | 3.06 | f |
| Bisnonna | 1 | 3.07 | f |
| Spilla | 1 | 3.07 | f |
| Bigne | 3 | 3.07 | f |
| Sonia | 3 | 3.07 | f |
| Sonia | 3 | 3.07 | f |
| Borsa | 3 | 3.08 | f |
| Stacy | 1 | 3.09 | f |
| Osso | 3 | 3.09 | f |
| Small | 1 | 3.1 | f |
| Sandro | 2 | 3.13 | m |
| Gigetto | 1 | 3.14 | m |
| Osso | 1 | 3.14 | f |
| Spiga | 1 | 3.14 | f |
| Gessica | 1 | 3.16 | f |
| E1M6 | 1 | 3.17 | f |
| Rosa | 1 | 3.18 | f |
| Stacy | 3 | 3.18 | f |
| Grappolo | 3 | 3.19 | f |
| Giangi | 3 | 3.2 | m |
| Giangi | 3 | 3.2 | m |
| Bratz | 1 | 3.21 | f |
| Sonia | 1 | 3.23 | f |
| Bisnonna | 3 | 3.23 | f |
| Giangi | 3 | 3.23 | m |
| Roll | 3 | 3.24 | f |
| Grappolo | 3 | 3.25 | f |
| Bijoux | 3 | 3.27 | f |
| Giangi | 3 | 3.27 | m |
| Bratz | 1 | 3.29 | f |
| Borsa | 2 | 3.29 | f |
| Bifida | 1 | 3.3 | f |
| Borsa | 3 | 3.3 | f |
| Grappolo | 3 | 3.3 | f |
| Biondo | 3 | 3.31 | m |
| Bratz | 3 | 3.31 | f |
| Borsa | 3 | 3.32 | f |
| Giangi | 3 | 3.32 | m |
| Neomamma | 3 | 3.32 | f |
| Grappolo | 3 | 3.33 | f |
| Bernoccolo | 1 | 3.34 | m |
| Bratz | 3 | 3.34 | f |
| Ghirlanda | 1 | 3.36 | f |
| Sandro | 3 | 3.36 | m |
| Severa | 2 | 3.37 | f |
| Sonia | 1 | 3.38 | f |
| Borsa | 1 | 3.4 | f |
| Gigetto | 1 | 3.4 | m |
| Grappolo | 3 | 3.4 | f |
| Sciura | 1 | 3.41 | f |
| Giangi | 3 | 3.42 | m |
| Spiga | 3 | 3.43 | f |
| Roll | 1 | 3.44 | f |
| Small | 1 | 3.45 | f |
| Strega | 1 | 3.46 | f |
| Evasa | 1 | 3.49 | f |
| Barbie | 3 | 3.49 | f |
| Rocco | 3 | 3.49 | m |
| Borsa | 1 | 3.52 | f |
| Spilla | 1 | 3.59 | f |
| Sciura | 2 | 3.59 | f |
| Giangi | 2 | 3.61 | m |
| Evasa | 1 | 3.62 | f |
| Giangi | 3 | 3.65 | m |
| Grappolo | 3 | 3.65 | f |
| Sguercia | 3 | 3.67 | f |
| Schiarita | 1 | 3.68 | f |
| Schiarita | 3 | 3.72 | f |
| Spilla | 1 | 3.73 | f |
| Tinino1 | 1 | 3.73 | m |
| Secco | 3 | 3.74 | m |
| Spot | 1 | 3.75 | f |
| Grappolo | 2 | 3.76 | f |
| Grappolo | 3 | 3.76 | f |
| Rocco | 3 | 3.76 | m |
| Stacy | 3 | 3.76 | f |
| Spilla | 1 | 3.78 | f |
| Roll | 1 | 3.8 | f |
| Roll | 3 | 3.8 | f |
| Roll | 1 | 3.83 | f |
| Grappolo | 3 | 3.86 | f |
| Neomamma | 1 | 3.89 | f |
| Gatta | 3 | 3.89 | f |
| Gessica | 3 | 3.94 | f |
| Sciura | 3 | 3.97 | f |
| Ruga | 3 | 4 | f |
| Spilla | 3 | 4 | f |
| Betta | 1 | 4.02 | f |
| Schiarita | 1 | 4.03 | f |
| Tino | 3 | 4.03 | m |
| Sciura | 3 | 4.04 | f |
| Gianna | 1 | 4.07 | f |
| Sciura | 3 | 4.08 | f |
| Borsa | 3 | 4.12 | f |
| Bratz | 3 | 4.18 | f |
| Giangi | 3 | 4.22 | m |
| Severa | 1 | 4.25 | f |
| Severa | 1 | 4.25 | f |
| Susy | 3 | 4.25 | f |
| Ghiozza | 3 | 4.26 | f |
| Strip | 3 | 4.29 | f |
| Ruga | 1 | 4.3 | f |
| Bigne | 1 | 4.31 | f |
| Spiga | 3 | 4.31 | f |
| Roll | 1 | 4.32 | f |
| Rocco | 3 | 4.35 | m |
| Grappolo | 1 | 4.37 | f |
| Rocco | 3 | 4.39 | m |
| Basic | 3 | 4.41 | f |
| Giangi | 2 | 4.46 | m |
| Giangi | 3 | 4.47 | m |
| Blanca | 1 | 4.57 | f |
| Bortolo | 1 | 4.59 | m |
| Schiarita | 1 | 4.59 | f |
| Severa | 3 | 4.82 | f |
| Sandro | 3 | 4.98 | m |
| Rocco | 3 | 5 | m |
| Basic | 1 | 5.04 | f |
| Severa | 1 | 5.06 | f |
| Gessica | 3 | 5.11 | f |
| Sciura | 1 | 5.14 | f |
| Barbie | 3 | 5.17 | f |
| Sciura | 1 | 5.18 | f |
| Ghiozza | 1 | 5.23 | f |
| Sonia | 3 | 5.39 | f |
| Roll | 1 | 5.41 | f |
| Schiarita | 3 | 5.48 | f |
| Rocco | 3 | 5.49 | m |
| Sandro | 3 | 5.52 | m |
| Spiga | 1 | 5.59 | f |
| Sfasciato | 1 | 5.68 | m |
| Strip | 3 | 5.77 | f |
| Susy | 1 | 5.85 | f |
| Roll | 3 | 6.34 | f |
| Spilla | 2 | 6.38 | f |
| Giangi | 3 | 6.47 | m |
| Neomamma | 3 | 6.92 | f |
| Grappolo | 3 | 7.07 | f |
| Bigne | 3 | 7.1 | f |
| Roll | 2 | 9.19 | f |
| Giangi | 2 | 10.93 | m |
| Schiarita | 3 | 11.22 | f |

YAWN CONTAGION

| yawn | trigger | typeT | lengthT | receiver | perception | response | audience size | sexT | sexR | groomingindex | proximityindex | spontaneous yawning frequency of the receiver | period |
| --- | --- | --- | --- | --- | --- | --- | --- | --- | --- | --- | --- | --- | --- |
| barbie1.741 | barbie | 1 | 1.74 | bratz | only seen | 0 | 5 | f | f | 0.2692307 | 0 | 0.059322034 | POST |
| barbie1.741 | barbie | 1 | 1.74 | betta | 0 | 0 | 5 | f | f | 0 | 0.2105263 | 0.00408998 | POST |
| barbie1.741 | barbie | 1 | 1.74 | borsa | 0 | 0 | 5 | f | f | 0 | 0.4166667 | 0.067901235 | POST |
| barbie1.741 | barbie | 1 | 1.74 | black | 0 | 0 | 5 | f | m | 0.047619 | 0 | 0.008130081 | POST |
| barbie1.741 | barbie | 1 | 1.74 | bortolo | 0 | 0 | 5 | f | m | 0 | 0 | 0.032051282 | POST |
| barbie1.741 | barbie | 1 | 1.74 | blind | 0 | 0 | 10 | f | f | 0 | 0.12903225 | 0.053763441 | POST |
| barbie1.741 | barbie | 1 | 1.74 | basic | 0 | 0 | 10 | f | f | 0 | 0.2 | 0.053030303 | POST |
| barbie1.741 | barbie | 1 | 1.74 | bigne | 0 | 0 | 10 | f | f | 0 | 0 | 0.038011696 | POST |
| barbie1.741 | barbie | 1 | 1.74 | braccio | 0 | 0 | 10 | f | f | 0 | 0 | 0.02 | POST |
| barbie1.741 | barbie | 1 | 1.74 | blanca | 0 | 0 | 10 | f | f | 0.08 | 0.04 | 0.032921811 | POST |
| barbie1.741 | barbie | 1 | 1.74 | black | 0 | 0 | 10 | f | m | 0.047619 | 0 | 0.008130081 | POST |
| barbie1.741 | barbie | 1 | 1.74 | bernoccolo | 0 | 0 | 10 | f | m | 0 | 0 | 0.01754386 | POST |
| barbie1.741 | barbie | 1 | 1.74 | bortolo | 0 | 0 | 10 | f | m | 0 | 0 | 0.032051282 | POST |
| barbie1.741 | barbie | 1 | 1.74 | betta | 0 | 0 | 10 | f | f | 0 | 0.2105263 | 0.00408998 | POST |
| barbie1.741 | barbie | 1 | 1.74 | bisnonna | 0 | 0 | 10 | f | f | 0.0434782 | 0.0869565 | 0.049019608 | POST |
| barbie2.291 | barbie | 1 | 2.29 | borsa | only seen | 0 | 14 | f | f | 0 | 0.4166667 | 0.067901235 | POST |
| barbie2.291 | barbie | 1 | 2.29 | blanca | 0 | 0 | 14 | f | f | 0.08 | 0.04 | 0.032921811 | POST |
| barbie2.291 | barbie | 1 | 2.29 | basic | 0 | 0 | 14 | f | f | 0 | 0.2 | 0.053030303 | POST |
| barbie2.291 | barbie | 1 | 2.29 | betta | 0 | 0 | 14 | f | f | 0 | 0.2105263 | 0.00408998 | POST |
| barbie2.291 | barbie | 1 | 2.29 | blind | 0 | 0 | 14 | f | f | 0 | 0.12903225 | 0.053763441 | POST |
| barbie2.291 | barbie | 1 | 2.29 | bisnonna | 0 | 0 | 14 | f | f | 0.0434782 | 0.0869565 | 0.049019608 | POST |
| barbie2.291 | barbie | 1 | 2.29 | braccio | 0 | 0 | 14 | f | f | 0 | 0 | 0.02 | POST |
| barbie2.291 | barbie | 1 | 2.29 | bifida | 0 | 0 | 14 | f | f | 0.08 | 0.08 | 0.078014184 | POST |
| barbie2.291 | barbie | 1 | 2.29 | bigne | 0 | 0 | 14 | f | f | 0 | 0 | 0.038011696 | POST |
| barbie2.291 | barbie | 1 | 2.29 | black | 0 | 0 | 14 | f | m | 0.047619 | 0 | 0.008130081 | POST |
| barbie2.291 | barbie | 1 | 2.29 | bernoccolo | 0 | 0 | 14 | f | m | 0 | 0 | 0.01754386 | PRE |
| barbie2.291 | barbie | 1 | 2.29 | bortolo | 0 | 0 | 14 | f | m | 0 | 0 | 0.032051282 | PRE |
| barbie2.291 | barbie | 1 | 2.29 | belly | 0 | 0 | 14 | f | f | 0 | 0 | 0.024390244 | PRE |
| barbie2.291 | barbie | 1 | 2.29 | biba | 0 | 0 | 14 | f | f | 0 | 0 | 0.011111111 | PRE |
| barbie3.493 | barbie | 3 | 3.49 | borsa | only seen | 0 | 2 | f | f | 0 | 0.4166667 | 0.067901235 | PRE |
| barbie3.493 | barbie | 3 | 3.49 | betta | 0 | 0 | 2 | f | f | 0 | 0.2105263 | 0.00408998 | PRE |
| barbie5.183 | barbie | 3 | 5.18 | basic | only seen | 0 | 15 | f | f | 0 | 0.2 | 0.053030303 | PRE |
| barbie5.183 | barbie | 3 | 5.18 | bratz | only seen | 0 | 15 | f | f | 0.2692307 | 0 | 0.059322034 | PRE |
| barbie5.183 | barbie | 3 | 5.18 | betta | only seen | 0 | 15 | f | f | 0 | 0.2105263 | 0.00408998 | PRE |
| barbie5.183 | barbie | 3 | 5.18 | borsa | only seen | 0 | 15 | f | f | 0 | 0.4166667 | 0.067901235 | PRE |
| barbie5.183 | barbie | 3 | 5.18 | bifida | only seen | 0 | 15 | f | f | 0.08 | 0.08 | 0.078014184 | PRE |
| barbie5.183 | barbie | 3 | 5.18 | black | only seen | 0 | 15 | f | m | 0.047619 | 0 | 0.008130081 | PRE |
| barbie5.183 | barbie | 3 | 5.18 | bisnonna | only seen | 0 | 15 | f | f | 0.0434782 | 0.0869565 | 0.049019608 | PRE |
| barbie5.183 | barbie | 3 | 5.18 | biondo | 0 | 0 | 15 | f | m | 0 | 0 | 0.14021164 | PRE |
| barbie5.183 | barbie | 3 | 5.18 | blind | 0 | 0 | 15 | f | f | 0 | 0.12903225 | 0.053763441 | PRE |
| barbie5.183 | barbie | 3 | 5.18 | braccio | 0 | 0 | 15 | f | f | 0 | 0 | 0.02 | PRE |
| barbie5.183 | barbie | 3 | 5.18 | bernoccolo | 0 | 0 | 15 | f | m | 0 | 0 | 0.01754386 | PRE |
| barbie5.183 | barbie | 3 | 5.18 | bortolo | 0 | 0 | 15 | f | m | 0 | 0 | 0.032051282 | PRE |
| barbie5.183 | barbie | 3 | 5.18 | blanca | 0 | 0 | 15 | f | f | 0.08 | 0.04 | 0.032921811 | PRE |
| barbie5.183 | barbie | 3 | 5.18 | belly | 0 | 0 | 15 | f | f | 0 | 0 | 0.024390244 | PRE |
| barbie5.183 | barbie | 3 | 5.18 | biba | 0 | 0 | 15 | f | f | 0 | 0 | 0.011111111 | PRE |
| basic0.753 | basic | 3 | 0.75 | barbie | only seen | 1 | 8 | f | f | 0 | 0.2 | 0.026666667 | POST |
| basic0.753 | basic | 3 | 0.75 | blind | only seen | 1 | 8 | f | f | 0 | 0.0263157 | 0.053763441 | POST |
| basic0.753 | basic | 3 | 0.75 | bigne | only seen | 0 | 8 | f | f | 0.0714286 | 0.1428571 | 0.038011696 | POST |
| basic0.753 | basic | 3 | 0.75 | braccio | only seen | 0 | 8 | f | f | 0 | 0.0833333 | 0.02 | POST |
| basic0.753 | basic | 3 | 0.75 | blanca | 0 | 0 | 8 | f | f | 0.047619 | 0.1428571 | 0.032921811 | POST |
| basic0.753 | basic | 3 | 0.75 | black | 0 | 0 | 8 | f | m | 0 | 0.0869565 | 0.008130081 | POST |
| basic0.753 | basic | 3 | 0.75 | bortolo | 0 | 0 | 8 | f | m | 0 | 0 | 0.032051282 | POST |
| basic0.753 | basic | 3 | 0.75 | bernoccolo | 0 | 0 | 8 | f | m | 0 | 0 | 0.01754386 | POST |
| basic1.383 | basic | 3 | 1.38 | betta | only seen | 0 | 2 | f | f | 0.0731707 | 0.1463415 | 0.00408998 | POST |
| basic1.383 | basic | 3 | 1.38 | biondo | 0 | 0 | 2 | f | m | 0 | 0.0196078 | 0.14021164 | POST |
| basic1.541 | basic | 1 | 1.54 | betta | seen and heard | 0 | 9 | f | f | 0.0731707 | 0.1463415 | 0.00408998 | POST |
| basic1.541 | basic | 1 | 1.54 | biondo | only heard | 1 | 9 | f | m | 0 | 0.0196078 | 0.14021164 | POST |
| basic1.541 | basic | 1 | 1.54 | bisnonna | only heard | 0 | 9 | f | f | 0.0196078 | 0.0980392 | 0.049019608 | POST |
| basic1.541 | basic | 1 | 1.54 | blanca | only heard | 0 | 9 | f | f | 0.047619 | 0.1428571 | 0.032921811 | POST |
| basic1.541 | basic | 1 | 1.54 | bratz | only heard | 0 | 9 | f | f | 0.3111111 | 0.0444444 | 0.059322034 | POST |
| basic1.541 | basic | 1 | 1.54 | bortolo | only heard | 0 | 9 | f | m | 0 | 0 | 0.032051282 | POST |
| basic1.541 | basic | 1 | 1.54 | bifida | only heard | 0 | 9 | f | f | 0 | 0 | 0.078014184 | POST |
| basic1.541 | basic | 1 | 1.54 | bijoux | only heard | 0 | 9 | f | f | 0 | 0 | 0.061403509 | POST |
| basic1.6151 | basic | 1 | 1.615 | biondo | 0 | 0 | 1 | f | m | 0 | 0.0196078 | 0.14021164 | POST |
| basic1.6461 | basic | 1 | 1.646 | betta | only seen | 0 | 5 | f | f | 0.0731707 | 0.1463415 | 0.00408998 | POST |
| basic1.6461 | basic | 1 | 1.646 | bigne | 0 | 0 | 5 | f | f | 0.0714286 | 0.1427581 | 0.038011696 | POST |
| basic1.6461 | basic | 1 | 1.646 | belly | 0 | 0 | 5 | f | f | 0.076923 | 0 | 0.024390244 | POST |
| basic1.6461 | basic | 1 | 1.646 | blanca | 0 | 0 | 5 | f | f | 0.047619 | 0.1428571 | 0.032921811 | POST |
| basic1.6461 | basic | 1 | 1.646 | bisnonna | 0 | 0 | 5 | f | f | 0.0196078 | 0.0980392 | 0.049019608 | POST |
| basic1.7591 | basic | 1 | 1.759 | biondo | only seen | 0 | 2 | f | m | 0 | 0.0196078 | 0.14021164 | POST |
| basic1.7591 | basic | 1 | 1.759 | bisnonna | only seen | 0 | 2 | f | f | 0.0196078 | 0.0980392 | 0.049019608 | POST |
| basic1.861 | basic | 1 | 1.86 | belly | 0 | 0 | 2 | f | f | 0.076923 | 0 | 0.024390244 | POST |
| basic1.861 | basic | 1 | 1.86 | black | 0 | 0 | 2 | f | m | 0 | 0.0869565 | 0.008130081 | POST |
| basic2.151 | basic | 1 | 2.15 | bisnonna | seen and heard | 0 | 3 | f | f | 0.0196078 | 0.0980392 | 0.049019608 | POST |
| basic2.151 | basic | 1 | 2.15 | bratz | only heard | 0 | 3 | f | f | 0.3111111 | 0.0444444 | 0.059322034 | POST |
| basic2.381 | basic | 1 | 2.38 | bisnonna | seen and heard | 0 | 3 | f | f | 0.0196078 | 0.0982392 | 0.049019608 | POST |
| basic2.381 | basic | 1 | 2.38 | bigne | only heard | 1 | 3 | f | f | 0.0714286 | 0.1428571 | 0.038011696 | PRE |
| basic2.413 | basic | 3 | 2.41 | blanca | only seen | 0 | 1 | f | f | 0.047619 | 0.1428571 | 0.032921811 | PRE |
| basic2.7571 | basic | 1 | 2.757 | blanca | 0 | 0 | 5 | f | f | 0.047619 | 0.1428571 | 0.032921811 | PRE |
| basic2.7571 | basic | 1 | 2.757 | bigne | 0 | 0 | 5 | f | f | 0.0714286 | 0.1427581 | 0.038011696 | PRE |
| basic2.7571 | basic | 1 | 2.757 | belly | 0 | 0 | 5 | f | f | 0.076923 | 0 | 0.024390244 | PRE |
| basic2.7571 | basic | 1 | 2.757 | bisnonna | 0 | 0 | 5 | f | f | 0.0196078 | 0.0980392 | 0.049019608 | PRE |
| basic2.973 | basic | 3 | 2.97 | bratz | only seen | 0 | 1 | f | f | 0.3111111 | 0.0444444 | 0.059322034 | PRE |
| basic5.041 | basic | 1 | 5.04 | bisnonna | seen and heard | 0 | 2 | f | f | 0.0196078 | 0.0980392 | 0.049019608 | PRE |
| basic5.041 | basic | 1 | 5.04 | biondo | only heard | 0 | 2 | f | m | 0 | 0.0196078 | 0.14021164 | PRE |
| bernoccolo2.153 | bernoccolo | 3 | 2.15 | blind | 0 | 0 | 11 | m | f | 0 | 0.08 | 0.053763441 | POST |
| bernoccolo2.153 | bernoccolo | 3 | 2.15 | biondo | 0 | 0 | 11 | m | m | 0 | 0 | 0.14021164 | POST |
| bernoccolo2.153 | bernoccolo | 3 | 2.15 | bisnonna | 0 | 0 | 11 | m | f | 0 | 0 | 0.049019608 | POST |
| bernoccolo2.153 | bernoccolo | 3 | 2.15 | bijoux | 0 | 0 | 11 | m | f | 0 | 0 | 0.061403509 | POST |
| bernoccolo2.153 | bernoccolo | 3 | 2.15 | bortolo | 0 | 0 | 11 | m | m | 0.1 | 0.16666667 | 0.032051282 | POST |
| bernoccolo2.153 | bernoccolo | 3 | 2.15 | blanca | 0 | 0 | 11 | m | f | 0 | 0 | 0.032921811 | POST |
| bernoccolo2.153 | bernoccolo | 3 | 2.15 | bratz | 0 | 0 | 11 | m | f | 0 | 0.14285714 | 0.059322034 | POST |
| bernoccolo2.153 | bernoccolo | 3 | 2.15 | bigne | 0 | 0 | 11 | m | f | 0 | 0.2 | 0.038011696 | POST |
| bernoccolo2.153 | bernoccolo | 3 | 2.15 | bifida | 0 | 0 | 11 | m | f | 0 | 0.09090909 | 0.078014184 | POST |
| bernoccolo2.153 | bernoccolo | 3 | 2.15 | basic | 0 | 0 | 11 | m | f | 0 | 0 | 0.053030303 | POST |
| betta2.131 | betta | 1 | 2.13 | bernoccolo | only seen | 0 | 4 | f | m | 0 | 0 | 0.01754386 | POST |
| betta2.131 | betta | 1 | 2.13 | biondo | 0 | 0 | 4 | f | m | 0.4551724 | 0.0965517 | 0.14021164 | POST |
| betta2.131 | betta | 1 | 2.13 | black | 0 | 0 | 4 | f | m | 0.047619 | 0.0238095 | 0.008130081 | POST |
| betta2.131 | betta | 1 | 2.13 | bortolo | 0 | 0 | 4 | f | m | 0 | 0 | 0.032051282 | POST |
| betta2.31 | betta | 1 | 2.3 | biondo | seen and heard | 1 | 1 | f | m | 0.4551724 | 0.0965517 | 0.14021164 | POST |
| betta2.451 | betta | 1 | 2.45 | basic | only seen | 0 | 6 | f | f | 0.0731707 | 0.14634146 | 0.053030303 | POST |
| betta2.451 | betta | 1 | 2.45 | borsa | only seen | 0 | 6 | f | f | 0.016129 | 0.0806452 | 0.067901235 | POST |
| betta2.451 | betta | 1 | 2.45 | bigne | only seen | 0 | 6 | f | f | 0 | 0.0862069 | 0.038011696 | POST |
| betta2.451 | betta | 1 | 2.45 | barbie | 0 | 0 | 6 | f | f | 0 | 0.2105263 | 0.026666667 | PRE |
| betta2.451 | betta | 1 | 2.45 | bifida | 0 | 0 | 6 | f | f | 0 | 0.1153846 | 0.078014184 | PRE |
| betta2.451 | betta | 1 | 2.45 | black | 0 | 0 | 6 | f | m | 0.047619 | 0.0238095 | 0.008130081 | PRE |
| bifida0.8961 | bifida | 1 | 0.896 | bisnonna | 0 | 0 | 1 | f | f | 0 | 0.106383 | 0.049019608 | POST |
| bifida1.11* | bifida | * | 1.11 | bigne | 0 | 0 | 2 | f | f | 0.1764706 | 0.1372549 | 0.038011696 | POST |
| bifida1.11* | bifida | * | 1.11 | basic | 0 | 0 | 2 | f | f | 0 | 0 | 0.053030303 | POST |
| bifida1.281 | bifida | 1 | 1.28 | bigne | seen and heard | 0 | 2 | f | f | 0.1764706 | 0.1372549 | 0.038011696 | POST |
| bifida1.281 | bifida | 1 | 1.28 | biondo | only heard | 0 | 2 | f | m | 0.0135135 | 0.027027 | 0.14021164 | POST |
| bifida1.673 | bifida | 3 | 1.67 | black | 0 | 1 | 2 | f | m | 0.0344828 | 0.1724138 | 0.008130081 | POST |
| bifida1.673 | bifida | 3 | 1.67 | bratz | 0 | 0 | 2 | f | f | 0.0681818 | 0.1590909 | 0.059322034 | POST |
| bifida1.711 | bifida | 1 | 1.71 | black | only seen | 0 | 14 | f | m | 0.0344828 | 0.1724138 | 0.008130081 | POST |
| bifida1.711 | bifida | 1 | 1.71 | bortolo | only seen | 0 | 14 | f | m | 0 | 0 | 0.032051282 | POST |
| bifida1.711 | bifida | 1 | 1.71 | biba | only seen | 0 | 14 | f | f | 0 | 0.2 | 0.011111111 | POST |
| bifida1.711 | bifida | 1 | 1.71 | belly | 0 | 0 | 14 | f | f | 0 | 0.17647058 | 0.024390244 | POST |
| bifida1.711 | bifida | 1 | 1.71 | bernoccolo | 0 | 0 | 14 | f | m | 0 | 0.09090909 | 0.01754386 | POST |
| bifida1.711 | bifida | 1 | 1.71 | bratz | 0 | 0 | 14 | f | f | 0.0681818 | 0.1590909 | 0.059322034 | POST |
| bifida1.711 | bifida | 1 | 1.71 | betta | 0 | 0 | 14 | f | f | 0.0192308 | 0.1153846 | 0.00408998 | POST |
| bifida1.711 | bifida | 1 | 1.71 | basic | 0 | 0 | 14 | f | f | 0 | 0 | 0.053030303 | POST |
| bifida1.711 | bifida | 1 | 1.71 | barbie | 0 | 0 | 14 | f | f | 0.08 | 0.08 | 0.026666667 | POST |
| bifida1.711 | bifida | 1 | 1.71 | blanca | 0 | 0 | 14 | f | f | 0.1538462 | 0.0512821 | 0.032921811 | POST |
| bifida1.711 | bifida | 1 | 1.71 | borsa | 0 | 0 | 14 | f | f | 0 | 0.6666667 | 0.067901235 | POST |
| bifida1.711 | bifida | 1 | 1.71 | bisnonna | 0 | 0 | 14 | f | f | 0 | 0.106383 | 0.049019608 | POST |
| bifida1.711 | bifida | 1 | 1.71 | braccio | 0 | 0 | 14 | f | f | 0.0204082 | 0.122449 | 0.02 | POST |
| bifida1.711 | bifida | 1 | 1.71 | blind | 0 | 0 | 14 | f | f | 0 | 0.025 | 0.053763441 | PRE |
| bifida1.9843 | bifida | 3 | 1.984 | biondo | only heard | 0 | 1 | f | m | 0.0135135 | 0.027027 | 0.14021164 | PRE |
| bifida2.173 | bifida | 3 | 2.17 | blanca | only seen | 0 | 1 | f | f | 0.1538462 | 0.0512821 | 0.032921811 | PRE |
| bifida2.2023 | bifida | 3 | 2.202 | basic | only seen | 0 | 3 | f | f | 0 | 0 | 0.053030303 | PRE |
| bifida2.2023 | bifida | 3 | 2.202 | biondo | only seen | 0 | 3 | f | m | 0.0135135 | 0.027027 | 0.14021164 | PRE |
| bifida2.2023 | bifida | 3 | 2.202 | bisnonna | only seen | 0 | 3 | f | f | 0 | 0.106383 | 0.049019608 | PRE |
| bifida2.3783 | bifida | 3 | 2.378 | biondo | only heard | 0 | 2 | f | m | 0.0135135 | 0.027027 | 0.14021164 | PRE |
| bifida2.3783 | bifida | 3 | 2.378 | basic | only heard | 1 | 2 | f | f | 0 | 0 | 0.053030303 | PRE |
| bifida2.6283 | bifida | 3 | 2.628 | bisnonna | only seen | 0 | 1 | f | f | 0 | 0.106383 | 0.049019608 | PRE |
| bifida2.681 | bifida | 1 | 2.68 | blanca | only seen | 0 | 3 | f | f | 0.1538462 | 0.0512821 | 0.032921811 | PRE |
| bifida2.681 | bifida | 1 | 2.68 | barbie | 0 | 0 | 3 | f | f | 0.08 | 0.08 | 0.026666667 | PRE |
| bifida2.681 | bifida | 1 | 2.68 | bratz | 0 | 0 | 3 | f | f | 0.0681818 | 0.1590909 | 0.059322034 | PRE |
| bigne0.823 | bigne | 3 | 0.82 | biondo | 0 | 0 | 2 | f | m | 0 | 0.1190476 | 0.14021164 | POST |
| bigne0.823 | bigne | 3 | 0.82 | betta | 0 | 0 | 2 | f | f | 0 | 0.0862069 | 0.00408998 | POST |
| bigne1.091 | bigne | 1 | 1.09 | basic | only heard | 0 | 1 | f | f | 0.07142857 | 0.14285714 | 0.053030303 | POST |
| bigne1.31 | bigne | 1 | 1.3 | blanca | 0 | 0 | 1 | f | f | 0 | 0.1914894 | 0.032921811 | POST |
| bigne1.413 | bigne | 3 | 1.41 | blind | only seen | 0 | 6 | f | f | 0.0163934 | 0.1147541 | 0.053763441 | POST |
| bigne1.413 | bigne | 3 | 1.41 | borsa | 0 | 0 | 6 | f | f | 0.2045455 | 0.1136364 | 0.067901235 | POST |
| bigne1.413 | bigne | 3 | 1.41 | blanca | 0 | 0 | 6 | f | f | 0 | 0.1914894 | 0.032921811 | POST |
| bigne1.413 | bigne | 3 | 1.41 | biondo | 0 | 0 | 6 | f | m | 0 | 0.1190476 | 0.14021164 | POST |
| bigne1.413 | bigne | 3 | 1.41 | betta | 0 | 0 | 6 | f | f | 0 | 0.0862069 | 0.00408998 | POST |
| bigne1.413 | bigne | 3 | 1.41 | braccio | 0 | 0 | 6 | f | f | 0.0350877 | 0.17543859 | 0.02 | POST |
| bigne1.421 | bigne | 1 | 1.42 | black | only seen | 0 | 13 | f | m | 0 | 0 | 0.008130081 | POST |
| bigne1.421 | bigne | 1 | 1.42 | basic | 0 | 0 | 13 | f | f | 0.07142857 | 0.14285714 | 0.053030303 | POST |
| bigne1.421 | bigne | 1 | 1.42 | barbie | 0 | 0 | 13 | f | f | 0 | 0 | 0.026666667 | POST |
| bigne1.421 | bigne | 1 | 1.42 | betta | 0 | 0 | 13 | f | f | 0 | 0.0862069 | 0.00408998 | POST |
| bigne1.421 | bigne | 1 | 1.42 | borsa | 0 | 0 | 13 | f | f | 0.2045455 | 0.1136364 | 0.067901235 | POST |
| bigne1.421 | bigne | 1 | 1.42 | blind | 0 | 0 | 13 | f | f | 0.0163934 | 0.1148541 | 0.053763441 | POST |
| bigne1.421 | bigne | 1 | 1.42 | bisnonna | 0 | 0 | 13 | f | f | 0.1527778 | 0.1388889 | 0.049019608 | POST |
| bigne1.421 | bigne | 1 | 1.42 | braccio | 0 | 0 | 13 | f | f | 0.0350877 | 0.17543859 | 0.02 | POST |
| bigne1.421 | bigne | 1 | 1.42 | blanca | 0 | 0 | 13 | f | f | 0 | 0.1914894 | 0.032921811 | POST |
| bigne1.421 | bigne | 1 | 1.42 | bortolo | 0 | 0 | 13 | f | m | 0 | 0.2857143 | 0.032051282 | POST |
| bigne1.421 | bigne | 1 | 1.42 | bernoccolo | 0 | 0 | 13 | f | m | 0 | 0.2 | 0.01754386 | POST |
| bigne1.421 | bigne | 1 | 1.42 | bratz | 0 | 0 | 13 | f | f | 0 | 0.0454545 | 0.059322034 | POST |
| bigne1.421 | bigne | 1 | 1.42 | biba | 0 | 0 | 13 | f | f | 0.21428571 | 0 | 0.011111111 | POST |
| bigne1.443 | bigne | 3 | 1.44 | blanca | only seen | 1 | 5 | f | f | 0 | 0.1914894 | 0.032921811 | POST |
| bigne1.443 | bigne | 3 | 1.44 | biondo | only seen | 1 | 5 | f | m | 0 | 0.1190476 | 0.14021164 | POST |
| bigne1.443 | bigne | 3 | 1.44 | bisnonna | 0 | 0 | 5 | f | f | 0.1527778 | 0.1388889 | 0.049019608 | POST |
| bigne1.443 | bigne | 3 | 1.44 | basic | 0 | 0 | 5 | f | f | 0.07142857 | 0.14285714 | 0.053030303 | POST |
| bigne1.443 | bigne | 3 | 1.44 | blind | 0 | 0 | 5 | f | f | 0.0163934 | 0.1147541 | 0.053763441 | POST |
| bigne1.493 | bigne | 3 | 1.49 | biba | only seen | 0 | 9 | f | f | 0.21428571 | 0 | 0.011111111 | POST |
| bigne1.493 | bigne | 3 | 1.49 | black | only seen | 0 | 9 | f | m | 0 | 0 | 0.008130081 | POST |
| bigne1.493 | bigne | 3 | 1.49 | bisnonna | 0 | 0 | 9 | f | f | 0.1527778 | 0.1388889 | 0.049019608 | POST |
| bigne1.493 | bigne | 3 | 1.49 | basic | 0 | 0 | 9 | f | f | 0.07142857 | 0.14285714 | 0.053030303 | POST |
| bigne1.493 | bigne | 3 | 1.49 | blind | 0 | 0 | 9 | f | f | 0.0163934 | 0.1148541 | 0.053763441 | POST |
| bigne1.493 | bigne | 3 | 1.49 | betta | 0 | 0 | 9 | f | f | 0 | 0.0862069 | 0.00408998 | POST |
| bigne1.493 | bigne | 3 | 1.49 | braccio | 0 | 0 | 9 | f | f | 0.0350877 | 0.17543859 | 0.02 | POST |
| bigne1.493 | bigne | 3 | 1.49 | bernoccolo | 0 | 0 | 9 | f | m | 0 | 0.2 | 0.01754386 | POST |
| bigne1.5711 | bigne | 1 | 1.571 | blanca | 0 | 0 | 7 | f | f | 0 | 0.1914894 | 0.032921811 | POST |
| bigne1.5711 | bigne | 1 | 1.571 | bratz | 0 | 0 | 7 | f | f | 0 | 0.0454545 | 0.059322034 | POST |
| bigne1.5711 | bigne | 1 | 1.571 | belly | 0 | 0 | 7 | f | f | 0.17647058 | 0 | 0.024390244 | POST |
| bigne1.5711 | bigne | 1 | 1.571 | betta | 0 | 0 | 7 | f | f | 0 | 0.0862069 | 0.00408998 | POST |
| bigne1.5711 | bigne | 1 | 1.571 | basic | 0 | 1 | 7 | f | f | 0.07142857 | 0.14285714 | 0.053030303 | POST |
| bigne1.5711 | bigne | 1 | 1.571 | bisnonna | 0 | 0 | 7 | f | f | 0.1527778 | 0.1388889 | 0.049019608 | POST |
| bigne1.5711 | bigne | 1 | 1.571 | biondo | 0 | 1 | 7 | f | m | 0 | 0.1190476 | 0.14021164 | POST |
| bigne1.71 | bigne | 1 | 1.7 | basic | 0 | 0 | 13 | f | f | 0.07142857 | 0.14285714 | 0.053030303 | POST |
| bigne1.71 | bigne | 1 | 1.7 | barbie | 0 | 0 | 13 | f | f | 0 | 0 | 0.026666667 | POST |
| bigne1.71 | bigne | 1 | 1.7 | betta | 0 | 0 | 13 | f | f | 0 | 0.0862069 | 0.00408998 | POST |
| bigne1.71 | bigne | 1 | 1.7 | borsa | 0 | 0 | 13 | f | f | 0.2045455 | 0.1136364 | 0.067901235 | POST |
| bigne1.71 | bigne | 1 | 1.7 | blind | 0 | 0 | 13 | f | f | 0.0163934 | 0.1148541 | 0.053763441 | POST |
| bigne1.71 | bigne | 1 | 1.7 | bisnonna | 0 | 0 | 13 | f | f | 0.1527778 | 0.1388889 | 0.049019608 | POST |
| bigne1.71 | bigne | 1 | 1.7 | braccio | 0 | 0 | 13 | f | f | 0.0350877 | 0.17543859 | 0.02 | POST |
| bigne1.71 | bigne | 1 | 1.7 | blanca | 0 | 0 | 13 | f | f | 0 | 0.1914894 | 0.032921811 | POST |
| bigne1.71 | bigne | 1 | 1.7 | bortolo | 0 | 0 | 13 | f | m | 0 | 0.2857143 | 0.032051282 | POST |
| bigne1.71 | bigne | 1 | 1.7 | bernoccolo | 0 | 0 | 13 | f | m | 0 | 0.2 | 0.01754386 | POST |
| bigne1.71 | bigne | 1 | 1.7 | bratz | 0 | 0 | 13 | f | f | 0 | 0.0454545 | 0.059322034 | PRE |
| bigne1.71 | bigne | 1 | 1.7 | black | 0 | 0 | 13 | f | m | 0 | 0 | 0.008130081 | PRE |
| bigne1.71 | bigne | 1 | 1.7 | biba | 0 | 0 | 13 | f | f | 0.21428571 | 0 | 0.011111111 | PRE |
| bigne1.81 | bigne | 1 | 1.8 | borsa | 0 | 0 | 1 | f | f | 0.2045455 | 0.1136364 | 0.067901235 | PRE |
| bigne1.953 | bigne | 3 | 1.95 | braccio | only seen | 0 | 4 | f | f | 0.0350877 | 0.17543859 | 0.02 | PRE |
| bigne1.953 | bigne | 3 | 1.95 | blanca | only seen | 0 | 4 | f | f | 0 | 0.1914894 | 0.032921811 | PRE |
| bigne1.953 | bigne | 3 | 1.95 | betta | 0 | 0 | 4 | f | f | 0 | 0.0862069 | 0.00408998 | PRE |
| bigne1.953 | bigne | 3 | 1.95 | borsa | 0 | 0 | 4 | f | f | 0.2045455 | 0.1136364 | 0.067901235 | PRE |
| bigne2.013 | bigne | 3 | 2.01 | biba | 0 | 0 | 6 | f | f | 0.21428571 | 0 | 0.011111111 | PRE |
| bigne2.013 | bigne | 3 | 2.01 | blanca | 0 | 0 | 6 | f | f | 0 | 0.1914894 | 0.032921811 | PRE |
| bigne2.013 | bigne | 3 | 2.01 | braccio | 0 | 0 | 6 | f | f | 0.0350877 | 0.17543859 | 0.02 | PRE |
| bigne2.013 | bigne | 3 | 2.01 | blind | 0 | 0 | 6 | f | f | 0.0163934 | 0.1147541 | 0.053763441 | PRE |
| bigne2.013 | bigne | 3 | 2.01 | borsa | 0 | 0 | 6 | f | f | 0.2045455 | 0.1136364 | 0.067901235 | PRE |
| bigne2.013 | bigne | 3 | 2.01 | betta | 0 | 0 | 6 | f | f | 0 | 0.0862069 | 0.00408998 | PRE |
| bigne2.091 | bigne | 1 | 2.09 | bisnonna | 0 | 0 | 1 | f | f | 0.1527778 | 0.1388889 | 0.049019608 | PRE |
| bigne2.251 | bigne | 1 | 2.25 | bisnonna | 0 | 0 | 3 | f | f | 0.1527778 | 0.1388889 | 0.049019608 | PRE |
| bigne2.251 | bigne | 1 | 2.25 | biondo | 0 | 0 | 3 | f | m | 0 | 0.1190476 | 0.14021164 | PRE |
| bigne2.251 | bigne | 1 | 2.25 | bijoux | 0 | 0 | 3 | f | f | 0 | 0.4666667 | 0.061403509 | PRE |
| bigne4.311 | bigne | 1 | 4.31 | blanca | only seen | 0 | 3 | f | f | 0 | 0.1914894 | 0.032921811 | PRE |
| bigne4.311 | bigne | 1 | 4.31 | biondo | 0 | 1 | 3 | f | m | 0 | 0.1190476 | 0.14021164 | PRE |
| bigne4.311 | bigne | 1 | 4.31 | braccio | 0 | 0 | 3 | f | f | 0.0350877 | 0.17543859 | 0.02 | PRE |
| bigne5.07* | bigne | * | 5.07 | biondo | 0 | 0 | 3 | f | m | 0 | 0.1190476 | 0.14021164 | PRE |
| bigne5.07* | bigne | * | 5.07 | betta | 0 | 0 | 3 | f | f | 0 | 0.0862069 | 0.00408998 | PRE |
| bigne7.13 | bigne | 3 | 7.1 | biondo | 0 | 0 | 9 | f | m | 0 | 0.1190476 | 0.14021164 | PRE |
| bigne7.13 | bigne | 3 | 7.1 | bifida | 0 | 0 | 9 | f | f | 0.1764706 | 0.1372549 | 0.078014184 | PRE |
| bigne7.13 | bigne | 3 | 7.1 | betta | 0 | 0 | 9 | f | f | 0 | 0.0862069 | 0.00408998 | PRE |
| bigne7.13 | bigne | 3 | 7.1 | basic | 0 | 0 | 9 | f | f | 0.07142857 | 0.14285714 | 0.053030303 | PRE |
| bigne7.13 | bigne | 3 | 7.1 | bortolo | 0 | 0 | 9 | f | m | 0 | 0.2857143 | 0.032051282 | PRE |
| bigne7.13 | bigne | 3 | 7.1 | bratz | 0 | 0 | 9 | f | f | 0 | 0.0454545 | 0.059322034 | PRE |
| bigne7.13 | bigne | 3 | 7.1 | blanca | 0 | 0 | 9 | f | f | 0 | 0.1914894 | 0.032921811 | PRE |
| bigne7.13 | bigne | 3 | 7.1 | bisnonna | 0 | 0 | 9 | f | f | 0.1527778 | 0.1388889 | 0.049019608 | PRE |
| bigne7.13 | bigne | 3 | 7.1 | bijoux | 0 | 0 | 9 | f | f | 0 | 0.4666667 | 0.061403509 | PRE |
| bijoux1.641 | bijoux | 1 | 1.64 | blind | 0 | 0 | 9 | f | f | 0 | 0 | 0.053763441 | POST |
| bijoux1.641 | bijoux | 1 | 1.64 | basic | 0 | 0 | 9 | f | f | 0 | 0 | 0.053030303 | POST |
| bijoux1.641 | bijoux | 1 | 1.64 | biondo | 0 | 0 | 9 | f | m | 0 | 0.0869565 | 0.14021164 | POST |
| bijoux1.641 | bijoux | 1 | 1.64 | bigne | 0 | 0 | 9 | f | f | 0 | 0.4666667 | 0.038011696 | POST |
| bijoux1.641 | bijoux | 1 | 1.64 | blanca | 0 | 0 | 9 | f | f | 0 | 0 | 0.032921811 | POST |
| bijoux1.641 | bijoux | 1 | 1.64 | bisnonna | 0 | 0 | 9 | f | f | 0 | 0.055555556 | 0.049019608 | POST |
| bijoux1.641 | bijoux | 1 | 1.64 | betta | 0 | 0 | 9 | f | f | 0 | 0.0454545 | 0.00408998 | POST |
| bijoux1.641 | bijoux | 1 | 1.64 | belly | 0 | 0 | 9 | f | f | 0 | 0.285714286 | 0.024390244 | POST |
| bijoux1.641 | bijoux | 1 | 1.64 | bernoccolo | 0 | 0 | 9 | f | m | 0 | 0 | 0.01754386 | POST |
| bijoux1.992 | bijoux | 2 | 1.99 | black | 0 | 0 | 1 | f | m | 0.22222222 | 0.1666667 | 0.008130081 | POST |
| bijoux2.511 | bijoux | 1 | 2.51 | biondo | only heard | 0 | 16 | f | m | 0 | 0.0869565 | 0.14021164 | POST |
| bijoux2.511 | bijoux | 1 | 2.51 | blanca | only heard | 0 | 16 | f | f | 0 | 0 | 0.032921811 | POST |
| bijoux2.511 | bijoux | 1 | 2.51 | barbie | only heard | 0 | 16 | f | f | 0 | 0.191489362 | 0.026666667 | POST |
| bijoux2.511 | bijoux | 1 | 2.51 | basic | only heard | 0 | 16 | f | f | 0 | 0 | 0.053030303 | POST |
| bijoux2.511 | bijoux | 1 | 2.51 | betta | only heard | 0 | 16 | f | f | 0 | 0.0454545 | 0.00408998 | POST |
| bijoux2.511 | bijoux | 1 | 2.51 | borsa | only heard | 0 | 16 | f | f | 0 | 0.3125 | 0.067901235 | POST |
| bijoux2.511 | bijoux | 1 | 2.51 | blind | only heard | 0 | 16 | f | f | 0 | 0 | 0.053763441 | POST |
| bijoux2.511 | bijoux | 1 | 2.51 | bisnonna | only heard | 0 | 16 | f | f | 0 | 0.055555556 | 0.049019608 | POST |
| bijoux2.511 | bijoux | 1 | 2.51 | braccio | only heard | 0 | 16 | f | f | 0 | 0.25 | 0.02 | POST |
| bijoux2.511 | bijoux | 1 | 2.51 | bifida | only heard | 0 | 16 | f | f | 0 | 0 | 0.078014184 | POST |
| bijoux2.511 | bijoux | 1 | 2.51 | bigne | only heard | 0 | 16 | f | f | 0 | 0.4666667 | 0.038011696 | POST |
| bijoux2.511 | bijoux | 1 | 2.51 | black | only heard | 0 | 16 | f | m | 0.22222222 | 0.1666667 | 0.008130081 | POST |
| bijoux2.511 | bijoux | 1 | 2.51 | bernoccolo | only heard | 0 | 16 | f | m | 0 | 0 | 0.01754386 | POST |
| bijoux2.511 | bijoux | 1 | 2.51 | bortolo | only heard | 1 | 16 | f | m | 0.15384615 | 0 | 0.032051282 | POST |
| bijoux2.511 | bijoux | 1 | 2.51 | belly | only heard | 0 | 16 | f | f | 0 | 0.191489362 | 0.024390244 | POST |
| bijoux2.511 | bijoux | 1 | 2.51 | biba | only heard | 0 | 16 | f | f | 0 | 0.3333333 | 0.011111111 | POST |
| bijoux2.921 | bijoux | 1 | 2.92 | black | 0 | 0 | 1 | f | m | 0.22222222 | 0.1666667 | 0.008130081 | POST |
| biondo0.911 | biondo | 1 | 0.91 | betta | only heard | 0 | 1 | m | f | 0.4551724 | 0.0965517 | 0.00408998 | POST |
| biondo0.941 | biondo | 1 | 0.94 | bisnonna | only heard | 0 | 2 | m | f | 0.0571428 | 0.1333333 | 0.049019608 | POST |
| biondo0.941 | biondo | 1 | 0.94 | betta | only heard | 0 | 2 | m | f | 0.4551724 | 0.0965517 | 0.00408998 | POST |
| biondo1.0161 | biondo | 1 | 1.016 | bisnonna | only heard | 0 | 1 | m | f | 0.0571428 | 0.1333333 | 0.049019608 | POST |
| biondo1.033 | biondo | 3 | 1.03 | braccio | seen and heard | 0 | 4 | m | f | 0.1209677 | 0.1048387 | 0.02 | POST |
| biondo1.033 | biondo | 3 | 1.03 | betta | only heard | 1 | 4 | m | f | 0.4551724 | 0.0965517 | 0.00408998 | POST |
| biondo1.033 | biondo | 3 | 1.03 | bernoccolo | only heard | 0 | 4 | m | m | 0 | 0 | 0.01754386 | POST |
| biondo1.033 | biondo | 3 | 1.03 | bortolo | only heard | 1 | 4 | m | m | 0 | 0.0588235 | 0.032051282 | POST |
| biondo1.042 | biondo | 2 | 1.04 | betta | only heard | 0 | 1 | m | f | 0.4551724 | 0.0965517 | 0.00408998 | POST |
| biondo1.052 | biondo | 2 | 1.05 | braccio | only heard | 0 | 7 | m | f | 0.1209677 | 0.1048387 | 0.02 | POST |
| biondo1.052 | biondo | 2 | 1.05 | bijoux | only heard | 0 | 7 | m | f | 0 | 0.0869565 | 0.061403509 | POST |
| biondo1.052 | biondo | 2 | 1.05 | black | only heard | 0 | 7 | m | m | 0 | 0.02 | 0.008130081 | POST |
| biondo1.052 | biondo | 2 | 1.05 | barbie | only heard | 0 | 7 | m | f | 0 | 0 | 0.026666667 | POST |
| biondo1.052 | biondo | 2 | 1.05 | blanca | only heard | 0 | 7 | m | f | 0 | 0.0491803 | 0.032921811 | POST |
| biondo1.052 | biondo | 2 | 1.05 | blind | only heard | 0 | 7 | m | f | 0 | 0.1553398 | 0.053763441 | POST |
| biondo1.052 | biondo | 2 | 1.05 | betta | only heard | 0 | 7 | m | f | 0.4551724 | 0.0965517 | 0.00408998 | POST |
| biondo1.11 | biondo | 1 | 1.1 | black | seen and heard | 0 | 12 | m | m | 0 | 0.02 | 0.008130081 | POST |
| biondo1.11 | biondo | 1 | 1.1 | bifida | seen and heard | 0 | 12 | m | f | 0.0135135 | 0.027027 | 0.078014184 | POST |
| biondo1.11 | biondo | 1 | 1.1 | bigne | seen and heard | 1 | 12 | m | f | 0 | 0.1190476 | 0.038011696 | POST |
| biondo1.11 | biondo | 1 | 1.1 | bernoccolo | only heard | 0 | 12 | m | m | 0 | 0 | 0.01754386 | POST |
| biondo1.11 | biondo | 1 | 1.1 | braccio | only heard | 0 | 12 | m | f | 0.1209677 | 0.1048387 | 0.02 | POST |
| biondo1.11 | biondo | 1 | 1.1 | blind | only heard | 0 | 12 | m | f | 0 | 0.1553398 | 0.053763441 | POST |
| biondo1.11 | biondo | 1 | 1.1 | bisnonna | only heard | 0 | 12 | m | f | 0.0571428 | 0.1333333 | 0.049019608 | POST |
| biondo1.11 | biondo | 1 | 1.1 | borsa | only heard | 0 | 12 | m | f | 0.0547945 | 0.109589 | 0.067901235 | POST |
| biondo1.11 | biondo | 1 | 1.1 | betta | only heard | 0 | 12 | m | f | 0.4551724 | 0.0965517 | 0.00408998 | POST |
| biondo1.11 | biondo | 1 | 1.1 | barbie | only heard | 0 | 12 | m | f | 0 | 0 | 0.026666667 | POST |
| biondo1.11 | biondo | 1 | 1.1 | basic | only heard | 0 | 12 | m | f | 0 | 0.0196078 | 0.053030303 | POST |
| biondo1.11 | biondo | 1 | 1.1 | blanca | only heard | 1 | 12 | m | f | 0 | 0.0491803 | 0.032921811 | POST |
| biondo1.111 | biondo | 1 | 1.11 | blind | only heard | 0 | 3 | m | f | 0 | 0.1553398 | 0.053763441 | POST |
| biondo1.111 | biondo | 1 | 1.11 | bisnonna | only heard | 1 | 3 | m | f | 0.0571428 | 0.1333333 | 0.049019608 | POST |
| biondo1.111 | biondo | 1 | 1.11 | bigne | only heard | 0 | 3 | m | f | 0 | 0.1190476 | 0.038011696 | POST |
| biondo1.121 | biondo | 1 | 1.12 | braccio | only seen | 0 | 7 | m | f | 0.1209677 | 0.1048387 | 0.02 | POST |
| biondo1.121 | biondo | 1 | 1.12 | bratz | only seen | 1 | 7 | m | f | 0 | 0.0740741 | 0.059322034 | POST |
| biondo1.121 | biondo | 1 | 1.12 | borsa | 0 | 0 | 7 | m | f | 0.0547945 | 0.109589 | 0.067901235 | POST |
| biondo1.121 | biondo | 1 | 1.12 | bigne | 0 | 0 | 7 | m | f | 0 | 0.1190476 | 0.038011696 | POST |
| biondo1.121 | biondo | 1 | 1.12 | betta | 0 | 0 | 7 | m | f | 0.4551724 | 0.0965517 | 0.00408998 | POST |
| biondo1.121 | biondo | 1 | 1.12 | blind | 0 | 0 | 7 | m | f | 0 | 0.1553398 | 0.053763441 | POST |
| biondo1.133 | biondo | 3 | 1.13 | bijoux | only heard | 0 | 12 | m | f | 0 | 0.0869565 | 0.061403509 | POST |
| biondo1.133 | biondo | 3 | 1.13 | bisnonna | only heard | 0 | 12 | m | f | 0.0571428 | 0.1333333 | 0.049019608 | POST |
| biondo1.133 | biondo | 3 | 1.13 | bifida | only heard | 0 | 12 | m | f | 0.0135135 | 0.027027 | 0.078014184 | POST |
| biondo1.133 | biondo | 3 | 1.13 | bigne | only heard | 0 | 12 | m | f | 0 | 0.1190476 | 0.038011696 | POST |
| biondo1.133 | biondo | 3 | 1.13 | bortolo | only heard | 0 | 12 | m | m | 0 | 0.0588235 | 0.032051282 | POST |
| biondo1.133 | biondo | 3 | 1.13 | blanca | only heard | 0 | 12 | m | f | 0 | 0.0491803 | 0.032921811 | POST |
| biondo1.133 | biondo | 3 | 1.13 | betta | only heard | 0 | 12 | m | f | 0.4551724 | 0.0965517 | 0.00408998 | POST |
| biondo1.133 | biondo | 3 | 1.13 | basic | only heard | 0 | 12 | m | f | 0 | 0.0196078 | 0.053030303 | POST |
| biondo1.133 | biondo | 3 | 1.13 | bratz | only heard | 0 | 12 | m | f | 0 | 0.0740741 | 0.059322034 | POST |
| biondo1.133 | biondo | 3 | 1.13 | braccio | only heard | 0 | 12 | m | f | 0.1209677 | 0.1048387 | 0.02 | POST |
| biondo1.133 | biondo | 3 | 1.13 | blind | only heard | 1 | 12 | m | f | 0 | 0.1553398 | 0.053763441 | POST |
| biondo1.133 | biondo | 3 | 1.13 | bernoccolo | only heard | 1 | 12 | m | m | 0 | 0 | 0.01754386 | POST |
| biondo1.1983 | biondo | 3 | 1.198 | biba | seen and heard | 0 | 5 | m | f | 0 | 0 | 0.011111111 | POST |
| biondo1.1983 | biondo | 3 | 1.198 | braccio | seen and heard | 0 | 5 | m | f | 0.1209677 | 0.1048387 | 0.02 | POST |
| biondo1.1983 | biondo | 3 | 1.198 | betta | seen and heard | 0 | 5 | m | f | 0.4551724 | 0.0965517 | 0.00408998 | POST |
| biondo1.1983 | biondo | 3 | 1.198 | black | only heard | 0 | 5 | m | m | 0 | 0.02 | 0.008130081 | POST |
| biondo1.1983 | biondo | 3 | 1.198 | giangi | only heard | 1 | 5 | m | m | 0 | 0 | 0.215686275 | POST |
| biondo1.22* | biondo | * | 1.22 | betta | 0 | 0 | 3 | m | f | 0.4551724 | 0.0965517 | 0.00408998 | POST |
| biondo1.22* | biondo | * | 1.22 | borsa | 0 | 0 | 3 | m | f | 0.0547945 | 0.109589 | 0.067901235 | POST |
| biondo1.22* | biondo | * | 1.22 | bernoccolo | 0 | 0 | 3 | m | m | 0 | 0 | 0.01754386 | POST |
| biondo1.233 | biondo | 3 | 1.23 | blind | only heard | 0 | 2 | m | f | 0 | 0.1553398 | 0.053763441 | POST |
| biondo1.233 | biondo | 3 | 1.23 | bigne | only heard | 0 | 2 | m | f | 0 | 0.1190476 | 0.038011696 | POST |
| biondo1.242 | biondo | 2 | 1.24 | betta | 0 | 0 | 1 | m | f | 0.4551724 | 0.0965517 | 0.00408998 | POST |
| biondo1.283 | biondo | 3 | 1.28 | braccio | only heard | 0 | 1 | m | f | 0.1209677 | 0.1048387 | 0.02 | POST |
| biondo1.281 | biondo | 1 | 1.28 | braccio | seen and heard | 0 | 6 | m | f | 0.1209677 | 0.1048387 | 0.02 | POST |
| biondo1.281 | biondo | 1 | 1.28 | black | seen and heard | 0 | 6 | m | m | 0 | 0.02 | 0.008130081 | POST |
| biondo1.281 | biondo | 1 | 1.28 | belly | seen and heard | 0 | 6 | m | f | 0 | 0 | 0.024390244 | POST |
| biondo1.281 | biondo | 1 | 1.28 | bifida | 0 | 0 | 6 | m | f | 0.0135135 | 0.027027 | 0.078014184 | POST |
| biondo1.281 | biondo | 1 | 1.28 | bratz | 0 | 0 | 6 | m | f | 0 | 0.0740741 | 0.059322034 | POST |
| biondo1.281 | biondo | 1 | 1.28 | betta | 0 | 0 | 6 | m | f | 0.4551724 | 0.0965517 | 0.00408998 | POST |
| biondo1.33 | biondo | 3 | 1.3 | blind | only seen | 0 | 10 | m | f | 0 | 0.1553398 | 0.053763441 | POST |
| biondo1.33 | biondo | 3 | 1.3 | bijoux | only seen | 0 | 10 | m | f | 0 | 0.0869565 | 0.061403509 | POST |
| biondo1.33 | biondo | 3 | 1.3 | bortolo | only seen | 0 | 10 | m | m | 0 | 0.0588235 | 0.032051282 | POST |
| biondo1.33 | biondo | 3 | 1.3 | borsa | only seen | 0 | 10 | m | f | 0.0547945 | 0.109589 | 0.067901235 | POST |
| biondo1.33 | biondo | 3 | 1.3 | barbie | only seen | 0 | 10 | m | f | 0 | 0 | 0.026666667 | POST |
| biondo1.33 | biondo | 3 | 1.3 | betta | only seen | 0 | 10 | m | f | 0.4551724 | 0.0965517 | 0.00408998 | POST |
| biondo1.33 | biondo | 3 | 1.3 | bifida | 0 | 0 | 10 | m | f | 0.0135135 | 0.027027 | 0.078014184 | POST |
| biondo1.33 | biondo | 3 | 1.3 | black | 0 | 0 | 10 | m | m | 0 | 0.02 | 0.008130081 | POST |
| biondo1.33 | biondo | 3 | 1.3 | bernoccolo | 0 | 0 | 10 | m | m | 0 | 0 | 0.01754386 | POST |
| biondo1.443 | biondo | 3 | 1.44 | barbie | 0 | 0 | 6 | m | f | 0 | 0 | 0.026666667 | POST |
| biondo1.443 | biondo | 3 | 1.44 | betta | 0 | 0 | 6 | m | f | 0.4551724 | 0.0965517 | 0.00408998 | POST |
| biondo1.443 | biondo | 3 | 1.44 | bratz | 0 | 0 | 6 | m | f | 0 | 0.0740741 | 0.059322034 | POST |
| biondo1.443 | biondo | 3 | 1.44 | braccio | 0 | 0 | 6 | m | f | 0.1209677 | 0.1048387 | 0.02 | POST |
| biondo1.443 | biondo | 3 | 1.44 | borsa | 0 | 0 | 6 | m | f | 0.0547945 | 0.109589 | 0.067901235 | POST |
| biondo1.443 | biondo | 3 | 1.44 | bifida | 0 | 0 | 6 | m | f | 0.0135135 | 0.027027 | 0.078014184 | POST |
| biondo1.452 | biondo | 2 | 1.45 | betta | seen and heard | 0 | 6 | m | f | 0.4551724 | 0.0965517 | 0.00408998 | POST |
| biondo1.452 | biondo | 2 | 1.45 | barbie | seen and heard | 0 | 6 | m | f | 0 | 0 | 0.026666667 | POST |
| biondo1.452 | biondo | 2 | 1.45 | blanca | only heard | 0 | 6 | m | f | 0 | 0.0491803 | 0.032921811 | POST |
| biondo1.452 | biondo | 2 | 1.45 | bratz | only heard | 0 | 6 | m | f | 0 | 0.0740741 | 0.059322034 | POST |
| biondo1.452 | biondo | 2 | 1.45 | bigne | only heard | 0 | 6 | m | f | 0 | 0.1190476 | 0.038011696 | POST |
| biondo1.452 | biondo | 2 | 1.45 | braccio | seen and heard | 0 | 6 | m | f | 0.1209677 | 0.1048387 | 0.02 | POST |
| biondo1.452 | biondo | 2 | 1.45 | blanca | only heard | 0 | 6 | m | f | 0 | 0.0491803 | 0.032921811 | POST |
| biondo1.452 | biondo | 2 | 1.45 | bisnonna | only heard | 0 | 6 | m | f | 0.0571428 | 0.1333333 | 0.049019608 | POST |
| biondo1.452 | biondo | 2 | 1.45 | blind | only heard | 0 | 6 | m | f | 0 | 0.1553398 | 0.053763441 | POST |
| biondo1.452 | biondo | 2 | 1.45 | betta | only heard | 0 | 6 | m | f | 0.4551724 | 0.0965517 | 0.00408998 | POST |
| biondo1.452 | biondo | 2 | 1.45 | bratz | only heard | 0 | 6 | m | f | 0 | 0.0740741 | 0.059322034 | POST |
| biondo1.463 | biondo | 3 | 1.46 | bifida | only heard | 0 | 5 | m | f | 0.0135135 | 0.027027 | 0.078014184 | POST |
| biondo1.463 | biondo | 3 | 1.46 | bisnonna | only heard | 0 | 5 | m | f | 0.0571428 | 0.1333333 | 0.049019608 | POST |
| biondo1.463 | biondo | 3 | 1.46 | bigne | only heard | 0 | 5 | m | f | 0 | 0.1190476 | 0.038011696 | POST |
| biondo1.463 | biondo | 3 | 1.46 | blind | only heard | 0 | 5 | m | f | 0 | 0.1553398 | 0.053763441 | POST |
| biondo1.463 | biondo | 3 | 1.46 | braccio | only heard | 0 | 5 | m | f | 0.1209677 | 0.1048387 | 0.02 | POST |
| biondo1.482 | biondo | 2 | 1.48 | betta | seen and heard | 0 | 4 | m | f | 0.4551724 | 0.0965517 | 0.00408998 | POST |
| biondo1.482 | biondo | 2 | 1.48 | blind | only heard | 0 | 4 | m | f | 0 | 0.1553398 | 0.053763441 | POST |
| biondo1.482 | biondo | 2 | 1.48 | bisnonna | only heard | 0 | 4 | m | f | 0.0571428 | 0.1333333 | 0.049019608 | POST |
| biondo1.482 | biondo | 2 | 1.48 | basic | only heard | 0 | 4 | m | f | 0 | 0.0196078 | 0.053030303 | POST |
| biondo1.521 | biondo | 1 | 1.52 | black | 0 | 0 | 8 | m | m | 0 | 0.02 | 0.008130081 | POST |
| biondo1.521 | biondo | 1 | 1.52 | blanca | 0 | 0 | 8 | m | f | 0 | 0.0491803 | 0.032921811 | POST |
| biondo1.521 | biondo | 1 | 1.52 | bernoccolo | 0 | 0 | 8 | m | m | 0 | 0 | 0.01754386 | POST |
| biondo1.521 | biondo | 1 | 1.52 | braccio | 0 | 0 | 8 | m | f | 0.1209677 | 0.1048387 | 0.02 | POST |
| biondo1.521 | biondo | 1 | 1.52 | bratz | 0 | 0 | 8 | m | f | 0 | 0.0740741 | 0.059322034 | POST |
| biondo1.521 | biondo | 1 | 1.52 | bifida | 0 | 0 | 8 | m | f | 0.0135135 | 0.027027 | 0.078014184 | POST |
| biondo1.521 | biondo | 1 | 1.52 | blind | 0 | 0 | 8 | m | f | 0 | 0.1553398 | 0.053763441 | POST |
| biondo1.521 | biondo | 1 | 1.52 | bisnonna | 0 | 0 | 8 | m | f | 0.0571428 | 0.1333333 | 0.049019608 | POST |
| biondo1.543 | biondo | 3 | 1.54 | black | seen and heard | 0 | 9 | m | m | 0 | 0.02 | 0.008130081 | POST |
| biondo1.543 | biondo | 3 | 1.54 | borsa | seen and heard | 0 | 9 | m | f | 0.0547945 | 0.109589 | 0.067901235 | POST |
| biondo1.543 | biondo | 3 | 1.54 | betta | seen and heard | 0 | 9 | m | f | 0.4551724 | 0.0965517 | 0.00408998 | POST |
| biondo1.543 | biondo | 3 | 1.54 | blind | 0 | 0 | 9 | m | f | 0 | 0.1553398 | 0.053763441 | POST |
| biondo1.543 | biondo | 3 | 1.54 | basic | 0 | 0 | 9 | m | f | 0 | 0.0196078 | 0.053030303 | POST |
| biondo1.543 | biondo | 3 | 1.54 | barbie | 0 | 0 | 9 | m | f | 0 | 0 | 0.026666667 | POST |
| biondo1.543 | biondo | 3 | 1.54 | bijoux | 0 | 0 | 9 | m | f | 0 | 0.0869565 | 0.061403509 | POST |
| biondo1.543 | biondo | 3 | 1.54 | belly | 0 | 0 | 9 | m | f | 0 | 0 | 0.024390244 | POST |
| biondo1.543 | biondo | 3 | 1.54 | braccio | 0 | 0 | 9 | m | f | 0.1209677 | 0.1048387 | 0.02 | POST |
| biondo1.551 | biondo | 1 | 1.55 | betta | seen and heard | 0 | 3 | m | f | 0.4551724 | 0.0965517 | 0.00408998 | POST |
| biondo1.551 | biondo | 1 | 1.55 | basic | only heard | 0 | 3 | m | f | 0 | 0.0196078 | 0.053030303 | POST |
| biondo1.551 | biondo | 1 | 1.55 | braccio | only heard | 1 | 3 | m | f | 0.1209677 | 0.1048387 | 0.02 | POST |
| biondo1.572 | biondo | 2 | 1.57 | giangi | seen and heard | 1 | 7 | m | m | 0 | 0 | 0.215686275 | POST |
| biondo1.572 | biondo | 2 | 1.57 | ghirlanda | only heard | 0 | 7 | m | f | 0 | 0 | 0.023148148 | POST |
| biondo1.572 | biondo | 2 | 1.57 | gessica | only heard | 0 | 7 | m | f | 0 | 0 | 0.039735099 | POST |
| biondo1.572 | biondo | 2 | 1.57 | gianna | only heard | 0 | 7 | m | f | 0 | 0 | 0.03163017 | POST |
| biondo1.572 | biondo | 2 | 1.57 | gigetto | only heard | 0 | 7 | m | m | 0 | 0 | 0.039548023 | POST |
| biondo1.583 | biondo | 3 | 1.58 | bifida | seen and heard | 0 | 4 | m | f | 0.0135135 | 0.027027 | 0.078014184 | POST |
| biondo1.583 | biondo | 3 | 1.58 | barbie | seen and heard | 0 | 4 | m | f | 0 | 0 | 0.026666667 | POST |
| biondo1.583 | biondo | 3 | 1.58 | borsa | seen and heard | 0 | 4 | m | f | 0.0547945 | 0.109589 | 0.067901235 | POST |
| biondo1.593 | biondo | 3 | 1.59 | borsa | only heard | 0 | 4 | m | f | 0.0547945 | 0.109589 | 0.067901235 | POST |
| biondo1.593 | biondo | 3 | 1.59 | bisnonna | only heard | 0 | 4 | m | f | 0.0571428 | 0.1333333 | 0.049019608 | POST |
| biondo1.593 | biondo | 3 | 1.59 | braccio | only heard | 0 | 4 | m | f | 0.1209677 | 0.1048387 | 0.02 | POST |
| biondo1.593 | biondo | 3 | 1.59 | betta | only heard | 0 | 4 | m | f | 0.4551724 | 0.0965517 | 0.00408998 | POST |
| biondo1.613 | biondo | 3 | 1.61 | betta | only seen | 0 | 6 | m | f | 0.4551724 | 0.0965517 | 0.00408998 | POST |
| biondo1.613 | biondo | 3 | 1.61 | blind | only seen | 1 | 6 | m | f | 0 | 0.1553398 | 0.053763441 | POST |
| biondo1.613 | biondo | 3 | 1.61 | bratz | only seen | 0 | 6 | m | f | 0 | 0.0740741 | 0.059322034 | POST |
| biondo1.613 | biondo | 3 | 1.61 | bigne | only seen | 0 | 6 | m | f | 0 | 0.1190476 | 0.038011696 | POST |
| biondo1.613 | biondo | 3 | 1.61 | braccio | 0 | 0 | 6 | m | f | 0.1209677 | 0.1048387 | 0.02 | POST |
| biondo1.613 | biondo | 3 | 1.61 | blanca | 0 | 0 | 6 | m | f | 0 | 0.0491803 | 0.032921811 | POST |
| biondo1.623 | biondo | 3 | 1.62 | betta | seen and heard | 0 | 3 | m | f | 0.4551724 | 0.0965517 | 0.00408998 | POST |
| biondo1.623 | biondo | 3 | 1.62 | braccio | only heard | 1 | 3 | m | f | 0.1209677 | 0.1048387 | 0.02 | POST |
| biondo1.623 | biondo | 3 | 1.62 | bortolo | only heard | 0 | 3 | m | m | 0 | 0.0588235 | 0.032051282 | POST |
| biondo1.633 | biondo | 3 | 1.63 | blind | seen and heard | 0 | 13 | m | f | 0 | 0.1553398 | 0.053763441 | POST |
| biondo1.633 | biondo | 3 | 1.63 | borsa | seen and heard | 0 | 13 | m | f | 0.0547945 | 0.109589 | 0.067901235 | POST |
| biondo1.633 | biondo | 3 | 1.63 | bifida | seen and heard | 0 | 13 | m | f | 0.0135135 | 0.027027 | 0.078014184 | POST |
| biondo1.633 | biondo | 3 | 1.63 | belly | seen and heard | 0 | 13 | m | f | 0 | 0 | 0.024390244 | POST |
| biondo1.633 | biondo | 3 | 1.63 | betta | only heard | 0 | 13 | m | f | 0.4551724 | 0.0965517 | 0.00408998 | POST |
| biondo1.633 | biondo | 3 | 1.63 | black | only heard | 0 | 13 | m | m | 0 | 0.02 | 0.008130081 | POST |
| biondo1.633 | biondo | 3 | 1.63 | bijoux | only heard | 0 | 13 | m | f | 0 | 0.0869565 | 0.061403509 | POST |
| biondo1.633 | biondo | 3 | 1.63 | bortolo | only heard | 0 | 13 | m | m | 0 | 0.0588235 | 0.032051282 | POST |
| biondo1.633 | biondo | 3 | 1.63 | bernoccolo | only heard | 0 | 13 | m | m | 0 | 0 | 0.01754386 | POST |
| biondo1.633 | biondo | 3 | 1.63 | bigne | only heard | 0 | 13 | m | f | 0 | 0.1190476 | 0.038011696 | POST |
| biondo1.633 | biondo | 3 | 1.63 | braccio | only heard | 0 | 13 | m | f | 0.1209677 | 0.1048387 | 0.02 | POST |
| biondo1.633 | biondo | 3 | 1.63 | blanca | only heard | 0 | 13 | m | f | 0 | 0.0491803 | 0.032921811 | POST |
| biondo1.633 | biondo | 3 | 1.63 | bratz | only heard | 1 | 13 | m | f | 0 | 0.0740741 | 0.059322034 | POST |
| biondo1.652 | biondo | 2 | 1.65 | barbie | seen and heard | 0 | 7 | m | f | 0 | 0 | 0.026666667 | POST |
| biondo1.652 | biondo | 2 | 1.65 | braccio | seen and heard | 0 | 7 | m | f | 0.1209677 | 0.1048387 | 0.02 | POST |
| biondo1.652 | biondo | 2 | 1.65 | blind | only heard | 0 | 7 | m | f | 0 | 0.1553398 | 0.053763441 | POST |
| biondo1.652 | biondo | 2 | 1.65 | bernoccolo | only heard | 0 | 7 | m | m | 0 | 0 | 0.01754386 | POST |
| biondo1.652 | biondo | 2 | 1.65 | bortolo | only heard | 0 | 7 | m | m | 0 | 0.0588235 | 0.032051282 | POST |
| biondo1.652 | biondo | 2 | 1.65 | betta | only heard | 0 | 7 | m | f | 0.4551724 | 0.0965517 | 0.00408998 | POST |
| biondo1.661 | biondo | 1 | 1.66 | betta | seen and heard | 0 | 6 | m | f | 0.4551724 | 0.0965517 | 0.00408998 | POST |
| biondo1.661 | biondo | 1 | 1.66 | barbie | seen and heard | 0 | 6 | m | f | 0 | 0 | 0.026666667 | POST |
| biondo1.661 | biondo | 1 | 1.66 | bisnonna | only heard | 0 | 6 | m | f | 0.0571428 | 0.1333333 | 0.049019608 | POST |
| biondo1.661 | biondo | 1 | 1.66 | blind | only heard | 0 | 6 | m | f | 0 | 0.1553398 | 0.053763441 | POST |
| biondo1.661 | biondo | 1 | 1.66 | biba | only heard | 0 | 6 | m | f | 0 | 0 | 0.011111111 | POST |
| biondo1.661 | biondo | 1 | 1.66 | bigne | only heard | 0 | 6 | m | f | 0 | 0.1190476 | 0.038011696 | POST |
| biondo1.663 | biondo | 3 | 1.66 | bisnonna | seen and heard | 0 | 12 | m | f | 0.0571428 | 0.1333333 | 0.049019608 | POST |
| biondo1.663 | biondo | 3 | 1.66 | borsa | seen and heard | 0 | 12 | m | f | 0.0547945 | 0.109589 | 0.067901235 | POST |
| biondo1.663 | biondo | 3 | 1.66 | betta | only heard | 0 | 12 | m | f | 0.4551724 | 0.0965517 | 0.00408998 | POST |
| biondo1.663 | biondo | 3 | 1.66 | blind | only heard | 0 | 12 | m | f | 0 | 0.1553398 | 0.053763441 | POST |
| biondo1.663 | biondo | 3 | 1.66 | black | only heard | 0 | 12 | m | m | 0 | 0.02 | 0.008130081 | POST |
| biondo1.663 | biondo | 3 | 1.66 | bijoux | only heard | 0 | 12 | m | f | 0 | 0.0869565 | 0.061403509 | POST |
| biondo1.663 | biondo | 3 | 1.66 | bortolo | only heard | 0 | 12 | m | m | 0 | 0.0588235 | 0.032051282 | POST |
| biondo1.663 | biondo | 3 | 1.66 | bernoccolo | only heard | 0 | 12 | m | m | 0 | 0 | 0.01754386 | POST |
| biondo1.663 | biondo | 3 | 1.66 | bigne | only heard | 0 | 12 | m | f | 0 | 0.1190476 | 0.038011696 | POST |
| biondo1.663 | biondo | 3 | 1.66 | braccio | only heard | 1 | 12 | m | f | 0.1209677 | 0.1048387 | 0.02 | POST |
| biondo1.663 | biondo | 3 | 1.66 | blanca | only heard | 0 | 12 | m | f | 0 | 0.0491803 | 0.032921811 | POST |
| biondo1.663 | biondo | 3 | 1.66 | belly | only heard | 0 | 12 | m | f | 0 | 0 | 0.024390244 | POST |
| biondo1.723 | biondo | 3 | 1.72 | bisnonna | seen and heard | 0 | 8 | m | f | 0.0571428 | 0.1333333 | 0.049019608 | POST |
| biondo1.723 | biondo | 3 | 1.72 | barbie | seen and heard | 0 | 8 | m | f | 0 | 0 | 0.026666667 | POST |
| biondo1.723 | biondo | 3 | 1.72 | bigne | only heard | 0 | 8 | m | f | 0 | 0.1190476 | 0.038011696 | POST |
| biondo1.723 | biondo | 3 | 1.72 | blind | only heard | 0 | 8 | m | f | 0 | 0.1553398 | 0.053763441 | POST |
| biondo1.723 | biondo | 3 | 1.72 | bratz | only heard | 0 | 8 | m | f | 0 | 0.0740741 | 0.059322034 | POST |
| biondo1.723 | biondo | 3 | 1.72 | braccio | only heard | 0 | 8 | m | f | 0.1209677 | 0.1048387 | 0.02 | POST |
| biondo1.723 | biondo | 3 | 1.72 | betta | only heard | 0 | 8 | m | f | 0.4551724 | 0.0965517 | 0.00408998 | POST |
| biondo1.723 | biondo | 3 | 1.72 | borsa | only heard | 1 | 8 | m | f | 0.0547945 | 0.109589 | 0.067901235 | POST |
| biondo1.723 | biondo | 3 | 1.72 | betta | seen and heard | 0 | 6 | m | f | 0.4551724 | 0.0965517 | 0.00408998 | POST |
| biondo1.723 | biondo | 3 | 1.72 | basic | seen and heard | 1 | 6 | m | f | 0 | 0.0196078 | 0.053030303 | POST |
| biondo1.723 | biondo | 3 | 1.72 | bratz | seen and heard | 0 | 6 | m | f | 0 | 0.0740741 | 0.059322034 | POST |
| biondo1.723 | biondo | 3 | 1.72 | blind | seen and heard | 0 | 6 | m | f | 0 | 0.1553398 | 0.053763441 | POST |
| biondo1.723 | biondo | 3 | 1.72 | borsa | only heard | 0 | 6 | m | f | 0.0547945 | 0.109589 | 0.067901235 | POST |
| biondo1.723 | biondo | 3 | 1.72 | braccio | only heard | 0 | 6 | m | f | 0.1209677 | 0.1048387 | 0.02 | POST |
| biondo1.852 | biondo | 2 | 1.85 | bisnonna | only heard | 0 | 6 | m | f | 0.0571428 | 0.1333333 | 0.049019608 | POST |
| biondo1.852 | biondo | 2 | 1.85 | blind | only heard | 0 | 6 | m | f | 0 | 0.1553398 | 0.053763441 | POST |
| biondo1.852 | biondo | 2 | 1.85 | braccio | only heard | 0 | 6 | m | f | 0.1209677 | 0.1048387 | 0.02 | POST |
| biondo1.852 | biondo | 2 | 1.85 | borsa | only heard | 0 | 6 | m | f | 0.0547945 | 0.109589 | 0.067901235 | POST |
| biondo1.863 | biondo | 3 | 1.86 | blind | only seen | 0 | 4 | m | f | 0 | 0.1553398 | 0.053763441 | POST |
| biondo1.863 | biondo | 3 | 1.86 | braccio | only seen | 0 | 4 | m | f | 0.1209677 | 0.1048387 | 0.02 | POST |
| biondo1.863 | biondo | 3 | 1.86 | bigne | 0 | 1 | 4 | m | f | 0 | 0.1190476 | 0.038011696 | POST |
| biondo1.863 | biondo | 3 | 1.86 | blanca | 0 | 0 | 4 | m | f | 0 | 0.0491803 | 0.032921811 | POST |
| biondo1.873 | biondo | 3 | 1.87 | black | only seen | 0 | 2 | m | m | 0 | 0.02 | 0.008130081 | POST |
| biondo1.873 | biondo | 3 | 1.87 | braccio | 0 | 0 | 2 | m | f | 0.1209677 | 0.1048387 | 0.02 | POST |
| biondo1.891 | biondo | 1 | 1.89 | betta | seen and heard | 0 | 2 | m | f | 0.4551724 | 0.0965517 | 0.00408998 | POST |
| biondo1.891 | biondo | 1 | 1.89 | braccio | only heard | 0 | 2 | m | f | 0.1209677 | 0.1048387 | 0.02 | POST |
| biondo1.93 | biondo | 3 | 1.9 | bifida | seen and heard | 1 | 2 | m | f | 0.0135135 | 0.027027 | 0.078014184 | POST |
| biondo1.93 | biondo | 3 | 1.9 | barbie | only heard | 0 | 2 | m | f | 0 | 0 | 0.026666667 | POST |
| biondo1.942 | biondo | 2 | 1.94 | betta | seen and heard | 0 | 7 | m | f | 0.4551724 | 0.0965517 | 0.00408998 | POST |
| biondo1.942 | biondo | 2 | 1.94 | bratz | seen and heard | 0 | 7 | m | f | 0 | 0.0740741 | 0.059322034 | POST |
| biondo1.942 | biondo | 2 | 1.94 | bifida | only heard | 0 | 7 | m | f | 0.0135135 | 0.027027 | 0.078014184 | POST |
| biondo1.942 | biondo | 2 | 1.94 | black | only heard | 0 | 7 | m | m | 0 | 0.02 | 0.008130081 | POST |
| biondo1.942 | biondo | 2 | 1.94 | barbie | only heard | 0 | 7 | m | f | 0 | 0 | 0.026666667 | POST |
| biondo1.942 | biondo | 2 | 1.94 | bisnonna | only heard | 0 | 7 | m | f | 0.0571428 | 0.1333333 | 0.049019608 | POST |
| biondo1.942 | biondo | 2 | 1.94 | blind | only heard | 0 | 7 | m | f | 0 | 0.1553398 | 0.053763441 | POST |
| biondo1.943 | biondo | 3 | 1.94 | bifida | 0 | 0 | 7 | m | f | 0.0135135 | 0.027027 | 0.078014184 | POST |
| biondo1.943 | biondo | 3 | 1.94 | betta | 0 | 0 | 7 | m | f | 0.4551724 | 0.0965517 | 0.00408998 | POST |
| biondo1.943 | biondo | 3 | 1.94 | basic | 0 | 0 | 7 | m | f | 0 | 0.0196078 | 0.053030303 | POST |
| biondo1.943 | biondo | 3 | 1.94 | bratz | 0 | 0 | 7 | m | f | 0 | 0.0740741 | 0.059322034 | POST |
| biondo1.943 | biondo | 3 | 1.94 | bortolo | 0 | 0 | 7 | m | m | 0 | 0.0588235 | 0.032051282 | POST |
| biondo1.943 | biondo | 3 | 1.94 | blanca | 0 | 0 | 7 | m | f | 0 | 0.0491803 | 0.032921811 | POST |
| biondo1.943 | biondo | 3 | 1.94 | bijoux | 0 | 0 | 7 | m | f | 0 | 0.0869565 | 0.061403509 | POST |
| biondo1.982 | biondo | 2 | 1.98 | betta | seen and heard | 0 | 1 | m | f | 0.4551724 | 0.0965517 | 0.00408998 | POST |
| biondo1.983 | biondo | 3 | 1.98 | braccio | 0 | 0 | 1 | m | f | 0.1209677 | 0.1048387 | 0.02 | POST |
| biondo2.023 | biondo | 3 | 2.02 | barbie | only seen | 1 | 16 | m | f | 0 | 0 | 0.026666667 | POST |
| biondo2.023 | biondo | 3 | 2.02 | blanca | only seen | 0 | 16 | m | f | 0 | 0.0491803 | 0.032921811 | POST |
| biondo2.023 | biondo | 3 | 2.02 | bernoccolo | only seen | 0 | 16 | m | m | 0 | 0 | 0.01754386 | POST |
| biondo2.023 | biondo | 3 | 2.02 | bratz | only seen | 0 | 16 | m | f | 0 | 0.0740741 | 0.059322034 | POST |
| biondo2.023 | biondo | 3 | 2.02 | black | only seen | 0 | 16 | m | m | 0 | 0.02 | 0.008130081 | POST |
| biondo2.023 | biondo | 3 | 2.02 | borsa | 0 | 0 | 16 | m | f | 0.0547945 | 0.109589 | 0.067901235 | POST |
| biondo2.023 | biondo | 3 | 2.02 | betta | 0 | 0 | 16 | m | f | 0.4551724 | 0.0965517 | 0.00408998 | POST |
| biondo2.023 | biondo | 3 | 2.02 | braccio | 0 | 0 | 16 | m | f | 0.1209677 | 0.1048387 | 0.02 | POST |
| biondo2.023 | biondo | 3 | 2.02 | blind | 0 | 0 | 16 | m | f | 0 | 0.1553398 | 0.053763441 | POST |
| biondo2.023 | biondo | 3 | 2.02 | bisnonna | 0 | 0 | 16 | m | f | 0.0571428 | 0.1333333 | 0.049019608 | POST |
| biondo2.023 | biondo | 3 | 2.02 | bortolo | 0 | 0 | 16 | m | m | 0 | 0.0588235 | 0.032051282 | POST |
| biondo2.023 | biondo | 3 | 2.02 | belly | 0 | 0 | 16 | m | f | 0 | 0 | 0.024390244 | POST |
| biondo2.023 | biondo | 3 | 2.02 | bigne | 0 | 0 | 16 | m | f | 0 | 0.1190476 | 0.038011696 | POST |
| biondo2.023 | biondo | 3 | 2.02 | biba | 0 | 0 | 16 | m | f | 0 | 0 | 0.011111111 | POST |
| biondo2.041 | biondo | 1 | 2.04 | bigne | only seen | 0 | 4 | m | f | 0 | 0.1190476 | 0.038011696 | POST |
| biondo2.041 | biondo | 1 | 2.04 | blanca | only seen | 1 | 4 | m | f | 0 | 0.0491803 | 0.032921811 | POST |
| biondo2.041 | biondo | 1 | 2.04 | bisnonna | only seen | 0 | 4 | m | f | 0.0571428 | 0.1333333 | 0.049019608 | POST |
| biondo2.041 | biondo | 1 | 2.04 | blind | 0 | 0 | 4 | m | f | 0 | 0.1553398 | 0.053763441 | POST |
| biondo2.073 | biondo | 3 | 2.07 | barbie | only seen | 0 | 8 | m | f | 0 | 0 | 0.026666667 | POST |
| biondo2.073 | biondo | 3 | 2.07 | black | only seen | 0 | 8 | m | m | 0 | 0.02 | 0.008130081 | POST |
| biondo2.073 | biondo | 3 | 2.07 | braccio | only seen | 0 | 8 | m | f | 0.1209677 | 0.1048387 | 0.02 | POST |
| biondo2.073 | biondo | 3 | 2.07 | betta | only seen | 0 | 8 | m | f | 0.4551724 | 0.0965517 | 0.00408998 | POST |
| biondo2.073 | biondo | 3 | 2.07 | bortolo | only seen | 0 | 8 | m | m | 0 | 0.0588235 | 0.032051282 | POST |
| biondo2.073 | biondo | 3 | 2.07 | blind | 0 | 0 | 8 | m | f | 0 | 0.1553398 | 0.053763441 | POST |
| biondo2.073 | biondo | 3 | 2.07 | bisnonna | 0 | 0 | 8 | m | f | 0.0571428 | 0.1333333 | 0.049019608 | POST |
| biondo2.073 | biondo | 3 | 2.07 | bernoccolo | 0 | 0 | 8 | m | m | 0 | 0 | 0.01754386 | POST |
| biondo2.131 | biondo | 1 | 2.13 | braccio | seen and heard | 0 | 5 | m | f | 0.1209677 | 0.1048387 | 0.02 | POST |
| biondo2.131 | biondo | 1 | 2.13 | borsa | seen and heard | 1 | 5 | m | f | 0.0547945 | 0.109589 | 0.067901235 | POST |
| biondo2.131 | biondo | 1 | 2.13 | blind | 0 | 0 | 5 | m | f | 0 | 0.1553398 | 0.053763441 | POST |
| biondo2.131 | biondo | 1 | 2.13 | bisnonna | 0 | 0 | 5 | m | f | 0.0571428 | 0.1333333 | 0.049019608 | POST |
| biondo2.131 | biondo | 1 | 2.13 | bratz | 0 | 0 | 5 | m | f | 0 | 0.0740741 | 0.059322034 | POST |
| biondo2.23 | biondo | 3 | 2.2 | black | only seen | 0 | 5 | m | m | 0 | 0.02 | 0.008130081 | POST |
| biondo2.23 | biondo | 3 | 2.2 | betta | 0 | 0 | 5 | m | f | 0.4551724 | 0.0965517 | 0.00408998 | POST |
| biondo2.23 | biondo | 3 | 2.2 | borsa | 0 | 0 | 5 | m | f | 0.0547945 | 0.109589 | 0.067901235 | POST |
| biondo2.23 | biondo | 3 | 2.2 | bernoccolo | 0 | 0 | 5 | m | m | 0 | 0 | 0.01754386 | POST |
| biondo2.23 | biondo | 3 | 2.2 | bortolo | 0 | 0 | 5 | m | m | 0 | 0.0588235 | 0.032051282 | POST |
| biondo2.2081 | biondo | 1 | 2.208 | betta | seen and heard | 0 | 4 | m | f | 0.4551724 | 0.0965517 | 0.00408998 | POST |
| biondo2.2081 | biondo | 1 | 2.208 | blanca | only heard | 0 | 4 | m | f | 0 | 0.0491803 | 0.032921811 | POST |
| biondo2.2081 | biondo | 1 | 2.208 | bratz | only heard | 1 | 4 | m | f | 0 | 0.0740741 | 0.059322034 | POST |
| biondo2.233 | biondo | 3 | 2.23 | bifida | 0 | 0 | 5 | m | f | 0.0135135 | 0.027027 | 0.078014184 | POST |
| biondo2.233 | biondo | 3 | 2.23 | bisnonna | 0 | 0 | 5 | m | f | 0.0571428 | 0.1333333 | 0.049019608 | POST |
| biondo2.233 | biondo | 3 | 2.23 | bigne | 0 | 0 | 5 | m | f | 0 | 0.1190476 | 0.038011696 | POST |
| biondo2.233 | biondo | 3 | 2.23 | blind | 0 | 0 | 5 | m | f | 0 | 0.1553398 | 0.053763441 | POST |
| biondo2.233 | biondo | 3 | 2.23 | braccio | 0 | 0 | 5 | m | f | 0.1209677 | 0.1048387 | 0.02 | POST |
| biondo2.282 | biondo | 2 | 2.28 | black | seen and heard | 0 | 12 | m | m | 0 | 0.02 | 0.008130081 | POST |
| biondo2.282 | biondo | 2 | 2.28 | bifida | seen and heard | 0 | 12 | m | f | 0.0135135 | 0.027027 | 0.078014184 | POST |
| biondo2.282 | biondo | 2 | 2.28 | bigne | seen and heard | 0 | 12 | m | f | 0 | 0.1190476 | 0.038011696 | POST |
| biondo2.282 | biondo | 2 | 2.28 | bernoccolo | only heard | 0 | 12 | m | m | 0 | 0 | 0.01754386 | POST |
| biondo2.282 | biondo | 2 | 2.28 | braccio | only heard | 0 | 12 | m | f | 0.1209677 | 0.1048387 | 0.02 | POST |
| biondo2.282 | biondo | 2 | 2.28 | blind | only heard | 0 | 12 | m | f | 0 | 0.1553398 | 0.053763441 | POST |
| biondo2.282 | biondo | 2 | 2.28 | bisnonna | only heard | 0 | 12 | m | f | 0.0571428 | 0.1333333 | 0.049019608 | POST |
| biondo2.282 | biondo | 2 | 2.28 | barbie | only heard | 0 | 12 | m | f | 0 | 0 | 0.026666667 | POST |
| biondo2.282 | biondo | 2 | 2.28 | basic | only heard | 0 | 12 | m | f | 0 | 0.0196078 | 0.053030303 | POST |
| biondo2.282 | biondo | 2 | 2.28 | borsa | only heard | 0 | 12 | m | f | 0.0547945 | 0.109589 | 0.067901235 | POST |
| biondo2.282 | biondo | 2 | 2.28 | betta | only heard | 0 | 12 | m | f | 0.4551724 | 0.0965517 | 0.00408998 | POST |
| biondo2.283 | biondo | 3 | 2.28 | basic | only seen | 0 | 13 | m | f | 0 | 0.0196078 | 0.053030303 | POST |
| biondo2.283 | biondo | 3 | 2.28 | barbie | only seen | 0 | 13 | m | f | 0 | 0 | 0.026666667 | POST |
| biondo2.283 | biondo | 3 | 2.28 | bortolo | only seen | 0 | 13 | m | m | 0 | 0.0588235 | 0.032051282 | POST |
| biondo2.283 | biondo | 3 | 2.28 | bernoccolo | only seen | 0 | 13 | m | m | 0 | 0 | 0.01754386 | POST |
| biondo2.283 | biondo | 3 | 2.28 | bratz | only seen | 0 | 13 | m | f | 0 | 0.0740741 | 0.059322034 | POST |
| biondo2.283 | biondo | 3 | 2.28 | black | only seen | 0 | 13 | m | m | 0 | 0.02 | 0.008130081 | POST |
| biondo2.283 | biondo | 3 | 2.28 | borsa | 0 | 0 | 13 | m | f | 0.0547945 | 0.109589 | 0.067901235 | POST |
| biondo2.283 | biondo | 3 | 2.28 | betta | 0 | 0 | 13 | m | f | 0.4551724 | 0.0965517 | 0.00408998 | POST |
| biondo2.283 | biondo | 3 | 2.28 | blind | 0 | 0 | 13 | m | f | 0 | 0.1553398 | 0.053763441 | POST |
| biondo2.283 | biondo | 3 | 2.28 | bisnonna | 0 | 0 | 13 | m | f | 0.0571428 | 0.1333333 | 0.049019608 | POST |
| biondo2.283 | biondo | 3 | 2.28 | braccio | 0 | 0 | 13 | m | f | 0.1209677 | 0.1048387 | 0.02 | POST |
| biondo2.283 | biondo | 3 | 2.28 | belly | 0 | 0 | 13 | m | f | 0 | 0 | 0.024390244 | POST |
| biondo2.283 | biondo | 3 | 2.28 | bifida | 0 | 0 | 13 | m | f | 0.0135135 | 0.027027 | 0.078014184 | POST |
| biondo2.312 | biondo | 2 | 2.31 | betta | seen and heard | 0 | 9 | m | f | 0.4551724 | 0.0965517 | 0.00408998 | POST |
| biondo2.312 | biondo | 2 | 2.31 | bernoccolo | seen and heard | 1 | 9 | m | m | 0 | 0 | 0.01754386 | POST |
| biondo2.312 | biondo | 2 | 2.31 | black | seen and heard | 0 | 9 | m | m | 0 | 0.02 | 0.008130081 | POST |
| biondo2.312 | biondo | 2 | 2.31 | borsa | seen and heard | 0 | 9 | m | f | 0.0547945 | 0.109589 | 0.067901235 | POST |
| biondo2.312 | biondo | 2 | 2.31 | barbie | seen and heard | 0 | 9 | m | f | 0 | 0 | 0.026666667 | POST |
| biondo2.312 | biondo | 2 | 2.31 | bisnonna | only heard | 0 | 9 | m | f | 0.0571428 | 0.1333333 | 0.049019608 | POST |
| biondo2.312 | biondo | 2 | 2.31 | blind | only heard | 0 | 9 | m | f | 0 | 0.1553398 | 0.053763441 | POST |
| biondo2.312 | biondo | 2 | 2.31 | braccio | only heard | 0 | 9 | m | f | 0.1209677 | 0.1048387 | 0.02 | POST |
| biondo2.312 | biondo | 2 | 2.31 | bortolo | only heard | 0 | 9 | m | m | 0 | 0.0588235 | 0.032051282 | POST |
| biondo2.481 | biondo | 1 | 2.48 | bisnonna | only seen | 0 | 8 | m | f | 0.0571428 | 0.1333333 | 0.049019608 | POST |
| biondo2.481 | biondo | 1 | 2.48 | bigne | only seen | 0 | 8 | m | f | 0 | 0.1190476 | 0.038011696 | POST |
| biondo2.481 | biondo | 1 | 2.48 | blanca | only seen | 0 | 8 | m | f | 0 | 0.0491803 | 0.032921811 | POST |
| biondo2.481 | biondo | 1 | 2.48 | basic | 0 | 0 | 8 | m | f | 0 | 0.0196078 | 0.053030303 | POST |
| biondo2.481 | biondo | 1 | 2.48 | blind | 0 | 0 | 8 | m | f | 0 | 0.1553398 | 0.053763441 | POST |
| biondo2.481 | biondo | 1 | 2.48 | bijoux | 0 | 0 | 8 | m | f | 0 | 0.0869565 | 0.061403509 | POST |
| biondo2.481 | biondo | 1 | 2.48 | bernoccolo | 0 | 0 | 8 | m | m | 0 | 0 | 0.01754386 | POST |
| biondo2.481 | biondo | 1 | 2.48 | betta | 0 | 0 | 8 | m | f | 0.4551724 | 0.0965517 | 0.00408998 | POST |
| biondo2.511 | biondo | 1 | 2.51 | borsa | 0 | 0 | 13 | m | f | 0.0547945 | 0.109589 | 0.067901235 | POST |
| biondo2.511 | biondo | 1 | 2.51 | betta | 0 | 0 | 13 | m | f | 0.4551724 | 0.0965517 | 0.00408998 | POST |
| biondo2.511 | biondo | 1 | 2.51 | blind | 0 | 0 | 13 | m | f | 0 | 0.1553398 | 0.053763441 | POST |
| biondo2.511 | biondo | 1 | 2.51 | black | 0 | 0 | 13 | m | m | 0 | 0.02 | 0.008130081 | POST |
| biondo2.511 | biondo | 1 | 2.51 | bijoux | 0 | 0 | 13 | m | f | 0 | 0.0869565 | 0.061403509 | POST |
| biondo2.511 | biondo | 1 | 2.51 | bortolo | 0 | 0 | 13 | m | m | 0 | 0.0588235 | 0.032051282 | POST |
| biondo2.511 | biondo | 1 | 2.51 | bernoccolo | 0 | 0 | 13 | m | m | 0 | 0 | 0.01754386 | POST |
| biondo2.511 | biondo | 1 | 2.51 | braccio | 0 | 0 | 13 | m | f | 0.1209677 | 0.1048387 | 0.02 | POST |
| biondo2.511 | biondo | 1 | 2.51 | bisnonna | 0 | 0 | 13 | m | f | 0.0571428 | 0.1333333 | 0.049019608 | POST |
| biondo2.511 | biondo | 1 | 2.51 | blanca | 0 | 0 | 13 | m | f | 0 | 0.0491803 | 0.032921811 | POST |
| biondo2.511 | biondo | 1 | 2.51 | belly | 0 | 0 | 13 | m | f | 0 | 0 | 0.024390244 | POST |
| biondo2.511 | biondo | 1 | 2.51 | bratz | 0 | 0 | 13 | m | f | 0 | 0.0740741 | 0.059322034 | POST |
| biondo2.511 | biondo | 1 | 2.51 | bigne | 0 | 0 | 13 | m | f | 0 | 0.1190476 | 0.038011696 | POST |
| biondo2.631 | biondo | 1 | 2.63 | braccio | 0 | 0 | 1 | m | f | 0.1209677 | 0.1048387 | 0.02 | POST |
| biondo2.933 | biondo | 3 | 2.93 | borsa | only heard | 0 | 13 | m | f | 0.0547945 | 0.109589 | 0.067901235 | POST |
| biondo2.933 | biondo | 3 | 2.93 | betta | only heard | 0 | 13 | m | f | 0.4551724 | 0.0965517 | 0.00408998 | POST |
| biondo2.933 | biondo | 3 | 2.93 | blind | only heard | 0 | 13 | m | f | 0 | 0.1553398 | 0.053763441 | POST |
| biondo2.933 | biondo | 3 | 2.93 | black | only heard | 0 | 13 | m | m | 0 | 0.02 | 0.008130081 | PRE |
| biondo2.933 | biondo | 3 | 2.93 | bijoux | only heard | 0 | 13 | m | f | 0 | 0.0869565 | 0.061403509 | PRE |
| biondo2.933 | biondo | 3 | 2.93 | bortolo | only heard | 0 | 13 | m | m | 0 | 0.0588235 | 0.032051282 | PRE |
| biondo2.933 | biondo | 3 | 2.93 | bernoccolo | only heard | 0 | 13 | m | m | 0 | 0 | 0.01754386 | PRE |
| biondo2.933 | biondo | 3 | 2.93 | braccio | only heard | 0 | 13 | m | f | 0.1209677 | 0.1048387 | 0.02 | PRE |
| biondo2.933 | biondo | 3 | 2.93 | blanca | only heard | 0 | 13 | m | f | 0 | 0.0491803 | 0.032921811 | PRE |
| biondo2.933 | biondo | 3 | 2.93 | belly | only heard | 0 | 13 | m | f | 0 | 0 | 0.024390244 | PRE |
| biondo2.933 | biondo | 3 | 2.93 | bratz | only heard | 0 | 13 | m | f | 0 | 0.0740741 | 0.059322034 | PRE |
| biondo2.933 | biondo | 3 | 2.93 | bigne | only heard | 0 | 13 | m | f | 0 | 0.1190476 | 0.038011696 | PRE |
| biondo3.053 | biondo | 3 | 3.05 | basic | only seen | 0 | 14 | m | f | 0 | 0.0196078 | 0.053030303 | PRE |
| biondo3.053 | biondo | 3 | 3.05 | barbie | only seen | 0 | 14 | m | f | 0 | 0 | 0.026666667 | PRE |
| biondo3.053 | biondo | 3 | 3.05 | black | only seen | 0 | 14 | m | m | 0 | 0.02 | 0.008130081 | PRE |
| biondo3.053 | biondo | 3 | 3.05 | belly | only seen | 0 | 14 | m | f | 0 | 0 | 0.024390244 | PRE |
| biondo3.053 | biondo | 3 | 3.05 | bisnonna | 0 | 0 | 14 | m | f | 0.0571428 | 0.1333333 | 0.049019608 | PRE |
| biondo3.053 | biondo | 3 | 3.05 | blind | 0 | 0 | 14 | m | f | 0 | 0.1553398 | 0.053763441 | PRE |
| biondo3.053 | biondo | 3 | 3.05 | braccio | 0 | 0 | 14 | m | f | 0.1209677 | 0.1048387 | 0.02 | PRE |
| biondo3.053 | biondo | 3 | 3.05 | blanca | 0 | 0 | 14 | m | f | 0 | 0.0491803 | 0.032921811 | PRE |
| biondo3.053 | biondo | 3 | 3.05 | bernoccolo | 0 | 0 | 14 | m | m | 0 | 0 | 0.01754386 | PRE |
| biondo3.053 | biondo | 3 | 3.05 | bortolo | 0 | 0 | 14 | m | m | 0 | 0.0588235 | 0.032051282 | PRE |
| biondo3.053 | biondo | 3 | 3.05 | biba | 0 | 0 | 14 | m | f | 0 | 0 | 0.011111111 | PRE |
| biondo3.053 | biondo | 3 | 3.05 | borsa | 0 | 0 | 14 | m | f | 0.0547945 | 0.109589 | 0.067901235 | PRE |
| biondo3.313 | biondo | 3 | 3.31 | borsa | 0 | 0 | 12 | m | f | 0.0547945 | 0.109589 | 0.067901235 | PRE |
| biondo3.313 | biondo | 3 | 3.31 | betta | 0 | 0 | 12 | m | f | 0.4551724 | 0.0965517 | 0.00408998 | PRE |
| biondo3.313 | biondo | 3 | 3.31 | blind | 0 | 0 | 12 | m | f | 0 | 0.1553398 | 0.053763441 | PRE |
| biondo3.313 | biondo | 3 | 3.31 | black | 0 | 0 | 12 | m | m | 0 | 0.02 | 0.008130081 | PRE |
| biondo3.313 | biondo | 3 | 3.31 | bijoux | 0 | 0 | 12 | m | f | 0 | 0.0869565 | 0.061403509 | PRE |
| biondo3.313 | biondo | 3 | 3.31 | bortolo | 0 | 0 | 12 | m | m | 0 | 0.0588235 | 0.032051282 | PRE |
| biondo3.313 | biondo | 3 | 3.31 | bernoccolo | 0 | 0 | 12 | m | m | 0 | 0 | 0.01754386 | PRE |
| biondo3.313 | biondo | 3 | 3.31 | bigne | 0 | 0 | 12 | m | f | 0 | 0.1190476 | 0.038011696 | PRE |
| biondo3.313 | biondo | 3 | 3.31 | braccio | 0 | 0 | 12 | m | f | 0.1209677 | 0.1048387 | 0.02 | PRE |
| biondo3.313 | biondo | 3 | 3.31 | bisnonna | 0 | 0 | 12 | m | f | 0.0571428 | 0.1333333 | 0.049019608 | PRE |
| biondo3.313 | biondo | 3 | 3.31 | blanca | 0 | 0 | 12 | m | f | 0 | 0.0491803 | 0.032921811 | PRE |
| biondo3.313 | biondo | 3 | 3.31 | bifida | 0 | 1 | 12 | m | f | 0.0135135 | 0.027027 | 0.078014184 | PRE |
| bisnonna1.54* | bisnonna | * | 1.54 | bigne | 0 | 0 | 3 | f | f | 0.1527778 | 0.1388889 | 0.038011696 | POST |
| bisnonna1.54* | bisnonna | * | 1.54 | biondo | 0 | 0 | 3 | f | m | 0.0517428 | 0.1333333 | 0.14021164 | POST |
| bisnonna1.761 | bisnonna | 1 | 1.76 | borsa | 0 | 0 | 5 | f | f | 0.155555556 | 0.044444444 | 0.067901235 | POST |
| bisnonna1.761 | bisnonna | 1 | 1.76 | braccio | 0 | 0 | 5 | f | f | 0.027027027 | 0.040540541 | 0.02 | POST |
| bisnonna1.761 | bisnonna | 1 | 1.76 | bigne | 0 | 0 | 5 | f | f | 0.1527778 | 0.1388889 | 0.038011696 | POST |
| bisnonna1.761 | bisnonna | 1 | 1.76 | blind | 0 | 0 | 5 | f | f | 0.19047619 | 0.07142851 | 0.053763441 | POST |
| bisnonna1.761 | bisnonna | 1 | 1.76 | bifida | 0 | 0 | 5 | f | f | 0 | 0.106383 | 0.078014184 | POST |
| bisnonna1.771 | bisnonna | 1 | 1.77 | borsa | seen and heard | 0 | 2 | f | f | 0.155555556 | 0.044444444 | 0.067901235 | POST |
| bisnonna1.771 | bisnonna | 1 | 1.77 | blind | only heard | 0 | 2 | f | f | 0.19047619 | 0.07142851 | 0.053763441 | POST |
| bisnonna1.841 | bisnonna | 1 | 1.84 | borsa | only seen | 0 | 1 | f | f | 0.155555556 | 0.044444444 | 0.067901235 | POST |
| bisnonna1.861 | bisnonna | 1 | 1.86 | bigne | only seen | 1 | 2 | f | f | 0.1527778 | 0.1388889 | 0.038011696 | POST |
| bisnonna1.861 | bisnonna | 1 | 1.86 | basic | 0 | 0 | 2 | f | f | 0.01960784 | 0.0980392 | 0.053030303 | POST |
| bisnonna1.993 | bisnonna | 3 | 1.99 | basic | only seen | 0 | 2 | f | f | 0.01960784 | 0.0980392 | 0.053030303 | POST |
| bisnonna1.993 | bisnonna | 3 | 1.99 | bigne | only seen | 0 | 2 | f | f | 0.1527778 | 0.1388889 | 0.038011696 | POST |
| bisnonna2.221 | bisnonna | 1 | 2.22 | blind | only seen | 0 | 1 | f | f | 0.19047619 | 0.07142851 | 0.053763441 | POST |
| bisnonna2.363 | bisnonna | 3 | 2.36 | bigne | only seen | 0 | 2 | f | f | 0.1527778 | 0.1388889 | 0.038011696 | POST |
| bisnonna2.363 | bisnonna | 3 | 2.36 | basic | 0 | 0 | 2 | f | f | 0.01960784 | 0.0980392 | 0.053030303 | POST |
| bisnonna2.421 | bisnonna | 1 | 2.42 | blind | only seen | 0 | 2 | f | f | 0.19047619 | 0.07142851 | 0.053763441 | POST |
| bisnonna2.421 | bisnonna | 1 | 2.42 | bigne | 0 | 0 | 2 | f | f | 0.1527778 | 0.1388889 | 0.038011696 | POST |
| bisnonna2.631 | bisnonna | 1 | 2.63 | borsa | only seen | 0 | 1 | f | f | 0.155555556 | 0.044444444 | 0.067901235 | POST |
| bisnonna2.923 | bisnonna | 3 | 2.92 | borsa | 0 | 0 | 12 | f | f | 0.155555556 | 0.044444444 | 0.067901235 | POST |
| bisnonna2.923 | bisnonna | 3 | 2.92 | betta | 0 | 0 | 12 | f | f | 0 | 0.078125 | 0.00408998 | POST |
| bisnonna2.923 | bisnonna | 3 | 2.92 | blind | 0 | 0 | 12 | f | f | 0.19047619 | 0.07142851 | 0.053763441 | POST |
| bisnonna2.923 | bisnonna | 3 | 2.92 | black | 0 | 0 | 12 | f | m | 0 | 0.066666667 | 0.008130081 | POST |
| bisnonna2.923 | bisnonna | 3 | 2.92 | bijoux | 0 | 0 | 12 | f | f | 0 | 0.05555556 | 0.061403509 | POST |
| bisnonna2.923 | bisnonna | 3 | 2.92 | bortolo | 0 | 0 | 12 | f | m | 0 | 0.12 | 0.032051282 | POST |
| bisnonna2.923 | bisnonna | 3 | 2.92 | bernoccolo | 0 | 0 | 12 | f | m | 0 | 0 | 0.01754386 | POST |
| bisnonna2.923 | bisnonna | 3 | 2.92 | braccio | 0 | 0 | 12 | f | f | 0.027027027 | 0.040540541 | 0.02 | POST |
| bisnonna2.923 | bisnonna | 3 | 2.92 | biondo | 0 | 0 | 12 | f | m | 0.0517428 | 0.1333333 | 0.14021164 | POST |
| bisnonna2.923 | bisnonna | 3 | 2.92 | blanca | 0 | 0 | 12 | f | f | 0 | 0.25714285 | 0.032921811 | POST |
| bisnonna2.923 | bisnonna | 3 | 2.92 | belly | 0 | 0 | 12 | f | f | 0 | 0.285714286 | 0.024390244 | POST |
| bisnonna2.923 | bisnonna | 3 | 2.92 | bigne | 0 | 0 | 12 | f | f | 0.1527778 | 0.1388889 | 0.038011696 | POST |
| bisnonna3.061 | bisnonna | 1 | 3.06 | bigne | seen and heard | 0 | 1 | f | f | 0.1527778 | 0.1388889 | 0.038011696 | POST |
| bisnonna3.071 | bisnonna | 1 | 3.07 | borsa | only seen | 0 | 1 | f | f | 0.155555556 | 0.044444444 | 0.067901235 | POST |
| black1.451 | black | 1 | 1.45 | bijoux | only seen | 1 | 1 | m | f | 0.22222222 | 0.1666667 | 0.061403509 | POST |
| black1.451 | black | 1 | 1.45 | bijoux | 0 | 1 | 1 | m | f | 0.22222222 | 0.1666667 | 0.061403509 | POST |
| black1.771 | black | 1 | 1.77 | bratz | only seen | 0 | 5 | m | f | 0.34146341 | 0.2439024 | 0.059322034 | POST |
| black1.771 | black | 1 | 1.77 | borsa | 0 | 0 | 5 | m | f | 0.14285714 | 0.22857142 | 0.067901235 | POST |
| black1.771 | black | 1 | 1.77 | bortolo | 0 | 0 | 5 | m | m | 0 | 0.066666667 | 0.032051282 | POST |
| black1.771 | black | 1 | 1.77 | betta | 0 | 0 | 5 | m | f | 0.047619 | 0.0238095 | 0.00408998 | POST |
| black1.771 | black | 1 | 1.77 | barbie | 0 | 0 | 5 | m | f | 0.04761904 | 0.113636364 | 0.026666667 | POST |
| black2.11 | black | 1 | 2.1 | bratz | 0 | 0 | 2 | m | f | 0.34146341 | 0.2439024 | 0.059322034 | POST |
| black2.11 | black | 1 | 2.1 | bifida | 0 | 0 | 2 | m | f | 0.0344828 | 0.1724138 | 0.078014184 | POST |
| black2.981 | black | 1 | 2.98 | bijoux | only heard | 0 | 8 | m | f | 0.22222222 | 0.1666667 | 0.061403509 | POST |
| black2.981 | black | 1 | 2.98 | blind | 0 | 1 | 8 | m | f | 0 | 0.1875 | 0.053763441 | POST |
| black2.981 | black | 1 | 2.98 | bisnonna | 0 | 0 | 8 | m | f | 0 | 0.06666667 | 0.049019608 | POST |
| black2.981 | black | 1 | 2.98 | basic | 0 | 0 | 8 | m | f | 0 | 0.0869565 | 0.053030303 | POST |
| black2.981 | black | 1 | 2.98 | betta | 0 | 0 | 8 | m | f | 0.047619 | 0.0238095 | 0.00408998 | POST |
| black2.981 | black | 1 | 2.98 | biondo | 0 | 0 | 8 | m | m | 0 | 0.02 | 0.14021164 | POST |
| black2.981 | black | 1 | 2.98 | braccio | 0 | 0 | 8 | m | f | 0.02380951 | 0.23809523 | 0.02 | POST |
| black2.981 | black | 1 | 2.98 | bigne | 0 | 0 | 8 | m | f | 0 | 0 | 0.038011696 | POST |
| blanca1.081 | blanca | 1 | 1.08 | bisnonna | only seen | 0 | 7 | f | f | 0 | 0.25714285 | 0.049019608 | POST |
| blanca1.081 | blanca | 1 | 1.08 | blind | only seen | 0 | 7 | f | f | 0.345454545 | 0.1272727 | 0.053763441 | POST |
| blanca1.081 | blanca | 1 | 1.08 | braccio | 0 | 0 | 7 | f | f | 0 | 0.14583333 | 0.02 | POST |
| blanca1.081 | blanca | 1 | 1.08 | betta | 0 | 0 | 7 | f | f | 0 | 0.1764706 | 0.00408998 | POST |
| blanca1.081 | blanca | 1 | 1.08 | borsa | 0 | 0 | 7 | f | f | 0.11111111 | 0.08333333 | 0.067901235 | POST |
| blanca1.081 | blanca | 1 | 1.08 | bigne | 0 | 0 | 7 | f | f | 0 | 0.1914894 | 0.038011696 | POST |
| blanca1.081 | blanca | 1 | 1.08 | bratz | 0 | 0 | 7 | f | f | 0.08163269 | 0.12244897 | 0.059322034 | POST |
| blanca1.2271 | blanca | 1 | 1.227 | blind | only seen | 0 | 7 | f | f | 0.345454545 | 0.1272727 | 0.053763441 | POST |
| blanca1.2271 | blanca | 1 | 1.227 | borsa | only seen | 0 | 7 | f | f | 0.11111111 | 0.08333333 | 0.067901235 | POST |
| blanca1.2271 | blanca | 1 | 1.227 | black | 0 | 0 | 7 | f | m | 0 | 0 | 0.008130081 | POST |
| blanca1.2271 | blanca | 1 | 1.227 | bratz | 0 | 0 | 7 | f | f | 0.08163269 | 0.12244897 | 0.059322034 | POST |
| blanca1.2271 | blanca | 1 | 1.227 | biondo | 0 | 0 | 7 | f | m | 0 | 0.0491803 | 0.14021164 | POST |
| blanca1.2271 | blanca | 1 | 1.227 | giangi | 0 | 0 | 7 | f | m | 0 | 0 | 0.215686275 | POST |
| blanca1.2271 | blanca | 1 | 1.227 | ghirlanda | 0 | 0 | 7 | f | f | 0 | 0 | 0.023148148 | POST |
| blanca1.351 | blanca | 1 | 1.35 | bifida | only seen | 1 | 3 | f | f | 0.1538462 | 0.0512821 | 0.078014184 | POST |
| blanca1.351 | blanca | 1 | 1.35 | barbie | 0 | 0 | 3 | f | f | 0.08 | 0.04 | 0.026666667 | POST |
| blanca1.351 | blanca | 1 | 1.35 | bratz | 0 | 0 | 3 | f | f | 0.08163269 | 0.12244897 | 0.059322034 | POST |
| blanca1.592* | blanca | * | 1.592 | borsa | 0 | 0 | 3 | f | f | 0.11111111 | 0.08333333 | 0.067901235 | POST |
| blanca1.693 | blanca | 3 | 1.69 | bigne | only seen | 0 | 2 | f | f | 0 | 0.1914894 | 0.038011696 | POST |
| blanca1.693 | blanca | 3 | 1.69 | blind | 0 | 0 | 2 | f | f | 0.345454545 | 0.1272727 | 0.053763441 | POST |
| blanca1.791 | blanca | 1 | 1.79 | blind | only seen | 0 | 3 | f | f | 0.345454545 | 0.1272727 | 0.053763441 | POST |
| blanca1.791 | blanca | 1 | 1.79 | belly | 0 | 0 | 3 | f | f | 0 | 0.05263157 | 0.024390244 | POST |
| blanca1.791 | blanca | 1 | 1.79 | bratz | 0 | 0 | 3 | f | f | 0.08163269 | 0.12244897 | 0.059322034 | POST |
| blanca1.911 | blanca | 1 | 1.91 | black | only seen | 0 | 15 | f | m | 0 | 0 | 0.008130081 | POST |
| blanca1.911 | blanca | 1 | 1.91 | bifida | only seen | 0 | 15 | f | f | 0.1538462 | 0.0512821 | 0.078014184 | POST |
| blanca1.911 | blanca | 1 | 1.91 | bigne | only seen | 0 | 15 | f | f | 0 | 0.1914894 | 0.038011696 | POST |
| blanca1.911 | blanca | 1 | 1.91 | biba | only seen | 0 | 15 | f | f | 0 | 0 | 0.011111111 | POST |
| blanca1.911 | blanca | 1 | 1.91 | bernoccolo | only seen | 1 | 15 | f | m | 0 | 0 | 0.01754386 | POST |
| blanca1.911 | blanca | 1 | 1.91 | bortolo | only seen | 0 | 15 | f | m | 0 | 0 | 0.032051282 | POST |
| blanca1.911 | blanca | 1 | 1.91 | bratz | only seen | 1 | 15 | f | f | 0.08163269 | 0.12244897 | 0.059322034 | POST |
| blanca1.911 | blanca | 1 | 1.91 | belly | 0 | 0 | 15 | f | f | 0 | 0.05263157 | 0.024390244 | POST |
| blanca1.911 | blanca | 1 | 1.91 | barbie | 0 | 0 | 15 | f | f | 0.08 | 0.04 | 0.026666667 | POST |
| blanca1.911 | blanca | 1 | 1.91 | basic | 0 | 0 | 15 | f | f | 0.04761904 | 0.14285714 | 0.053030303 | POST |
| blanca1.911 | blanca | 1 | 1.91 | borsa | 0 | 0 | 15 | f | f | 0.11111111 | 0.08333333 | 0.067901235 | POST |
| blanca1.911 | blanca | 1 | 1.91 | betta | 0 | 0 | 15 | f | f | 0 | 0.1764706 | 0.00408998 | POST |
| blanca1.911 | blanca | 1 | 1.91 | bisnonna | 0 | 0 | 15 | f | f | 0 | 0.25714285 | 0.049019608 | POST |
| blanca1.911 | blanca | 1 | 1.91 | braccio | 0 | 0 | 15 | f | f | 0 | 0.14583333 | 0.02 | POST |
| blanca1.911 | blanca | 1 | 1.91 | blind | 0 | 0 | 15 | f | f | 0.345454545 | 0.1272727 | 0.053763441 | POST |
| blanca1.961 | blanca | 1 | 1.96 | braccio | only seen | 0 | 7 | f | f | 0 | 0.14583333 | 0.02 | POST |
| blanca1.961 | blanca | 1 | 1.96 | bratz | 0 | 0 | 7 | f | f | 0.08163269 | 0.12244897 | 0.059322034 | POST |
| blanca1.961 | blanca | 1 | 1.96 | blind | 0 | 0 | 7 | f | f | 0.345454545 | 0.1272727 | 0.053763441 | POST |
| blanca1.961 | blanca | 1 | 1.96 | biondo | 0 | 1 | 7 | f | m | 0 | 0.0491803 | 0.14021164 | POST |
| blanca1.961 | blanca | 1 | 1.96 | betta | 0 | 0 | 7 | f | f | 0 | 0.1764706 | 0.00408998 | POST |
| blanca1.961 | blanca | 1 | 1.96 | borsa | 0 | 0 | 7 | f | f | 0.11111111 | 0.08333333 | 0.067901235 | POST |
| blanca1.961 | blanca | 1 | 1.96 | bigne | 0 | 0 | 7 | f | f | 0 | 0.1914894 | 0.038011696 | POST |
| blanca2.071 | blanca | 1 | 2.07 | barbie | only seen | 0 | 6 | f | f | 0.08 | 0.04 | 0.026666667 | POST |
| blanca2.071 | blanca | 1 | 2.07 | betta | only seen | 0 | 6 | f | f | 0 | 0.1764706 | 0.00408998 | POST |
| blanca2.071 | blanca | 1 | 2.07 | blind | 0 | 1 | 6 | f | f | 0.345454545 | 0.1272727 | 0.053763441 | POST |
| blanca2.071 | blanca | 1 | 2.07 | biondo | 0 | 1 | 6 | f | m | 0 | 0.0491803 | 0.14021164 | POST |
| blanca2.071 | blanca | 1 | 2.07 | bigne | 0 | 0 | 6 | f | f | 0 | 0.1914894 | 0.038011696 | POST |
| blanca2.071 | blanca | 1 | 2.07 | bratz | 0 | 0 | 6 | f | f | 0.08163269 | 0.12244897 | 0.059322034 | PRE |
| blanca2.451 | blanca | 1 | 2.45 | blind | seen and heard | 0 | 3 | f | f | 0.345454545 | 0.1272727 | 0.053763441 | PRE |
| blanca2.451 | blanca | 1 | 2.45 | bifida | only heard | 0 | 3 | f | f | 0.1538462 | 0.0512821 | 0.078014184 | PRE |
| blanca2.451 | blanca | 1 | 2.45 | bigne | only heard | 0 | 3 | f | f | 0 | 0.1914894 | 0.038011696 | PRE |
| blanca4.571 | blanca | 1 | 4.57 | bigne | only seen | 0 | 10 | f | f | 0 | 0.1914894 | 0.038011696 | PRE |
| blanca4.571 | blanca | 1 | 4.57 | bijoux | only seen | 1 | 10 | f | f | 0 | 0 | 0.061403509 | PRE |
| blanca4.571 | blanca | 1 | 4.57 | biba | only seen | 0 | 10 | f | f | 0 | 0 | 0.011111111 | PRE |
| blanca4.571 | blanca | 1 | 4.57 | bisnonna | 0 | 0 | 10 | f | f | 0 | 0.25714285 | 0.049019608 | PRE |
| blanca4.571 | blanca | 1 | 4.57 | basic | 0 | 0 | 10 | f | f | 0.04761904 | 0.14285714 | 0.053030303 | PRE |
| blanca4.571 | blanca | 1 | 4.57 | blind | 0 | 0 | 10 | f | f | 0.345454545 | 0.1272727 | 0.053763441 | PRE |
| blanca4.571 | blanca | 1 | 4.57 | betta | 0 | 0 | 10 | f | f | 0 | 0.1764706 | 0.00408998 | PRE |
| blanca4.571 | blanca | 1 | 4.57 | braccio | 0 | 0 | 10 | f | f | 0 | 0.14583333 | 0.02 | PRE |
| blanca4.571 | blanca | 1 | 4.57 | black | 0 | 0 | 10 | f | m | 0 | 0 | 0.008130081 | PRE |
| blanca4.571 | blanca | 1 | 4.57 | bernoccolo | 0 | 0 | 10 | f | m | 0 | 0 | 0.01754386 | PRE |
| blind0.81 | blind | 1 | 0.8 | braccio | only seen | 0 | 3 | f | f | 0.0253164 | 0.12658227 | 0.02 | POST |
| blind0.81 | blind | 1 | 0.8 | bisnonna | 0 | 0 | 3 | f | f | 0.19047619 | 0.07142857 | 0.049019608 | POST |
| blind0.81 | blind | 1 | 0.8 | borsa | 0 | 1 | 3 | f | f | 0 | 0.1382549 | 0.067901235 | POST |
| blind1.131 | blind | 1 | 1.13 | biondo | only seen | 0 | 6 | f | m | 0 | 0.1553398 | 0.14021164 | POST |
| blind1.131 | blind | 1 | 1.13 | betta | 0 | 0 | 6 | f | f | 0 | 0.1212121 | 0.00408998 | POST |
| blind1.131 | blind | 1 | 1.13 | blanca | 0 | 0 | 6 | f | f | 0.345454545 | 0.127272727 | 0.032921811 | POST |
| blind1.131 | blind | 1 | 1.13 | barbie | 0 | 0 | 6 | f | f | 0 | 0.12903225 | 0.026666667 | POST |
| blind1.131 | blind | 1 | 1.13 | bratz | 0 | 0 | 6 | f | f | 0 | 0.074074074 | 0.059322034 | POST |
| blind1.131 | blind | 1 | 1.13 | bigne | 0 | 0 | 6 | f | f | 0.0163934 | 0.1147541 | 0.038011696 | POST |
| blind1.213 | blind | 3 | 1.21 | basic | only seen | 0 | 9 | f | f | 0 | 0.02631578 | 0.053030303 | POST |
| blind1.213 | blind | 3 | 1.21 | bigne | 0 | 0 | 9 | f | f | 0.0163934 | 0.1147541 | 0.038011696 | POST |
| blind1.213 | blind | 3 | 1.21 | braccio | 0 | 0 | 9 | f | f | 0.0253164 | 0.12658227 | 0.02 | POST |
| blind1.213 | blind | 3 | 1.21 | blanca | 0 | 0 | 9 | f | f | 0.345454545 | 0.127272727 | 0.032921811 | POST |
| blind1.213 | blind | 3 | 1.21 | black | 0 | 0 | 9 | f | m | 0 | 0.1875 | 0.008130081 | POST |
| blind1.213 | blind | 3 | 1.21 | bernoccolo | 0 | 0 | 9 | f | m | 0 | 0.08 | 0.01754386 | POST |
| blind1.213 | blind | 3 | 1.21 | bortolo | 0 | 0 | 9 | f | m | 0 | 0 | 0.032051282 | POST |
| blind1.213 | blind | 3 | 1.21 | betta | 0 | 0 | 9 | f | f | 0 | 0.1212121 | 0.00408998 | POST |
| blind1.213 | blind | 3 | 1.21 | bisnonna | 0 | 0 | 9 | f | f | 0.19047619 | 0.07142857 | 0.049019608 | POST |
| blind1.221 | blind | 1 | 1.22 | bigne | only seen | 0 | 5 | f | f | 0.0163934 | 0.1147541 | 0.038011696 | POST |
| blind1.221 | blind | 1 | 1.22 | betta | 0 | 0 | 5 | f | f | 0 | 0.1212121 | 0.00408998 | POST |
| blind1.221 | blind | 1 | 1.22 | barbie | 0 | 0 | 5 | f | f | 0 | 0.12903225 | 0.026666667 | POST |
| blind1.221 | blind | 1 | 1.22 | blanca | 0 | 0 | 5 | f | f | 0.345454545 | 0.127272727 | 0.032921811 | POST |
| blind1.221 | blind | 1 | 1.22 | bratz | 0 | 0 | 5 | f | f | 0 | 0.074074074 | 0.059322034 | POST |
| blind1.321 | blind | 1 | 1.32 | blanca | only seen | 0 | 3 | f | f | 0.345454545 | 0.127272727 | 0.032921811 | POST |
| blind1.321 | blind | 1 | 1.32 | belly | 0 | 0 | 3 | f | f | 0 | 0 | 0.024390244 | POST |
| blind1.321 | blind | 1 | 1.32 | bratz | 0 | 0 | 3 | f | f | 0 | 0.074074074 | 0.059322034 | POST |
| blind1.333 | blind | 3 | 1.33 | blanca | only seen | 0 | 9 | f | f | 0.345454545 | 0.127272727 | 0.032921811 | POST |
| blind1.333 | blind | 3 | 1.33 | basic | only seen | 0 | 9 | f | f | 0 | 0.02631578 | 0.053030303 | POST |
| blind1.333 | blind | 3 | 1.33 | bisnonna | only seen | 0 | 9 | f | f | 0.19047619 | 0.07142857 | 0.049019608 | POST |
| blind1.333 | blind | 3 | 1.33 | braccio | only seen | 0 | 9 | f | f | 0.0253164 | 0.12658227 | 0.02 | POST |
| blind1.333 | blind | 3 | 1.33 | barbie | 0 | 0 | 9 | f | f | 0 | 0.12903225 | 0.026666667 | POST |
| blind1.333 | blind | 3 | 1.33 | bigne | 0 | 0 | 9 | f | f | 0.0163934 | 0.1147541 | 0.038011696 | POST |
| blind1.333 | blind | 3 | 1.33 | black | 0 | 0 | 9 | f | m | 0 | 0.1875 | 0.008130081 | POST |
| blind1.333 | blind | 3 | 1.33 | bernoccolo | 0 | 0 | 9 | f | m | 0 | 0.08 | 0.01754386 | POST |
| blind1.333 | blind | 3 | 1.33 | bortolo | 0 | 0 | 9 | f | m | 0 | 0 | 0.032051282 | POST |
| blind1.381 | blind | 1 | 1.38 | bisnonna | only heard | 0 | 10 | f | f | 0.19047619 | 0.07142857 | 0.049019608 | POST |
| blind1.381 | blind | 1 | 1.38 | biondo | only heard | 0 | 10 | f | m | 0 | 0.1553398 | 0.14021164 | POST |
| blind1.381 | blind | 1 | 1.38 | bernoccolo | only heard | 0 | 10 | f | m | 0 | 0.08 | 0.01754386 | POST |
| blind1.381 | blind | 1 | 1.38 | basic | only heard | 0 | 10 | f | f | 0 | 0.02631578 | 0.053030303 | POST |
| blind1.381 | blind | 1 | 1.38 | betta | only heard | 1 | 10 | f | f | 0 | 0.1212121 | 0.00408998 | POST |
| blind1.381 | blind | 1 | 1.38 | braccio | only heard | 0 | 10 | f | f | 0.0253164 | 0.12658227 | 0.02 | POST |
| blind1.381 | blind | 1 | 1.38 | bifida | only heard | 0 | 10 | f | f | 0 | 0.025 | 0.078014184 | POST |
| blind1.381 | blind | 1 | 1.38 | bijoux | only heard | 0 | 10 | f | f | 0 | 0 | 0.061403509 | POST |
| blind1.381 | blind | 1 | 1.38 | barbie | only heard | 1 | 10 | f | f | 0 | 0.12903225 | 0.026666667 | POST |
| blind1.381 | blind | 1 | 1.38 | bratz | only heard | 1 | 10 | f | f | 0 | 0.074074074 | 0.059322034 | POST |
| blind1.451 | blind | 1 | 1.45 | blanca | only seen | 1 | 3 | f | f | 0.345454545 | 0.127272727 | 0.032921811 | POST |
| blind1.451 | blind | 1 | 1.45 | bratz | 0 | 0 | 3 | f | f | 0 | 0.074074074 | 0.059322034 | POST |
| blind1.451 | blind | 1 | 1.45 | belly | 0 | 0 | 3 | f | f | 0 | 0 | 0.024390244 | POST |
| blind1.573 | blind | 3 | 1.57 | bisnonna | only heard | 0 | 4 | f | f | 0.19047619 | 0.07142857 | 0.049019608 | POST |
| blind1.573 | blind | 3 | 1.57 | biondo | only heard | 0 | 4 | f | m | 0 | 0.1553398 | 0.14021164 | POST |
| blind1.573 | blind | 3 | 1.57 | bratz | only heard | 0 | 4 | f | f | 0 | 0.074074074 | 0.059322034 | POST |
| blind1.573 | blind | 3 | 1.57 | basic | only heard | 0 | 4 | f | f | 0 | 0.02631578 | 0.053030303 | POST |
| blind1.621 | blind | 1 | 1.62 | borsa | only seen | 0 | 7 | f | f | 0 | 0.1382549 | 0.067901235 | POST |
| blind1.621 | blind | 1 | 1.62 | bortolo | 0 | 0 | 7 | f | m | 0 | 0 | 0.032051282 | POST |
| blind1.621 | blind | 1 | 1.62 | bernoccolo | 0 | 0 | 7 | f | m | 0 | 0.08 | 0.01754386 | POST |
| blind1.621 | blind | 1 | 1.62 | barbie | 0 | 0 | 7 | f | f | 0 | 0.12903225 | 0.026666667 | POST |
| blind1.621 | blind | 1 | 1.62 | betta | 0 | 0 | 7 | f | f | 0 | 0.1212121 | 0.00408998 | POST |
| blind1.621 | blind | 1 | 1.62 | biondo | 0 | 1 | 7 | f | m | 0 | 0.1553398 | 0.14021164 | POST |
| blind1.691 | blind | 1 | 1.69 | blanca | only seen | 0 | 3 | f | f | 0.345454545 | 0.127272727 | 0.032921811 | POST |
| blind1.691 | blind | 1 | 1.69 | bratz | 0 | 0 | 3 | f | f | 0 | 0.074074074 | 0.059322034 | POST |
| blind1.691 | blind | 1 | 1.69 | belly | 0 | 1 | 3 | f | f | 0 | 0 | 0.024390244 | POST |
| blind1.951 | blind | 1 | 1.95 | braccio | only seen | 1 | 5 | f | f | 0.0253164 | 0.12658227 | 0.02 | POST |
| blind1.951 | blind | 1 | 1.95 | bratz | only seen | 0 | 5 | f | f | 0 | 0.074074074 | 0.059322034 | POST |
| blind1.951 | blind | 1 | 1.95 | betta | 0 | 0 | 5 | f | f | 0 | 0.1212121 | 0.00408998 | POST |
| blind1.951 | blind | 1 | 1.95 | bigne | 0 | 0 | 5 | f | f | 0.0163934 | 0.1147541 | 0.038011696 | POST |
| blind1.951 | blind | 1 | 1.95 | blanca | 0 | 0 | 5 | f | f | 0.345454545 | 0.127272727 | 0.032921811 | POST |
| blind2.061 | blind | 1 | 2.06 | bisnonna | only seen | 1 | 4 | f | f | 0.19047619 | 0.07142857 | 0.049019608 | POST |
| blind2.061 | blind | 1 | 2.06 | borsa | 0 | 0 | 4 | f | f | 0 | 0.1382549 | 0.067901235 | POST |
| blind2.061 | blind | 1 | 2.06 | braccio | 0 | 0 | 4 | f | f | 0.0253164 | 0.12658227 | 0.02 | POST |
| blind2.061 | blind | 1 | 2.06 | bigne | 0 | 0 | 4 | f | f | 0.0163934 | 0.1147541 | 0.038011696 | POST |
| blind2.391 | blind | 1 | 2.39 | bortolo | 0 | 0 | 7 | f | m | 0 | 0 | 0.032051282 | POST |
| blind2.391 | blind | 1 | 2.39 | bratz | 0 | 0 | 7 | f | f | 0 | 0.074074074 | 0.059322034 | POST |
| blind2.391 | blind | 1 | 2.39 | blanca | 0 | 0 | 7 | f | f | 0.345454545 | 0.127272727 | 0.032921811 | POST |
| blind2.391 | blind | 1 | 2.39 | biondo | 0 | 0 | 7 | f | m | 0 | 0.1553398 | 0.14021164 | POST |
| blind2.391 | blind | 1 | 2.39 | betta | 0 | 0 | 7 | f | f | 0 | 0.1212121 | 0.00408998 | POST |
| blind2.391 | blind | 1 | 2.39 | basic | 0 | 0 | 7 | f | f | 0 | 0.02631578 | 0.053030303 | POST |
| blind2.391 | blind | 1 | 2.39 | bifida | 0 | 0 | 7 | f | f | 0 | 0.025 | 0.078014184 | POST |
| borsa0.841 | borsa | 1 | 0.84 | biondo | 0 | 0 | 2 | f | m | 0.0547945 | 0.109589 | 0.14021164 | POST |
| borsa0.841 | borsa | 1 | 0.84 | blanca | 0 | 0 | 2 | f | f | 0.1111111 | 0.0833333 | 0.032921811 | POST |
| borsa1.172 | borsa | 2 | 1.17 | biondo | 0 | 0 | 2 | f | m | 0.0547945 | 0.109589 | 0.14021164 | POST |
| borsa1.172 | borsa | 2 | 1.17 | blanca | 0 | 0 | 2 | f | f | 0.1111111 | 0.0833333 | 0.032921811 | POST |
| borsa1.432 | borsa | 2 | 1.43 | betta | only seen | 0 | 6 | f | f | 0.016129 | 0.0806452 | 0.00408998 | POST |
| borsa1.432 | borsa | 2 | 1.43 | bratz | only seen | 1 | 6 | f | f | 0.09090909 | 0.09090909 | 0.059322034 | POST |
| borsa1.432 | borsa | 2 | 1.43 | bifida | 0 | 0 | 6 | f | f | 0 | 0.0666667 | 0.078014184 | POST |
| borsa1.432 | borsa | 2 | 1.43 | barbie | 0 | 0 | 6 | f | f | 0 | 0.41666666 | 0.026666667 | POST |
| borsa1.432 | borsa | 2 | 1.43 | biondo | 0 | 0 | 6 | f | m | 0.0547945 | 0.109589 | 0.14021164 | POST |
| borsa1.432 | borsa | 2 | 1.43 | braccio | 0 | 0 | 6 | f | f | 0.0350877 | 0.1403509 | 0.02 | POST |
| borsa1.51 | borsa | 1 | 1.5 | bigne | seen and heard | 0 | 1 | f | f | 0.2045455 | 0.1136364 | 0.038011696 | POST |
| borsa1.61 | borsa | 1 | 1.6 | belly | only seen | 0 | 1 | f | f | 0.0625 | 0.0625 | 0.024390244 | POST |
| borsa1.651 | borsa | 1 | 1.65 | bigne | only seen | 1 | 1 | f | f | 0.2045455 | 0.1136364 | 0.038011696 | POST |
| borsa1.681 | borsa | 1 | 1.68 | basic | 0 | 0 | 2 | f | f | 0.1 | 0.1666667 | 0.053030303 | POST |
| borsa1.681 | borsa | 1 | 1.68 | braccio | 0 | 0 | 2 | f | f | 0.0350877 | 0.1403509 | 0.02 | POST |
| borsa1.891 | borsa | 1 | 1.89 | bigne | only seen | 0 | 11 | f | f | 0.2045455 | 0.1136364 | 0.038011696 | POST |
| borsa1.891 | borsa | 1 | 1.89 | braccio | only seen | 0 | 11 | f | f | 0.0350877 | 0.1403509 | 0.02 | POST |
| borsa1.891 | borsa | 1 | 1.89 | bisnonna | only seen | 0 | 11 | f | f | 0.155555556 | 0.044444444 | 0.049019608 | POST |
| borsa1.891 | borsa | 1 | 1.89 | blanca | only seen | 0 | 11 | f | f | 0.1111111 | 0.0833333 | 0.032921811 | POST |
| borsa1.891 | borsa | 1 | 1.89 | biondo | 0 | 0 | 11 | f | m | 0.0547945 | 0.109589 | 0.14021164 | POST |
| borsa1.891 | borsa | 1 | 1.89 | betta | 0 | 0 | 11 | f | f | 0.016129 | 0.0806452 | 0.00408998 | POST |
| borsa1.891 | borsa | 1 | 1.89 | blind | 0 | 0 | 11 | f | f | 0 | 0.1382549 | 0.053763441 | POST |
| borsa1.891 | borsa | 1 | 1.89 | black | 0 | 0 | 11 | f | m | 0.1428571 | 0.2285714 | 0.008130081 | POST |
| borsa1.891 | borsa | 1 | 1.89 | bijoux | 0 | 0 | 11 | f | f | 0 | 0.3125 | 0.061403509 | POST |
| borsa1.891 | borsa | 1 | 1.89 | bortolo | 0 | 0 | 11 | f | m | 0 | 0.136363636 | 0.032051282 | POST |
| borsa1.891 | borsa | 1 | 1.89 | bernoccolo | 0 | 0 | 11 | f | m | 0.05263157 | 0.21052631 | 0.01754386 | POST |
| borsa2.371 | borsa | 1 | 2.37 | bigne | only seen | 0 | 1 | f | f | 0.2045455 | 0.1136364 | 0.038011696 | POST |
| borsa2.512 | borsa | 2 | 2.51 | black | only seen | 0 | 8 | f | m | 0.1428571 | 0.2285714 | 0.008130081 | POST |
| borsa2.512 | borsa | 2 | 2.51 | betta | only seen | 0 | 8 | f | f | 0.016129 | 0.0806452 | 0.00408998 | POST |
| borsa2.512 | borsa | 2 | 2.51 | braccio | only seen | 1 | 8 | f | f | 0.0350877 | 0.1403509 | 0.02 | POST |
| borsa2.512 | borsa | 2 | 2.51 | bifida | 0 | 0 | 8 | f | f | 0 | 0.0666667 | 0.078014184 | POST |
| borsa2.512 | borsa | 2 | 2.51 | basic | 0 | 0 | 8 | f | f | 0.1 | 0.1666667 | 0.053030303 | POST |
| borsa2.512 | borsa | 2 | 2.51 | bratz | 0 | 0 | 8 | f | f | 0.09090909 | 0.09090909 | 0.059322034 | POST |
| borsa2.512 | borsa | 2 | 2.51 | barbie | 0 | 0 | 8 | f | f | 0 | 0.41666666 | 0.026666667 | POST |
| borsa2.512 | borsa | 2 | 2.51 | biondo | 0 | 0 | 8 | f | m | 0.0547945 | 0.109589 | 0.14021164 | POST |
| borsa2.61 | borsa | 1 | 2.6 | braccio | 0 | 0 | 4 | f | f | 0.0350877 | 0.1403509 | 0.02 | POST |
| borsa2.61 | borsa | 1 | 2.6 | bigne | 0 | 0 | 4 | f | f | 0.2045455 | 0.1136364 | 0.038011696 | POST |
| borsa2.61 | borsa | 1 | 2.6 | blind | 0 | 0 | 4 | f | f | 0 | 0.1382549 | 0.053763441 | POST |
| borsa2.61 | borsa | 1 | 2.6 | bisnonna | 0 | 0 | 4 | f | f | 0.155555556 | 0.044444444 | 0.049019608 | POST |
| borsa2.821 | borsa | 1 | 2.82 | blanca | 0 | 0 | 2 | f | f | 0.1111111 | 0.0833333 | 0.032921811 | POST |
| borsa2.821 | borsa | 1 | 2.82 | bigne | 0 | 1 | 2 | f | f | 0.2045455 | 0.1136364 | 0.038011696 | POST |
| borsa2.961 | borsa | 1 | 2.96 | bigne | only seen | 0 | 7 | f | f | 0.2045455 | 0.1136364 | 0.038011696 | POST |
| borsa2.961 | borsa | 1 | 2.96 | biondo | 0 | 1 | 7 | f | m | 0.0547945 | 0.109589 | 0.14021164 | POST |
| borsa2.961 | borsa | 1 | 2.96 | betta | 0 | 0 | 7 | f | f | 0.016129 | 0.0806452 | 0.00408998 | POST |
| borsa2.961 | borsa | 1 | 2.96 | braccio | 0 | 0 | 7 | f | f | 0.0350877 | 0.1403509 | 0.02 | POST |
| borsa2.961 | borsa | 1 | 2.96 | blanca | 0 | 0 | 7 | f | f | 0.1111111 | 0.0833333 | 0.032921811 | POST |
| borsa2.961 | borsa | 1 | 2.96 | blind | 0 | 0 | 7 | f | f | 0 | 0.1382549 | 0.053763441 | POST |
| borsa2.961 | borsa | 1 | 2.96 | bratz | 0 | 0 | 7 | f | f | 0.09090909 | 0.09090909 | 0.059322034 | POST |
| borsa2.971 | borsa | 1 | 2.97 | braccio | only seen | 0 | 5 | f | f | 0.0350877 | 0.1403509 | 0.02 | POST |
| borsa2.971 | borsa | 1 | 2.97 | barbie | 0 | 0 | 5 | f | f | 0 | 0.41666666 | 0.026666667 | POST |
| borsa2.971 | borsa | 1 | 2.97 | betta | 0 | 0 | 5 | f | f | 0.016129 | 0.0806452 | 0.00408998 | POST |
| borsa2.971 | borsa | 1 | 2.97 | bratz | 0 | 0 | 5 | f | f | 0.09090909 | 0.09090909 | 0.059322034 | POST |
| borsa2.971 | borsa | 1 | 2.97 | bifida | 0 | 0 | 5 | f | f | 0 | 0.0666667 | 0.078014184 | POST |
| borsa3.083 | borsa | 3 | 3.08 | biondo | only seen | 1 | 9 | f | m | 0.0547945 | 0.109589 | 0.14021164 | POST |
| borsa3.083 | borsa | 3 | 3.08 | bisnonna | only seen | 0 | 9 | f | f | 0.155555556 | 0.044444444 | 0.049019608 | POST |
| borsa3.083 | borsa | 3 | 3.08 | braccio | only seen | 0 | 9 | f | f | 0.0350877 | 0.1403509 | 0.02 | POST |
| borsa3.083 | borsa | 3 | 3.08 | black | only seen | 0 | 9 | f | m | 0.1428571 | 0.2285714 | 0.008130081 | POST |
| borsa3.083 | borsa | 3 | 3.08 | blanca | only seen | 0 | 9 | f | f | 0.1111111 | 0.0833333 | 0.032921811 | POST |
| borsa3.083 | borsa | 3 | 3.08 | bortolo | 0 | 0 | 9 | f | m | 0 | 0.136363636 | 0.032051282 | POST |
| borsa3.083 | borsa | 3 | 3.08 | bernoccolo | 0 | 0 | 9 | f | m | 0.05263157 | 0.21052631 | 0.01754386 | POST |
| borsa3.083 | borsa | 3 | 3.08 | bijoux | 0 | 0 | 9 | f | f | 0 | 0.3125 | 0.061403509 | POST |
| borsa3.083 | borsa | 3 | 3.08 | betta | 0 | 0 | 9 | f | f | 0.016129 | 0.0806452 | 0.00408998 | POST |
| borsa3.323 | borsa | 3 | 3.32 | black | only seen | 0 | 12 | f | m | 0.1428571 | 0.2285714 | 0.008130081 | POST |
| borsa3.323 | borsa | 3 | 3.32 | bratz | only seen | 1 | 12 | f | f | 0.09090909 | 0.09090909 | 0.059322034 | POST |
| borsa3.323 | borsa | 3 | 3.32 | bisnonna | 0 | 0 | 12 | f | f | 0.155555556 | 0.044444444 | 0.049019608 | POST |
| borsa3.323 | borsa | 3 | 3.32 | braccio | 0 | 0 | 12 | f | f | 0.0350877 | 0.1403509 | 0.02 | POST |
| borsa3.323 | borsa | 3 | 3.32 | bigne | 0 | 0 | 12 | f | f | 0.2045455 | 0.1136364 | 0.038011696 | POST |
| borsa3.323 | borsa | 3 | 3.32 | biondo | 0 | 0 | 12 | f | m | 0.0547945 | 0.109589 | 0.14021164 | POST |
| borsa3.323 | borsa | 3 | 3.32 | blanca | 0 | 0 | 12 | f | f | 0.1111111 | 0.0833333 | 0.032921811 | POST |
| borsa3.323 | borsa | 3 | 3.32 | basic | 0 | 0 | 12 | f | f | 0.1 | 0.1666667 | 0.053030303 | POST |
| borsa3.323 | borsa | 3 | 3.32 | blind | 0 | 0 | 12 | f | f | 0 | 0.1382549 | 0.053763441 | POST |
| borsa3.323 | borsa | 3 | 3.32 | betta | 0 | 0 | 12 | f | f | 0.016129 | 0.0806452 | 0.00408998 | POST |
| borsa3.323 | borsa | 3 | 3.32 | bijoux | 0 | 0 | 12 | f | f | 0 | 0.3125 | 0.061403509 | POST |
| borsa3.323 | borsa | 3 | 3.32 | belly | 0 | 0 | 12 | f | f | 0.0625 | 0.0625 | 0.024390244 | POST |
| borsa3.521 | borsa | 1 | 3.52 | bigne | only seen | 0 | 6 | f | f | 0.2045455 | 0.1136364 | 0.038011696 | POST |
| borsa3.521 | borsa | 1 | 3.52 | biondo | 0 | 0 | 6 | f | m | 0.0547945 | 0.109589 | 0.14021164 | POST |
| borsa3.521 | borsa | 1 | 3.52 | betta | 0 | 0 | 6 | f | f | 0.016129 | 0.0806452 | 0.00408998 | POST |
| borsa3.521 | borsa | 1 | 3.52 | blind | 0 | 0 | 6 | f | f | 0 | 0.1382549 | 0.053763441 | POST |
| borsa3.521 | borsa | 1 | 3.52 | blanca | 0 | 0 | 6 | f | f | 0.1111111 | 0.0833333 | 0.032921811 | POST |
| borsa3.521 | borsa | 1 | 3.52 | bratz | 0 | 0 | 6 | f | f | 0.09090909 | 0.09090909 | 0.059322034 | POST |
| borsa4.123 | borsa | 3 | 4.12 | blanca | 0 | 0 | 3 | f | f | 0.1111111 | 0.0833333 | 0.032921811 | POST |
| borsa4.123 | borsa | 3 | 4.12 | bigne | 0 | 0 | 3 | f | f | 0.2045455 | 0.1136364 | 0.038011696 | POST |
| borsa4.123 | borsa | 3 | 4.12 | black | 0 | 0 | 3 | f | m | 0.1428571 | 0.2285714 | 0.008130081 | POST |
| bortolo1.561 | bortolo | 1 | 1.56 | betta | 0 | 0 | 5 | m | f | 0 | 0 | 0.00408998 | POST |
| bortolo1.561 | bortolo | 1 | 1.56 | borsa | 0 | 0 | 5 | m | f | 0 | 0.136363636 | 0.067901235 | POST |
| bortolo1.561 | bortolo | 1 | 1.56 | black | 0 | 0 | 5 | m | m | 0 | 0.066666667 | 0.008130081 | POST |
| bortolo1.561 | bortolo | 1 | 1.56 | bratz | 0 | 0 | 5 | m | f | 0 | 0.1666667 | 0.059322034 | POST |
| bortolo1.561 | bortolo | 1 | 1.56 | barbie | 0 | 1 | 5 | m | f | 0 | 0.113636364 | 0.026666667 | POST |
| bortolo1.621 | bortolo | 1 | 1.62 | biondo | only seen | 0 | 6 | m | m | 0 | 0.0588235 | 0.14021164 | POST |
| bortolo1.621 | bortolo | 1 | 1.62 | black | 0 | 0 | 6 | m | m | 0 | 0.066666667 | 0.008130081 | POST |
| bortolo1.621 | bortolo | 1 | 1.62 | bisnonna | 0 | 0 | 6 | m | f | 0 | 0.12 | 0.049019608 | POST |
| bortolo1.621 | bortolo | 1 | 1.62 | blind | 0 | 0 | 6 | m | f | 0 | 0 | 0.053763441 | POST |
| bortolo1.621 | bortolo | 1 | 1.62 | braccio | 0 | 0 | 6 | m | f | 0 | 0.074074074 | 0.02 | POST |
| bortolo1.621 | bortolo | 1 | 1.62 | bigne | 0 | 0 | 6 | m | f | 0 | 0.2857143 | 0.038011696 | POST |
| bortolo1.962 | bortolo | 2 | 1.96 | bisnonna | only seen | 0 | 8 | m | f | 0 | 0.12 | 0.049019608 | POST |
| bortolo1.962 | bortolo | 2 | 1.96 | blind | only seen | 0 | 8 | m | f | 0 | 0 | 0.053763441 | POST |
| bortolo1.962 | bortolo | 2 | 1.96 | bijoux | only seen | 0 | 8 | m | f | 0.15384615 | 0 | 0.061403509 | POST |
| bortolo1.962 | bortolo | 2 | 1.96 | barbie | only seen | 0 | 8 | m | f | 0 | 0.285714286 | 0.026666667 | POST |
| bortolo1.962 | bortolo | 2 | 1.96 | basic | only seen | 0 | 8 | m | f | 0 | 0 | 0.053030303 | POST |
| bortolo1.962 | bortolo | 2 | 1.96 | borsa | 0 | 0 | 8 | m | f | 0 | 0.136363636 | 0.067901235 | POST |
| bortolo1.962 | bortolo | 2 | 1.96 | black | 0 | 0 | 8 | m | m | 0 | 0.066666667 | 0.008130081 | POST |
| bortolo2.291 | bortolo | 1 | 2.29 | betta | only seen | 0 | 5 | m | f | 0 | 0 | 0.00408998 | POST |
| bortolo2.291 | bortolo | 1 | 2.29 | braccio | only seen | 0 | 5 | m | f | 0 | 0.074074074 | 0.02 | POST |
| bortolo2.291 | bortolo | 1 | 2.29 | borsa | only seen | 0 | 5 | m | f | 0 | 0.136363636 | 0.067901235 | POST |
| bortolo2.291 | bortolo | 1 | 2.29 | black | 0 | 0 | 5 | m | m | 0 | 0.066666667 | 0.008130081 | POST |
| bortolo2.291 | bortolo | 1 | 2.29 | bernoccolo | 0 | 0 | 5 | m | m | 0.1 | 0.2173913 | 0.01754386 | POST |
| bortolo4.591 | bortolo | 1 | 4.59 | biondo | 0 | 0 | 3 | m | m | 0 | 0.0588235 | 0.14021164 | POST |
| bortolo4.591 | bortolo | 1 | 4.59 | bigne | 0 | 0 | 3 | m | f | 0 | 0.2857143 | 0.038011696 | PRE |
| bortolo4.591 | bortolo | 1 | 4.59 | bifida | 0 | 0 | 3 | m | f | 0 | 0 | 0.078014184 | PRE |
| braccio1.211 | braccio | 1 | 1.21 | blanca | only seen | 1 | 4 | f | f | 0 | 0.145833333 | 0.032921811 | POST |
| braccio1.211 | braccio | 1 | 1.21 | bratz | only seen | 0 | 4 | f | f | 0.05172413 | 0.08620689 | 0.059322034 | POST |
| braccio1.211 | braccio | 1 | 1.21 | biondo | only seen | 0 | 4 | f | m | 0.1209677 | 0.1048387 | 0.14021164 | POST |
| braccio1.211 | braccio | 1 | 1.21 | blind | only seen | 0 | 4 | f | f | 0.0253164 | 0.12658227 | 0.053763441 | POST |
| braccio1.313 | braccio | 3 | 1.31 | biondo | 0 | 0 | 3 | f | m | 0.1209677 | 0.1048387 | 0.14021164 | POST |
| braccio1.313 | braccio | 3 | 1.31 | betta | 0 | 0 | 3 | f | f | 0.0568182 | 0.25 | 0.00408998 | POST |
| braccio1.313 | braccio | 3 | 1.31 | bortolo | 0 | 0 | 3 | f | m | 0 | 0.074074074 | 0.032051282 | POST |
| braccio1.5981 | braccio | 1 | 1.598 | bifida | only seen | 0 | 3 | f | f | 0.0204082 | 0.122449 | 0.078014184 | POST |
| braccio1.5981 | braccio | 1 | 1.598 | bisnonna | 0 | 0 | 3 | f | f | 0.027027027 | 0.040540541 | 0.049019608 | POST |
| braccio1.5981 | braccio | 1 | 1.598 | bigne | 0 | 0 | 3 | f | f | 0.0350877 | 0.17543859 | 0.038011696 | POST |
| braccio1.6191 | braccio | 1 | 1.619 | bigne | only seen | 0 | 1 | f | f | 0.0350877 | 0.17543859 | 0.038011696 | POST |
| braccio1.621 | braccio | 1 | 1.62 | bisnonna | 0 | 0 | 1 | f | f | 0.027027027 | 0.040540541 | 0.049019608 | POST |
| braccio1.6821 | braccio | 1 | 1.682 | bifida | only seen | 1 | 3 | f | f | 0.0204082 | 0.122449 | 0.078014184 | POST |
| braccio1.6821 | braccio | 1 | 1.682 | bisnonna | 0 | 0 | 3 | f | f | 0.027027027 | 0.040540541 | 0.049019608 | POST |
| braccio1.6821 | braccio | 1 | 1.682 | bigne | 0 | 0 | 3 | f | f | 0.0350877 | 0.17543859 | 0.038011696 | POST |
| braccio1.721 | braccio | 1 | 1.72 | bifida | only seen | 0 | 8 | f | f | 0.0204082 | 0.122449 | 0.078014184 | POST |
| braccio1.721 | braccio | 1 | 1.72 | bisnonna | only seen | 0 | 8 | f | f | 0.027027027 | 0.040540541 | 0.049019608 | POST |
| braccio1.721 | braccio | 1 | 1.72 | bratz | only seen | 0 | 8 | f | f | 0.05172413 | 0.08620689 | 0.059322034 | POST |
| braccio1.721 | braccio | 1 | 1.72 | blanca | only seen | 0 | 8 | f | f | 0 | 0.145833333 | 0.032921811 | POST |
| braccio1.721 | braccio | 1 | 1.72 | blind | only seen | 0 | 8 | f | f | 0.0253164 | 0.12658227 | 0.053763441 | PRE |
| braccio1.721 | braccio | 1 | 1.72 | borsa | only seen | 0 | 8 | f | f | 0.0350877 | 0.1403509 | 0.067901235 | PRE |
| braccio1.721 | braccio | 1 | 1.72 | belly | 0 | 0 | 8 | f | f | 0 | 0.05263157 | 0.024390244 | PRE |
| braccio1.721 | braccio | 1 | 1.72 | betta | 0 | 0 | 8 | f | f | 0.0568182 | 0.25 | 0.00408998 | PRE |
| braccio2.333 | braccio | 3 | 2.33 | biondo | only seen | 1 | 1 | f | m | 0.1209677 | 0.1048387 | 0.14021164 | PRE |
| bratz1.151 | bratz | 1 | 1.15 | betta | only seen | 0 | 2 | f | f | 0 | 0.140625 | 0.00408998 | POST |
| bratz1.151 | bratz | 1 | 1.15 | basic | 0 | 0 | 2 | f | f | 0.31111111 | 0.04444444 | 0.053030303 | POST |
| bratz1.541 | bratz | 1 | 1.54 | belly | 0 | 0 | 2 | f | f | 0.296296296 | 0.037037037 | 0.024390244 | POST |
| bratz1.541 | bratz | 1 | 1.54 | barbie | 0 | 0 | 2 | f | f | 0.26923076 | 0.285714286 | 0.026666667 | POST |
| bratz1.551 | bratz | 1 | 1.55 | barbie | only seen | 0 | 5 | f | f | 0.26923076 | 0.285714286 | 0.026666667 | POST |
| bratz1.551 | bratz | 1 | 1.55 | biondo | only seen | 0 | 5 | f | m | 0 | 0.0740741 | 0.14021164 | POST |
| bratz1.551 | bratz | 1 | 1.55 | bigne | 0 | 0 | 5 | f | f | 0 | 0.0454545 | 0.038011696 | POST |
| bratz1.551 | bratz | 1 | 1.55 | blanca | 0 | 0 | 5 | f | f | 0.08163265 | 0.12244897 | 0.032921811 | POST |
| bratz1.551 | bratz | 1 | 1.55 | betta | 0 | 0 | 5 | f | f | 0 | 0.140625 | 0.00408998 | POST |
| bratz1.581 | bratz | 1 | 1.58 | basic | 0 | 0 | 1 | f | f | 0.31111111 | 0.04444444 | 0.053030303 | POST |
| bratz1.641 | bratz | 1 | 1.64 | braccio | 0 | 0 | 9 | f | f | 0.05172413 | 0.08620689 | 0.02 | POST |
| bratz1.641 | bratz | 1 | 1.64 | bigne | 0 | 0 | 9 | f | f | 0 | 0.0454545 | 0.038011696 | POST |
| bratz1.641 | bratz | 1 | 1.64 | biondo | 0 | 0 | 9 | f | m | 0 | 0.0740741 | 0.14021164 | POST |
| bratz1.641 | bratz | 1 | 1.64 | bisnonna | 0 | 0 | 9 | f | f | 0 | 0.0625 | 0.049019608 | POST |
| bratz1.641 | bratz | 1 | 1.64 | blind | 0 | 0 | 9 | f | f | 0 | 0.074074074 | 0.053763441 | POST |
| bratz1.641 | bratz | 1 | 1.64 | bijoux | 0 | 0 | 9 | f | f | 0 | 0 | 0.061403509 | POST |
| bratz1.641 | bratz | 1 | 1.64 | betta | 0 | 0 | 9 | f | f | 0 | 0.140625 | 0.00408998 | POST |
| bratz1.641 | bratz | 1 | 1.64 | black | 0 | 0 | 9 | f | m | 0.3414634 | 0.2439024 | 0.008130081 | POST |
| bratz1.641 | bratz | 1 | 1.64 | belly | 0 | 0 | 9 | f | f | 0.296296296 | 0.037037037 | 0.024390244 | POST |
| bratz1.81 | bratz | 1 | 1.8 | blanca | only heard | 0 | 3 | f | f | 0.08163265 | 0.12244897 | 0.032921811 | POST |
| bratz1.81 | bratz | 1 | 1.8 | bifida | only heard | 0 | 3 | f | f | 0.0681818 | 0.1590909 | 0.078014184 | POST |
| bratz1.81 | bratz | 1 | 1.8 | belly | only heard | 0 | 3 | f | f | 0.296296296 | 0.037037037 | 0.024390244 | POST |
| bratz1.911 | bratz | 1 | 1.91 | basic | 0 | 0 | 1 | f | f | 0.31111111 | 0.04444444 | 0.053030303 | POST |
| bratz1.9763 | bratz | 3 | 1.976 | blanca | only seen | 0 | 3 | f | f | 0.08163265 | 0.12244897 | 0.032921811 | POST |
| bratz1.9763 | bratz | 3 | 1.976 | biondo | 0 | 0 | 3 | f | m | 0 | 0.0740741 | 0.14021164 | POST |
| bratz1.9763 | bratz | 3 | 1.976 | betta | 0 | 0 | 3 | f | f | 0 | 0.140625 | 0.00408998 | POST |
| bratz2.033 | bratz | 3 | 2.03 | biondo | only heard | 0 | 7 | f | m | 0 | 0.0740741 | 0.14021164 | POST |
| bratz2.033 | bratz | 3 | 2.03 | bisnonna | only heard | 0 | 7 | f | f | 0 | 0.0625 | 0.049019608 | POST |
| bratz2.033 | bratz | 3 | 2.03 | bortolo | only heard | 0 | 7 | f | m | 0 | 0.1666667 | 0.032051282 | POST |
| bratz2.033 | bratz | 3 | 2.03 | black | only heard | 0 | 7 | f | m | 0.3416434 | 0.2439024 | 0.008130081 | POST |
| bratz2.033 | bratz | 3 | 2.03 | basic | only heard | 0 | 7 | f | f | 0.31111111 | 0.04444444 | 0.053030303 | POST |
| bratz2.033 | bratz | 3 | 2.03 | bigne | only heard | 0 | 7 | f | f | 0 | 0.0454545 | 0.038011696 | POST |
| bratz2.033 | bratz | 3 | 2.03 | bifida | only heard | 0 | 7 | f | f | 0.0681818 | 0.1590909 | 0.078014184 | POST |
| bratz2.083 | bratz | 3 | 2.08 | black | 0 | 0 | 1 | f | m | 0.3414634 | 0.2439024 | 0.008130081 | POST |
| bratz2.233 | bratz | 3 | 2.23 | blind | only seen | 0 | 3 | f | f | 0 | 0.074074074 | 0.053763441 | POST |
| bratz2.233 | bratz | 3 | 2.23 | betta | only seen | 0 | 3 | f | f | 0 | 0.140625 | 0.00408998 | POST |
| bratz2.233 | bratz | 3 | 2.23 | bijoux | only seen | 0 | 3 | f | f | 0 | 0 | 0.061403509 | POST |
| bratz2.2511 | bratz | 1 | 2.251 | black | only seen | 0 | 7 | f | m | 0.3416434 | 0.2439024 | 0.008130081 | POST |
| bratz2.2511 | bratz | 1 | 2.251 | betta | only seen | 0 | 7 | f | f | 0 | 0.140625 | 0.00408998 | POST |
| bratz2.2511 | bratz | 1 | 2.251 | bifida | only seen | 0 | 7 | f | f | 0.0681818 | 0.1590909 | 0.078014184 | POST |
| bratz2.2511 | bratz | 1 | 2.251 | bisnonna | 0 | 0 | 7 | f | f | 0 | 0.0625 | 0.049019608 | POST |
| bratz2.2511 | bratz | 1 | 2.251 | blanca | 0 | 0 | 7 | f | f | 0.08163265 | 0.12244897 | 0.032921811 | POST |
| bratz2.2511 | bratz | 1 | 2.251 | biondo | 0 | 0 | 7 | f | m | 0 | 0.0740741 | 0.14021164 | POST |
| bratz2.2511 | bratz | 1 | 2.251 | blind | 0 | 0 | 7 | f | f | 0 | 0.074074074 | 0.053763441 | POST |
| bratz2.2951 | bratz | 1 | 2.295 | biba | only seen | 0 | 8 | f | f | 0 | 0.125 | 0.011111111 | POST |
| bratz2.2951 | bratz | 1 | 2.295 | braccio | only seen | 0 | 8 | f | f | 0.05172413 | 0.08620869 | 0.02 | POST |
| bratz2.2951 | bratz | 1 | 2.295 | bifida | only seen | 0 | 8 | f | f | 0.0681818 | 0.1590909 | 0.078014184 | POST |
| bratz2.2951 | bratz | 1 | 2.295 | betta | 0 | 0 | 8 | f | f | 0 | 0.140625 | 0.00408998 | POST |
| bratz2.2951 | bratz | 1 | 2.295 | bisnonna | 0 | 0 | 8 | f | f | 0 | 0.0625 | 0.049019608 | POST |
| bratz2.2951 | bratz | 1 | 2.295 | blanca | 0 | 0 | 8 | f | f | 0.08163265 | 0.12244897 | 0.032921811 | POST |
| bratz2.2951 | bratz | 1 | 2.295 | biondo | 0 | 0 | 8 | f | m | 0 | 0.0740741 | 0.14021164 | POST |
| bratz2.2951 | bratz | 1 | 2.295 | blind | 0 | 0 | 8 | f | f | 0 | 0.074074074 | 0.053763441 | POST |
| bratz2.751 | bratz | 1 | 2.75 | braccio | only seen | 0 | 6 | f | f | 0.05172413 | 0.08620689 | 0.02 | POST |
| bratz2.751 | bratz | 1 | 2.75 | bisnonna | 0 | 0 | 6 | f | f | 0 | 0.0625 | 0.049019608 | POST |
| bratz2.751 | bratz | 1 | 2.75 | borsa | 0 | 0 | 6 | f | f | 0.09090909 | 0.09090909 | 0.067901235 | POST |
| bratz2.751 | bratz | 1 | 2.75 | bigne | 0 | 0 | 6 | f | f | 0 | 0.0454545 | 0.038011696 | POST |
| bratz2.751 | bratz | 1 | 2.75 | blind | 0 | 0 | 6 | f | f | 0 | 0.074074074 | 0.053763441 | POST |
| bratz2.751 | bratz | 1 | 2.75 | betta | 0 | 0 | 6 | f | f | 0 | 0.140625 | 0.00408998 | POST |
| bratz2.931 | bratz | 1 | 2.93 | black | seen and heard | 1 | 5 | f | m | 0.3414634 | 0.2439024 | 0.008130081 | POST |
| bratz2.931 | bratz | 1 | 2.93 | borsa | only heard | 0 | 5 | f | f | 0.204545455 | 0.113636364 | 0.067901235 | POST |
| bratz2.931 | bratz | 1 | 2.93 | bortolo | only heard | 0 | 5 | f | m | 0 | 0.1666667 | 0.032051282 | POST |
| bratz2.931 | bratz | 1 | 2.93 | betta | only heard | 0 | 5 | f | f | 0 | 0.140625 | 0.00408998 | POST |
| bratz2.931 | bratz | 1 | 2.93 | barbie | only heard | 0 | 5 | f | f | 0.26923076 | 0.113636364 | 0.026666667 | POST |
| bratz3.211 | bratz | 1 | 3.21 | betta | only seen | 0 | 11 | f | f | 0 | 0.140625 | 0.00408998 | POST |
| bratz3.211 | bratz | 1 | 3.21 | bisnonna | only seen | 0 | 11 | f | f | 0 | 0.0625 | 0.049019608 | POST |
| bratz3.211 | bratz | 1 | 3.21 | bernoccolo | only seen | 0 | 11 | f | m | 0 | 0.14285714 | 0.01754386 | POST |
| bratz3.211 | bratz | 1 | 3.21 | blanca | only seen | 0 | 11 | f | f | 0.08163265 | 0.12244897 | 0.032921811 | POST |
| bratz3.211 | bratz | 1 | 3.21 | basic | 0 | 0 | 11 | f | f | 0.31111111 | 0.04444444 | 0.053030303 | PRE |
| bratz3.211 | bratz | 1 | 3.21 | barbie | 0 | 0 | 11 | f | f | 0.26923076 | 0.285714286 | 0.026666667 | PRE |
| bratz3.211 | bratz | 1 | 3.21 | borsa | 0 | 0 | 11 | f | f | 0.09090909 | 0.09090909 | 0.067901235 | PRE |
| bratz3.211 | bratz | 1 | 3.21 | black | 0 | 0 | 11 | f | m | 0.3416434 | 0.2439024 | 0.008130081 | PRE |
| bratz3.211 | bratz | 1 | 3.21 | bifida | 0 | 0 | 11 | f | f | 0.0681818 | 0.1590909 | 0.078014184 | PRE |
| bratz3.211 | bratz | 1 | 3.21 | biondo | 0 | 0 | 11 | f | m | 0 | 0.0740741 | 0.14021164 | PRE |
| bratz3.3113 | bratz | 3 | 3.311 | blind | only seen | 0 | 5 | f | f | 0 | 0.074074074 | 0.053763441 | PRE |
| bratz3.3113 | bratz | 3 | 3.311 | braccio | 0 | 0 | 5 | f | f | 0.05172413 | 0.08620869 | 0.02 | PRE |
| bratz3.3113 | bratz | 3 | 3.311 | biondo | 0 | 0 | 5 | f | m | 0 | 0.0740741 | 0.14021164 | PRE |
| bratz3.3113 | bratz | 3 | 3.311 | borsa | 0 | 0 | 5 | f | f | 0.09090909 | 0.09090909 | 0.067901235 | PRE |
| bratz3.3113 | bratz | 3 | 3.311 | blanca | 0 | 0 | 5 | f | f | 0.08163265 | 0.12244897 | 0.032921811 | PRE |
| bratz3.433 | bratz | 3 | 3.43 | black | seen and heard | 0 | 10 | f | m | 0.3414634 | 0.2439024 | 0.008130081 | PRE |
| bratz3.433 | bratz | 3 | 3.43 | bijoux | only heard | 0 | 10 | f | f | 0 | 0 | 0.061403509 | PRE |
| bratz3.433 | bratz | 3 | 3.43 | bisnonna | only heard | 0 | 10 | f | f | 0 | 0.0625 | 0.049019608 | PRE |
| bratz3.433 | bratz | 3 | 3.43 | betta | only heard | 0 | 10 | f | f | 0 | 0.140625 | 0.00408998 | PRE |
| bratz3.433 | bratz | 3 | 3.43 | biondo | only heard | 0 | 10 | f | m | 0 | 0.0740741 | 0.14021164 | PRE |
| bratz3.433 | bratz | 3 | 3.43 | blanca | only heard | 0 | 10 | f | f | 0.08163265 | 0.12244897 | 0.032921811 | PRE |
| bratz3.433 | bratz | 3 | 3.43 | basic | only heard | 0 | 10 | f | f | 0.31111111 | 0.04444444 | 0.053030303 | PRE |
| bratz3.433 | bratz | 3 | 3.43 | blind | only heard | 0 | 10 | f | f | 0 | 0.074074074 | 0.053763441 | PRE |
| bratz3.433 | bratz | 3 | 3.43 | belly | only heard | 0 | 10 | f | f | 0.296296296 | 0.037037037 | 0.024390244 | PRE |
| bratz4.183 | bratz | 3 | 4.18 | biondo | only seen | 0 | 12 | f | m | 0 | 0.0740741 | 0.14021164 | PRE |
| bratz4.183 | bratz | 3 | 4.18 | black | 0 | 0 | 12 | f | m | 0.3416434 | 0.2439024 | 0.008130081 | PRE |
| bratz4.183 | bratz | 3 | 4.18 | bifida | 0 | 0 | 12 | f | f | 0.0681818 | 0.1590909 | 0.078014184 | PRE |
| bratz4.183 | bratz | 3 | 4.18 | basic | 0 | 0 | 12 | f | f | 0.31111111 | 0.04444444 | 0.053030303 | PRE |
| bratz4.183 | bratz | 3 | 4.18 | bigne | 0 | 0 | 12 | f | f | 0 | 0.0454545 | 0.038011696 | PRE |
| bratz4.183 | bratz | 3 | 4.18 | braccio | 0 | 0 | 12 | f | f | 0.05172413 | 0.08620689 | 0.02 | PRE |
| bratz4.183 | bratz | 3 | 4.18 | bortolo | 0 | 0 | 12 | f | m | 0 | 0.1666667 | 0.032051282 | PRE |
| bratz4.183 | bratz | 3 | 4.18 | barbie | 0 | 0 | 12 | f | f | 0.26923076 | 0.285714286 | 0.026666667 | PRE |
| bratz4.183 | bratz | 3 | 4.18 | belly | 0 | 0 | 12 | f | f | 0.296296296 | 0.037037037 | 0.024390244 | PRE |
| bratz4.183 | bratz | 3 | 4.18 | betta | 0 | 0 | 12 | f | f | 0 | 0.140625 | 0.00408998 | PRE |
| bratz4.183 | bratz | 3 | 4.18 | borsa | 0 | 0 | 12 | f | f | 0.09090909 | 0.09090909 | 0.067901235 | PRE |
| e1m61.9571 | e1m6 | 1 | 1.957 | striscia | only seen | 0 | 1 | f | f | 0 | 0.13095238 | 0.00862069 | PRE |
| e2m61.4591 | e2m6 | 1 | 1.459 | elly | 0 | 0 | 2 | f | f | 0.08695652 | 0.04347826 | 0 | PRE |
| e2m61.4591 | e2m6 | 1 | 1.459 | edera | 0 | 0 | 2 | f | f | 0 | 0.06896551 | 0 | PRE |
| edera1.8071 | edera | 1 | 1.807 | strip | 0 | 0 | 4 | f | f | 0 | 0.11392405 | 0.020114943 | PRE |
| edera1.8071 | edera | 1 | 1.807 | striscia | 0 | 0 | 4 | f | f | 0.01265822 | 0.1392405 | 0.00862069 | PRE |
| edera1.8071 | edera | 1 | 1.807 | elly | 0 | 0 | 4 | f | f | 0 | 0.21428571 | 0 | PRE |
| edera1.8071 | edera | 1 | 1.807 | enzo | 0 | 0 | 4 | f | m | 0 | 0 | 0.181102362 | PRE |
| elly1.211 | elly | 1 | 1.21 | evasa | 0 | 0 | 3 | f | f | 0 | 0 | 0.024024024 | PRE |
| elly1.211 | elly | 1 | 1.21 | edera | 0 | 0 | 3 | f | f | 0 | 0.21428571 | 0 | PRE |
| elly1.211 | elly | 1 | 1.21 | e2m6 | 0 | 0 | 3 | f | f | 0.08695652 | 0.04347826 | 0 | PRE |
| enzo1.0693 | enzo | 3 | 1.069 | strip | seen and heard | 0 | 4 | m | f | 0.04672897 | 0.18691588 | 0.020114943 | POST |
| enzo1.0693 | enzo | 3 | 1.069 | evasa | only heard | 0 | 4 | m | f | 0 | 0.14432989 | 0.024024024 | POST |
| enzo1.0693 | enzo | 3 | 1.069 | striscia | only heard | 0 | 4 | m | f | 0.06862745 | 0.14705882 | 0.00862069 | POST |
| enzo1.0693 | enzo | 3 | 1.069 | edera | only heard | 0 | 4 | m | f | 0 | 0.133333333 | 0 | POST |
| enzo1.1363 | enzo | 3 | 1.136 | sumo | seen and heard | 0 | 1 | m | m | 0 | 0 | 0.203125 | POST |
| enzo1.223 | enzo | 3 | 1.22 | e1m6 | seen and heard | 0 | 3 | m | f | 0 | 0.16470588 | 0.007092199 | POST |
| enzo1.223 | enzo | 3 | 1.22 | striscia | seen and heard | 0 | 3 | m | f | 0.06862745 | 0.14705882 | 0.00862069 | POST |
| enzo1.223 | enzo | 3 | 1.22 | e2m6 | only heard | 0 | 3 | m | f | 0 | 0.10526315 | 0 | POST |
| enzo1.2393 | enzo | 3 | 1.239 | evasa | seen and heard | 0 | 7 | m | f | 0 | 0.14432989 | 0.024024024 | POST |
| enzo1.2393 | enzo | 3 | 1.239 | e2m6 | seen and heard | 0 | 7 | m | f | 0 | 0.10526315 | 0 | POST |
| enzo1.2393 | enzo | 3 | 1.239 | e1m6 | seen and heard | 1 | 7 | m | f | 0 | 0.16470588 | 0.007092199 | POST |
| enzo1.2393 | enzo | 3 | 1.239 | edera | seen and heard | 0 | 7 | m | f | 0 | 0.133333333 | 0 | POST |
| enzo1.2393 | enzo | 3 | 1.239 | striscia | seen and heard | 0 | 7 | m | f | 0.06862745 | 0.14705882 | 0.00862069 | POST |
| enzo1.2393 | enzo | 3 | 1.239 | strip | seen and heard | 0 | 7 | m | f | 0.04672897 | 0.18691588 | 0.020114943 | POST |
| enzo1.2393 | enzo | 3 | 1.239 | elly | seen and heard | 0 | 7 | m | f | 0.05882352 | 0.17647058 | 0 | POST |
| enzo1.2462 | enzo | 2 | 1.246 | evasa | only heard | 1 | 3 | m | f | 0 | 0.14432989 | 0.024024024 | POST |
| enzo1.2462 | enzo | 2 | 1.246 | striscia | only heard | 0 | 3 | m | f | 0.06862745 | 0.14705882 | 0.00862069 | POST |
| enzo1.2462 | enzo | 2 | 1.246 | strip | only heard | 0 | 3 | m | f | 0.04672897 | 0.18691588 | 0.020114943 | POST |
| enzo1.2483 | enzo | 3 | 1.248 | evasa | seen and heard | 0 | 3 | m | f | 0 | 0.14432989 | 0.024024024 | POST |
| enzo1.2483 | enzo | 3 | 1.248 | strip | seen and heard | 0 | 3 | m | f | 0.04672897 | 0.18691588 | 0.020114943 | POST |
| enzo1.2483 | enzo | 3 | 1.248 | striscia | only heard | 0 | 3 | m | f | 0.06862745 | 0.14705882 | 0.00862069 | POST |
| enzo1.251 | enzo | 1 | 1.25 | evasa | seen and heard | 0 | 2 | m | f | 0 | 0.14432989 | 0.024024024 | POST |
| enzo1.251 | enzo | 1 | 1.25 | striscia | only heard | 0 | 2 | m | f | 0.06862745 | 0.14705882 | 0.00862069 | POST |
| enzo1.3333 | enzo | 3 | 1.333 | striscia | seen and heard | 0 | 1 | m | f | 0.06862745 | 0.14705882 | 0.00862069 | POST |
| enzo1.3381 | enzo | 1 | 1.338 | evasa | only heard | 1 | 2 | m | f | 0 | 0.14432989 | 0.024024024 | POST |
| enzo1.3381 | enzo | 1 | 1.338 | striscia | only heard | 0 | 2 | m | f | 0.06862745 | 0.14705882 | 0.00862069 | POST |
| enzo1.3423 | enzo | 3 | 1.342 | e2m6 | seen and heard | 0 | 5 | m | f | 0 | 0.10526315 | 0 | POST |
| enzo1.3423 | enzo | 3 | 1.342 | e1m6 | seen and heard | 0 | 5 | m | f | 0 | 0.16470588 | 0.007092199 | POST |
| enzo1.3423 | enzo | 3 | 1.342 | strip | only heard | 0 | 5 | m | f | 0.04672897 | 0.18691588 | 0.020114943 | POST |
| enzo1.3423 | enzo | 3 | 1.342 | striscia | only heard | 1 | 5 | m | f | 0.06862745 | 0.14705882 | 0.00862069 | POST |
| enzo1.3423 | enzo | 3 | 1.342 | evasa | only heard | 1 | 5 | m | f | 0 | 0.14432989 | 0.024024024 | POST |
| enzo1.353 | enzo | 3 | 1.35 | evasa | seen and heard | 0 | 3 | m | f | 0 | 0.14432989 | 0.024024024 | POST |
| enzo1.353 | enzo | 3 | 1.35 | strip | seen and heard | 0 | 3 | m | f | 0.04672897 | 0.18691588 | 0.020114943 | POST |
| enzo1.353 | enzo | 3 | 1.35 | striscia | seen and heard | 0 | 3 | m | f | 0.06862745 | 0.14705882 | 0.00862069 | POST |
| enzo1.3792 | enzo | 2 | 1.379 | evasa | only heard | 0 | 3 | m | f | 0 | 0.14432989 | 0.024024024 | POST |
| enzo1.3792 | enzo | 2 | 1.379 | striscia | only heard | 0 | 3 | m | f | 0.06862745 | 0.14705882 | 0.00862069 | POST |
| enzo1.3792 | enzo | 2 | 1.379 | strip | only heard | 0 | 3 | m | f | 0.04672897 | 0.18691588 | 0.020114943 | POST |
| enzo1.3833 | enzo | 3 | 1.383 | strip | seen and heard | 0 | 4 | m | f | 0.04672897 | 0.18691588 | 0.020114943 | POST |
| enzo1.3833 | enzo | 3 | 1.383 | edera | seen and heard | 0 | 4 | m | f | 0 | 0.133333333 | 0 | POST |
| enzo1.3833 | enzo | 3 | 1.383 | evasa | only heard | 0 | 4 | m | f | 0 | 0.14432989 | 0.024024024 | POST |
| enzo1.3833 | enzo | 3 | 1.383 | striscia | only heard | 0 | 4 | m | f | 0.06862745 | 0.14705882 | 0.00862069 | POST |
| enzo1.3991 | enzo | 1 | 1.399 | strip | only seen | 0 | 5 | m | f | 0.04672897 | 0.18695188 | 0.020114943 | POST |
| enzo1.3991 | enzo | 1 | 1.399 | striscia | 0 | 0 | 5 | m | f | 0.06862745 | 0.14705882 | 0.00862069 | POST |
| enzo1.3991 | enzo | 1 | 1.399 | evasa | 0 | 0 | 5 | m | f | 0 | 0.14432989 | 0.024024024 | POST |
| enzo1.3991 | enzo | 1 | 1.399 | e1m6 | 0 | 0 | 5 | m | f | 0 | 0.16470588 | 0.007092199 | POST |
| enzo1.3991 | enzo | 1 | 1.399 | e2m6 | 0 | 0 | 5 | m | f | 0 | 0.10526315 | 0 | POST |
| enzo1.4053 | enzo | 3 | 1.405 | evasa | seen and heard | 0 | 2 | m | f | 0 | 0.14432989 | 0.024024024 | POST |
| enzo1.4053 | enzo | 3 | 1.405 | sumo | only heard | 1 | 2 | m | m | 0 | 0 | 0.203125 | POST |
| enzo1.4313 | enzo | 3 | 1.431 | strip | only heard | 1 | 1 | m | f | 0.04672897 | 0.18691588 | 0.020114943 | POST |
| enzo1.4983 | enzo | 3 | 1.498 | edera | seen and heard | 0 | 4 | m | f | 0 | 0.133333333 | 0 | POST |
| enzo1.4983 | enzo | 3 | 1.498 | evasa | seen and heard | 0 | 4 | m | f | 0 | 0.14432989 | 0.024024024 | POST |
| enzo1.4983 | enzo | 3 | 1.498 | e2m6 | only heard | 0 | 4 | m | f | 0 | 0.10526315 | 0 | POST |
| enzo1.4983 | enzo | 3 | 1.498 | striscia | only heard | 0 | 4 | m | f | 0.06862745 | 0.14705882 | 0.00862069 | POST |
| enzo1.512 | enzo | 2 | 1.51 | strip | seen and heard | 0 | 3 | m | f | 0.04672897 | 0.18691588 | 0.020114943 | POST |
| enzo1.512 | enzo | 2 | 1.51 | evasa | only heard | 0 | 3 | m | f | 0 | 0.14432989 | 0.024024024 | POST |
| enzo1.512 | enzo | 2 | 1.51 | striscia | only heard | 0 | 3 | m | f | 0.06862745 | 0.14705882 | 0.00862069 | POST |
| enzo1.5422 | enzo | 2 | 1.542 | strip | only heard | 0 | 5 | m | f | 0.04672897 | 0.18691588 | 0.020114943 | POST |
| enzo1.5422 | enzo | 2 | 1.542 | e1m6 | only heard | 0 | 5 | m | f | 0 | 0.16470588 | 0.007092199 | POST |
| enzo1.5483 | enzo | 3 | 1.548 | strip | only seen | 0 | 3 | m | f | 0.04672897 | 0.18691588 | 0.020114943 | POST |
| enzo1.5482 | enzo | 2 | 1.548 | e1m6 | seen and heard | 0 | 6 | m | f | 0 | 0.16470588 | 0.007092199 | POST |
| enzo1.5482 | enzo | 2 | 1.548 | e2m6 | only heard | 0 | 6 | m | f | 0 | 0.10526315 | 0 | POST |
| enzo1.5482 | enzo | 2 | 1.548 | strip | only heard | 0 | 6 | m | f | 0.04672897 | 0.18691588 | 0.020114943 | POST |
| enzo1.5482 | enzo | 2 | 1.548 | striscia | only heard | 0 | 6 | m | f | 0.06862745 | 0.14705882 | 0.00862069 | POST |
| enzo1.5482 | enzo | 2 | 1.548 | evasa | only heard | 0 | 6 | m | f | 0 | 0.14432989 | 0.024024024 | POST |
| enzo1.5482 | enzo | 2 | 1.548 | edera | only heard | 0 | 6 | m | f | 0 | 0.133333333 | 0 | POST |
| enzo1.5483 | enzo | 3 | 1.548 | striscia | 0 | 0 | 3 | m | f | 0.06862745 | 0.14705882 | 0.00862069 | POST |
| enzo1.5483 | enzo | 3 | 1.548 | e1m6 | 0 | 0 | 3 | m | f | 0 | 0.16470588 | 0.007092199 | POST |
| enzo1.5652 | enzo | 2 | 1.565 | evasa | seen and heard | 0 | 5 | m | f | 0 | 0.14432989 | 0.024024024 | POST |
| enzo1.5652 | enzo | 2 | 1.565 | edera | seen and heard | 0 | 5 | m | f | 0 | 0.133333333 | 0 | POST |
| enzo1.5652 | enzo | 2 | 1.565 | e1m6 | seen and heard | 0 | 5 | m | f | 0 | 0.16470588 | 0.007092199 | POST |
| enzo1.5652 | enzo | 2 | 1.565 | e2m6 | only heard | 0 | 5 | m | f | 0 | 0.10526315 | 0 | POST |
| enzo1.5652 | enzo | 2 | 1.565 | striscia | only heard | 0 | 5 | m | f | 0.06862745 | 0.14705882 | 0.00862069 | POST |
| enzo1.5983 | enzo | 3 | 1.598 | striscia | seen and heard | 0 | 2 | m | f | 0.06862745 | 0.14705882 | 0.00862069 | POST |
| enzo1.5983 | enzo | 3 | 1.598 | elly | seen and heard | 0 | 2 | m | f | 0.05882352 | 0.17647058 | 0 | POST |
| enzo1.6233 | enzo | 3 | 1.623 | striscia | seen and heard | 0 | 2 | m | f | 0.06862745 | 0.14705882 | 0.00862069 | POST |
| enzo1.6233 | enzo | 3 | 1.623 | elly | seen and heard | 0 | 2 | m | f | 0.05882352 | 0.17647058 | 0 | POST |
| enzo1.641 | enzo | 1 | 1.64 | evasa | 0 | 0 | 2 | m | f | 0 | 0.14432989 | 0.024024024 | PRE |
| enzo1.641 | enzo | 1 | 1.64 | elly | 0 | 0 | 2 | m | f | 0.05882352 | 0.17647058 | 0 | PRE |
| enzo1.651 | enzo | 1 | 1.65 | strip | seen and heard | 0 | 5 | m | f | 0.04672897 | 0.18691588 | 0.020114943 | PRE |
| enzo1.651 | enzo | 1 | 1.65 | edera | seen and heard | 0 | 5 | m | f | 0 | 0.133333333 | 0 | PRE |
| enzo1.651 | enzo | 1 | 1.65 | e1m6 | seen and heard | 0 | 5 | m | f | 0 | 0.16470588 | 0.007092199 | PRE |
| enzo1.651 | enzo | 1 | 1.65 | evasa | only heard | 0 | 5 | m | f | 0 | 0.14432989 | 0.024024024 | PRE |
| enzo1.651 | enzo | 1 | 1.65 | striscia | only heard | 0 | 5 | m | f | 0.06862745 | 0.14705882 | 0.00862069 | PRE |
| enzo1.6572 | enzo | 2 | 1.657 | strip | seen and heard | 0 | 4 | m | f | 0.04672897 | 0.18691588 | 0.020114943 | PRE |
| enzo1.6572 | enzo | 2 | 1.657 | edera | seen and heard | 0 | 4 | m | f | 0 | 0.133333333 | 0 | PRE |
| enzo1.6572 | enzo | 2 | 1.657 | evasa | only heard | 0 | 4 | m | f | 0 | 0.14432989 | 0.024024024 | PRE |
| enzo1.6572 | enzo | 2 | 1.657 | striscia | only heard | 0 | 4 | m | f | 0.06862745 | 0.14705882 | 0.00862069 | PRE |
| enzo1.6693 | enzo | 3 | 1.669 | e1m6 | seen and heard | 0 | 1 | m | f | 0 | 0.16470588 | 0.007092199 | PRE |
| enzo1.8042 | enzo | 2 | 1.804 | striscia | only heard | 1 | 2 | m | f | 0.06862745 | 0.14705882 | 0.00862069 | PRE |
| enzo1.8042 | enzo | 2 | 1.804 | edera | only heard | 0 | 2 | m | f | 0 | 0.133333333 | 0 | PRE |
| enzo1.8062 | enzo | 2 | 1.806 | strip | seen and heard | 0 | 4 | m | f | 0.04672897 | 0.18691588 | 0.020114943 | PRE |
| enzo1.8062 | enzo | 2 | 1.806 | striscia | only heard | 0 | 4 | m | f | 0.06862745 | 0.14705882 | 0.00862069 | PRE |
| enzo1.8062 | enzo | 2 | 1.806 | evasa | only heard | 0 | 4 | m | f | 0 | 0.14432989 | 0.024024024 | PRE |
| enzo1.8062 | enzo | 2 | 1.806 | edera | only heard | 0 | 4 | m | f | 0 | 0.133333333 | 0 | PRE |
| enzo1.8151 | enzo | 1 | 1.815 | sumo | 0 | 0 | 1 | m | m | 0 | 0 | 0.203125 | PRE |
| enzo1.842 | enzo | 2 | 1.84 | evasa | only seen | 0 | 4 | m | f | 0 | 0.14432989 | 0.024024024 | PRE |
| enzo1.842 | enzo | 2 | 1.84 | edera | 0 | 0 | 4 | m | f | 0 | 0.133333333 | 0 | PRE |
| enzo1.842 | enzo | 2 | 1.84 | elly | 0 | 0 | 4 | m | f | 0.05882352 | 0.17647058 | 0 | PRE |
| enzo1.842 | enzo | 2 | 1.84 | e2m6 | 0 | 0 | 4 | m | f | 0 | 0.10526315 | 0 | PRE |
| enzo1.93 | enzo | 3 | 1.9 | evasa | seen and heard | 0 | 12 | m | f | 0 | 0.14432989 | 0.024024024 | PRE |
| enzo1.93 | enzo | 3 | 1.9 | strip | seen and heard | 0 | 12 | m | f | 0.04672897 | 0.18691588 | 0.020114943 | PRE |
| enzo1.93 | enzo | 3 | 1.9 | sguercia | seen and heard | 0 | 12 | m | f | 0 | 0 | 0 | PRE |
| enzo1.93 | enzo | 3 | 1.9 | severa | seen and heard | 0 | 12 | m | f | 0 | 0 | 0.066176471 | PRE |
| enzo1.93 | enzo | 3 | 1.9 | sfasciato | seen and heard | 0 | 12 | m | m | 0 | 0 | 0.024390244 | PRE |
| enzo1.93 | enzo | 3 | 1.9 | sonia | seen and heard | 0 | 12 | m | f | 0 | 0 | 0.041666667 | PRE |
| enzo1.93 | enzo | 3 | 1.9 | neomamma | seen and heard | 0 | 12 | m | f | 0 | 0 | 0.032840722 | PRE |
| enzo1.93 | enzo | 3 | 1.9 | schiarita | seen and heard | 0 | 12 | m | f | 0 | 0 | 0.041420118 | PRE |
| enzo1.93 | enzo | 3 | 1.9 | spiga | only heard | 0 | 12 | m | f | 0 | 0 | 0.018372703 | PRE |
| enzo1.93 | enzo | 3 | 1.9 | susy | only heard | 0 | 12 | m | f | 0 | 0 | 0.027777778 | PRE |
| enzo1.93 | enzo | 3 | 1.9 | sorcia | only heard | 0 | 12 | m | f | 0 | 0 | 0.022727273 | PRE |
| enzo1.93 | enzo | 3 | 1.9 | sine | only heard | 0 | 12 | m | f | 0 | 0 | 0.050682261 | PRE |
| enzo1.9183 | enzo | 3 | 1.918 | strip | seen and heard | 0 | 6 | m | f | 0.04672897 | 0.18691588 | 0.020114943 | PRE |
| enzo1.9183 | enzo | 3 | 1.918 | striscia | seen and heard | 0 | 6 | m | f | 0.06862745 | 0.14705882 | 0.00862069 | PRE |
| enzo1.9183 | enzo | 3 | 1.918 | evasa | only heard | 0 | 6 | m | f | 0 | 0.14432989 | 0.024024024 | PRE |
| enzo1.9183 | enzo | 3 | 1.918 | edera | only heard | 0 | 6 | m | f | 0 | 0.133333333 | 0 | PRE |
| enzo1.9183 | enzo | 3 | 1.918 | e1m6 | only heard | 0 | 6 | m | f | 0 | 0.16470588 | 0.007092199 | PRE |
| enzo1.9183 | enzo | 3 | 1.918 | e2m6 | only heard | 0 | 6 | m | f | 0 | 0.10526315 | 0 | PRE |
| enzo2.0362 | enzo | 2 | 2.036 | edera | 0 | 0 | 7 | m | f | 0 | 0.133333333 | 0 | PRE |
| enzo2.0362 | enzo | 2 | 2.036 | e1m6 | 0 | 0 | 7 | m | f | 0 | 0.16470588 | 0.007092199 | PRE |
| enzo2.0362 | enzo | 2 | 2.036 | e2m6 | 0 | 0 | 7 | m | f | 0 | 0.10526315 | 0 | PRE |
| enzo2.0362 | enzo | 2 | 2.036 | evasa | 0 | 0 | 7 | m | f | 0 | 0.14432989 | 0.024024024 | PRE |
| enzo2.0362 | enzo | 2 | 2.036 | striscia | 0 | 0 | 7 | m | f | 0.06862745 | 0.14705882 | 0.00862069 | PRE |
| enzo2.0362 | enzo | 2 | 2.036 | strip | 0 | 0 | 7 | m | f | 0.04672897 | 0.18691588 | 0.020114943 | PRE |
| enzo2.0362 | enzo | 2 | 2.036 | elly | 0 | 0 | 7 | m | f | 0.05882352 | 0.17647058 | 0 | PRE |
| enzo2.13 | enzo | 3 | 2.1 | strip | only heard | 0 | 3 | m | f | 0.04672897 | 0.18691588 | 0.020114943 | PRE |
| enzo2.13 | enzo | 3 | 2.1 | e1m6 | only heard | 0 | 3 | m | f | 0 | 0.16470588 | 0.007092199 | PRE |
| enzo2.13 | enzo | 3 | 2.1 | evasa | only heard | 0 | 3 | m | f | 0 | 0.14432989 | 0.024024024 | PRE |
| enzo2.1373 | enzo | 3 | 2.137 | strip | only seen | 0 | 3 | m | f | 0.04672897 | 0.18691588 | 0.020114943 | PRE |
| enzo2.1373 | enzo | 3 | 2.137 | elly | 0 | 0 | 3 | m | f | 0.05882352 | 0.17647058 | 0 | PRE |
| enzo2.1373 | enzo | 3 | 2.137 | striscia | 0 | 0 | 3 | m | f | 0.06862745 | 0.14705882 | 0.00862069 | PRE |
| enzo2.163 | enzo | 3 | 2.16 | evasa | 0 | 0 | 6 | m | f | 0 | 0.14432989 | 0.024024024 | PRE |
| enzo2.163 | enzo | 3 | 2.16 | strip | 0 | 0 | 6 | m | f | 0.04672897 | 0.18691588 | 0.020114943 | PRE |
| enzo2.163 | enzo | 3 | 2.16 | striscia | 0 | 0 | 6 | m | f | 0.06862745 | 0.14705882 | 0.00862069 | PRE |
| enzo2.163 | enzo | 3 | 2.16 | edera | 0 | 0 | 6 | m | f | 0 | 0.133333333 | 0 | PRE |
| enzo2.163 | enzo | 3 | 2.16 | e1m6 | 0 | 0 | 6 | m | f | 0 | 0.16470588 | 0.007092199 | PRE |
| enzo2.163 | enzo | 3 | 2.16 | e2m6 | 0 | 0 | 6 | m | f | 0 | 0.10526315 | 0 | PRE |
| enzo2.183 | enzo | 3 | 2.18 | strip | only heard | 0 | 5 | m | f | 0.04672897 | 0.18691588 | 0.020114943 | PRE |
| enzo2.183 | enzo | 3 | 2.18 | striscia | only heard | 0 | 5 | m | f | 0.06862745 | 0.14705882 | 0.00862069 | PRE |
| enzo2.183 | enzo | 3 | 2.18 | e2m6 | only heard | 0 | 5 | m | f | 0 | 0.10526315 | 0 | PRE |
| enzo2.183 | enzo | 3 | 2.18 | e1m6 | only heard | 0 | 5 | m | f | 0 | 0.16470588 | 0.007092199 | PRE |
| enzo2.183 | enzo | 3 | 2.18 | edera | only heard | 0 | 5 | m | f | 0 | 0.133333333 | 0 | PRE |
| enzo2.253 | enzo | 3 | 2.25 | striscia | only seen | 0 | 3 | m | f | 0.06862745 | 0.14705882 | 0.00862069 | PRE |
| enzo2.253 | enzo | 3 | 2.25 | elly | 0 | 0 | 3 | m | f | 0.05882352 | 0.17647058 | 0 | PRE |
| enzo2.2663 | enzo | 3 | 2.266 | strip | seen and heard | 0 | 3 | m | f | 0.04672897 | 0.18691588 | 0.020114943 | PRE |
| enzo2.2663 | enzo | 3 | 2.266 | elly | only heard | 0 | 3 | m | f | 0.05882352 | 0.17647058 | 0 | PRE |
| enzo2.2663 | enzo | 3 | 2.266 | striscia | only heard | 0 | 3 | m | f | 0.06862745 | 0.14705882 | 0.00862069 | PRE |
| enzo2.3183 | enzo | 3 | 2.318 | evasa | seen and heard | 0 | 4 | m | f | 0 | 0.14432989 | 0.024024024 | PRE |
| enzo2.3183 | enzo | 3 | 2.318 | edera | only heard | 0 | 4 | m | f | 0 | 0.133333333 | 0 | PRE |
| enzo2.3183 | enzo | 3 | 2.318 | elly | only heard | 0 | 4 | m | f | 0.05882352 | 0.17647058 | 0 | PRE |
| enzo2.3183 | enzo | 3 | 2.318 | e2m6 | only heard | 0 | 4 | m | f | 0 | 0.10526315 | 0 | PRE |
| enzo2.3553 | enzo | 3 | 2.355 | elly | seen and heard | 0 | 4 | m | f | 0.05882352 | 0.17647058 | 0 | PRE |
| enzo2.3553 | enzo | 3 | 2.355 | evasa | seen and heard | 0 | 4 | m | f | 0 | 0.14432989 | 0.024024024 | PRE |
| enzo2.3553 | enzo | 3 | 2.355 | e2m6 | 0 | 0 | 4 | m | f | 0 | 0.10526315 | 0 | PRE |
| enzo2.3553 | enzo | 3 | 2.355 | edera | 0 | 0 | 4 | m | f | 0 | 0.133333333 | 0 | PRE |
| enzo2.653 | enzo | 3 | 2.65 | sguercia | only seen | 0 | 11 | m | f | 0 | 0 | 0 | PRE |
| enzo2.653 | enzo | 3 | 2.65 | severa | only seen | 0 | 11 | m | f | 0 | 0 | 0.066176471 | PRE |
| enzo2.653 | enzo | 3 | 2.65 | schiarita | only seen | 0 | 11 | m | f | 0 | 0 | 0.041420118 | PRE |
| enzo2.653 | enzo | 3 | 2.65 | sumo | only seen | 1 | 11 | m | m | 0 | 0 | 0.203125 | PRE |
| enzo2.653 | enzo | 3 | 2.65 | spilla | only seen | 0 | 11 | m | f | 0 | 0 | 0.043678161 | PRE |
| enzo2.653 | enzo | 3 | 2.65 | strip | only seen | 0 | 11 | m | f | 0.04672897 | 0.18691588 | 0.020114943 | PRE |
| enzo2.653 | enzo | 3 | 2.65 | susy | 0 | 0 | 11 | m | f | 0 | 0 | 0.027777778 | PRE |
| enzo2.653 | enzo | 3 | 2.65 | neomamma | 0 | 0 | 11 | m | f | 0 | 0 | 0.032840722 | PRE |
| enzo2.653 | enzo | 3 | 2.65 | sfasciato | 0 | 0 | 11 | m | m | 0 | 0 | 0.024390244 | PRE |
| enzo2.653 | enzo | 3 | 2.65 | sonia | 0 | 0 | 11 | m | f | 0 | 0 | 0.041666667 | PRE |
| enzo2.653 | enzo | 3 | 2.65 | sine | 0 | 0 | 11 | m | f | 0 | 0 | 0.050682261 | PRE |
| enzo2.713 | enzo | 3 | 2.71 | strega | only seen | 0 | 10 | m | f | 0 | 0 | 0.016025641 | PRE |
| enzo2.713 | enzo | 3 | 2.71 | sumo | only seen | 0 | 10 | m | m | 0 | 0 | 0.203125 | PRE |
| enzo2.713 | enzo | 3 | 2.71 | severa | 0 | 0 | 10 | m | f | 0 | 0 | 0.066176471 | PRE |
| enzo2.713 | enzo | 3 | 2.71 | neomamma | 0 | 0 | 10 | m | f | 0 | 0 | 0.032840722 | PRE |
| enzo2.713 | enzo | 3 | 2.71 | spiga | 0 | 0 | 10 | m | f | 0 | 0 | 0.018372703 | PRE |
| enzo2.713 | enzo | 3 | 2.71 | sfasciato | 0 | 0 | 10 | m | m | 0 | 0 | 0.024390244 | PRE |
| enzo2.713 | enzo | 3 | 2.71 | susy | 0 | 0 | 10 | m | f | 0 | 0 | 0.027777778 | PRE |
| enzo2.713 | enzo | 3 | 2.71 | sonia | 0 | 0 | 10 | m | f | 0 | 0 | 0.041666667 | PRE |
| enzo2.713 | enzo | 3 | 2.71 | sorcia | 0 | 0 | 10 | m | f | 0 | 0 | 0.022727273 | PRE |
| enzo2.713 | enzo | 3 | 2.71 | sguercia | 0 | 0 | 10 | m | f | 0 | 0 | 0 | PRE |
| enzo2.7153 | enzo | 3 | 2.715 | evasa | only heard | 0 | 4 | m | f | 0 | 0.14432989 | 0.024024024 | PRE |
| enzo2.7153 | enzo | 3 | 2.715 | e2m6 | only heard | 0 | 4 | m | f | 0 | 0.10526315 | 0 | PRE |
| enzo2.7153 | enzo | 3 | 2.715 | striscia | only heard | 0 | 4 | m | f | 0.06862745 | 0.14705882 | 0.00862069 | PRE |
| enzo2.7153 | enzo | 3 | 2.715 | edera | only heard | 0 | 4 | m | f | 0 | 0.133333333 | 0 | PRE |
| enzo3.0393 | enzo | 3 | 3.039 | elly | seen and heard | 1 | 4 | m | f | 0.05882352 | 0.17647058 | 0 | PRE |
| enzo3.0393 | enzo | 3 | 3.039 | edera | seen and heard | 0 | 4 | m | f | 0 | 0.133333333 | 0 | PRE |
| enzo3.0393 | enzo | 3 | 3.039 | evasa | seen and heard | 0 | 4 | m | f | 0 | 0.14432989 | 0.024024024 | PRE |
| enzo3.0393 | enzo | 3 | 3.039 | e2m6 | only heard | 0 | 4 | m | f | 0 | 0.10526315 | 0 | PRE |
| evasa1.2321 | evasa | 1 | 1.232 | enzo | seen and heard | 1 | 5 | f | m | 0 | 0.14432989 | 0.181102362 | POST |
| evasa1.2321 | evasa | 1 | 1.232 | strip | seen and heard | 0 | 5 | f | f | 0 | 0.07216494 | 0.020114943 | POST |
| evasa1.2321 | evasa | 1 | 1.232 | e1m6 | seen and heard | 0 | 5 | f | f | 0.03614457 | 0.12048192 | 0.007092199 | POST |
| evasa1.3011 | evasa | 1 | 1.301 | e1m6 | only seen | 0 | 2 | f | f | 0.03614457 | 0.12048192 | 0.007092199 | POST |
| evasa1.3011 | evasa | 1 | 1.301 | e2m6 | only seen | 1 | 2 | f | f | 0 | 0.05633802 | 0 | POST |
| evasa1.5031 | evasa | 1 | 1.503 | enzo | seen and heard | 0 | 3 | f | m | 0 | 0.14432989 | 0.181102362 | POST |
| evasa1.5031 | evasa | 1 | 1.503 | striscia | seen and heard | 0 | 3 | f | f | 0.06185567 | 0.14432989 | 0.00862069 | POST |
| evasa1.5031 | evasa | 1 | 1.503 | strip | only heard | 0 | 3 | f | f | 0 | 0.07216494 | 0.020114943 | POST |
| evasa1.691 | evasa | 1 | 1.69 | striscia | seen and heard | 0 | 1 | f | f | 0.06185567 | 0.14432989 | 0.00862069 | POST |
| evasa1.8152 | evasa | 2 | 1.815 | striscia | only seen | 0 | 1 | f | f | 0.06185567 | 0.14432989 | 0.00862069 | POST |
| evasa1.8881 | evasa | 1 | 1.888 | enzo | only seen | 1 | 5 | f | m | 0 | 0.14432989 | 0.181102362 | POST |
| evasa1.8881 | evasa | 1 | 1.888 | e2m6 | only seen | 1 | 5 | f | f | 0 | 0.05633802 | 0 | POST |
| evasa1.8881 | evasa | 1 | 1.888 | e1m6 | 0 | 0 | 5 | f | f | 0.03614457 | 0.12048192 | 0.007092199 | POST |
| evasa1.8881 | evasa | 1 | 1.888 | strip | 0 | 0 | 5 | f | f | 0 | 0.07216494 | 0.020114943 | PRE |
| evasa1.8881 | evasa | 1 | 1.888 | striscia | 0 | 0 | 5 | f | f | 0.06185567 | 0.14432989 | 0.00862069 | PRE |
| evasa1.9841 | evasa | 1 | 1.984 | strip | 0 | 0 | 5 | f | f | 0 | 0.07216494 | 0.020114943 | PRE |
| evasa1.9841 | evasa | 1 | 1.984 | striscia | 0 | 0 | 5 | f | f | 0.06185567 | 0.14432989 | 0.00862069 | PRE |
| evasa1.9841 | evasa | 1 | 1.984 | e1m6 | 0 | 0 | 5 | f | f | 0.03614457 | 0.12048192 | 0.007092199 | PRE |
| evasa1.9841 | evasa | 1 | 1.984 | e2m6 | 0 | 0 | 5 | f | f | 0 | 0.05633802 | 0 | PRE |
| evasa1.9841 | evasa | 1 | 1.984 | enzo | 0 | 0 | 5 | f | m | 0 | 0.14432989 | 0.181102362 | PRE |
| evasa2.0821 | evasa | 1 | 2.082 | striscia | only seen | 0 | 2 | f | f | 0.06185567 | 0.14432989 | 0.00862069 | PRE |
| evasa2.0821 | evasa | 1 | 2.082 | e1m6 | only seen | 1 | 2 | f | f | 0.03614457 | 0.12048192 | 0.007092199 | PRE |
| evasa2.5491 | evasa | 1 | 2.549 | edera | only heard | 0 | 2 | f | f | 0.04054054 | 0.14864864 | 0 | PRE |
| evasa2.5491 | evasa | 1 | 2.549 | enzo | only heard | 0 | 2 | f | m | 0 | 0.14432989 | 0.181102362 | PRE |
| evasa2.6483 | evasa | 3 | 2.648 | e1m6 | only seen | 0 | 2 | f | f | 0.03614457 | 0.12048192 | 0.007092199 | PRE |
| evasa2.6483 | evasa | 3 | 2.648 | e2m6 | only seen | 1 | 2 | f | f | 0 | 0.05633802 | 0 | PRE |
| evasa3.4911 | evasa | 1 | 3.491 | e1m6 | only seen | 0 | 3 | f | f | 0.03614457 | 0.12048192 | 0.007092199 | PRE |
| evasa3.4911 | evasa | 1 | 3.491 | e2m6 | 0 | 0 | 3 | f | f | 0 | 0.05633802 | 0 | PRE |
| evasa3.4911 | evasa | 1 | 3.491 | strip | 0 | 0 | 3 | f | f | 0 | 0.07216494 | 0.020114943 | PRE |
| evasa3.6191 | evasa | 1 | 3.619 | striscia | 0 | 0 | 1 | f | f | 0.06185567 | 0.14432989 | 0.00862069 | PRE |
| gatta1.321 | gatta | 1 | 1.32 | grappolo | only seen | 1 | 3 | f | f | 0.022727273 | 0.159090909 | 0.05764411 | POST |
| gatta1.321 | gatta | 1 | 1.32 | gianna | 0 | 0 | 3 | f | f | 0.04651162 | 0.13953488 | 0.03163017 | POST |
| gatta1.321 | gatta | 1 | 1.32 | giangi | 0 | 0 | 3 | f | m | 0 | 0.05660371 | 0.215686275 | POST |
| gatta1.971 | gatta | 1 | 1.97 | giangi | only heard | 0 | 6 | f | m | 0 | 0.05660371 | 0.215686275 | POST |
| gatta1.971 | gatta | 1 | 1.97 | gianna | only heard | 0 | 6 | f | f | 0.04651162 | 0.13953488 | 0.03163017 | POST |
| gatta1.971 | gatta | 1 | 1.97 | grappolo | only heard | 0 | 6 | f | f | 0.022727273 | 0.159090909 | 0.05764411 | POST |
| gatta1.971 | gatta | 1 | 1.97 | gigetto | only heard | 0 | 6 | f | m | 0.52272727 | 0.0681818 | 0.039548023 | POST |
| gatta1.971 | gatta | 1 | 1.97 | gessica | only heard | 0 | 6 | f | f | 0.011111111 | 0.166666667 | 0.039735099 | POST |
| gatta1.971 | gatta | 1 | 1.97 | ghiozza | seen and heard | 0 | 6 | f | f | 0.14457831 | 0.18072289 | 0.017094017 | POST |
| gatta2.181 | gatta | 1 | 2.18 | gianna | only seen | 0 | 4 | f | f | 0.04651162 | 0.13953488 | 0.03163017 | POST |
| gatta2.181 | gatta | 1 | 2.18 | ghirlanda | only seen | 0 | 4 | f | f | 0.1125 | 0.175 | 0.023148148 | POST |
| gatta2.181 | gatta | 1 | 2.18 | giangi | only seen | 1 | 4 | f | m | 0 | 0.05660371 | 0.215686275 | POST |
| gatta2.181 | gatta | 1 | 2.18 | gessica | 0 | 0 | 4 | f | f | 0.011111111 | 0.166666667 | 0.039735099 | POST |
| gatta2.312 | gatta | 2 | 2.31 | ghiozza | only heard | 1 | 4 | f | f | 0.14457831 | 0.18072289 | 0.017094017 | POST |
| gatta2.312 | gatta | 2 | 2.31 | grappolo | only heard | 0 | 4 | f | f | 0.022727273 | 0.159090909 | 0.05764411 | POST |
| gatta2.51 | gatta | 1 | 2.5 | gessica | 0 | 0 | 2 | f | f | 0.011111111 | 0.166666667 | 0.039735099 | POST |
| gatta2.51 | gatta | 1 | 2.5 | gianna | 0 | 0 | 2 | f | f | 0.04651162 | 0.13953488 | 0.03163017 | POST |
| gatta2.631 | gatta | 1 | 2.63 | giangi | 0 | 0 | 4 | f | m | 0 | 0.05660371 | 0.215686275 | POST |
| gatta2.631 | gatta | 1 | 2.63 | gessica | 0 | 0 | 4 | f | f | 0.011111111 | 0.166666667 | 0.039735099 | POST |
| gatta2.631 | gatta | 1 | 2.63 | gianna | 0 | 0 | 4 | f | f | 0.04651162 | 0.13953488 | 0.03163017 | POST |
| gatta2.631 | gatta | 1 | 2.63 | ghirlanda | 0 | 0 | 4 | f | f | 0.1125 | 0.175 | 0.023148148 | POST |
| gatta2.641 | gatta | 1 | 2.64 | gigio | only seen | 0 | 3 | f | m | 0.0625 | 0.03125 | 0 | POST |
| gatta2.641 | gatta | 1 | 2.64 | ghirlanda | only seen | 0 | 3 | f | f | 0.1125 | 0.175 | 0.023148148 | POST |
| gatta2.641 | gatta | 1 | 2.64 | grappolo | 0 | 0 | 3 | f | f | 0.022727273 | 0.159090909 | 0.05764411 | POST |
| gatta3.893 | gatta | 3 | 3.89 | giangi | only heard | 0 | 1 | f | m | 0 | 0.05660371 | 0.215686275 | POST |
| gessica0.85* | gessica | * | 0.85 | giangi | 0 | 0 | 1 | f | m | 0.2189781 | 0.1094895 | 0.215686275 | POST |
| gessica1.241 | gessica | 1 | 1.24 | gatta | only seen | 0 | 4 | f | f | 0.011111111 | 0.166666667 | 0.016528926 | POST |
| gessica1.241 | gessica | 1 | 1.24 | ghiozza | 0 | 0 | 4 | f | f | 0 | 0.12328767 | 0.017094017 | POST |
| gessica1.241 | gessica | 1 | 1.24 | giangi | 0 | 0 | 4 | f | m | 0.2189781 | 0.1094895 | 0.215686275 | POST |
| gessica1.241 | gessica | 1 | 1.24 | grappolo | 0 | 0 | 4 | f | f | 0 | 0.15384615 | 0.05764411 | POST |
| gessica1.333 | gessica | 3 | 1.33 | giangi | 0 | 0 | 1 | f | m | 0.2189781 | 0.1094895 | 0.215686275 | POST |
| gessica1.561 | gessica | 1 | 1.56 | ghiozza | 0 | 0 | 2 | f | f | 0 | 0.12328767 | 0.017094017 | POST |
| gessica1.561 | gessica | 1 | 1.56 | ghirlanda | 0 | 0 | 2 | f | f | 0.01980198 | 0.07920792 | 0.023148148 | POST |
| gessica1.571 | gessica | 1 | 1.57 | giangi | only seen | 0 | 6 | f | m | 0.2189781 | 0.1094895 | 0.215686275 | POST |
| gessica1.571 | gessica | 1 | 1.57 | ghirlanda | only seen | 0 | 6 | f | f | 0.01980198 | 0.07920792 | 0.023148148 | POST |
| gessica1.571 | gessica | 1 | 1.57 | gianna | 0 | 0 | 6 | f | f | 0.01063829 | 0.23404255 | 0.03163017 | POST |
| gessica1.571 | gessica | 1 | 1.57 | ghiozza | 0 | 0 | 6 | f | f | 0 | 0.12328767 | 0.017094017 | POST |
| gessica1.571 | gessica | 1 | 1.57 | gatta | 0 | 0 | 6 | f | f | 0.011111111 | 0.166666667 | 0.016528926 | POST |
| gessica1.633 | gessica | 3 | 1.63 | gianna | only seen | 1 | 4 | f | f | 0.01063829 | 0.23404255 | 0.03163017 | POST |
| gessica1.633 | gessica | 3 | 1.63 | ghirlanda | 0 | 0 | 4 | f | f | 0.01980198 | 0.07920792 | 0.023148148 | POST |
| gessica1.633 | gessica | 3 | 1.63 | giangi | 0 | 0 | 4 | f | m | 0.2189781 | 0.1094895 | 0.215686275 | POST |
| gessica1.633 | gessica | 3 | 1.63 | gatta | 0 | 0 | 4 | f | f | 0.011111111 | 0.166666667 | 0.016528926 | POST |
| gessica1.73 | gessica | 3 | 1.7 | giangi | 0 | 0 | 1 | f | m | 0.2189781 | 0.1094895 | 0.215686275 | POST |
| gessica1.761 | gessica | 1 | 1.76 | gatta | only seen | 0 | 4 | f | f | 0.011111111 | 0.166666667 | 0.016528926 | POST |
| gessica1.761 | gessica | 1 | 1.76 | ghirlanda | only seen | 0 | 4 | f | f | 0.01980198 | 0.07920792 | 0.023148148 | POST |
| gessica1.761 | gessica | 1 | 1.76 | grappolo | 0 | 0 | 4 | f | f | 0 | 0.15384615 | 0.05764411 | POST |
| gessica1.761 | gessica | 1 | 1.76 | giangi | 0 | 0 | 4 | f | m | 0.2189781 | 0.1094895 | 0.215686275 | POST |
| gessica1.851 | gessica | 1 | 1.85 | giangi | only seen | 0 | 4 | f | m | 0.2189781 | 0.1094895 | 0.215686275 | POST |
| gessica1.851 | gessica | 1 | 1.85 | gianna | only seen | 1 | 4 | f | f | 0.01063829 | 0.23404255 | 0.03163017 | POST |
| gessica1.851 | gessica | 1 | 1.85 | grappolo | only seen | 0 | 4 | f | f | 0 | 0.15384615 | 0.05764411 | POST |
| gessica1.851 | gessica | 1 | 1.85 | ghirlanda | only seen | 0 | 4 | f | f | 0.01980198 | 0.07920792 | 0.023148148 | POST |
| gessica1.853 | gessica | 3 | 1.85 | giangi | 0 | 0 | 1 | f | m | 0.2189781 | 0.1094895 | 0.215686275 | POST |
| gessica1.9351 | gessica | 1 | 1.935 | giangi | only seen | 1 | 7 | f | m | 0.2189781 | 0.1094895 | 0.215686275 | POST |
| gessica1.9351 | gessica | 1 | 1.935 | grappolo | only seen | 1 | 7 | f | f | 0 | 0.15384615 | 0.05764411 | POST |
| gessica1.9351 | gessica | 1 | 1.935 | gigio | 0 | 0 | 7 | f | m | 0 | 0 | 0 | POST |
| gessica1.9351 | gessica | 1 | 1.935 | gigetto | 0 | 0 | 7 | f | m | 0.06060606 | 0 | 0.039548023 | POST |
| gessica1.9351 | gessica | 1 | 1.935 | gianna | 0 | 0 | 7 | f | f | 0.01063829 | 0.23404255 | 0.03163017 | POST |
| gessica1.9351 | gessica | 1 | 1.935 | ghirlanda | 0 | 0 | 7 | f | f | 0.01980198 | 0.07920792 | 0.023148148 | POST |
| gessica1.9351 | gessica | 1 | 1.935 | gatta | 0 | 0 | 7 | f | f | 0.011111111 | 0.166666667 | 0.016528926 | POST |
| gessica2.061 | gessica | 1 | 2.06 | gatta | only seen | 0 | 5 | f | f | 0.011111111 | 0.166666667 | 0.016528926 | POST |
| gessica2.061 | gessica | 1 | 2.06 | giangi | 0 | 0 | 5 | f | m | 0.2189781 | 0.1094895 | 0.215686275 | POST |
| gessica2.061 | gessica | 1 | 2.06 | gianna | 0 | 0 | 5 | f | f | 0.01063829 | 0.23404255 | 0.03163017 | POST |
| gessica2.061 | gessica | 1 | 2.06 | grappolo | 0 | 0 | 5 | f | f | 0 | 0.15384615 | 0.05764411 | PRE |
| gessica2.061 | gessica | 1 | 2.06 | ghirlanda | 0 | 0 | 5 | f | f | 0.01980198 | 0.07920792 | 0.023148148 | PRE |
| gessica2.383 | gessica | 3 | 2.38 | giangi | only seen | 1 | 2 | f | m | 0.2189781 | 0.1094895 | 0.215686275 | PRE |
| gessica2.383 | gessica | 3 | 2.38 | ghiozza | 0 | 0 | 2 | f | f | 0 | 0.12328767 | 0.017094017 | PRE |
| gessica2.8953 | gessica | 3 | 2.895 | giangi | 0 | 0 | 3 | f | m | 0.2189781 | 0.1094895 | 0.215686275 | PRE |
| gessica2.8953 | gessica | 3 | 2.895 | gatta | 0 | 0 | 3 | f | f | 0.011111111 | 0.166666667 | 0.016528926 | PRE |
| gessica2.8953 | gessica | 3 | 2.895 | ghiozza | 0 | 0 | 3 | f | f | 0 | 0.12328767 | 0.017094017 | PRE |
| gessica3.021 | gessica | 1 | 3.02 | giangi | only heard | 0 | 6 | f | m | 0.2189781 | 0.1094895 | 0.215686275 | PRE |
| gessica3.943 | gessica | 3 | 3.94 | giangi | 0 | 0 | 4 | f | m | 0.2189781 | 0.1094895 | 0.215686275 | PRE |
| gessica3.943 | gessica | 3 | 3.94 | grappolo | 0 | 0 | 4 | f | f | 0 | 0.15384615 | 0.05764411 | PRE |
| gessica3.943 | gessica | 3 | 3.94 | gianna | 0 | 0 | 4 | f | f | 0.01063829 | 0.23404255 | 0.03163017 | PRE |
| gessica3.943 | gessica | 3 | 3.94 | ghirlanda | 0 | 0 | 4 | f | f | 0.01980198 | 0.07920792 | 0.023148148 | PRE |
| ghiozza1.741 | ghiozza | 1 | 1.74 | ghirlanda | only seen | 0 | 3 | f | f | 0.066666667 | 0.026666667 | 0.023148148 | POST |
| ghiozza1.741 | ghiozza | 1 | 1.74 | giangi | 0 | 0 | 3 | f | m | 0 | 0.07920792 | 0.215686275 | POST |
| ghiozza1.741 | ghiozza | 1 | 1.74 | gessica | 0 | 0 | 3 | f | f | 0 | 0.12328767 | 0.039735099 | POST |
| ghiozza1.781 | ghiozza | 1 | 1.78 | grappolo | seen and heard | 0 | 5 | f | f | 0.11764705 | 0.11764705 | 0.05764411 | POST |
| ghiozza1.781 | ghiozza | 1 | 1.78 | giangi | only heard | 0 | 5 | f | m | 0 | 0.07920792 | 0.215686275 | POST |
| ghiozza1.781 | ghiozza | 1 | 1.78 | gessica | only heard | 0 | 5 | f | f | 0 | 0.12328767 | 0.039735099 | POST |
| ghiozza1.781 | ghiozza | 1 | 1.78 | ghirlanda | only heard | 0 | 5 | f | f | 0.066666667 | 0.026666667 | 0.023148148 | POST |
| ghiozza1.781 | ghiozza | 1 | 1.78 | gianna | only heard | 0 | 5 | f | f | 0.09523805 | 0.1547619 | 0.03163017 | POST |
| ghiozza2.321 | ghiozza | 1 | 2.32 | gatta | only seen | 0 | 3 | f | f | 0.14457831 | 0.18072289 | 0.016528926 | POST |
| ghiozza2.321 | ghiozza | 1 | 2.32 | gigetto | 0 | 0 | 3 | f | m | 0.15 | 0.125 | 0.039548023 | POST |
| ghiozza2.321 | ghiozza | 1 | 2.32 | gianna | 0 | 0 | 3 | f | f | 0.09523805 | 0.1547619 | 0.03163017 | POST |
| ghiozza2.381 | ghiozza | 1 | 2.38 | grappolo | only seen | 0 | 3 | f | f | 0.11764705 | 0.11764705 | 0.05764411 | POST |
| ghiozza2.381 | ghiozza | 1 | 2.38 | gatta | 0 | 0 | 3 | f | f | 0.14457831 | 0.18072289 | 0.016528926 | POST |
| ghiozza2.381 | ghiozza | 1 | 2.38 | gessica | 0 | 0 | 3 | f | f | 0 | 0.12328767 | 0.039735099 | POST |
| ghiozza2.51 | ghiozza | 1 | 2.5 | gianna | only seen | 0 | 4 | f | f | 0.09523805 | 0.1547619 | 0.03163017 | POST |
| ghiozza2.51 | ghiozza | 1 | 2.5 | giangi | 0 | 0 | 4 | f | m | 0 | 0.07920792 | 0.215686275 | POST |
| ghiozza2.51 | ghiozza | 1 | 2.5 | ghirlanda | 0 | 0 | 4 | f | f | 0.066666667 | 0.026666667 | 0.023148148 | POST |
| ghiozza2.51 | ghiozza | 1 | 2.5 | gatta | 0 | 0 | 4 | f | f | 0.14457831 | 0.18072289 | 0.016528926 | POST |
| ghiozza4.263 | ghiozza | 3 | 4.26 | grappolo | only seen | 0 | 5 | f | f | 0.11764705 | 0.11764705 | 0.05764411 | POST |
| ghiozza4.263 | ghiozza | 3 | 4.26 | gessica | only seen | 0 | 5 | f | f | 0 | 0.12328767 | 0.039735099 | POST |
| ghiozza4.263 | ghiozza | 3 | 4.26 | gianna | only seen | 0 | 5 | f | f | 0.09523805 | 0.1547619 | 0.03163017 | POST |
| ghiozza4.263 | ghiozza | 3 | 4.26 | ghirlanda | 0 | 0 | 5 | f | f | 0.066666667 | 0.026666667 | 0.023148148 | POST |
| ghiozza4.263 | ghiozza | 3 | 4.26 | gatta | 0 | 0 | 5 | f | f | 0.14457831 | 0.18072289 | 0.016528926 | POST |
| ghirlanda1.533 | ghirlanda | 3 | 1.53 | gatta | only seen | 1 | 4 | f | f | 0.1125 | 0.175 | 0.016528926 | POST |
| ghirlanda1.533 | ghirlanda | 3 | 1.53 | gigio | 0 | 0 | 4 | f | m | 0 | 0.17857142 | 0 | POST |
| ghirlanda1.533 | ghirlanda | 3 | 1.53 | grappolo | 0 | 0 | 4 | f | f | 0 | 0.13483146 | 0.05764411 | POST |
| ghirlanda1.61 | ghirlanda | 1 | 1.6 | giangi | 0 | 0 | 3 | f | m | 0.3043478 | 0.0791014 | 0.215686275 | POST |
| ghirlanda1.61 | ghirlanda | 1 | 1.6 | gessica | 0 | 0 | 3 | f | f | 0.01980198 | 0.07920792 | 0.039735099 | POST |
| ghirlanda1.61 | ghirlanda | 1 | 1.6 | gianna | 0 | 0 | 3 | f | f | 0.03296703 | 0.12087912 | 0.03163017 | POST |
| ghirlanda1.741 | ghirlanda | 1 | 1.74 | gianna | only seen | 1 | 5 | f | f | 0.03296703 | 0.12087912 | 0.03163017 | POST |
| ghirlanda1.741 | ghirlanda | 1 | 1.74 | ghiozza | only seen | 1 | 5 | f | f | 0.066666667 | 0.026666667 | 0.017094017 | POST |
| ghirlanda1.741 | ghirlanda | 1 | 1.74 | gessica | 0 | 0 | 5 | f | f | 0.01980198 | 0.07920792 | 0.039735099 | POST |
| ghirlanda1.741 | ghirlanda | 1 | 1.74 | gatta | 0 | 0 | 5 | f | f | 0.1125 | 0.175 | 0.016528926 | POST |
| ghirlanda1.741 | ghirlanda | 1 | 1.74 | giangi | 0 | 1 | 5 | f | m | 0.3043478 | 0.0791014 | 0.215686275 | POST |
| ghirlanda2.0063 | ghirlanda | 3 | 2.006 | gianna | only seen | 0 | 2 | f | f | 0.03296703 | 0.12087912 | 0.03163017 | POST |
| ghirlanda2.0063 | ghirlanda | 3 | 2.006 | giangi | 0 | 0 | 2 | f | m | 0.3043478 | 0.0791014 | 0.215686275 | POST |
| ghirlanda2.0431 | ghirlanda | 1 | 2.043 | giangi | only seen | 1 | 3 | f | m | 0.3043478 | 0.0791014 | 0.215686275 | POST |
| ghirlanda2.0431 | ghirlanda | 1 | 2.043 | gianna | 0 | 0 | 3 | f | f | 0.03296703 | 0.12087912 | 0.03163017 | POST |
| ghirlanda2.0431 | ghirlanda | 1 | 2.043 | gessica | 0 | 0 | 3 | f | f | 0.01980198 | 0.07920792 | 0.039735099 | POST |
| ghirlanda2.091 | ghirlanda | 1 | 2.09 | giangi | 0 | 0 | 3 | f | m | 0.3043478 | 0.0791014 | 0.215686275 | POST |
| ghirlanda2.091 | ghirlanda | 1 | 2.09 | gessica | 0 | 0 | 3 | f | f | 0.01980198 | 0.07920792 | 0.039735099 | POST |
| ghirlanda2.091 | ghirlanda | 1 | 2.09 | gianna | 0 | 0 | 3 | f | f | 0.03296703 | 0.12087912 | 0.03163017 | POST |
| ghirlanda2.123 | ghirlanda | 3 | 2.12 | gianna | only seen | 0 | 4 | f | f | 0.03296703 | 0.12087912 | 0.03163017 | POST |
| ghirlanda2.123 | ghirlanda | 3 | 2.12 | ghiozza | 0 | 0 | 4 | f | f | 0.066666667 | 0.026666667 | 0.017094017 | POST |
| ghirlanda2.123 | ghirlanda | 3 | 2.12 | giangi | 0 | 1 | 4 | f | m | 0.3043478 | 0.0791014 | 0.215686275 | POST |
| ghirlanda2.123 | ghirlanda | 3 | 2.12 | grappolo | 0 | 0 | 4 | f | f | 0 | 0.13483146 | 0.05764411 | POST |
| ghirlanda2.273 | ghirlanda | 3 | 2.27 | giangi | only seen | 0 | 4 | f | m | 0.3043478 | 0.0791014 | 0.215686275 | POST |
| ghirlanda2.273 | ghirlanda | 3 | 2.27 | gianna | only seen | 0 | 4 | f | f | 0.03296703 | 0.12087912 | 0.03163017 | POST |
| ghirlanda2.273 | ghirlanda | 3 | 2.27 | gessica | 0 | 1 | 4 | f | f | 0.01980198 | 0.07920792 | 0.039735099 | POST |
| ghirlanda2.273 | ghirlanda | 3 | 2.27 | grappolo | 0 | 0 | 4 | f | f | 0 | 0.13483146 | 0.05764411 | POST |
| ghirlanda2.331 | ghirlanda | 1 | 2.33 | giangi | 0 | 0 | 1 | f | m | 0.3043478 | 0.0791014 | 0.215686275 | POST |
| ghirlanda2.4321 | ghirlanda | 1 | 2.432 | biondo | 0 | 0 | 4 | f | m | 0 | 0 | 0.14021164 | POST |
| ghirlanda2.4321 | ghirlanda | 1 | 2.432 | black | 0 | 0 | 4 | f | m | 0 | 0 | 0.008130081 | POST |
| ghirlanda2.4321 | ghirlanda | 1 | 2.432 | bratz | 0 | 0 | 4 | f | f | 0 | 0 | 0.059322034 | POST |
| ghirlanda2.4321 | ghirlanda | 1 | 2.432 | giangi | 0 | 0 | 4 | f | m | 0.3043478 | 0.0791014 | 0.215686275 | POST |
| ghirlanda2.721 | ghirlanda | 1 | 2.72 | giangi | only heard | 0 | 4 | f | m | 0.3043478 | 0.0791014 | 0.215686275 | POST |
| ghirlanda2.721 | ghirlanda | 1 | 2.72 | gianna | only heard | 0 | 4 | f | f | 0.03296703 | 0.12087912 | 0.03163017 | PRE |
| ghirlanda2.721 | ghirlanda | 1 | 2.72 | grappolo | only heard | 0 | 4 | f | f | 0 | 0.13483146 | 0.05764411 | PRE |
| ghirlanda2.721 | ghirlanda | 1 | 2.72 | gatta | only heard | 1 | 4 | f | f | 0.1125 | 0.175 | 0.016528926 | PRE |
| ghirlanda3.051 | ghirlanda | 1 | 3.05 | grappolo | 0 | 0 | 4 | f | f | 0 | 0.13483146 | 0.05764411 | PRE |
| ghirlanda3.051 | ghirlanda | 1 | 3.05 | gianna | 0 | 0 | 4 | f | f | 0.03296703 | 0.12087912 | 0.03163017 | PRE |
| ghirlanda3.051 | ghirlanda | 1 | 3.05 | gigio | 0 | 0 | 4 | f | m | 0 | 0.17857142 | 0 | PRE |
| ghirlanda3.051 | ghirlanda | 1 | 3.05 | giangi | 0 | 0 | 4 | f | m | 0.3043478 | 0.0791014 | 0.215686275 | PRE |
| giangi1.081 | giangi | 1 | 1.08 | grappolo | only heard | 0 | 4 | m | f | 0.14529914 | 0.05982904 | 0.05764411 | POST |
| giangi1.081 | giangi | 1 | 1.08 | gessica | only heard | 0 | 4 | m | f | 0.2189781 | 0.1094895 | 0.039735099 | POST |
| giangi1.081 | giangi | 1 | 1.08 | ghirlanda | only heard | 0 | 4 | m | f | 0.3043478 | 0.0791014 | 0.023148148 | POST |
| giangi1.081 | giangi | 1 | 1.08 | gatta | only heard | 0 | 4 | m | f | 0 | 0.05660371 | 0.016528926 | POST |
| giangi1.093 | giangi | 3 | 1.09 | ghirlanda | only seen | 0 | 2 | m | f | 0.3043478 | 0.0791014 | 0.023148148 | POST |
| giangi1.093 | giangi | 3 | 1.09 | gianna | 0 | 0 | 2 | m | f | 0.31404958 | 0.1322314 | 0.03163017 | POST |
| giangi1.111 | giangi | 1 | 1.11 | ghiozza | seen and heard | 0 | 3 | m | f | 0 | 0.07920792 | 0.017094017 | POST |
| giangi1.111 | giangi | 1 | 1.11 | gessica | seen and heard | 1 | 3 | m | f | 0.2189781 | 0.1094895 | 0.039735099 | POST |
| giangi1.111 | giangi | 1 | 1.11 | ghirlanda | only heard | 0 | 3 | m | f | 0.3043478 | 0.0791014 | 0.023148148 | POST |
| giangi1.113 | giangi | 3 | 1.11 | ghiozza | only seen | 0 | 2 | m | f | 0 | 0.07920792 | 0.017094017 | POST |
| giangi1.113 | giangi | 3 | 1.11 | gatta | only seen | 0 | 2 | m | f | 0 | 0.05660371 | 0.016528926 | POST |
| giangi1.263 | giangi | 3 | 1.26 | grappolo | only seen | 0 | 1 | m | f | 0.14529914 | 0.05982904 | 0.05764411 | POST |
| giangi1.282 | giangi | 2 | 1.28 | grappolo | seen and heard | 0 | 3 | m | f | 0.14529914 | 0.05982904 | 0.05764411 | POST |
| giangi1.282 | giangi | 2 | 1.28 | ghirlanda | only heard | 0 | 3 | m | f | 0.3043478 | 0.0791014 | 0.023148148 | POST |
| giangi1.282 | giangi | 2 | 1.28 | gessica | only heard | 0 | 3 | m | f | 0.2189781 | 0.1094895 | 0.039735099 | POST |
| giangi1.293 | giangi | 3 | 1.29 | gessica | seen and heard | 0 | 2 | m | f | 0.2189781 | 0.1094895 | 0.039735099 | POST |
| giangi1.293 | giangi | 3 | 1.29 | ghirlanda | only heard | 0 | 2 | m | f | 0.3043478 | 0.0791014 | 0.023148148 | POST |
| giangi1.311 | giangi | 1 | 1.31 | gessica | only seen | 0 | 6 | m | f | 0.2189781 | 0.1094895 | 0.039735099 | POST |
| giangi1.312 | giangi | 2 | 1.31 | ghiozza | seen and heard | 0 | 4 | m | f | 0 | 0.07920792 | 0.017094017 | POST |
| giangi1.312 | giangi | 2 | 1.31 | ghirlanda | only heard | 0 | 4 | m | f | 0.3043478 | 0.0791014 | 0.023148148 | POST |
| giangi1.312 | giangi | 2 | 1.31 | grappolo | only heard | 0 | 4 | m | f | 0.14529914 | 0.05982904 | 0.05764411 | POST |
| giangi1.312 | giangi | 2 | 1.31 | gianna | only heard | 0 | 4 | m | f | 0.31404958 | 0.1322314 | 0.03163017 | POST |
| giangi1.311 | giangi | 1 | 1.31 | gianna | 0 | 0 | 6 | m | f | 0.31404958 | 0.1322314 | 0.03163017 | POST |
| giangi1.311 | giangi | 1 | 1.31 | ghiozza | 0 | 0 | 6 | m | f | 0 | 0.07920792 | 0.017094017 | POST |
| giangi1.311 | giangi | 1 | 1.31 | gigetto | 0 | 0 | 6 | m | m | 0 | 0 | 0.039548023 | POST |
| giangi1.311 | giangi | 1 | 1.31 | gianna | 0 | 0 | 6 | m | f | 0.31404958 | 0.1322314 | 0.03163017 | POST |
| giangi1.311 | giangi | 1 | 1.31 | grappolo | 0 | 0 | 6 | m | f | 0.14529914 | 0.05982904 | 0.05764411 | POST |
| giangi1.322 | giangi | 2 | 1.32 | ghiozza | only heard | 0 | 2 | m | f | 0 | 0.07920792 | 0.017094017 | POST |
| giangi1.411 | giangi | 1 | 1.41 | gessica | only seen | 0 | 3 | m | f | 0.2189781 | 0.1094895 | 0.039735099 | POST |
| giangi1.411 | giangi | 1 | 1.41 | ghirlanda | 0 | 0 | 3 | m | f | 0.3043478 | 0.0791014 | 0.023148148 | POST |
| giangi1.411 | giangi | 1 | 1.41 | ghiozza | 0 | 0 | 3 | m | f | 0 | 0.07920792 | 0.017094017 | POST |
| giangi1.433 | giangi | 3 | 1.43 | gessica | only heard | 0 | 1 | m | f | 0.2189781 | 0.1094895 | 0.039735099 | POST |
| giangi1.453 | giangi | 3 | 1.45 | gianna | seen and heard | 0 | 1 | m | f | 0.31404958 | 0.1322314 | 0.03163017 | POST |
| giangi1.463 | giangi | 3 | 1.46 | gianna | only seen | 0 | 2 | m | f | 0.31404958 | 0.1322314 | 0.03163017 | POST |
| giangi1.463 | giangi | 3 | 1.46 | gessica | only heard | 0 | 1 | m | f | 0.2189781 | 0.1094895 | 0.039735099 | POST |
| giangi1.463 | giangi | 3 | 1.46 | gessica | 0 | 0 | 2 | m | f | 0.2189781 | 0.1094895 | 0.039735099 | POST |
| giangi1.5312 | giangi | 2 | 1.531 | ghirlanda | seen and heard | 0 | 5 | m | f | 0.3043478 | 0.0791014 | 0.023148148 | POST |
| giangi1.5312 | giangi | 2 | 1.531 | grappolo | seen and heard | 0 | 5 | m | f | 0.14529914 | 0.05982904 | 0.05764411 | POST |
| giangi1.5312 | giangi | 2 | 1.531 | gianna | only heard | 0 | 5 | m | f | 0.31404958 | 0.1322314 | 0.03163017 | POST |
| giangi1.5312 | giangi | 2 | 1.531 | gigio | only heard | 0 | 5 | m | m | 0 | 0.027777778 | 0 | POST |
| giangi1.563 | giangi | 3 | 1.56 | ghirlanda | seen and heard | 0 | 6 | m | f | 0.3043478 | 0.0791014 | 0.023148148 | POST |
| giangi1.563 | giangi | 3 | 1.56 | gianna | seen and heard | 1 | 6 | m | f | 0.31404958 | 0.1322314 | 0.03163017 | POST |
| giangi1.563 | giangi | 3 | 1.56 | gessica | only heard | 0 | 6 | m | f | 0.2189781 | 0.1094895 | 0.039735099 | POST |
| giangi1.563 | giangi | 3 | 1.56 | gigetto | only heard | 0 | 6 | m | m | 0 | 0 | 0.039548023 | POST |
| giangi1.563 | giangi | 3 | 1.56 | ghiozza | only heard | 0 | 6 | m | f | 0 | 0.07920792 | 0.017094017 | POST |
| giangi1.563 | giangi | 3 | 1.56 | gatta | only heard | 0 | 6 | m | f | 0 | 0.05660371 | 0.016528926 | POST |
| giangi1.581 | giangi | 1 | 1.58 | ghirlanda | seen and heard | 0 | 2 | m | f | 0.3043478 | 0.0791014 | 0.023148148 | POST |
| giangi1.581 | giangi | 1 | 1.58 | gianna | only heard | 0 | 2 | m | f | 0.31404958 | 0.1322314 | 0.03163017 | POST |
| giangi1.63 | giangi | 3 | 1.6 | grappolo | seen and heard | 0 | 5 | m | f | 0.14529914 | 0.05982904 | 0.05764411 | POST |
| giangi1.63 | giangi | 3 | 1.6 | ghirlanda | seen and heard | 0 | 5 | m | f | 0.3043478 | 0.0791014 | 0.023148148 | POST |
| giangi1.63 | giangi | 3 | 1.6 | gatta | seen and heard | 0 | 5 | m | f | 0 | 0.05660371 | 0.016528926 | POST |
| giangi1.63 | giangi | 3 | 1.6 | gianna | only heard | 1 | 5 | m | f | 0.31404958 | 0.1322314 | 0.03163017 | POST |
| giangi1.63 | giangi | 3 | 1.6 | gessica | only heard | 1 | 5 | m | f | 0.2189781 | 0.1094895 | 0.039735099 | POST |
| giangi1.6043 | giangi | 3 | 1.604 | gianna | seen and heard | 0 | 3 | m | f | 0.31404958 | 0.1322314 | 0.03163017 | POST |
| giangi1.6043 | giangi | 3 | 1.604 | gigetto | only heard | 0 | 3 | m | m | 0 | 0 | 0.039548023 | POST |
| giangi1.6043 | giangi | 3 | 1.604 | ghiozza | only heard | 0 | 3 | m | f | 0 | 0.07920792 | 0.017094017 | POST |
| giangi1.612 | giangi | 2 | 1.61 | susy | only heard | 0 | 14 | m | f | 0 | 0 | 0.027777778 | POST |
| giangi1.612 | giangi | 2 | 1.61 | spot | only heard | 0 | 14 | m | f | 0 | 0 | 0.020576132 | POST |
| giangi1.612 | giangi | 2 | 1.61 | strega | only heard | 1 | 14 | m | f | 0 | 0 | 0.016025641 | POST |
| giangi1.612 | giangi | 2 | 1.61 | rosa | only heard | 0 | 14 | m | f | 0 | 0 | 0.027131783 | POST |
| giangi1.612 | giangi | 2 | 1.61 | sine | only heard | 0 | 14 | m | f | 0 | 0 | 0.050682261 | POST |
| giangi1.612 | giangi | 2 | 1.61 | scapola | only heard | 0 | 14 | m | f | 0 | 0 | 0.003787879 | POST |
| giangi1.612 | giangi | 2 | 1.61 | stacy | only heard | 0 | 14 | m | f | 0 | 0 | 0.021317829 | POST |
| giangi1.612 | giangi | 2 | 1.61 | small | only heard | 0 | 14 | m | f | 0 | 0 | 0.044117647 | POST |
| giangi1.612 | giangi | 2 | 1.61 | stella | only heard | 0 | 14 | m | f | 0 | 0 | 0.020080321 | POST |
| giangi1.612 | giangi | 2 | 1.61 | spiga | only heard | 0 | 14 | m | f | 0 | 0 | 0.018372703 | POST |
| giangi1.612 | giangi | 2 | 1.61 | sally | only heard | 0 | 14 | m | f | 0 | 0 | 0.002949853 | POST |
| giangi1.612 | giangi | 2 | 1.61 | schiarita | only heard | 0 | 14 | m | f | 0 | 0 | 0.041420118 | POST |
| giangi1.612 | giangi | 2 | 1.61 | secco | only heard | 0 | 14 | m | m | 0 | 0 | 0.123076923 | POST |
| giangi1.612 | giangi | 2 | 1.61 | neomamma | only heard | 0 | 14 | m | f | 0 | 0 | 0.032840722 | POST |
| giangi1.612 | giangi | 2 | 1.61 | gessica | seen and heard | 0 | 3 | m | f | 0.2189781 | 0.1094895 | 0.039735099 | POST |
| giangi1.612 | giangi | 2 | 1.61 | gatta | seen and heard | 0 | 3 | m | f | 0 | 0.05660371 | 0.016528926 | POST |
| giangi1.612 | giangi | 2 | 1.61 | ghirlanda | 0 | 0 | 3 | m | f | 0.3043478 | 0.0791014 | 0.023148148 | POST |
| giangi1.621 | giangi | 1 | 1.62 | gianna | only seen | 1 | 9 | m | f | 0.31404958 | 0.1322314 | 0.03163017 | POST |
| giangi1.621 | giangi | 1 | 1.62 | gessica | only seen | 0 | 9 | m | f | 0.2189781 | 0.1094895 | 0.039735099 | POST |
| giangi1.621 | giangi | 1 | 1.62 | ghirlanda | seen and heard | 0 | 2 | m | f | 0.3043478 | 0.0791014 | 0.023148148 | POST |
| giangi1.621 | giangi | 1 | 1.62 | gessica | only heard | 1 | 2 | m | f | 0.2189781 | 0.1094895 | 0.039735099 | POST |
| giangi1.621 | giangi | 1 | 1.62 | grappolo | 0 | 0 | 9 | m | f | 0.14529914 | 0.05982904 | 0.05764411 | POST |
| giangi1.621 | giangi | 1 | 1.62 | ghirlanda | 0 | 0 | 9 | m | f | 0.3043478 | 0.0791014 | 0.023148148 | POST |
| giangi1.621 | giangi | 1 | 1.62 | gatta | 0 | 0 | 9 | m | f | 0 | 0.05660371 | 0.016528926 | POST |
| giangi1.621 | giangi | 1 | 1.62 | ghiozza | 0 | 0 | 9 | m | f | 0 | 0.07920792 | 0.017094017 | POST |
| giangi1.621 | giangi | 1 | 1.62 | gigio | 0 | 0 | 9 | m | m | 0 | 0.027777778 | 0 | POST |
| giangi1.621 | giangi | 1 | 1.62 | gigetto | 0 | 0 | 9 | m | m | 0 | 0 | 0.039548023 | POST |
| giangi1.621 | giangi | 1 | 1.62 | gelly | 0 | 0 | 9 | m | f | 0 | 0 | 0 | POST |
| giangi1.633 | giangi | 3 | 1.63 | grappolo | only heard | 0 | 2 | m | f | 0.14529914 | 0.05982904 | 0.05764411 | POST |
| giangi1.633 | giangi | 3 | 1.63 | ghiozza | only heard | 0 | 2 | m | f | 0 | 0.07920792 | 0.017094017 | POST |
| giangi1.632 | giangi | 2 | 1.63 | gatta | only heard | 0 | 2 | m | f | 0 | 0.05660371 | 0.016528926 | POST |
| giangi1.632 | giangi | 2 | 1.63 | ghiozza | only heard | 0 | 2 | m | f | 0 | 0.07920792 | 0.017094017 | POST |
| giangi1.6393 | giangi | 3 | 1.639 | gianna | seen and heard | 0 | 6 | m | f | 0.31404958 | 0.1322314 | 0.03163017 | POST |
| giangi1.6393 | giangi | 3 | 1.639 | biondo | only heard | 0 | 6 | m | m | 0 | 0 | 0.14021164 | POST |
| giangi1.6393 | giangi | 3 | 1.639 | black | only heard | 0 | 6 | m | m | 0 | 0 | 0.008130081 | POST |
| giangi1.6393 | giangi | 3 | 1.639 | biba | only heard | 0 | 6 | m | f | 0 | 0 | 0.011111111 | POST |
| giangi1.6393 | giangi | 3 | 1.639 | bratz | only heard | 0 | 6 | m | f | 0 | 0 | 0.059322034 | POST |
| giangi1.6393 | giangi | 3 | 1.639 | ghirlanda | only heard | 1 | 6 | m | f | 0.3043478 | 0.0791014 | 0.023148148 | POST |
| giangi1.652 | giangi | 2 | 1.65 | grappolo | only heard | 0 | 4 | m | f | 0.14529914 | 0.05982904 | 0.05764411 | POST |
| giangi1.652 | giangi | 2 | 1.65 | gigio | only heard | 0 | 4 | m | m | 0 | 0.027777778 | 0 | POST |
| giangi1.652 | giangi | 2 | 1.65 | ghiozza | only heard | 0 | 4 | m | f | 0 | 0.07920792 | 0.017094017 | POST |
| giangi1.652 | giangi | 2 | 1.65 | gatta | only heard | 0 | 4 | m | f | 0 | 0.05660371 | 0.016528926 | POST |
| giangi1.711 | giangi | 1 | 1.71 | gatta | only heard | 0 | 3 | m | f | 0 | 0.05660371 | 0.016528926 | POST |
| giangi1.711 | giangi | 1 | 1.71 | gessica | only heard | 0 | 3 | m | f | 0.2189781 | 0.1094895 | 0.039735099 | POST |
| giangi1.711 | giangi | 1 | 1.71 | ghirlanda | only heard | 0 | 3 | m | f | 0.3043478 | 0.0791014 | 0.023148148 | POST |
| giangi1.743 | giangi | 3 | 1.74 | gatta | seen and heard | 0 | 7 | m | f | 0 | 0.05660371 | 0.016528926 | POST |
| giangi1.743 | giangi | 3 | 1.74 | ghirlanda | only heard | 0 | 7 | m | f | 0.3043478 | 0.0791014 | 0.023148148 | POST |
| giangi1.743 | giangi | 3 | 1.74 | grappolo | only heard | 1 | 7 | m | f | 0.14529914 | 0.05982904 | 0.05764411 | POST |
| giangi1.743 | giangi | 3 | 1.74 | gessica | only heard | 1 | 7 | m | f | 0.2189781 | 0.1094895 | 0.039735099 | POST |
| giangi1.743 | giangi | 3 | 1.74 | ghiozza | only heard | 1 | 7 | m | f | 0 | 0.07920792 | 0.017094017 | POST |
| giangi1.743 | giangi | 3 | 1.74 | gianna | only heard | 0 | 7 | m | f | 0.31404958 | 0.1322314 | 0.03163017 | POST |
| giangi1.743 | giangi | 3 | 1.74 | gigetto | only heard | 1 | 7 | m | m | 0 | 0 | 0.039548023 | POST |
| giangi1.823 | giangi | 3 | 1.82 | ghirlanda | only heard | 0 | 2 | m | f | 0.3043478 | 0.0791014 | 0.023148148 | POST |
| giangi1.823 | giangi | 3 | 1.82 | ghiozza | only heard | 0 | 2 | m | f | 0 | 0.07920792 | 0.017094017 | POST |
| giangi1.8442 | giangi | 2 | 1.844 | gianna | seen and heard | 0 | 3 | m | f | 0.31404958 | 0.1322314 | 0.03163017 | POST |
| giangi1.8442 | giangi | 2 | 1.844 | ghirlanda | only heard | 1 | 3 | m | f | 0.3043478 | 0.0791014 | 0.023148148 | POST |
| giangi1.8442 | giangi | 2 | 1.844 | gessica | only heard | 0 | 3 | m | f | 0.2189781 | 0.1094895 | 0.039735099 | POST |
| giangi1.92 | giangi | 2 | 1.9 | gianna | only heard | 0 | 3 | m | f | 0.31404958 | 0.1322314 | 0.03163017 | POST |
| giangi1.92 | giangi | 2 | 1.9 | ghirlanda | only heard | 0 | 3 | m | f | 0.3043478 | 0.0791014 | 0.023148148 | POST |
| giangi1.92 | giangi | 2 | 1.9 | gessica | only heard | 0 | 3 | m | f | 0.2189781 | 0.1094895 | 0.039735099 | POST |
| giangi1.9082 | giangi | 2 | 1.908 | gianna | 0 | 0 | 7 | m | f | 0.31404958 | 0.1322314 | 0.03163017 | POST |
| giangi1.9082 | giangi | 2 | 1.908 | gigio | 0 | 0 | 7 | m | m | 0 | 0.027777778 | 0 | POST |
| giangi1.9082 | giangi | 2 | 1.908 | gigetto | 0 | 0 | 7 | m | m | 0 | 0 | 0.039548023 | POST |
| giangi1.9082 | giangi | 2 | 1.908 | gatta | 0 | 0 | 7 | m | f | 0 | 0.05660371 | 0.016528926 | POST |
| giangi1.9082 | giangi | 2 | 1.908 | ghirlanda | 0 | 0 | 7 | m | f | 0.3043478 | 0.0791014 | 0.023148148 | POST |
| giangi1.9082 | giangi | 2 | 1.908 | grappolo | 0 | 0 | 7 | m | f | 0.14529914 | 0.05982904 | 0.05764411 | POST |
| giangi1.9082 | giangi | 2 | 1.908 | gessica | 0 | 0 | 7 | m | f | 0.2189781 | 0.1094895 | 0.039735099 | POST |
| giangi1.943 | giangi | 3 | 1.94 | ghirlanda | seen and heard | 0 | 5 | m | f | 0.3043478 | 0.0791014 | 0.023148148 | POST |
| giangi1.943 | giangi | 3 | 1.94 | grappolo | only heard | 0 | 5 | m | f | 0.14529914 | 0.05982904 | 0.05764411 | POST |
| giangi1.943 | giangi | 3 | 1.94 | gigio | only heard | 0 | 5 | m | m | 0 | 0.027777778 | 0 | POST |
| giangi1.943 | giangi | 3 | 1.94 | ghiozza | only heard | 0 | 5 | m | f | 0 | 0.07920792 | 0.017094017 | POST |
| giangi1.943 | giangi | 3 | 1.94 | gatta | only heard | 0 | 5 | m | f | 0 | 0.05660371 | 0.016528926 | POST |
| giangi1.962 | giangi | 2 | 1.96 | grappolo | seen and heard | 0 | 4 | m | f | 0.14529914 | 0.05982904 | 0.05764411 | POST |
| giangi1.962 | giangi | 2 | 1.96 | gigetto | only heard | 0 | 4 | m | m | 0 | 0 | 0.039548023 | POST |
| giangi1.962 | giangi | 2 | 1.96 | gessica | only heard | 0 | 4 | m | f | 0.2189781 | 0.1094895 | 0.039735099 | POST |
| giangi1.962 | giangi | 2 | 1.96 | gianna | only heard | 0 | 4 | m | f | 0.31404958 | 0.1322314 | 0.03163017 | POST |
| giangi23 | giangi | 3 | 2 | ghirlanda | only seen | 0 | 1 | m | f | 0.3043478 | 0.0791014 | 0.023148148 | POST |
| giangi2.022 | giangi | 2 | 2.02 | gessica | seen and heard | 0 | 3 | m | f | 0.2189781 | 0.1094895 | 0.039735099 | POST |
| giangi2.022 | giangi | 2 | 2.02 | grappolo | only heard | 0 | 3 | m | f | 0.14529914 | 0.05982904 | 0.05764411 | POST |
| giangi2.022 | giangi | 2 | 2.02 | ghiozza | only heard | 0 | 3 | m | f | 0 | 0.07920792 | 0.017094017 | POST |
| giangi2.073 | giangi | 3 | 2.07 | gatta | only seen | 0 | 3 | m | f | 0 | 0.05660371 | 0.016528926 | POST |
| giangi2.073 | giangi | 3 | 2.07 | ghirlanda | 0 | 0 | 3 | m | f | 0.3043478 | 0.0791014 | 0.023148148 | POST |
| giangi2.073 | giangi | 3 | 2.07 | gianna | 0 | 0 | 3 | m | f | 0.31404958 | 0.1322314 | 0.03163017 | POST |
| giangi2.21 | giangi | 1 | 2.2 | gessica | only heard | 1 | 6 | m | f | 0.2189781 | 0.1094895 | 0.039735099 | POST |
| giangi2.21 | giangi | 1 | 2.2 | gatta | only heard | 0 | 6 | m | f | 0 | 0.05660371 | 0.016528926 | POST |
| giangi2.21 | giangi | 1 | 2.2 | gigetto | only heard | 0 | 6 | m | m | 0 | 0 | 0.039548023 | POST |
| giangi2.21 | giangi | 1 | 2.2 | grappolo | only heard | 0 | 6 | m | f | 0.14529914 | 0.05982904 | 0.05764411 | POST |
| giangi2.21 | giangi | 1 | 2.2 | ghiozza | only heard | 0 | 6 | m | f | 0 | 0.07920792 | 0.017094017 | POST |
| giangi2.21 | giangi | 1 | 2.2 | gelly | only heard | 0 | 6 | m | f | 0 | 0 | 0 | POST |
| giangi2.22 | giangi | 2 | 2.2 | ghirlanda | seen and heard | 0 | 1 | m | f | 0.3043478 | 0.0791014 | 0.023148148 | POST |
| giangi2.292 | giangi | 2 | 2.29 | gessica | only heard | 0 | 2 | m | f | 0.2189781 | 0.1094895 | 0.039735099 | POST |
| giangi2.292 | giangi | 2 | 2.29 | gigetto | only heard | 0 | 2 | m | m | 0 | 0 | 0.039548023 | POST |
| giangi2.472 | giangi | 2 | 2.47 | grappolo | seen and heard | 0 | 4 | m | f | 0.14529914 | 0.05982904 | 0.05764411 | POST |
| giangi2.472 | giangi | 2 | 2.47 | gessica | only heard | 0 | 4 | m | f | 0.2189781 | 0.1094895 | 0.039735099 | POST |
| giangi2.472 | giangi | 2 | 2.47 | gianna | only heard | 0 | 4 | m | f | 0.31404958 | 0.1322314 | 0.03163017 | POST |
| giangi2.472 | giangi | 2 | 2.47 | ghirlanda | only heard | 0 | 4 | m | f | 0.3043478 | 0.0791014 | 0.023148148 | POST |
| giangi2.533 | giangi | 3 | 2.53 | gianna | seen and heard | 0 | 2 | m | f | 0.31404958 | 0.1322314 | 0.03163017 | POST |
| giangi2.533 | giangi | 3 | 2.53 | gatta | only heard | 1 | 2 | m | f | 0 | 0.05660371 | 0.016528926 | POST |
| giangi2.553 | giangi | 3 | 2.55 | ghirlanda | only seen | 0 | 5 | m | f | 0.3043478 | 0.0791014 | 0.023148148 | POST |
| giangi2.553 | giangi | 3 | 2.55 | grappolo | 0 | 0 | 5 | m | f | 0.14529914 | 0.05982904 | 0.05764411 | POST |
| giangi2.553 | giangi | 3 | 2.55 | gigio | 0 | 0 | 5 | m | m | 0 | 0.027777778 | 0 | POST |
| giangi2.553 | giangi | 3 | 2.55 | ghiozza | 0 | 0 | 5 | m | f | 0 | 0.07920792 | 0.017094017 | POST |
| giangi2.553 | giangi | 3 | 2.55 | gatta | 0 | 0 | 5 | m | f | 0 | 0.05660371 | 0.016528926 | POST |
| giangi2.62 | giangi | 2 | 2.6 | grappolo | seen and heard | 0 | 3 | m | f | 0.14529914 | 0.05982904 | 0.05764411 | POST |
| giangi2.62 | giangi | 2 | 2.6 | gessica | seen and heard | 0 | 3 | m | f | 0.2189781 | 0.1094895 | 0.039735099 | POST |
| giangi2.62 | giangi | 2 | 2.6 | gianna | only heard | 0 | 3 | m | f | 0.31404958 | 0.1322314 | 0.03163017 | POST |
| giangi2.673 | giangi | 3 | 2.67 | gianna | seen and heard | 0 | 1 | m | f | 0.31404958 | 0.1322314 | 0.03163017 | POST |
| giangi2.72 | giangi | 2 | 2.7 | ghirlanda | seen and heard | 0 | 7 | m | f | 0.3043478 | 0.0791014 | 0.023148148 | POST |
| giangi2.72 | giangi | 2 | 2.7 | grappolo | seen and heard | 0 | 7 | m | f | 0.14529914 | 0.05982904 | 0.05764411 | POST |
| giangi2.72 | giangi | 2 | 2.7 | gessica | only heard | 0 | 7 | m | f | 0.2189781 | 0.1094895 | 0.039735099 | POST |
| giangi2.72 | giangi | 2 | 2.7 | gatta | only heard | 0 | 7 | m | f | 0 | 0.05660371 | 0.016528926 | POST |
| giangi2.72 | giangi | 2 | 2.7 | gianna | only heard | 0 | 7 | m | f | 0.31404958 | 0.1322314 | 0.03163017 | POST |
| giangi2.72 | giangi | 2 | 2.7 | gelly | only heard | 0 | 7 | m | f | 0 | 0 | 0 | POST |
| giangi2.72 | giangi | 2 | 2.7 | gigio | only heard | 0 | 7 | m | m | 0 | 0.027777778 | 0 | POST |
| giangi2.852 | giangi | 2 | 2.85 | black | only heard | 0 | 4 | m | m | 0 | 0 | 0.008130081 | POST |
| giangi2.852 | giangi | 2 | 2.85 | betta | only heard | 0 | 4 | m | f | 0 | 0 | 0.00408998 | POST |
| giangi2.852 | giangi | 2 | 2.85 | braccio | only heard | 0 | 4 | m | f | 0 | 0 | 0.02 | POST |
| giangi3.23 | giangi | 3 | 3.2 | ghirlanda | seen and heard | 0 | 2 | m | f | 0.3043478 | 0.0791014 | 0.023148148 | POST |
| giangi3.23 | giangi | 3 | 3.2 | gessica | only heard | 0 | 2 | m | f | 0.2189781 | 0.1094895 | 0.039735099 | POST |
| giangi3.2313 | giangi | 3 | 3.231 | gianna | seen and heard | 0 | 7 | m | f | 0.31404958 | 0.1322314 | 0.03163017 | POST |
| giangi3.2313 | giangi | 3 | 3.231 | gigio | only heard | 0 | 7 | m | m | 0 | 0.027777778 | 0 | POST |
| giangi3.2313 | giangi | 3 | 3.231 | gigetto | only heard | 0 | 7 | m | m | 0 | 0 | 0.039548023 | POST |
| giangi3.2313 | giangi | 3 | 3.231 | gatta | only heard | 0 | 7 | m | f | 0 | 0.05660371 | 0.016528926 | POST |
| giangi3.2313 | giangi | 3 | 3.231 | ghirlanda | only heard | 0 | 7 | m | f | 0.3043478 | 0.0791014 | 0.023148148 | POST |
| giangi3.2313 | giangi | 3 | 3.231 | grappolo | only heard | 0 | 7 | m | f | 0.14529914 | 0.05982904 | 0.05764411 | POST |
| giangi3.2313 | giangi | 3 | 3.231 | gessica | only heard | 0 | 7 | m | f | 0.2189781 | 0.1094895 | 0.039735099 | POST |
| giangi3.323 | giangi | 3 | 3.32 | gianna | seen and heard | 0 | 5 | m | f | 0.31404958 | 0.1322314 | 0.03163017 | POST |
| giangi3.323 | giangi | 3 | 3.32 | grappolo | only heard | 0 | 5 | m | f | 0.14529914 | 0.05982904 | 0.05764411 | POST |
| giangi3.323 | giangi | 3 | 3.32 | ghiozza | only heard | 0 | 5 | m | f | 0 | 0.07920792 | 0.017094017 | POST |
| giangi3.323 | giangi | 3 | 3.32 | gessica | only heard | 0 | 5 | m | f | 0.2189781 | 0.1094895 | 0.039735099 | POST |
| giangi3.323 | giangi | 3 | 3.32 | ghirlanda | seen and heard | 0 | 5 | m | f | 0.3043478 | 0.0791014 | 0.023148148 | POST |
| giangi3.423 | giangi | 3 | 3.42 | ghirlanda | seen and heard | 0 | 2 | m | f | 0.3043478 | 0.0791014 | 0.023148148 | POST |
| giangi3.423 | giangi | 3 | 3.42 | ghiozza | only heard | 0 | 2 | m | f | 0 | 0.07920792 | 0.017094017 | POST |
| giangi3.612 | giangi | 2 | 3.61 | ghirlanda | seen and heard | 0 | 4 | m | f | 0.3043478 | 0.0791014 | 0.023148148 | POST |
| giangi3.612 | giangi | 2 | 3.61 | gatta | only heard | 0 | 4 | m | f | 0 | 0.05660371 | 0.016528926 | PRE |
| giangi3.612 | giangi | 2 | 3.61 | ghiozza | only heard | 1 | 4 | m | f | 0 | 0.07920792 | 0.017094017 | PRE |
| giangi3.612 | giangi | 2 | 3.61 | gianna | only heard | 0 | 4 | m | f | 0.31404958 | 0.1322314 | 0.03163017 | PRE |
| giangi3.653 | giangi | 3 | 3.65 | gigetto | seen and heard | 1 | 6 | m | m | 0 | 0 | 0.039548023 | PRE |
| giangi3.653 | giangi | 3 | 3.65 | ghirlanda | seen and heard | 0 | 6 | m | f | 0.3043478 | 0.0791014 | 0.023148148 | PRE |
| giangi3.653 | giangi | 3 | 3.65 | gessica | seen and heard | 0 | 6 | m | f | 0.2189781 | 0.1094895 | 0.039735099 | PRE |
| giangi3.653 | giangi | 3 | 3.65 | ghiozza | only heard | 0 | 6 | m | f | 0 | 0.07920792 | 0.017094017 | PRE |
| giangi3.653 | giangi | 3 | 3.65 | gatta | only heard | 0 | 6 | m | f | 0 | 0.05660371 | 0.016528926 | PRE |
| giangi3.653 | giangi | 3 | 3.65 | gianna | only heard | 0 | 6 | m | f | 0.31404958 | 0.1322314 | 0.03163017 | PRE |
| giangi4.223 | giangi | 3 | 4.22 | gessica | only heard | 1 | 2 | m | f | 0.2189781 | 0.1094895 | 0.039735099 | PRE |
| giangi4.223 | giangi | 3 | 4.22 | ghiozza | only heard | 0 | 2 | m | f | 0 | 0.07920792 | 0.017094017 | PRE |
| giangi6.473 | giangi | 3 | 6.47 | ghirlanda | only seen | 0 | 1 | m | f | 0.3043478 | 0.0791014 | 0.023148148 | PRE |
| giangi10.932 | giangi | 2 | 10.93 | gigetto | seen and heard | 0 | 3 | m | m | 0 | 0 | 0.039548023 | PRE |
| giangi10.932 | giangi | 2 | 10.93 | gessica | only heard | 0 | 3 | m | f | 0.2189781 | 0.1094895 | 0.039735099 | PRE |
| giangi10.932 | giangi | 2 | 10.93 | gianna | only heard | 0 | 3 | m | f | 0.31404958 | 0.1322314 | 0.03163017 | PRE |
| gianna0.71* | gianna | * | 0.71 | giangi | 0 | 0 | 1 | f | m | 0.31404958 | 0.1322314 | 0.215686275 | POST |
| gianna0.931 | gianna | 1 | 0.93 | gessica | only seen | 0 | 6 | f | f | 0.01063829 | 0.23404255 | 0.039735099 | POST |
| gianna0.931 | gianna | 1 | 0.93 | grappolo | only seen | 0 | 6 | f | f | 0.204545455 | 0.285714286 | 0.05764411 | POST |
| gianna0.931 | gianna | 1 | 0.93 | giangi | 0 | 0 | 6 | f | m | 0.31404958 | 0.1322314 | 0.215686275 | POST |
| gianna0.931 | gianna | 1 | 0.93 | ghirlanda | 0 | 0 | 6 | f | f | 0.03296703 | 0.12087912 | 0.023148148 | POST |
| gianna0.931 | gianna | 1 | 0.93 | gatta | 0 | 0 | 6 | f | f | 0.04651162 | 0.13953488 | 0.016528926 | POST |
| gianna0.931 | gianna | 1 | 0.93 | ghiozza | 0 | 0 | 6 | f | f | 0.09523805 | 0.1547619 | 0.017094017 | POST |
| gianna1.161 | gianna | 1 | 1.16 | ghiozza | only seen | 0 | 3 | f | f | 0.09523805 | 0.1547619 | 0.017094017 | POST |
| gianna1.161 | gianna | 1 | 1.16 | gigetto | only seen | 0 | 3 | f | m | 0 | 0.13157894 | 0.039548023 | POST |
| gianna1.161 | gianna | 1 | 1.16 | gatta | 0 | 0 | 3 | f | f | 0.04651162 | 0.13953488 | 0.016528926 | POST |
| gianna1.221 | gianna | 1 | 1.22 | giangi | only seen | 0 | 3 | f | m | 0.31404958 | 0.1322314 | 0.215686275 | POST |
| gianna1.221 | gianna | 1 | 1.22 | ghiozza | 0 | 0 | 3 | f | f | 0.09523805 | 0.1547619 | 0.017094017 | POST |
| gianna1.221 | gianna | 1 | 1.22 | gigetto | 0 | 0 | 3 | f | m | 0 | 0.13157894 | 0.039548023 | POST |
| gianna1.291 | gianna | 1 | 1.29 | ghiozza | only seen | 1 | 3 | f | f | 0.09523805 | 0.1547619 | 0.017094017 | POST |
| gianna1.291 | gianna | 1 | 1.29 | gigetto | 0 | 0 | 3 | f | m | 0 | 0.13157894 | 0.039548023 | POST |
| gianna1.291 | gianna | 1 | 1.29 | gatta | 0 | 0 | 3 | f | f | 0.04651162 | 0.13953488 | 0.016528926 | POST |
| gianna1.311 | gianna | 1 | 1.31 | giangi | 0 | 0 | 1 | f | m | 0.31404958 | 0.1322314 | 0.215686275 | POST |
| gianna1.341 | gianna | 1 | 1.34 | grappolo | only seen | 1 | 4 | f | f | 0.204545455 | 0.113636364 | 0.05764411 | POST |
| gianna1.341 | gianna | 1 | 1.34 | ghirlanda | only seen | 0 | 4 | f | f | 0.03296703 | 0.12087912 | 0.023148148 | POST |
| gianna1.341 | gianna | 1 | 1.34 | giangi | 0 | 0 | 4 | f | m | 0.31404958 | 0.1322314 | 0.215686275 | POST |
| gianna1.341 | gianna | 1 | 1.34 | gessica | 0 | 0 | 4 | f | f | 0.01063829 | 0.23404255 | 0.039735099 | POST |
| gianna1.551 | gianna | 1 | 1.55 | gessica | only seen | 1 | 3 | f | f | 0.01063829 | 0.23404255 | 0.039735099 | POST |
| gianna1.551 | gianna | 1 | 1.55 | giangi | 0 | 0 | 3 | f | m | 0.31404958 | 0.1322314 | 0.215686275 | POST |
| gianna1.611 | gianna | 1 | 1.61 | ghirlanda | only seen | 0 | 3 | f | f | 0.03296703 | 0.12087912 | 0.023148148 | POST |
| gianna1.611 | gianna | 1 | 1.61 | gessica | 0 | 0 | 3 | f | f | 0.01063829 | 0.23404255 | 0.039735099 | POST |
| gianna1.611 | gianna | 1 | 1.61 | ghiozza | 0 | 0 | 3 | f | f | 0.09523805 | 0.1547619 | 0.017094017 | POST |
| gianna1.621 | gianna | 1 | 1.62 | gatta | only seen | 0 | 5 | f | f | 0.04651162 | 0.13953488 | 0.016528926 | POST |
| gianna1.621 | gianna | 1 | 1.62 | gigetto | 0 | 0 | 5 | f | m | 0 | 0.13157894 | 0.039548023 | POST |
| gianna1.621 | gianna | 1 | 1.62 | ghirlanda | 0 | 0 | 5 | f | f | 0.03296703 | 0.12087912 | 0.023148148 | POST |
| gianna1.621 | gianna | 1 | 1.62 | gessica | 0 | 0 | 5 | f | f | 0.01063829 | 0.23404255 | 0.039735099 | POST |
| gianna1.621 | gianna | 1 | 1.62 | ghiozza | 0 | 0 | 5 | f | f | 0.09523805 | 0.1547619 | 0.017094017 | POST |
| gianna1.69* | gianna | * | 1.69 | giangi | 0 | 0 | 1 | f | m | 0.31404958 | 0.1322314 | 0.215686275 | POST |
| gianna1.9343 | gianna | 3 | 1.934 | ghirlanda | seen and heard | 0 | 3 | f | f | 0.03296703 | 0.12087912 | 0.023148148 | POST |
| gianna1.9343 | gianna | 3 | 1.934 | gessica | only heard | 0 | 3 | f | f | 0.01063829 | 0.23404255 | 0.039735099 | POST |
| gianna1.9343 | gianna | 3 | 1.934 | grappolo | only heard | 0 | 3 | f | f | 0.054347826 | 0.1413043 | 0.05764411 | POST |
| gianna2.051 | gianna | 1 | 2.05 | grappolo | 0 | 0 | 1 | f | f | 0.204545455 | 0.285714286 | 0.05764411 | POST |
| gianna2.181 | gianna | 1 | 2.18 | gessica | 0 | 0 | 4 | f | f | 0.01063829 | 0.23404255 | 0.039735099 | POST |
| gianna2.181 | gianna | 1 | 2.18 | ghirlanda | 0 | 0 | 4 | f | f | 0.03296703 | 0.12087912 | 0.023148148 | POST |
| gianna2.181 | gianna | 1 | 2.18 | grappolo | 0 | 0 | 4 | f | f | 0.204545455 | 0.285714286 | 0.05764411 | POST |
| gianna2.181 | gianna | 1 | 2.18 | gatta | 0 | 0 | 4 | f | f | 0.04651162 | 0.13953488 | 0.016528926 | POST |
| gianna2.221 | gianna | 1 | 2.22 | giangi | 0 | 0 | 9 | f | m | 0.31404958 | 0.1322314 | 0.215686275 | POST |
| gianna2.221 | gianna | 1 | 2.22 | gessica | 0 | 0 | 9 | f | f | 0.01063829 | 0.23404255 | 0.039735099 | POST |
| gianna2.221 | gianna | 1 | 2.22 | grappolo | 0 | 0 | 9 | f | f | 0.204545455 | 0.113636364 | 0.05764411 | POST |
| gianna2.221 | gianna | 1 | 2.22 | ghirlanda | 0 | 0 | 9 | f | f | 0.03296703 | 0.12087912 | 0.023148148 | POST |
| gianna2.221 | gianna | 1 | 2.22 | gatta | 0 | 0 | 9 | f | f | 0.04651162 | 0.13953488 | 0.016528926 | POST |
| gianna2.221 | gianna | 1 | 2.22 | ghiozza | 0 | 0 | 9 | f | f | 0.09523805 | 0.1547619 | 0.017094017 | POST |
| gianna2.221 | gianna | 1 | 2.22 | gigio | 0 | 0 | 9 | f | m | 0 | 0 | 0 | POST |
| gianna2.221 | gianna | 1 | 2.22 | gigetto | 0 | 0 | 9 | f | m | 0 | 0.13157894 | 0.039548023 | POST |
| gianna2.221 | gianna | 1 | 2.22 | gelly | 0 | 0 | 9 | f | f | 0 | 0.05882352 | 0 | POST |
| gianna2.281 | gianna | 1 | 2.28 | ghirlanda | only seen | 0 | 5 | f | f | 0.03296703 | 0.12089712 | 0.023148148 | POST |
| gianna2.281 | gianna | 1 | 2.28 | gessica | 0 | 0 | 5 | f | f | 0.01063829 | 0.23404255 | 0.039735099 | POST |
| gianna2.281 | gianna | 1 | 2.28 | grappolo | 0 | 0 | 5 | f | f | 0.204545455 | 0.113636364 | 0.05764411 | POST |
| gianna2.281 | gianna | 1 | 2.28 | gatta | 0 | 0 | 5 | f | f | 0.04651162 | 0.13953488 | 0.016528926 | POST |
| gianna2.281 | gianna | 1 | 2.28 | giangi | 0 | 0 | 5 | f | m | 0.31404958 | 0.1322314 | 0.215686275 | POST |
| gianna2.363 | gianna | 3 | 2.36 | giangi | 0 | 0 | 1 | f | m | 0.31404958 | 0.1322314 | 0.215686275 | POST |
| gianna2.873 | gianna | 3 | 2.87 | giangi | 0 | 0 | 1 | f | m | 0.31404958 | 0.1322314 | 0.215686275 | POST |
| gianna3.0211 | gianna | 1 | 3.021 | gessica | seen and heard | 0 | 4 | f | f | 0.01063829 | 0.23404255 | 0.039735099 | POST |
| gianna3.0211 | gianna | 1 | 3.021 | ghirlanda | only heard | 0 | 4 | f | f | 0.03296703 | 0.12087912 | 0.023148148 | POST |
| gianna3.0211 | gianna | 1 | 3.021 | ghiozza | only heard | 0 | 4 | f | f | 0.09523805 | 0.1547619 | 0.017094017 | POST |
| gianna3.0211 | gianna | 1 | 3.021 | grappolo | only heard | 0 | 4 | f | f | 0.054347826 | 0.1413043 | 0.05764411 | POST |
| gianna4.071 | gianna | 1 | 4.07 | gessica | seen and heard | 0 | 6 | f | f | 0.01063829 | 0.23404255 | 0.039735099 | PRE |
| gianna4.071 | gianna | 1 | 4.07 | giangi | seen and heard | 0 | 6 | f | m | 0.31404958 | 0.1322314 | 0.215686275 | PRE |
| gianna4.071 | gianna | 1 | 4.07 | grappolo | 0 | 0 | 6 | f | f | 0.204545455 | 0.285714286 | 0.05764411 | PRE |
| gianna4.071 | gianna | 1 | 4.07 | gatta | 0 | 0 | 6 | f | f | 0.04651162 | 0.13953488 | 0.016528926 | PRE |
| gianna4.071 | gianna | 1 | 4.07 | gigetto | 0 | 0 | 6 | f | m | 0 | 0.13157894 | 0.039548023 | PRE |
| gianna4.071 | gianna | 1 | 4.07 | ghiozza | 0 | 0 | 6 | f | f | 0.09523805 | 0.1547619 | 0.017094017 | PRE |
| gigetto1.41 | gigetto | 1 | 1.4 | gessica | only heard | 0 | 2 | m | f | 0.06060606 | 0 | 0.039735099 | POST |
| gigetto1.41 | gigetto | 1 | 1.4 | gatta | only heard | 0 | 2 | m | f | 0.52272727 | 0.0681818 | 0.016528926 | POST |
| gigetto1.5311 | gigetto | 1 | 1.531 | gatta | seen and heard | 0 | 3 | m | f | 0.52272727 | 0.0681818 | 0.016528926 | POST |
| gigetto1.5311 | gigetto | 1 | 1.531 | ghirlanda | only heard | 0 | 3 | m | f | 0 | 0 | 0.023148148 | POST |
| gigetto1.5311 | gigetto | 1 | 1.531 | gianna | only heard | 0 | 3 | m | f | 0 | 0.13157894 | 0.03163017 | POST |
| gigetto1.821 | gigetto | 1 | 1.82 | gessica | 0 | 0 | 2 | m | f | 0.06060606 | 0 | 0.039735099 | POST |
| gigetto1.821 | gigetto | 1 | 1.82 | gatta | 0 | 0 | 2 | m | f | 0.52272727 | 0.0681818 | 0.016528926 | POST |
| gigetto2.021 | gigetto | 1 | 2.02 | giangi | 0 | 0 | 6 | m | m | 0 | 0 | 0.215686275 | POST |
| gigetto2.121 | gigetto | 1 | 2.12 | giangi | only seen | 1 | 2 | m | m | 0 | 0 | 0.215686275 | POST |
| gigetto2.121 | gigetto | 1 | 2.12 | gessica | 0 | 0 | 2 | m | f | 0.06060606 | 0 | 0.039735099 | POST |
| gigetto2.291 | gigetto | 1 | 2.29 | giangi | 0 | 1 | 5 | m | m | 0 | 0 | 0.215686275 | POST |
| gigetto2.291 | gigetto | 1 | 2.29 | ghirlanda | 0 | 0 | 5 | m | f | 0 | 0 | 0.023148148 | POST |
| gigetto2.291 | gigetto | 1 | 2.29 | gessica | 0 | 0 | 5 | m | f | 0.06060606 | 0 | 0.039735099 | POST |
| gigetto2.291 | gigetto | 1 | 2.29 | ghiozza | 0 | 0 | 5 | m | f | 0.15 | 0.125 | 0.017094017 | POST |
| gigetto2.291 | gigetto | 1 | 2.29 | gianna | 0 | 0 | 5 | m | f | 0 | 0.13157894 | 0.03163017 | POST |
| gigetto3.141 | gigetto | 1 | 3.14 | gatta | seen and heard | 0 | 5 | m | f | 0.52272727 | 0.0681818 | 0.016528926 | POST |
| gigetto3.141 | gigetto | 1 | 3.14 | gessica | seen and heard | 0 | 5 | m | f | 0.06060606 | 0 | 0.039735099 | POST |
| gigetto3.141 | gigetto | 1 | 3.14 | ghirlanda | only heard | 0 | 5 | m | f | 0 | 0 | 0.023148148 | POST |
| gigetto3.141 | gigetto | 1 | 3.14 | ghiozza | only heard | 0 | 5 | m | f | 0.15 | 0.125 | 0.017094017 | POST |
| gigetto3.141 | gigetto | 1 | 3.14 | gianna | only heard | 0 | 5 | m | f | 0 | 0.13157894 | 0.03163017 | POST |
| grappolo1.141 | grappolo | 1 | 1.14 | gessica | 0 | 0 | 7 | f | f | 0 | 0.15384615 | 0.039735099 | POST |
| grappolo1.141 | grappolo | 1 | 1.14 | gatta | 0 | 0 | 7 | f | f | 0.022727273 | 0.159090909 | 0.016528926 | POST |
| grappolo1.141 | grappolo | 1 | 1.14 | gianna | 0 | 0 | 7 | f | f | 0.05434782 | 0.14130434 | 0.03163017 | POST |
| grappolo1.141 | grappolo | 1 | 1.14 | giangi | 0 | 0 | 7 | f | m | 0.14529914 | 0.05982904 | 0.215686275 | POST |
| grappolo1.141 | grappolo | 1 | 1.14 | ghiozza | 0 | 0 | 7 | f | f | 0.11764705 | 0.11764705 | 0.017094017 | POST |
| grappolo1.141 | grappolo | 1 | 1.14 | ghirlanda | 0 | 0 | 7 | f | f | 0 | 0.13483146 | 0.023148148 | POST |
| grappolo1.141 | grappolo | 1 | 1.14 | gelly | 0 | 0 | 7 | f | f | 0.15384613 | 0.11538461 | 0 | POST |
| grappolo1.323 | grappolo | 3 | 1.32 | giangi | only seen | 0 | 2 | f | m | 0.14529914 | 0.05982904 | 0.215686275 | POST |
| grappolo1.323 | grappolo | 3 | 1.32 | gianna | 0 | 0 | 2 | f | f | 0.05434782 | 0.14130434 | 0.03163017 | POST |
| grappolo1.381 | grappolo | 1 | 1.38 | gianna | 0 | 0 | 5 | f | f | 0.05434782 | 0.14130434 | 0.03163017 | POST |
| grappolo1.381 | grappolo | 1 | 1.38 | gessica | 0 | 0 | 5 | f | f | 0 | 0.15384615 | 0.039735099 | POST |
| grappolo1.381 | grappolo | 1 | 1.38 | ghirlanda | 0 | 0 | 5 | f | f | 0 | 0.13483146 | 0.023148148 | POST |
| grappolo1.381 | grappolo | 1 | 1.38 | gatta | 0 | 0 | 5 | f | f | 0.022727273 | 0.159090909 | 0.016528926 | POST |
| grappolo1.381 | grappolo | 1 | 1.38 | ghiozza | 0 | 0 | 5 | f | f | 0.11764705 | 0.11764705 | 0.017094017 | POST |
| grappolo1.691 | grappolo | 1 | 1.69 | giangi | 0 | 0 | 3 | f | m | 0.14529914 | 0.05982904 | 0.215686275 | POST |
| grappolo1.691 | grappolo | 1 | 1.69 | gianna | 0 | 1 | 3 | f | f | 0.05434782 | 0.14130434 | 0.03163017 | POST |
| grappolo1.691 | grappolo | 1 | 1.69 | ghirlanda | 0 | 0 | 3 | f | f | 0 | 0.13483146 | 0.023148148 | POST |
| grappolo1.773 | grappolo | 3 | 1.77 | giangi | 0 | 0 | 1 | f | m | 0.14529914 | 0.05982904 | 0.215686275 | POST |
| grappolo1.853 | grappolo | 3 | 1.85 | gelly | only seen | 0 | 1 | f | f | 0.15384613 | 0.11538461 | 0 | POST |
| grappolo1.931 | grappolo | 1 | 1.93 | gatta | seen and heard | 0 | 5 | f | f | 0.022727273 | 0.159090909 | 0.016528926 | POST |
| grappolo1.931 | grappolo | 1 | 1.93 | gianna | seen and heard | 0 | 5 | f | f | 0.05434782 | 0.14130434 | 0.03163017 | POST |
| grappolo1.931 | grappolo | 1 | 1.93 | ghirlanda | seen and heard | 0 | 5 | f | f | 0 | 0.13483146 | 0.023148148 | POST |
| grappolo1.931 | grappolo | 1 | 1.93 | gessica | seen and heard | 0 | 5 | f | f | 0 | 0.15384615 | 0.039735099 | POST |
| grappolo1.931 | grappolo | 1 | 1.93 | giangi | 0 | 0 | 5 | f | m | 0.14529914 | 0.05982904 | 0.215686275 | POST |
| grappolo2.083 | grappolo | 3 | 2.08 | gatta | only seen | 1 | 5 | f | f | 0.022727273 | 0.159090909 | 0.016528926 | POST |
| grappolo2.083 | grappolo | 3 | 2.08 | ghirlanda | only seen | 0 | 5 | f | f | 0 | 0.13483146 | 0.023148148 | POST |
| grappolo2.083 | grappolo | 3 | 2.08 | giangi | 0 | 0 | 5 | f | m | 0.14529914 | 0.05982904 | 0.215686275 | POST |
| grappolo2.083 | grappolo | 3 | 2.08 | gessica | 0 | 0 | 5 | f | f | 0 | 0.15384615 | 0.039735099 | POST |
| grappolo2.083 | grappolo | 3 | 2.08 | gianna | 0 | 0 | 5 | f | f | 0.05434782 | 0.14130434 | 0.03163017 | POST |
| grappolo2.383 | grappolo | 3 | 2.38 | gatta | 0 | 0 | 3 | f | f | 0.022727273 | 0.159090909 | 0.016528926 | POST |
| grappolo2.383 | grappolo | 3 | 2.38 | gigetto | 0 | 0 | 3 | f | m | 0.05714285 | 0.02857142 | 0.039548023 | POST |
| grappolo2.383 | grappolo | 3 | 2.38 | gessica | 0 | 0 | 3 | f | f | 0 | 0.15384615 | 0.039735099 | POST |
| grappolo2.393 | grappolo | 3 | 2.39 | gessica | 0 | 0 | 3 | f | f | 0 | 0.15384615 | 0.039735099 | POST |
| grappolo2.393 | grappolo | 3 | 2.39 | giangi | 0 | 0 | 3 | f | m | 0.14529914 | 0.05982904 | 0.215686275 | POST |
| grappolo2.393 | grappolo | 3 | 2.39 | gatta | 0 | 0 | 3 | f | f | 0.022727273 | 0.159090909 | 0.016528926 | POST |
| grappolo2.481 | grappolo | 1 | 2.48 | giangi | only seen | 0 | 5 | f | m | 0.14529914 | 0.05982904 | 0.215686275 | POST |
| grappolo2.481 | grappolo | 1 | 2.48 | gessica | 0 | 0 | 5 | f | f | 0 | 0.15384615 | 0.039735099 | POST |
| grappolo2.481 | grappolo | 1 | 2.48 | gianna | 0 | 0 | 5 | f | f | 0.05434782 | 0.14130434 | 0.03163017 | POST |
| grappolo2.481 | grappolo | 1 | 2.48 | gatta | 0 | 0 | 5 | f | f | 0.022727273 | 0.159090909 | 0.016528926 | POST |
| grappolo2.481 | grappolo | 1 | 2.48 | ghiozza | 0 | 0 | 5 | f | f | 0.11764705 | 0.11764705 | 0.017094017 | POST |
| grappolo2.683 | grappolo | 3 | 2.68 | gigio | only seen | 0 | 7 | f | m | 0.05128205 | 0.02564102 | 0 | POST |
| grappolo2.683 | grappolo | 3 | 2.68 | ghirlanda | only seen | 1 | 7 | f | f | 0 | 0.13483146 | 0.023148148 | POST |
| grappolo2.683 | grappolo | 3 | 2.68 | giangi | 0 | 1 | 7 | f | m | 0.14529914 | 0.05982904 | 0.215686275 | POST |
| grappolo2.683 | grappolo | 3 | 2.68 | gianna | 0 | 0 | 7 | f | f | 0.05434782 | 0.14130434 | 0.03163017 | POST |
| grappolo2.683 | grappolo | 3 | 2.68 | gessica | 0 | 0 | 7 | f | f | 0 | 0.15384615 | 0.039735099 | POST |
| grappolo2.683 | grappolo | 3 | 2.68 | gatta | 0 | 0 | 7 | f | f | 0.022727273 | 0.159090909 | 0.016528926 | POST |
| grappolo2.683 | grappolo | 3 | 2.68 | ghiozza | 0 | 0 | 7 | f | f | 0.11764705 | 0.11764705 | 0.017094017 | POST |
| grappolo2.71 | grappolo | 1 | 2.7 | gigetto | only seen | 0 | 7 | f | m | 0.05714285 | 0.02857142 | 0.039548023 | POST |
| grappolo2.71 | grappolo | 1 | 2.7 | gianna | only seen | 0 | 7 | f | f | 0.05434782 | 0.14130434 | 0.03163017 | POST |
| grappolo2.71 | grappolo | 1 | 2.7 | gigio | 0 | 0 | 7 | f | m | 0.05128205 | 0.02564102 | 0 | POST |
| grappolo2.71 | grappolo | 1 | 2.7 | giangi | 0 | 0 | 7 | f | m | 0.14529914 | 0.05982904 | 0.215686275 | POST |
| grappolo2.71 | grappolo | 1 | 2.7 | gessica | 0 | 0 | 7 | f | f | 0 | 0.15384615 | 0.039735099 | POST |
| grappolo2.71 | grappolo | 1 | 2.7 | gatta | 0 | 0 | 7 | f | f | 0.022727273 | 0.159090909 | 0.016528926 | POST |
| grappolo2.71 | grappolo | 1 | 2.7 | ghirlanda | 0 | 0 | 7 | f | f | 0 | 0.13483146 | 0.023148148 | POST |
| grappolo2.713 | grappolo | 3 | 2.71 | ghirlanda | only seen | 0 | 4 | f | f | 0 | 0.13483146 | 0.023148148 | POST |
| grappolo2.713 | grappolo | 3 | 2.71 | gatta | only seen | 0 | 4 | f | f | 0.022727273 | 0.159090909 | 0.016528926 | POST |
| grappolo2.713 | grappolo | 3 | 2.71 | gessica | only seen | 0 | 4 | f | f | 0 | 0.15384615 | 0.039735099 | POST |
| grappolo2.713 | grappolo | 3 | 2.71 | gianna | 0 | 0 | 4 | f | f | 0.05434782 | 0.14130434 | 0.03163017 | POST |
| grappolo3.253 | grappolo | 3 | 3.25 | giangi | 0 | 0 | 4 | f | m | 0.14529914 | 0.05982904 | 0.215686275 | POST |
| grappolo3.253 | grappolo | 3 | 3.25 | ghiozza | 0 | 0 | 4 | f | f | 0.11764705 | 0.11764705 | 0.017094017 | POST |
| grappolo3.253 | grappolo | 3 | 3.25 | gianna | 0 | 0 | 4 | f | f | 0.05434782 | 0.14130434 | 0.03163017 | POST |
| grappolo3.253 | grappolo | 3 | 3.25 | gatta | 0 | 0 | 4 | f | f | 0.022727273 | 0.159090909 | 0.016528926 | POST |
| grappolo3.43 | grappolo | 3 | 3.4 | giangi | 0 | 0 | 4 | f | m | 0.14529914 | 0.05982904 | 0.215686275 | POST |
| grappolo3.43 | grappolo | 3 | 3.4 | ghiozza | 0 | 0 | 4 | f | f | 0.11764705 | 0.11764705 | 0.017094017 | POST |
| grappolo3.43 | grappolo | 3 | 3.4 | gianna | 0 | 0 | 4 | f | f | 0.05434782 | 0.14130434 | 0.03163017 | POST |
| grappolo3.43 | grappolo | 3 | 3.4 | gatta | 0 | 0 | 4 | f | f | 0.022727273 | 0.159090909 | 0.016528926 | POST |
| grappolo3.653 | grappolo | 3 | 3.65 | giangi | 0 | 0 | 1 | f | m | 0.14529914 | 0.05982904 | 0.215686275 | POST |
| grappolo3.762 | grappolo | 2 | 3.76 | ghirlanda | only seen | 0 | 4 | f | f | 0 | 0.13483146 | 0.023148148 | POST |
| grappolo3.762 | grappolo | 2 | 3.76 | gianna | 0 | 0 | 4 | f | f | 0.05434782 | 0.14130434 | 0.03163017 | POST |
| grappolo3.762 | grappolo | 2 | 3.76 | giangi | 0 | 0 | 4 | f | m | 0.14529914 | 0.05982904 | 0.215686275 | POST |
| grappolo3.762 | grappolo | 2 | 3.76 | gessica | 0 | 0 | 4 | f | f | 0 | 0.15384615 | 0.039735099 | POST |
| grappolo3.863 | grappolo | 3 | 3.86 | gatta | only seen | 0 | 5 | f | f | 0.022727273 | 0.159090909 | 0.016528926 | POST |
| grappolo3.863 | grappolo | 3 | 3.86 | ghirlanda | only seen | 0 | 5 | f | f | 0 | 0.13483146 | 0.023148148 | POST |
| grappolo3.863 | grappolo | 3 | 3.86 | gianna | 0 | 0 | 5 | f | f | 0.05434782 | 0.14130434 | 0.03163017 | POST |
| grappolo3.863 | grappolo | 3 | 3.86 | giangi | 0 | 0 | 5 | f | m | 0.14529914 | 0.05982904 | 0.215686275 | POST |
| grappolo3.863 | grappolo | 3 | 3.86 | gessica | 0 | 0 | 5 | f | f | 0 | 0.15384615 | 0.039735099 | POST |
| grappolo4.371 | grappolo | 1 | 4.37 | giangi | only heard | 0 | 4 | f | m | 0.14529914 | 0.05982904 | 0.215686275 | POST |
| grappolo4.371 | grappolo | 1 | 4.37 | ghiozza | only heard | 0 | 4 | f | f | 0.11764705 | 0.11764705 | 0.017094017 | POST |
| grappolo4.371 | grappolo | 1 | 4.37 | gianna | only heard | 0 | 4 | f | f | 0.05434782 | 0.14130434 | 0.03163017 | POST |
| grappolo4.371 | grappolo | 1 | 4.37 | gatta | only heard | 0 | 4 | f | f | 0.022727273 | 0.159090909 | 0.016528926 | PRE |
| grappolo7.073 | grappolo | 3 | 7.07 | giangi | 0 | 0 | 4 | f | m | 0.14529914 | 0.05982904 | 0.215686275 | PRE |
| grappolo7.073 | grappolo | 3 | 7.07 | ghiozza | 0 | 0 | 4 | f | f | 0.11764705 | 0.11764705 | 0.017094017 | PRE |
| grappolo7.073 | grappolo | 3 | 7.07 | gianna | 0 | 0 | 4 | f | f | 0.05434782 | 0.14130434 | 0.03163017 | PRE |
| grappolo7.073 | grappolo | 3 | 7.07 | gatta | 0 | 0 | 4 | f | f | 0.022727273 | 0.159090909 | 0.016528926 | PRE |
| neomamma1.471 | neomamma | 1 | 1.47 | spot | only seen | 0 | 2 | f | f | 0.16806722 | 0.18487394 | 0.020576132 | POST |
| neomamma1.471 | neomamma | 1 | 1.47 | rosa | only seen | 0 | 2 | f | f | 0 | 0.18032786 | 0.027131783 | POST |
| neomamma1.621 | neomamma | 1 | 1.62 | spot | 0 | 0 | 3 | f | f | 0.16806722 | 0.18487394 | 0.020576132 | POST |
| neomamma1.621 | neomamma | 1 | 1.62 | roll | 0 | 0 | 3 | f | f | 0.111111111 | 0.155555556 | 0.036781609 | POST |
| neomamma1.621 | neomamma | 1 | 1.62 | sciura | 0 | 0 | 3 | f | f | 0.16 | 0.21333333 | 0.06377858 | POST |
| neomamma1.721 | neomamma | 1 | 1.72 | sine | only seen | 0 | 2 | f | f | 0.0420168 | 0.20168067 | 0.050682261 | POST |
| neomamma1.721 | neomamma | 1 | 1.72 | schiarita | only seen | 0 | 2 | f | f | 0 | 0.10091743 | 0.041420118 | POST |
| neomamma1.721 | neomamma | 1 | 1.72 | small | only seen | 0 | 2 | f | f | 0.03846153 | 0.13461538 | 0.044117647 | POST |
| neomamma1.721 | neomamma | 1 | 1.72 | spiga | 0 | 0 | 2 | f | f | 0.14150943 | 0.16981132 | 0.018372703 | POST |
| neomamma1.721 | neomamma | 1 | 1.72 | rosa | 0 | 0 | 2 | f | f | 0 | 0.18032786 | 0.027131783 | POST |
| neomamma1.922 | neomamma | 2 | 1.92 | severa | 0 | 0 | 8 | f | f | 0.14285714 | 0.19047619 | 0.066176471 | POST |
| neomamma1.922 | neomamma | 2 | 1.92 | tris | 0 | 0 | 8 | f | f | 0 | 0 | 0.010449321 | POST |
| neomamma1.922 | neomamma | 2 | 1.92 | spilla | 0 | 0 | 8 | f | f | 0 | 0.05555556 | 0.043678161 | POST |
| neomamma1.922 | neomamma | 2 | 1.92 | rosa | 0 | 0 | 8 | f | f | 0 | 0.18032786 | 0.027131783 | POST |
| neomamma1.922 | neomamma | 2 | 1.92 | schiarita | 0 | 0 | 8 | f | f | 0 | 0.10091743 | 0.041420118 | POST |
| neomamma1.922 | neomamma | 2 | 1.92 | osso | 0 | 0 | 8 | f | f | 0 | 0 | 0.03030303 | POST |
| neomamma1.922 | neomamma | 2 | 1.92 | stella | 0 | 0 | 8 | f | f | 0.02325581 | 0.06976744 | 0.020080321 | POST |
| neomamma1.922 | neomamma | 2 | 1.92 | sfasciato | 0 | 0 | 8 | f | m | 0 | 0.1 | 0.024390244 | POST |
| neomamma1.941 | neomamma | 1 | 1.94 | roll | only seen | 0 | 3 | f | f | 0.111111111 | 0.155555556 | 0.036781609 | POST |
| neomamma1.941 | neomamma | 1 | 1.94 | ruga | 0 | 0 | 3 | f | f | 0 | 0.20689655 | 0.004608295 | POST |
| neomamma1.941 | neomamma | 1 | 1.94 | stella | 0 | 0 | 3 | f | f | 0.02325581 | 0.06976744 | 0.020080321 | POST |
| neomamma1.971 | neomamma | 1 | 1.97 | scapola | only seen | 0 | 8 | f | f | 0 | 0.12820512 | 0.003787879 | POST |
| neomamma1.971 | neomamma | 1 | 1.97 | stacy | only seen | 0 | 8 | f | f | 0.01904761 | 0.19047619 | 0.021317829 | POST |
| neomamma1.971 | neomamma | 1 | 1.97 | schiarita | 0 | 0 | 8 | f | f | 0 | 0.10091743 | 0.041420118 | PRE |
| neomamma1.971 | neomamma | 1 | 1.97 | stella | 0 | 0 | 8 | f | f | 0.02325581 | 0.06976744 | 0.020080321 | PRE |
| neomamma1.971 | neomamma | 1 | 1.97 | spilla | 0 | 0 | 8 | f | f | 0 | 0.05555556 | 0.043678161 | PRE |
| neomamma1.971 | neomamma | 1 | 1.97 | sorcia | 0 | 0 | 8 | f | f | 0 | 0 | 0.022727273 | PRE |
| neomamma1.971 | neomamma | 1 | 1.97 | spot | 0 | 0 | 8 | f | f | 0.16806722 | 0.18487394 | 0.020576132 | PRE |
| neomamma1.971 | neomamma | 1 | 1.97 | spiga | 0 | 0 | 8 | f | f | 0.14150943 | 0.16981132 | 0.018372703 | PRE |
| neomamma2.491 | neomamma | 1 | 2.49 | osso | seen and heard | 0 | 7 | f | f | 0 | 0 | 0.03030303 | PRE |
| neomamma2.491 | neomamma | 1 | 2.49 | sfasciato | only heard | 0 | 7 | f | m | 0 | 0.1 | 0.024390244 | PRE |
| neomamma2.491 | neomamma | 1 | 2.49 | rosa | only heard | 1 | 7 | f | f | 0 | 0.18032786 | 0.027131783 | PRE |
| neomamma2.491 | neomamma | 1 | 2.49 | schiarita | only heard | 1 | 7 | f | f | 0 | 0.10091743 | 0.041420118 | PRE |
| neomamma2.491 | neomamma | 1 | 2.49 | severa | only heard | 0 | 7 | f | f | 0.14285714 | 0.19047619 | 0.066176471 | PRE |
| neomamma2.491 | neomamma | 1 | 2.49 | spilla | only heard | 0 | 7 | f | f | 0 | 0.05555556 | 0.043678161 | PRE |
| neomamma2.491 | neomamma | 1 | 2.49 | stella | only heard | 1 | 7 | f | f | 0.02325581 | 0.06976744 | 0.020080321 | PRE |
| neomamma2.591 | neomamma | 1 | 2.59 | roll | 0 | 0 | 2 | f | f | 0.111111111 | 0.155555556 | 0.036781609 | PRE |
| neomamma2.591 | neomamma | 1 | 2.59 | sciura | 0 | 0 | 2 | f | f | 0.16 | 0.21333333 | 0.06377858 | PRE |
| neomamma3.323 | neomamma | 3 | 3.32 | spiga | only seen | 1 | 1 | f | f | 0.14150943 | 0.16981132 | 0.018372703 | PRE |
| neomamma3.891 | neomamma | 1 | 3.89 | sonia | only seen | 1 | 4 | f | f | 0 | 0.155555556 | 0.041666667 | PRE |
| neomamma3.891 | neomamma | 1 | 3.89 | ruga | only seen | 0 | 4 | f | f | 0 | 0.20689655 | 0.004608295 | PRE |
| neomamma3.891 | neomamma | 1 | 3.89 | stella | 0 | 0 | 4 | f | f | 0.02325581 | 0.06976744 | 0.020080321 | PRE |
| neomamma3.891 | neomamma | 1 | 3.89 | roll | 0 | 0 | 4 | f | f | 0.111111111 | 0.155555556 | 0.036781609 | PRE |
| neomamma6.923 | neomamma | 3 | 6.92 | spot | 0 | 0 | 2 | f | f | 0.16806722 | 0.18487394 | 0.020576132 | PRE |
| neomamma6.923 | neomamma | 3 | 6.92 | stacy | 0 | 0 | 2 | f | f | 0.01904761 | 0.19047619 | 0.021317829 | PRE |
| osso1.191 | osso | 1 | 1.19 | severa | only heard | 1 | 1 | f | f | 0.27536231 | 0.11594202 | 0.066176471 | POST |
| osso1.221 | osso | 1 | 1.22 | spilla | only seen | 0 | 2 | f | f | 0.08064516 | 0.27419354 | 0.043678161 | POST |
| osso1.221 | osso | 1 | 1.22 | severa | 0 | 0 | 2 | f | f | 0.27536231 | 0.11594202 | 0.066176471 | POST |
| osso1.361 | osso | 1 | 1.36 | sciura | only seen | 0 | 1 | f | f | 0.15384615 | 0.03846153 | 0.06377858 | POST |
| osso1.411 | osso | 1 | 1.41 | severa | only seen | 0 | 6 | f | f | 0.27536231 | 0.11594202 | 0.066176471 | POST |
| osso1.411 | osso | 1 | 1.41 | sciura | 0 | 0 | 6 | f | f | 0.15384615 | 0.03846153 | 0.06377858 | POST |
| osso1.411 | osso | 1 | 1.41 | tris | 0 | 0 | 6 | f | f | 0.03773584 | 0.14150943 | 0.010449321 | POST |
| osso1.411 | osso | 1 | 1.41 | spilla | 0 | 0 | 6 | f | f | 0.08064516 | 0.27419354 | 0.043678161 | POST |
| osso1.411 | osso | 1 | 1.41 | sonia | 0 | 0 | 6 | f | f | 0 | 0.19047619 | 0.041666667 | POST |
| osso1.411 | osso | 1 | 1.41 | stella | 0 | 0 | 6 | f | f | 0 | 0 | 0.020080321 | POST |
| osso1.41* | osso | * | 1.41 | spilla | 0 | 0 | 3 | f | f | 0.08064516 | 0.27419354 | 0.043678161 | POST |
| osso1.41* | osso | * | 1.41 | sorcia | 0 | 0 | 3 | f | f | 0 | 0.07692307 | 0.022727273 | POST |
| osso1.421 | osso | 1 | 1.42 | severa | seen and heard | 1 | 8 | f | f | 0.27536231 | 0.11594202 | 0.066176471 | POST |
| osso1.421 | osso | 1 | 1.42 | spilla | seen and heard | 0 | 8 | f | f | 0.08064516 | 0.27419354 | 0.043678161 | POST |
| osso1.421 | osso | 1 | 1.42 | sorcia | only heard | 0 | 8 | f | f | 0 | 0.07692307 | 0.022727273 | POST |
| osso1.421 | osso | 1 | 1.42 | sonia | only heard | 0 | 8 | f | f | 0 | 0.19047619 | 0.041666667 | POST |
| osso1.421 | osso | 1 | 1.42 | roll | only heard | 0 | 8 | f | f | 0.0117647 | 0.15294117 | 0.036781609 | POST |
| osso1.421 | osso | 1 | 1.42 | ruga | only heard | 0 | 8 | f | f | 0.01369863 | 0.09589041 | 0.004608295 | POST |
| osso1.421 | osso | 1 | 1.42 | sciura | only heard | 0 | 8 | f | f | 0.15384615 | 0.03846153 | 0.06377858 | POST |
| osso1.421 | osso | 1 | 1.42 | sguercia | only heard | 0 | 8 | f | f | 0 | 0.125 | 0 | POST |
| osso1.531 | osso | 1 | 1.53 | severa | only seen | 1 | 2 | f | f | 0.27536231 | 0.11594202 | 0.066176471 | POST |
| osso1.531 | osso | 1 | 1.53 | tris | 0 | 0 | 2 | f | f | 0.03773584 | 0.14150943 | 0.010449321 | POST |
| osso1.611 | osso | 1 | 1.61 | severa | only seen | 0 | 3 | f | f | 0.27536231 | 0.11594202 | 0.066176471 | PRE |
| osso1.611 | osso | 1 | 1.61 | ruga | 0 | 0 | 3 | f | f | 0.01369863 | 0.09589041 | 0.004608295 | PRE |
| osso1.611 | osso | 1 | 1.61 | sciura | 0 | 0 | 3 | f | f | 0.15384615 | 0.03846153 | 0.06377858 | PRE |
| osso1.663 | osso | 3 | 1.66 | roll | 0 | 0 | 8 | f | f | 0.0117647 | 0.15294117 | 0.036781609 | PRE |
| osso1.663 | osso | 3 | 1.66 | sciura | 0 | 0 | 8 | f | f | 0.15384615 | 0.03846153 | 0.06377858 | PRE |
| osso1.663 | osso | 3 | 1.66 | stacy | 0 | 0 | 8 | f | f | 0 | 0 | 0.021317829 | PRE |
| osso1.663 | osso | 3 | 1.66 | stella | 0 | 0 | 8 | f | f | 0 | 0 | 0.020080321 | PRE |
| osso1.663 | osso | 3 | 1.66 | sfasciato | 0 | 0 | 8 | f | m | 0 | 0.25 | 0.024390244 | PRE |
| osso1.663 | osso | 3 | 1.66 | secco | 0 | 0 | 8 | f | m | 0 | 0 | 0.123076923 | PRE |
| osso1.663 | osso | 3 | 1.66 | ruga | 0 | 0 | 8 | f | f | 0.01369863 | 0.09589041 | 0.004608295 | PRE |
| osso1.663 | osso | 3 | 1.66 | sonia | 0 | 0 | 8 | f | f | 0 | 0.19047619 | 0.041666667 | PRE |
| osso1.881 | osso | 1 | 1.88 | severa | 0 | 0 | 1 | f | f | 0.27536231 | 0.11594202 | 0.066176471 | PRE |
| osso2.51 | osso | 1 | 2.5 | tris | only seen | 0 | 3 | f | f | 0.03773584 | 0.14150943 | 0.010449321 | PRE |
| osso2.53 | osso | 3 | 2.5 | tris | only seen | 0 | 4 | f | f | 0.03773584 | 0.14150943 | 0.010449321 | PRE |
| osso2.51 | osso | 1 | 2.5 | sonia | 0 | 0 | 3 | f | f | 0 | 0.19047619 | 0.041666667 | PRE |
| osso2.51 | osso | 1 | 2.5 | severa | 0 | 0 | 3 | f | f | 0.27536231 | 0.11594202 | 0.066176471 | PRE |
| osso2.53 | osso | 3 | 2.5 | stella | 0 | 1 | 4 | f | f | 0 | 0 | 0.020080321 | PRE |
| osso2.53 | osso | 3 | 2.5 | sorcia | 0 | 0 | 4 | f | f | 0 | 0.07692307 | 0.022727273 | PRE |
| osso3.093 | osso | 3 | 3.09 | severa | only seen | 0 | 1 | f | f | 0.27536231 | 0.11594202 | 0.066176471 | PRE |
| rocco1.005* | rocco | * | 1.005 | osso | 0 | 0 | 5 | m | f | 0.21153846 | 0.21153846 | 0.03030303 | POST |
| rocco1.005* | rocco | * | 1.005 | ruga | 0 | 0 | 5 | m | f | 0.12698412 | 0.2222222 | 0.004608295 | POST |
| rocco1.005* | rocco | * | 1.005 | scapola | 0 | 0 | 5 | m | f | 0 | 0 | 0.003787879 | POST |
| rocco1.005* | rocco | * | 1.005 | roll | 0 | 0 | 5 | m | f | 0.152777778 | 0.191489362 | 0.036781609 | POST |
| rocco1.011 | rocco | 1 | 1.01 | tris | seen and heard | 1 | 5 | m | f | 0.10144928 | 0.23188405 | 0.010449321 | POST |
| rocco1.011 | rocco | 1 | 1.01 | scapola | only heard | 0 | 5 | m | f | 0 | 0 | 0.003787879 | POST |
| rocco1.011 | rocco | 1 | 1.01 | osso | only heard | 1 | 5 | m | f | 0.21153846 | 0.21153846 | 0.03030303 | POST |
| rocco1.011 | rocco | 1 | 1.01 | ruga | only heard | 0 | 5 | m | f | 0.12698412 | 0.2222222 | 0.004608295 | POST |
| rocco1.011 | rocco | 1 | 1.01 | roll | only heard | 0 | 5 | m | f | 0.152777778 | 0.191489362 | 0.036781609 | POST |
| rocco1.0661 | rocco | 1 | 1.066 | tris | seen and heard | 0 | 4 | m | f | 0.10144928 | 0.23188405 | 0.010449321 | POST |
| rocco1.0661 | rocco | 1 | 1.066 | roll | only heard | 0 | 4 | m | f | 0.152777778 | 0.191489362 | 0.036781609 | POST |
| rocco1.0661 | rocco | 1 | 1.066 | ruga | only heard | 0 | 4 | m | f | 0.12698412 | 0.2222222 | 0.004608295 | POST |
| rocco1.0661 | rocco | 1 | 1.066 | scapola | only heard | 0 | 4 | m | f | 0 | 0 | 0.003787879 | POST |
| rocco1.2333 | rocco | 3 | 1.233 | osso | seen and heard | 0 | 5 | m | f | 0.21153846 | 0.21153846 | 0.03030303 | POST |
| rocco1.2333 | rocco | 3 | 1.233 | ruga | seen and heard | 0 | 5 | m | f | 0.12698412 | 0.2222222 | 0.004608295 | POST |
| rocco1.2333 | rocco | 3 | 1.233 | scapola | only heard | 0 | 5 | m | f | 0 | 0 | 0.003787879 | POST |
| rocco1.2333 | rocco | 3 | 1.233 | tris | only heard | 0 | 5 | m | f | 0.10144928 | 0.23188405 | 0.010449321 | POST |
| rocco1.2333 | rocco | 3 | 1.233 | roll | only heard | 0 | 5 | m | f | 0.045454545 | 0.1818182 | 0.036781609 | POST |
| rocco1.2451 | rocco | 1 | 1.245 | osso | seen and heard | 0 | 7 | m | f | 0.21153846 | 0.21153846 | 0.03030303 | POST |
| rocco1.2451 | rocco | 1 | 1.245 | roll | only heard | 0 | 7 | m | f | 0.045454545 | 0.1818182 | 0.036781609 | POST |
| rocco1.2451 | rocco | 1 | 1.245 | ruga | only heard | 0 | 7 | m | f | 0.12698412 | 0.2222222 | 0.004608295 | POST |
| rocco1.2451 | rocco | 1 | 1.245 | tinino1 | only heard | 0 | 7 | m | m | 0 | 0 | 0.021505376 | POST |
| rocco1.2451 | rocco | 1 | 1.245 | tris | only heard | 0 | 7 | m | f | 0.10144928 | 0.23188405 | 0.010449321 | POST |
| rocco1.2451 | rocco | 1 | 1.245 | scapola | only heard | 0 | 7 | m | f | 0 | 0 | 0.003787879 | POST |
| rocco1.2451 | rocco | 1 | 1.245 | sandro | only heard | 1 | 7 | m | m | 0 | 0.09375 | 0.089430894 | POST |
| rocco1.3113 | rocco | 3 | 1.311 | sandro | seen and heard | 0 | 7 | m | m | 0 | 0.09375 | 0.089430894 | POST |
| rocco1.3113 | rocco | 3 | 1.311 | roll | seen and heard | 0 | 7 | m | f | 0.152777778 | 0.191489362 | 0.036781609 | POST |
| rocco1.3113 | rocco | 3 | 1.311 | tinino2 | only heard | 1 | 7 | m | m | 0 | 0 | 0.037037037 | POST |
| rocco1.3113 | rocco | 3 | 1.311 | tinino1 | only heard | 0 | 7 | m | m | 0 | 0 | 0.021505376 | POST |
| rocco1.3113 | rocco | 3 | 1.311 | scapola | only heard | 0 | 7 | m | f | 0 | 0 | 0.003787879 | POST |
| rocco1.3113 | rocco | 3 | 1.311 | osso | only heard | 0 | 7 | m | f | 0.21153846 | 0.21153846 | 0.03030303 | POST |
| rocco1.3113 | rocco | 3 | 1.311 | tris | only heard | 0 | 7 | m | f | 0.10144928 | 0.23188405 | 0.010449321 | POST |
| rocco1.421 | rocco | 1 | 1.42 | tris | seen and heard | 1 | 6 | m | f | 0.10144928 | 0.23188405 | 0.010449321 | POST |
| rocco1.421 | rocco | 1 | 1.42 | tinino1 | only heard | 0 | 6 | m | m | 0 | 0 | 0.021505376 | POST |
| rocco1.421 | rocco | 1 | 1.42 | tinino2 | only heard | 0 | 6 | m | m | 0 | 0 | 0.037037037 | POST |
| rocco1.421 | rocco | 1 | 1.42 | scapola | only heard | 0 | 6 | m | f | 0 | 0 | 0.003787879 | POST |
| rocco1.421 | rocco | 1 | 1.42 | roll | only heard | 0 | 6 | m | f | 0.152777778 | 0.191489362 | 0.036781609 | POST |
| rocco1.491 | rocco | 1 | 1.49 | roll | seen and heard | 0 | 8 | m | f | 0.152777778 | 0.191489362 | 0.036781609 | POST |
| rocco1.491 | rocco | 1 | 1.49 | ruga | seen and heard | 0 | 8 | m | f | 0.12698412 | 0.2222222 | 0.004608295 | POST |
| rocco1.491 | rocco | 1 | 1.49 | tris | seen and heard | 0 | 8 | m | f | 0.10144928 | 0.23188405 | 0.010449321 | POST |
| rocco1.491 | rocco | 1 | 1.49 | tinino1 | only heard | 0 | 8 | m | m | 0 | 0 | 0.021505376 | POST |
| rocco1.491 | rocco | 1 | 1.49 | tinino2 | only heard | 0 | 8 | m | m | 0 | 0 | 0.037037037 | POST |
| rocco1.491 | rocco | 1 | 1.49 | sandro | only heard | 0 | 8 | m | m | 0 | 0.09375 | 0.089430894 | POST |
| rocco1.491 | rocco | 1 | 1.49 | scapola | only heard | 0 | 8 | m | f | 0 | 0 | 0.003787879 | POST |
| rocco1.491 | rocco | 1 | 1.49 | osso | only heard | 0 | 8 | m | f | 0.21153846 | 0.21153846 | 0.03030303 | POST |
| rocco1.511 | rocco | 1 | 1.51 | tris | seen and heard | 0 | 6 | m | f | 0.10144928 | 0.23188405 | 0.010449321 | POST |
| rocco1.511 | rocco | 1 | 1.51 | ruga | seen and heard | 0 | 6 | m | f | 0.12698412 | 0.2222222 | 0.004608295 | POST |
| rocco1.511 | rocco | 1 | 1.51 | sandro | seen and heard | 1 | 6 | m | m | 0 | 0.09375 | 0.089430894 | POST |
| rocco1.511 | rocco | 1 | 1.51 | scapola | only heard | 0 | 6 | m | f | 0 | 0 | 0.003787879 | POST |
| rocco1.511 | rocco | 1 | 1.51 | roll | only heard | 0 | 6 | m | f | 0.152777778 | 0.191489362 | 0.036781609 | POST |
| rocco1.511 | rocco | 1 | 1.51 | osso | only heard | 0 | 6 | m | f | 0.21153846 | 0.21153846 | 0.03030303 | POST |
| rocco1.631 | rocco | 1 | 1.63 | tris | seen and heard | 0 | 7 | m | f | 0.10144928 | 0.23188405 | 0.010449321 | POST |
| rocco1.631 | rocco | 1 | 1.63 | roll | seen and heard | 0 | 7 | m | f | 0.152777778 | 0.191489362 | 0.036781609 | POST |
| rocco1.631 | rocco | 1 | 1.63 | osso | seen and heard | 0 | 7 | m | f | 0.21153846 | 0.21153846 | 0.03030303 | POST |
| rocco1.631 | rocco | 1 | 1.63 | sandro | seen and heard | 1 | 7 | m | m | 0 | 0.09375 | 0.089430894 | POST |
| rocco1.631 | rocco | 1 | 1.63 | scapola | seen and heard | 0 | 7 | m | f | 0 | 0 | 0.003787879 | POST |
| rocco1.631 | rocco | 1 | 1.63 | tinino2 | seen and heard | 0 | 7 | m | m | 0 | 0 | 0.037037037 | POST |
| rocco1.631 | rocco | 1 | 1.63 | ruga | only heard | 0 | 7 | m | f | 0.12698412 | 0.2222222 | 0.004608295 | POST |
| rocco1.662 | rocco | 2 | 1.66 | osso | seen and heard | 0 | 4 | m | f | 0.21153846 | 0.21153846 | 0.03030303 | POST |
| rocco1.662 | rocco | 2 | 1.66 | ruga | seen and heard | 0 | 4 | m | f | 0.12698412 | 0.2222222 | 0.004608295 | POST |
| rocco1.662 | rocco | 2 | 1.66 | scapola | only heard | 0 | 4 | m | f | 0 | 0 | 0.003787879 | POST |
| rocco1.662 | rocco | 2 | 1.66 | tris | only heard | 0 | 4 | m | f | 0.10144928 | 0.23188405 | 0.010449321 | POST |
| rocco1.7231 | rocco | 1 | 1.723 | ruga | seen and heard | 0 | 5 | m | f | 0.12698412 | 0.2222222 | 0.004608295 | POST |
| rocco1.7231 | rocco | 1 | 1.723 | scapola | only heard | 0 | 5 | m | f | 0 | 0 | 0.003787879 | POST |
| rocco1.7231 | rocco | 1 | 1.723 | tris | only heard | 0 | 5 | m | f | 0.10144928 | 0.23188405 | 0.010449321 | POST |
| rocco1.7231 | rocco | 1 | 1.723 | osso | only heard | 1 | 5 | m | f | 0.21153846 | 0.21153846 | 0.03030303 | POST |
| rocco1.7422 | rocco | 2 | 1.742 | scapola | only heard | 0 | 4 | m | f | 0 | 0 | 0.003787879 | POST |
| rocco1.7422 | rocco | 2 | 1.742 | tris | only heard | 1 | 4 | m | f | 0.10144928 | 0.23188405 | 0.010449321 | POST |
| rocco1.7422 | rocco | 2 | 1.742 | ruga | only heard | 0 | 4 | m | f | 0.12698412 | 0.2222222 | 0.004608295 | POST |
| rocco1.8173 | rocco | 3 | 1.817 | tinino1 | seen and heard | 0 | 6 | m | m | 0 | 0 | 0.021505376 | POST |
| rocco1.8173 | rocco | 3 | 1.817 | roll | only heard | 0 | 6 | m | f | 0.045454545 | 0.1818182 | 0.036781609 | POST |
| rocco1.8173 | rocco | 3 | 1.817 | tris | only heard | 0 | 6 | m | f | 0.10144928 | 0.23188405 | 0.010449321 | POST |
| rocco1.8173 | rocco | 3 | 1.817 | scapola | only heard | 0 | 6 | m | f | 0 | 0 | 0.003787879 | POST |
| rocco1.8173 | rocco | 3 | 1.817 | ruga | only heard | 0 | 6 | m | f | 0.12698412 | 0.2222222 | 0.004608295 | POST |
| rocco1.8173 | rocco | 3 | 1.817 | osso | only heard | 0 | 6 | m | f | 0.21153846 | 0.21153846 | 0.03030303 | POST |
| rocco1.8233 | rocco | 3 | 1.823 | osso | seen and heard | 0 | 7 | m | f | 0.21153846 | 0.21153846 | 0.03030303 | POST |
| rocco1.8233 | rocco | 3 | 1.823 | roll | only heard | 0 | 7 | m | f | 0.045454545 | 0.1818182 | 0.036781609 | POST |
| rocco1.8233 | rocco | 3 | 1.823 | tinino1 | only heard | 0 | 7 | m | m | 0 | 0 | 0.021505376 | POST |
| rocco1.8233 | rocco | 3 | 1.823 | tris | only heard | 0 | 7 | m | f | 0.10144928 | 0.23188405 | 0.010449321 | POST |
| rocco1.8233 | rocco | 3 | 1.823 | scapola | only heard | 0 | 7 | m | f | 0 | 0 | 0.003787879 | POST |
| rocco1.8233 | rocco | 3 | 1.823 | ruga | only heard | 0 | 7 | m | f | 0.12698412 | 0.2222222 | 0.004608295 | POST |
| rocco1.8233 | rocco | 3 | 1.823 | sandro | only heard | 0 | 7 | m | m | 0 | 0.09375 | 0.089430894 | POST |
| rocco1.853 | rocco | 3 | 1.85 | sandro | seen and heard | 0 | 8 | m | m | 0 | 0.09375 | 0.089430894 | POST |
| rocco1.853 | rocco | 3 | 1.85 | tinino1 | seen and heard | 0 | 8 | m | m | 0 | 0 | 0.021505376 | POST |
| rocco1.853 | rocco | 3 | 1.85 | sally | seen and heard | 0 | 8 | m | f | 0.6 | 0.4 | 0.002949853 | POST |
| rocco1.853 | rocco | 3 | 1.85 | osso | only heard | 0 | 8 | m | f | 0.21153846 | 0.21153846 | 0.03030303 | POST |
| rocco1.853 | rocco | 3 | 1.85 | tris | only heard | 0 | 8 | m | f | 0.10144928 | 0.23188405 | 0.010449321 | POST |
| rocco1.853 | rocco | 3 | 1.85 | roll | only heard | 0 | 8 | m | f | 0.152777778 | 0.191489362 | 0.036781609 | POST |
| rocco1.853 | rocco | 3 | 1.85 | scapola | only heard | 0 | 8 | m | f | 0 | 0 | 0.003787879 | POST |
| rocco1.853 | rocco | 3 | 1.85 | ruga | only heard | 0 | 8 | m | f | 0.12698412 | 0.2222222 | 0.004608295 | POST |
| rocco1.861 | rocco | 1 | 1.86 | osso | seen and heard | 0 | 3 | m | f | 0.21153846 | 0.21153846 | 0.03030303 | POST |
| rocco1.861 | rocco | 1 | 1.86 | sandro | only heard | 1 | 3 | m | m | 0 | 0.09375 | 0.089430894 | POST |
| rocco1.861 | rocco | 1 | 1.86 | tino | only heard | 0 | 3 | m | m | 0 | 0 | 0.111111111 | POST |
| rocco1.9253 | rocco | 3 | 1.925 | osso | seen and heard | 0 | 7 | m | f | 0.21153846 | 0.21153846 | 0.03030303 | POST |
| rocco1.9253 | rocco | 3 | 1.925 | roll | only heard | 0 | 7 | m | f | 0.045454545 | 0.1818182 | 0.036781609 | POST |
| rocco1.9253 | rocco | 3 | 1.925 | tinino1 | only heard | 0 | 7 | m | m | 0 | 0 | 0.021505376 | POST |
| rocco1.9253 | rocco | 3 | 1.925 | tris | only heard | 0 | 7 | m | f | 0.10144928 | 0.23188405 | 0.010449321 | POST |
| rocco1.9253 | rocco | 3 | 1.925 | scapola | only heard | 0 | 7 | m | f | 0 | 0 | 0.003787879 | POST |
| rocco1.9253 | rocco | 3 | 1.925 | ruga | only heard | 0 | 7 | m | f | 0.12698412 | 0.2222222 | 0.004608295 | POST |
| rocco1.9253 | rocco | 3 | 1.925 | sandro | only heard | 0 | 7 | m | m | 0 | 0.09375 | 0.089430894 | POST |
| rocco1.953 | rocco | 3 | 1.95 | sally | seen and heard | 0 | 6 | m | f | 0.6 | 0.4 | 0.002949853 | POST |
| rocco1.953 | rocco | 3 | 1.95 | scapola | only heard | 0 | 6 | m | f | 0 | 0 | 0.003787879 | POST |
| rocco1.953 | rocco | 3 | 1.95 | roll | only heard | 0 | 6 | m | f | 0.152777778 | 0.191489362 | 0.036781609 | POST |
| rocco1.953 | rocco | 3 | 1.95 | ruga | only heard | 0 | 6 | m | f | 0.12698412 | 0.2222222 | 0.004608295 | PRE |
| rocco1.953 | rocco | 3 | 1.95 | tris | only heard | 0 | 6 | m | f | 0.10144928 | 0.23188405 | 0.010449321 | PRE |
| rocco1.953 | rocco | 3 | 1.95 | osso | only heard | 0 | 6 | m | f | 0.21153846 | 0.21153846 | 0.03030303 | PRE |
| rocco2.0983 | rocco | 3 | 2.098 | tris | only heard | 0 | 4 | m | f | 0.10144928 | 0.23188405 | 0.010449321 | PRE |
| rocco2.0983 | rocco | 3 | 2.098 | roll | only heard | 0 | 4 | m | f | 0.152777778 | 0.191489362 | 0.036781609 | PRE |
| rocco2.0983 | rocco | 3 | 2.098 | ruga | only heard | 0 | 4 | m | f | 0.12698412 | 0.2222222 | 0.004608295 | PRE |
| rocco2.0983 | rocco | 3 | 2.098 | scapola | only heard | 0 | 4 | m | f | 0 | 0 | 0.003787879 | PRE |
| rocco2.1921 | rocco | 1 | 2.192 | tris | seen and heard | 0 | 5 | m | f | 0.10144928 | 0.23188405 | 0.010449321 | PRE |
| rocco2.1921 | rocco | 1 | 2.192 | osso | seen and heard | 0 | 5 | m | f | 0.21153846 | 0.21153846 | 0.03030303 | PRE |
| rocco2.1921 | rocco | 1 | 2.192 | ruga | seen and heard | 0 | 5 | m | f | 0.12698412 | 0.2222222 | 0.004608295 | PRE |
| rocco2.1921 | rocco | 1 | 2.192 | roll | seen and heard | 0 | 5 | m | f | 0.152777778 | 0.191489362 | 0.036781609 | PRE |
| rocco2.1921 | rocco | 1 | 2.192 | scapola | seen and heard | 0 | 5 | m | f | 0 | 0 | 0.003787879 | PRE |
| rocco2.363 | rocco | 3 | 2.36 | tris | only heard | 0 | 6 | m | f | 0.10144928 | 0.23188405 | 0.010449321 | PRE |
| rocco2.363 | rocco | 3 | 2.36 | tinino1 | only heard | 0 | 6 | m | m | 0 | 0 | 0.021505376 | PRE |
| rocco2.363 | rocco | 3 | 2.36 | tinino2 | only heard | 0 | 6 | m | m | 0 | 0 | 0.037037037 | PRE |
| rocco2.363 | rocco | 3 | 2.36 | sandro | only heard | 1 | 6 | m | m | 0 | 0.09375 | 0.089430894 | PRE |
| rocco2.363 | rocco | 3 | 2.36 | scapola | only heard | 0 | 6 | m | f | 0 | 0 | 0.003787879 | PRE |
| rocco2.363 | rocco | 3 | 2.36 | osso | only heard | 0 | 6 | m | f | 0.21153846 | 0.21153846 | 0.03030303 | PRE |
| rocco2.453 | rocco | 3 | 2.45 | sally | seen and heard | 0 | 6 | m | f | 0.6 | 0.4 | 0.002949853 | PRE |
| rocco2.453 | rocco | 3 | 2.45 | osso | only heard | 0 | 6 | m | f | 0.21153846 | 0.21153846 | 0.03030303 | PRE |
| rocco2.453 | rocco | 3 | 2.45 | tris | only heard | 0 | 6 | m | f | 0.10144928 | 0.23188405 | 0.010449321 | PRE |
| rocco2.453 | rocco | 3 | 2.45 | scapola | only heard | 0 | 6 | m | f | 0 | 0 | 0.003787879 | PRE |
| rocco2.453 | rocco | 3 | 2.45 | ruga | only heard | 0 | 6 | m | f | 0.12698412 | 0.2222222 | 0.004608295 | PRE |
| rocco2.453 | rocco | 3 | 2.45 | roll | only heard | 0 | 6 | m | f | 0.152777778 | 0.191489362 | 0.036781609 | PRE |
| rocco2.5043 | rocco | 3 | 2.504 | sandro | seen and heard | 0 | 6 | m | m | 0 | 0.09375 | 0.089430894 | PRE |
| rocco2.5043 | rocco | 3 | 2.504 | tinino1 | seen and heard | 1 | 6 | m | m | 0 | 0 | 0.021505376 | PRE |
| rocco2.5043 | rocco | 3 | 2.504 | roll | seen and heard | 0 | 6 | m | f | 0.152777778 | 0.191489362 | 0.036781609 | PRE |
| rocco2.5043 | rocco | 3 | 2.504 | tris | seen and heard | 0 | 6 | m | f | 0.10144928 | 0.23188405 | 0.010449321 | PRE |
| rocco2.5043 | rocco | 3 | 2.504 | ruga | seen and heard | 0 | 6 | m | f | 0.12698412 | 0.2222222 | 0.004608295 | PRE |
| rocco2.5043 | rocco | 3 | 2.504 | tinino2 | only heard | 0 | 6 | m | m | 0 | 0 | 0.037037037 | PRE |
| rocco2.783 | rocco | 3 | 2.78 | sandro | seen and heard | 0 | 5 | m | m | 0 | 0.09375 | 0.089430894 | PRE |
| rocco2.783 | rocco | 3 | 2.78 | tinino1 | seen and heard | 0 | 5 | m | m | 0 | 0 | 0.021505376 | PRE |
| rocco2.783 | rocco | 3 | 2.78 | tris | seen and heard | 0 | 5 | m | f | 0.10144928 | 0.23188405 | 0.010449321 | PRE |
| rocco2.783 | rocco | 3 | 2.78 | ruga | only heard | 0 | 5 | m | f | 0.12698412 | 0.2222222 | 0.004608295 | PRE |
| rocco2.783 | rocco | 3 | 2.78 | roll | only heard | 0 | 5 | m | f | 0.152777778 | 0.191489362 | 0.036781609 | PRE |
| rocco2.83 | rocco | 3 | 2.8 | sally | only seen | 0 | 5 | m | f | 0.6 | 0.4 | 0.002949853 | PRE |
| rocco2.83 | rocco | 3 | 2.8 | roll | only seen | 0 | 5 | m | f | 0.152777778 | 0.191489362 | 0.036781609 | PRE |
| rocco2.83 | rocco | 3 | 2.8 | ruga | only seen | 0 | 5 | m | f | 0.12698412 | 0.2222222 | 0.004608295 | PRE |
| rocco2.83 | rocco | 3 | 2.8 | tris | 0 | 0 | 5 | m | f | 0.10144928 | 0.23188405 | 0.010449321 | PRE |
| rocco2.83 | rocco | 3 | 2.8 | scapola | 0 | 0 | 5 | m | f | 0 | 0 | 0.003787879 | PRE |
| rocco3.7583 | rocco | 3 | 3.758 | osso | seen and heard | 0 | 6 | m | f | 0.21153846 | 0.21153846 | 0.03030303 | PRE |
| rocco3.7583 | rocco | 3 | 3.758 | roll | only heard | 0 | 6 | m | f | 0.045454545 | 0.1818182 | 0.036781609 | PRE |
| rocco3.7583 | rocco | 3 | 3.758 | tris | only heard | 0 | 6 | m | f | 0.10144928 | 0.23188405 | 0.010449321 | PRE |
| rocco3.7583 | rocco | 3 | 3.758 | scapola | only heard | 0 | 6 | m | f | 0 | 0 | 0.003787879 | PRE |
| rocco3.7583 | rocco | 3 | 3.758 | ruga | only heard | 0 | 6 | m | f | 0.12698412 | 0.2222222 | 0.004608295 | PRE |
| rocco5.0023 | rocco | 3 | 5.002 | osso | seen and heard | 0 | 5 | m | f | 0.21153846 | 0.21153846 | 0.03030303 | PRE |
| rocco5.0023 | rocco | 3 | 5.002 | tris | seen and heard | 0 | 5 | m | f | 0.10144928 | 0.23188405 | 0.010449321 | PRE |
| rocco5.0023 | rocco | 3 | 5.002 | roll | seen and heard | 0 | 5 | m | f | 0.152777778 | 0.191489362 | 0.036781609 | PRE |
| rocco5.0023 | rocco | 3 | 5.002 | ruga | 0 | 0 | 5 | m | f | 0.12698412 | 0.2222222 | 0.004608295 | PRE |
| rocco5.0023 | rocco | 3 | 5.002 | scapola | 0 | 0 | 5 | m | f | 0 | 0 | 0.003787879 | PRE |
| rocco5.493 | rocco | 3 | 5.49 | ruga | only heard | 0 | 2 | m | f | 0.12698412 | 0.2222222 | 0.004608295 | PRE |
| rocco5.493 | rocco | 3 | 5.49 | tris | only heard | 0 | 2 | m | f | 0.10144928 | 0.23188405 | 0.010449321 | PRE |
| roll1.0171 | roll | 1 | 1.017 | scapola | only heard | 0 | 4 | f | f | 0.05333333 | 0.08 | 0.003787879 | POST |
| roll1.0171 | roll | 1 | 1.017 | tris | only heard | 0 | 4 | f | f | 0.02097902 | 0.08391608 | 0.010449321 | POST |
| roll1.0171 | roll | 1 | 1.017 | ruga | only heard | 0 | 4 | f | f | 0.04166667 | 0.30208333 | 0.004608295 | POST |
| roll1.0171 | roll | 1 | 1.017 | osso | only heard | 0 | 4 | f | f | 0.0117647 | 0.15294117 | 0.03030303 | POST |
| roll1.181 | roll | 1 | 1.18 | stella | 0 | 0 | 4 | f | f | 0 | 0.07894736 | 0.020080321 | POST |
| roll1.181 | roll | 1 | 1.18 | sonia | 0 | 0 | 4 | f | f | 0.1 | 0.1 | 0.041666667 | POST |
| roll1.181 | roll | 1 | 1.18 | ruga | 0 | 0 | 4 | f | f | 0.04166667 | 0.30208333 | 0.004608295 | POST |
| roll1.181 | roll | 1 | 1.18 | neomamma | 0 | 1 | 4 | f | f | 0.111111111 | 0.155555556 | 0.032840722 | POST |
| roll1.223 | roll | 3 | 1.22 | ruga | 0 | 0 | 2 | f | f | 0.04166667 | 0.30208333 | 0.004608295 | POST |
| roll1.223 | roll | 3 | 1.22 | sciura | 0 | 0 | 2 | f | f | 0.25139664 | 0.24581005 | 0.06377858 | POST |
| roll1.221 | roll | 1 | 1.22 | spilla | 0 | 0 | 1 | f | f | 0 | 0.11475409 | 0.043678161 | POST |
| roll1.271 | roll | 1 | 1.27 | sonia | only heard | 1 | 1 | f | f | 0.1 | 0.1 | 0.041666667 | POST |
| roll1.331 | roll | 1 | 1.33 | tris | 0 | 0 | 2 | f | f | 0.02097902 | 0.08391608 | 0.010449321 | POST |
| roll1.331 | roll | 1 | 1.33 | sciura | 0 | 0 | 2 | f | f | 0.25139664 | 0.24581005 | 0.06377858 | POST |
| roll1.381 | roll | 1 | 1.38 | spilla | only seen | 0 | 4 | f | f | 0 | 0.11475409 | 0.043678161 | POST |
| roll1.381 | roll | 1 | 1.38 | tris | 0 | 0 | 4 | f | f | 0.02097902 | 0.08391608 | 0.010449321 | POST |
| roll1.381 | roll | 1 | 1.38 | ruga | 0 | 0 | 4 | f | f | 0.04166667 | 0.30208333 | 0.004608295 | POST |
| roll1.381 | roll | 1 | 1.38 | severa | 0 | 1 | 4 | f | f | 0.02083333 | 0 | 0.066176471 | POST |
| roll1.51 | roll | 1 | 1.5 | severa | 0 | 0 | 3 | f | f | 0.02083333 | 0 | 0.066176471 | POST |
| roll1.51 | roll | 1 | 1.5 | spilla | 0 | 0 | 3 | f | f | 0 | 0.11475409 | 0.043678161 | POST |
| roll1.51 | roll | 1 | 1.5 | tris | 0 | 0 | 3 | f | f | 0.02097902 | 0.08391608 | 0.010449321 | POST |
| roll1.521 | roll | 1 | 1.52 | tris | only seen | 0 | 3 | f | f | 0.02097902 | 0.08391608 | 0.010449321 | POST |
| roll1.521 | roll | 1 | 1.52 | sciura | 0 | 0 | 3 | f | f | 0.25139664 | 0.24581005 | 0.06377858 | POST |
| roll1.521 | roll | 1 | 1.52 | ruga | 0 | 0 | 3 | f | f | 0.04166667 | 0.30208333 | 0.004608295 | POST |
| roll1.651 | roll | 1 | 1.65 | neomamma | only seen | 0 | 4 | f | f | 0.111111111 | 0.155555556 | 0.032840722 | POST |
| roll1.651 | roll | 1 | 1.65 | sciura | 0 | 0 | 4 | f | f | 0.25139664 | 0.24581005 | 0.06377858 | POST |
| roll1.651 | roll | 1 | 1.65 | schiarita | 0 | 0 | 4 | f | f | 0.0625 | 0.125 | 0.041420118 | POST |
| roll1.651 | roll | 1 | 1.65 | sally | 0 | 0 | 4 | f | f | 0 | 0.16666667 | 0.002949853 | POST |
| roll1.831 | roll | 1 | 1.83 | severa | only heard | 0 | 5 | f | f | 0.02083333 | 0 | 0.066176471 | POST |
| roll1.831 | roll | 1 | 1.83 | tris | only heard | 1 | 5 | f | f | 0.02097902 | 0.08391608 | 0.010449321 | POST |
| roll1.831 | roll | 1 | 1.83 | osso | only heard | 0 | 5 | f | f | 0.0117647 | 0.15294117 | 0.03030303 | POST |
| roll1.831 | roll | 1 | 1.83 | spilla | only heard | 1 | 5 | f | f | 0 | 0.11475409 | 0.043678161 | POST |
| roll1.831 | roll | 1 | 1.83 | sonia | only heard | 0 | 5 | f | f | 0.1 | 0.1 | 0.041666667 | POST |
| roll1.861 | roll | 1 | 1.86 | spilla | 0 | 0 | 2 | f | f | 0 | 0.11475409 | 0.043678161 | POST |
| roll1.861 | roll | 1 | 1.86 | sciura | 0 | 0 | 2 | f | f | 0.25139664 | 0.24581005 | 0.06377858 | POST |
| roll1.961 | roll | 1 | 1.96 | severa | only heard | 0 | 3 | f | f | 0.02083333 | 0 | 0.066176471 | POST |
| roll1.961 | roll | 1 | 1.96 | spilla | only heard | 0 | 3 | f | f | 0 | 0.11475409 | 0.043678161 | POST |
| roll1.961 | roll | 1 | 1.96 | tris | only heard | 0 | 3 | f | f | 0.02097902 | 0.08391608 | 0.010449321 | PRE |
| roll2.021 | roll | 1 | 2.02 | sciura | only heard | 0 | 2 | f | f | 0.25139664 | 0.24581005 | 0.06377858 | PRE |
| roll2.021 | roll | 1 | 2.02 | ruga | only heard | 0 | 2 | f | f | 0.04166667 | 0.30208333 | 0.004608295 | PRE |
| roll2.241 | roll | 1 | 2.24 | sciura | only seen | 0 | 2 | f | f | 0.25139664 | 0.24581005 | 0.06377858 | PRE |
| roll2.241 | roll | 1 | 2.24 | ruga | 0 | 0 | 2 | f | f | 0.04166667 | 0.30208333 | 0.004608295 | PRE |
| roll2.55* | roll | * | 2.55 | ruga | 0 | 0 | 1 | f | f | 0.04166667 | 0.30208333 | 0.004608295 | PRE |
| roll2.931 | roll | 1 | 2.93 | schiarita | 0 | 0 | 2 | f | f | 0.0625 | 0.125 | 0.041420118 | PRE |
| roll2.931 | roll | 1 | 2.93 | stacy | 0 | 0 | 2 | f | f | 0 | 0.22222222 | 0.021317829 | PRE |
| roll3.243 | roll | 3 | 3.24 | spilla | 0 | 0 | 2 | f | f | 0 | 0.11475409 | 0.043678161 | PRE |
| roll3.243 | roll | 3 | 3.24 | sciura | 0 | 0 | 2 | f | f | 0.25139664 | 0.24581005 | 0.06377858 | PRE |
| roll3.441 | roll | 1 | 3.44 | sciura | only seen | 0 | 2 | f | f | 0.25139664 | 0.24581005 | 0.06377858 | PRE |
| roll3.441 | roll | 1 | 3.44 | spilla | 0 | 0 | 2 | f | f | 0 | 0.11475409 | 0.043678161 | PRE |
| roll3.81 | roll | 1 | 3.8 | spilla | 0 | 0 | 2 | f | f | 0 | 0.11475409 | 0.043678161 | PRE |
| roll3.81 | roll | 1 | 3.8 | sciura | 0 | 0 | 2 | f | f | 0.25139664 | 0.24581005 | 0.06377858 | PRE |
| roll3.831 | roll | 1 | 3.83 | sciura | only seen | 1 | 2 | f | f | 0.25139664 | 0.24581005 | 0.06377858 | PRE |
| roll3.831 | roll | 1 | 3.83 | ruga | 0 | 0 | 2 | f | f | 0.04166667 | 0.30208333 | 0.004608295 | PRE |
| roll4.321 | roll | 1 | 4.32 | stacy | 0 | 0 | 1 | f | f | 0 | 0.22222222 | 0.021317829 | PRE |
| roll5.411 | roll | 1 | 5.41 | severa | 0 | 0 | 1 | f | f | 0.02083333 | 0 | 0.066176471 | PRE |
| roll6.343 | roll | 3 | 6.34 | sciura | seen and heard | 0 | 2 | f | f | 0.25139664 | 0.24581005 | 0.06377858 | PRE |
| roll6.343 | roll | 3 | 6.34 | ruga | only heard | 0 | 2 | f | f | 0.04166667 | 0.30208333 | 0.004608295 | PRE |
| roll9.192 | roll | 2 | 9.19 | tris | only seen | 0 | 3 | f | f | 0.02097902 | 0.08391608 | 0.010449321 | PRE |
| roll9.192 | roll | 2 | 9.19 | ruga | only seen | 0 | 3 | f | f | 0.04166667 | 0.30208333 | 0.004608295 | PRE |
| roll9.192 | roll | 2 | 9.19 | sciura | 0 | 0 | 3 | f | f | 0.25139664 | 0.24581005 | 0.06377858 | PRE |
| rosa1.331 | rosa | 1 | 1.33 | sine | only seen | 0 | 6 | f | f | 0 | 0.108108108 | 0.050682261 | POST |
| rosa1.331 | rosa | 1 | 1.33 | schiarita | only seen | 0 | 6 | f | f | 0.06122448 | 0.08163265 | 0.041420118 | POST |
| rosa1.331 | rosa | 1 | 1.33 | neomamma | 0 | 0 | 6 | f | f | 0 | 0.18032786 | 0.032840722 | POST |
| rosa1.331 | rosa | 1 | 1.33 | scapola | 0 | 0 | 6 | f | f | 0 | 0.02564102 | 0.003787879 | POST |
| rosa1.331 | rosa | 1 | 1.33 | spiga | 0 | 0 | 6 | f | f | 0 | 0.08510638 | 0.018372703 | POST |
| rosa1.331 | rosa | 1 | 1.33 | spot | 0 | 0 | 6 | f | f | 0 | 0.04651162 | 0.020576132 | POST |
| rosa1.93 | rosa | 3 | 1.9 | stacy | only seen | 1 | 7 | f | f | 0.06382978 | 0 | 0.021317829 | POST |
| rosa1.93 | rosa | 3 | 1.9 | sine | only seen | 0 | 7 | f | f | 0 | 0.108108108 | 0.050682261 | POST |
| rosa1.93 | rosa | 3 | 1.9 | spot | 0 | 0 | 7 | f | f | 0 | 0.04651162 | 0.020576132 | POST |
| rosa1.93 | rosa | 3 | 1.9 | secco | 0 | 0 | 7 | f | m | 0.10344827 | 0 | 0.123076923 | POST |
| rosa1.93 | rosa | 3 | 1.9 | sfasciato | 0 | 0 | 7 | f | m | 0 | 0.10344827 | 0.024390244 | POST |
| rosa1.93 | rosa | 3 | 1.9 | schiarita | 0 | 0 | 7 | f | f | 0.06122448 | 0.08163265 | 0.041420118 | POST |
| rosa1.93 | rosa | 3 | 1.9 | strega | 0 | 0 | 7 | f | f | 0.1707317 | 0.04878048 | 0.016025641 | POST |
| rosa1.93* | rosa | * | 1.93 | sfasciato | 0 | 0 | 4 | f | m | 0 | 0.10344827 | 0.024390244 | POST |
| rosa1.93* | rosa | * | 1.93 | spilla | 0 | 0 | 4 | f | f | 0 | 0.16666667 | 0.043678161 | POST |
| rosa1.93* | rosa | * | 1.93 | osso | 0 | 0 | 4 | f | f | 0 | 0.5 | 0.03030303 | POST |
| rosa1.93* | rosa | * | 1.93 | severa | 0 | 0 | 4 | f | f | 0 | 0.16666667 | 0.066176471 | PRE |
| rosa2.443 | rosa | 3 | 2.44 | stacy | only seen | 1 | 9 | f | f | 0.06382978 | 0 | 0.021317829 | PRE |
| rosa2.443 | rosa | 3 | 2.44 | strega | only seen | 1 | 9 | f | f | 0.1707317 | 0.04878048 | 0.016025641 | PRE |
| rosa2.443 | rosa | 3 | 2.44 | schiarita | 0 | 0 | 9 | f | f | 0.6122448 | 0.08163265 | 0.041420118 | PRE |
| rosa2.443 | rosa | 3 | 2.44 | neomamma | 0 | 0 | 9 | f | f | 0 | 0.18032786 | 0.032840722 | PRE |
| rosa2.443 | rosa | 3 | 2.44 | spiga | 0 | 0 | 9 | f | f | 0 | 0.08510638 | 0.018372703 | PRE |
| rosa2.443 | rosa | 3 | 2.44 | sfasciato | 0 | 0 | 9 | f | m | 0 | 0.10344827 | 0.024390244 | PRE |
| rosa2.443 | rosa | 3 | 2.44 | scapola | 0 | 0 | 9 | f | f | 0 | 0.02564102 | 0.003787879 | PRE |
| rosa2.443 | rosa | 3 | 2.44 | spot | 0 | 0 | 9 | f | f | 0 | 0.04651162 | 0.020576132 | PRE |
| rosa2.443 | rosa | 3 | 2.44 | sally | 0 | 0 | 9 | f | f | 0 | 0.07692307 | 0.002949853 | PRE |
| rosa2.533 | rosa | 3 | 2.53 | sfasciato | 0 | 0 | 2 | f | m | 0 | 0.10344827 | 0.024390244 | PRE |
| rosa2.533 | rosa | 3 | 2.53 | stacy | 0 | 0 | 2 | f | f | 0.06382978 | 0 | 0.021317829 | PRE |
| rosa3.181 | rosa | 1 | 3.18 | stacy | only seen | 0 | 2 | f | f | 0.06382978 | 0 | 0.021317829 | PRE |
| rosa3.181 | rosa | 1 | 3.18 | sfasciato | 0 | 0 | 2 | f | m | 0 | 0.10344827 | 0.024390244 | PRE |
| ruga2.251 | ruga | 1 | 2.25 | sonia | only seen | 0 | 3 | f | f | 0.16129032 | 0.12903225 | 0.041666667 | POST |
| ruga2.251 | ruga | 1 | 2.25 | sciura | 0 | 0 | 3 | f | f | 0.3649635 | 0.19708029 | 0.06377858 | PRE |
| ruga2.251 | ruga | 1 | 2.25 | tris | 0 | 0 | 3 | f | f | 0.02803738 | 0.13084112 | 0.010449321 | PRE |
| sally1.8711 | sally | 1 | 1.871 | strega | only seen | 0 | 3 | f | f | 0.07142857 | 0 | 0.016025641 | PRE |
| sally1.8711 | sally | 1 | 1.871 | susy | 0 | 0 | 3 | f | f | 0.26 | 0.02 | 0.027777778 | PRE |
| sally1.8711 | sally | 1 | 1.871 | schiarita | 0 | 0 | 3 | f | f | 0.17567567 | 0.24324324 | 0.041420118 | PRE |
| sandro1.063 | sandro | 3 | 1.06 | tino | only seen | 0 | 3 | m | m | 0.5 | 0 | 0.111111111 | POST |
| sandro1.063 | sandro | 3 | 1.06 | osso | 0 | 0 | 3 | m | f | 0 | 0 | 0.03030303 | POST |
| sandro1.063 | sandro | 3 | 1.06 | rocco | 0 | 0 | 3 | m | m | 0 | 0.09375 | 0.25 | POST |
| sandro1.953 | sandro | 3 | 1.95 | tinino2 | seen and heard | 0 | 6 | m | m | 0.44 | 0.12 | 0.037037037 | POST |
| sandro1.953 | sandro | 3 | 1.95 | osso | only heard | 0 | 6 | m | f | 0 | 0 | 0.03030303 | POST |
| sandro1.953 | sandro | 3 | 1.95 | tris | only heard | 0 | 6 | m | f | 0 | 0.09375 | 0.010449321 | POST |
| sandro1.953 | sandro | 3 | 1.95 | ruga | only heard | 0 | 6 | m | f | 0 | 0.10344827 | 0.004608295 | POST |
| sandro1.953 | sandro | 3 | 1.95 | roll | only heard | 0 | 6 | m | f | 0 | 0 | 0.036781609 | POST |
| sandro1.953 | sandro | 3 | 1.95 | scapola | only heard | 0 | 6 | m | f | 0 | 0 | 0.003787879 | POST |
| sandro2.111 | sandro | 1 | 2.11 | tinino1 | seen and heard | 0 | 6 | m | m | 0.34482759 | 0.03448275 | 0.021505376 | POST |
| sandro2.111 | sandro | 1 | 2.11 | tinino2 | seen and heard | 0 | 6 | m | m | 0.44 | 0.12 | 0.037037037 | POST |
| sandro2.111 | sandro | 1 | 2.11 | scapola | only heard | 0 | 6 | m | f | 0 | 0 | 0.003787879 | POST |
| sandro2.111 | sandro | 1 | 2.11 | roll | only heard | 0 | 6 | m | f | 0 | 0 | 0.036781609 | POST |
| sandro2.111 | sandro | 1 | 2.11 | ruga | only heard | 0 | 6 | m | f | 0 | 0.10344827 | 0.004608295 | POST |
| sandro2.111 | sandro | 1 | 2.11 | tris | only heard | 0 | 6 | m | f | 0 | 0.09375 | 0.010449321 | POST |
| sandro2.651 | sandro | 1 | 2.65 | roll | only heard | 0 | 5 | m | f | 0 | 0 | 0.036781609 | POST |
| sandro2.651 | sandro | 1 | 2.65 | tris | only heard | 0 | 5 | m | f | 0 | 0.09375 | 0.010449321 | POST |
| sandro2.651 | sandro | 1 | 2.65 | scapola | only heard | 0 | 5 | m | f | 0 | 0 | 0.003787879 | POST |
| sandro2.651 | sandro | 1 | 2.65 | ruga | only heard | 0 | 5 | m | f | 0 | 0.10344827 | 0.004608295 | POST |
| sandro2.651 | sandro | 1 | 2.65 | osso | only heard | 0 | 5 | m | f | 0 | 0 | 0.03030303 | POST |
| sandro2.983 | sandro | 3 | 2.98 | tinino1 | seen and heard | 0 | 6 | m | m | 0.34482759 | 0.03448275 | 0.021505376 | POST |
| sandro2.983 | sandro | 3 | 2.98 | rocco | only heard | 0 | 6 | m | m | 0 | 0.09375 | 0.25 | POST |
| sandro2.983 | sandro | 3 | 2.98 | scapola | only heard | 0 | 6 | m | f | 0 | 0 | 0.003787879 | POST |
| sandro2.983 | sandro | 3 | 2.98 | tris | only heard | 0 | 6 | m | f | 0 | 0.09375 | 0.010449321 | POST |
| sandro2.983 | sandro | 3 | 2.98 | roll | only heard | 0 | 6 | m | f | 0 | 0 | 0.036781609 | PRE |
| sandro2.983 | sandro | 3 | 2.98 | tinino2 | only heard | 0 | 6 | m | m | 0.44 | 0.12 | 0.037037037 | PRE |
| sandro3.132 | sandro | 2 | 3.13 | tinino1 | seen and heard | 1 | 8 | m | m | 0.34482759 | 0.03448275 | 0.021505376 | PRE |
| sandro3.132 | sandro | 2 | 3.13 | tinino2 | seen and heard | 0 | 8 | m | m | 0.44 | 0.12 | 0.037037037 | PRE |
| sandro3.132 | sandro | 2 | 3.13 | ruga | only heard | 0 | 8 | m | f | 0 | 0.10344827 | 0.004608295 | PRE |
| sandro3.132 | sandro | 2 | 3.13 | roll | only heard | 0 | 8 | m | f | 0 | 0 | 0.036781609 | PRE |
| sandro3.132 | sandro | 2 | 3.13 | tris | only heard | 0 | 8 | m | f | 0 | 0.09375 | 0.010449321 | PRE |
| sandro3.132 | sandro | 2 | 3.13 | scapola | only heard | 0 | 8 | m | f | 0 | 0 | 0.003787879 | PRE |
| sandro3.132 | sandro | 2 | 3.13 | osso | only heard | 0 | 8 | m | f | 0 | 0 | 0.03030303 | PRE |
| sandro3.132 | sandro | 2 | 3.13 | rocco | only heard | 0 | 8 | m | m | 0 | 0.09375 | 0.25 | PRE |
| sandro5.233 | sandro | 3 | 5.23 | tinino2 | seen and heard | 0 | 7 | m | m | 0.44 | 0.12 | 0.037037037 | PRE |
| sandro5.233 | sandro | 3 | 5.23 | osso | only heard | 0 | 7 | m | f | 0 | 0 | 0.03030303 | PRE |
| sandro5.233 | sandro | 3 | 5.23 | roll | only heard | 0 | 7 | m | f | 0 | 0 | 0.036781609 | PRE |
| sandro5.233 | sandro | 3 | 5.23 | tris | only heard | 0 | 7 | m | f | 0 | 0.09375 | 0.010449321 | PRE |
| sandro5.233 | sandro | 3 | 5.23 | ruga | only heard | 1 | 7 | m | f | 0 | 0.10344827 | 0.004608295 | PRE |
| sandro5.233 | sandro | 3 | 5.23 | scapola | only heard | 0 | 7 | m | f | 0 | 0 | 0.003787879 | PRE |
| sandro5.233 | sandro | 3 | 5.23 | rocco | only heard | 0 | 7 | m | m | 0 | 0.09375 | 0.25 | PRE |
| scapola1.341 | scapola | 1 | 1.34 | spot | only seen | 0 | 6 | f | f | 0 | 0.15584415 | 0.020576132 | POST |
| scapola1.341 | scapola | 1 | 1.34 | susy | only seen | 0 | 6 | f | f | 0.26388889 | 0.22222222 | 0.027777778 | POST |
| scapola1.341 | scapola | 1 | 1.34 | schiarita | only seen | 0 | 6 | f | f | 0 | 0.08620689 | 0.041420118 | POST |
| scapola1.341 | scapola | 1 | 1.34 | neomamma | 0 | 0 | 6 | f | f | 0 | 0.12820512 | 0.032840722 | POST |
| scapola1.341 | scapola | 1 | 1.34 | rosa | 0 | 0 | 6 | f | f | 0 | 0.02564102 | 0.027131783 | POST |
| scapola1.341 | scapola | 1 | 1.34 | spiga | 0 | 0 | 6 | f | f | 0 | 0.14285714 | 0.018372703 | POST |
| scapola1.531 | scapola | 1 | 1.53 | sine | 0 | 0 | 6 | f | f | 0 | 0.20833333 | 0.050682261 | POST |
| scapola1.531 | scapola | 1 | 1.53 | rosa | 0 | 0 | 6 | f | f | 0 | 0.02564102 | 0.027131783 | POST |
| scapola1.531 | scapola | 1 | 1.53 | stacy | 0 | 0 | 6 | f | f | 0.01428571 | 0.15714285 | 0.021317829 | POST |
| scapola1.531 | scapola | 1 | 1.53 | susy | 0 | 0 | 6 | f | f | 0.26388889 | 0.22222222 | 0.027777778 | POST |
| scapola1.531 | scapola | 1 | 1.53 | neomamma | 0 | 0 | 6 | f | f | 0 | 0.12820512 | 0.032840722 | PRE |
| scapola1.531 | scapola | 1 | 1.53 | spot | 0 | 0 | 6 | f | f | 0 | 0.15584415 | 0.020576132 | PRE |
| scapola1.542 | scapola | 2 | 1.54 | spiga | 0 | 0 | 4 | f | f | 0 | 0.14285714 | 0.018372703 | PRE |
| scapola1.542 | scapola | 2 | 1.54 | sine | 0 | 1 | 4 | f | f | 0 | 0.20833333 | 0.050682261 | PRE |
| scapola1.542 | scapola | 2 | 1.54 | neomamma | 0 | 0 | 4 | f | f | 0 | 0.12820512 | 0.032840722 | PRE |
| scapola1.542 | scapola | 2 | 1.54 | spot | 0 | 0 | 4 | f | f | 0 | 0.15584415 | 0.020576132 | PRE |
| scapola1.61 | scapola | 1 | 1.6 | schiarita | 0 | 0 | 1 | f | f | 0 | 0.08620689 | 0.041420118 | PRE |
| scapola2.871 | scapola | 1 | 2.87 | stacy | only seen | 0 | 7 | f | f | 0.01428571 | 0.15714285 | 0.021317829 | PRE |
| scapola2.871 | scapola | 1 | 2.87 | spot | only seen | 0 | 7 | f | f | 0 | 0.15584415 | 0.020576132 | PRE |
| scapola2.871 | scapola | 1 | 2.87 | sfasciato | 0 | 0 | 7 | f | m | 0 | 0.13333333 | 0.024390244 | PRE |
| scapola2.871 | scapola | 1 | 2.87 | sine | 0 | 0 | 7 | f | f | 0 | 0.20833333 | 0.050682261 | PRE |
| scapola2.871 | scapola | 1 | 2.87 | secco | 0 | 0 | 7 | f | m | 0.08888889 | 0.06666667 | 0.123076923 | PRE |
| scapola2.871 | scapola | 1 | 2.87 | neomamma | 0 | 0 | 7 | f | f | 0 | 0.12820512 | 0.032840722 | PRE |
| scapola2.871 | scapola | 1 | 2.87 | small | 0 | 0 | 7 | f | f | 0.02941176 | 0 | 0.044117647 | PRE |
| schiarita0.121 | schiarita | 1 | 0.12 | ruga | 0 | 0 | 4 | f | f | 0.14285714 | 0.07142857 | 0.004608295 | POST |
| schiarita0.121 | schiarita | 1 | 0.12 | stella | 0 | 0 | 4 | f | f | 0 | 0.14285714 | 0.020080321 | POST |
| schiarita0.121 | schiarita | 1 | 0.12 | stacy | 0 | 0 | 4 | f | f | 0.25490196 | 0.15686274 | 0.021317829 | POST |
| schiarita0.121 | schiarita | 1 | 0.12 | small | 0 | 0 | 4 | f | f | 0 | 0.1372549 | 0.044117647 | POST |
| schiarita1.2923 | schiarita | 3 | 1.292 | sumo | only seen | 0 | 2 | f | m | 0 | 0.23255813 | 0.203125 | POST |
| schiarita1.2923 | schiarita | 3 | 1.292 | stacy | only seen | 0 | 2 | f | f | 0.25490196 | 0.15686274 | 0.021317829 | POST |
| schiarita1.563 | schiarita | 3 | 1.56 | stacy | 0 | 0 | 1 | f | f | 0.25490196 | 0.15686274 | 0.021317829 | POST |
| schiarita1.723 | schiarita | 3 | 1.72 | osso | only seen | 0 | 3 | f | f | 0 | 0 | 0.03030303 | POST |
| schiarita1.723 | schiarita | 3 | 1.72 | schiarita | 0 | 0 | 3 | f | f | 0 | 0 | 0.041420118 | POST |
| schiarita1.723 | schiarita | 3 | 1.72 | spilla | 0 | 0 | 3 | f | f | 0 | 0.17857142 | 0.043678161 | POST |
| schiarita1.851 | schiarita | 1 | 1.85 | stacy | only seen | 0 | 12 | f | f | 0.25490196 | 0.15686274 | 0.021317829 | POST |
| schiarita1.851 | schiarita | 1 | 1.85 | spot | 0 | 0 | 12 | f | f | 0.03658536 | 0.20731707 | 0.020576132 | POST |
| schiarita1.851 | schiarita | 1 | 1.85 | susy | 0 | 0 | 12 | f | f | 0.01639344 | 0.09836065 | 0.027777778 | POST |
| schiarita1.851 | schiarita | 1 | 1.85 | scapola | 0 | 0 | 12 | f | f | 0 | 0.08620689 | 0.003787879 | POST |
| schiarita1.851 | schiarita | 1 | 1.85 | secco | 0 | 0 | 12 | f | m | 0 | 0.1 | 0.123076923 | POST |
| schiarita1.851 | schiarita | 1 | 1.85 | neomamma | 0 | 0 | 12 | f | f | 0 | 0.10091743 | 0.032840722 | POST |
| schiarita1.851 | schiarita | 1 | 1.85 | sally | 0 | 0 | 12 | f | f | 0.17567567 | 0.24324324 | 0.002949853 | POST |
| schiarita1.851 | schiarita | 1 | 1.85 | small | 0 | 0 | 12 | f | f | 0 | 0.1372549 | 0.044117647 | POST |
| schiarita1.851 | schiarita | 1 | 1.85 | sine | 0 | 0 | 12 | f | f | 0.04494387 | 0.23595505 | 0.050682261 | POST |
| schiarita1.851 | schiarita | 1 | 1.85 | sfasciato | 0 | 0 | 12 | f | m | 0 | 0.16666667 | 0.024390244 | POST |
| schiarita1.851 | schiarita | 1 | 1.85 | strega | 0 | 0 | 12 | f | f | 0.04411764 | 0.07352941 | 0.016025641 | POST |
| schiarita1.851 | schiarita | 1 | 1.85 | spiga | 0 | 0 | 12 | f | f | 0.08641975 | 0.13580246 | 0.018372703 | POST |
| schiarita2.081 | schiarita | 1 | 2.08 | scapola | only seen | 1 | 12 | f | f | 0 | 0.08620689 | 0.003787879 | POST |
| schiarita2.081 | schiarita | 1 | 2.08 | stacy | 0 | 0 | 12 | f | f | 0.25490196 | 0.15686274 | 0.021317829 | POST |
| schiarita2.081 | schiarita | 1 | 2.08 | sorcia | 0 | 0 | 12 | f | f | 0 | 0.027027027 | 0.022727273 | POST |
| schiarita2.081 | schiarita | 1 | 2.08 | small | 0 | 0 | 12 | f | f | 0 | 0.1372549 | 0.044117647 | POST |
| schiarita2.081 | schiarita | 1 | 2.08 | ruga | 0 | 0 | 12 | f | f | 0.14285714 | 0.07142857 | 0.004608295 | POST |
| schiarita2.081 | schiarita | 1 | 2.08 | sciura | 0 | 0 | 12 | f | f | 0 | 0.23076923 | 0.06377858 | POST |
| schiarita2.081 | schiarita | 1 | 2.08 | stella | 0 | 0 | 12 | f | f | 0 | 0.14285714 | 0.020080321 | POST |
| schiarita2.081 | schiarita | 1 | 2.08 | spiga | 0 | 0 | 12 | f | f | 0.08641975 | 0.13580246 | 0.018372703 | POST |
| schiarita2.081 | schiarita | 1 | 2.08 | sally | 0 | 0 | 12 | f | f | 0.17567567 | 0.24324324 | 0.002949853 | PRE |
| schiarita2.081 | schiarita | 1 | 2.08 | neomamma | 0 | 0 | 12 | f | f | 0 | 0.10091743 | 0.032840722 | PRE |
| schiarita2.081 | schiarita | 1 | 2.08 | roll | 0 | 0 | 12 | f | f | 0.0625 | 0.125 | 0.036781609 | PRE |
| schiarita2.081 | schiarita | 1 | 2.08 | spot | 0 | 0 | 12 | f | f | 0.03658536 | 0.20731707 | 0.020576132 | PRE |
| schiarita2.141 | schiarita | 1 | 2.14 | spiga | only seen | 0 | 1 | f | f | 0.08641975 | 0.13580246 | 0.018372703 | PRE |
| schiarita2.681 | schiarita | 1 | 2.68 | stacy | only seen | 0 | 2 | f | f | 0.25490196 | 0.15686274 | 0.021317829 | PRE |
| schiarita2.681 | schiarita | 1 | 2.68 | spilla | only seen | 0 | 3 | f | f | 0 | 0.17857142 | 0.043678161 | PRE |
| schiarita2.681 | schiarita | 1 | 2.68 | osso | only seen | 0 | 3 | f | f | 0 | 0 | 0.03030303 | PRE |
| schiarita2.681 | schiarita | 1 | 2.68 | roll | 0 | 1 | 2 | f | f | 0.0625 | 0.125 | 0.036781609 | PRE |
| schiarita2.681 | schiarita | 1 | 2.68 | severa | 0 | 0 | 3 | f | f | 0.04166667 | 0.04166667 | 0.066176471 | PRE |
| schiarita33 | schiarita | 3 | 3 | roll | only seen | 0 | 4 | f | f | 0.0625 | 0.125 | 0.036781609 | PRE |
| schiarita33 | schiarita | 3 | 3 | ruga | only seen | 0 | 4 | f | f | 0.14285714 | 0.07142857 | 0.004608295 | PRE |
| schiarita33 | schiarita | 3 | 3 | sally | 0 | 0 | 4 | f | f | 0.17567567 | 0.24324324 | 0.002949853 | PRE |
| schiarita33 | schiarita | 3 | 3 | sciura | 0 | 0 | 4 | f | f | 0 | 0.23073923 | 0.06377858 | PRE |
| schiarita3.723 | schiarita | 3 | 3.72 | spot | only seen | 0 | 1 | f | f | 0.03658536 | 0.20731707 | 0.020576132 | PRE |
| schiarita4.591 | schiarita | 1 | 4.59 | rosa | only seen | 0 | 9 | f | f | 0.06122448 | 0.08163265 | 0.027131783 | PRE |
| schiarita4.591 | schiarita | 1 | 4.59 | neomamma | only seen | 0 | 9 | f | f | 0 | 0.10091743 | 0.032840722 | PRE |
| schiarita4.591 | schiarita | 1 | 4.59 | sally | 0 | 0 | 9 | f | f | 0.17567567 | 0.24324324 | 0.002949853 | PRE |
| schiarita4.591 | schiarita | 1 | 4.59 | scapola | 0 | 0 | 9 | f | f | 0 | 0.08620689 | 0.003787879 | PRE |
| schiarita4.591 | schiarita | 1 | 4.59 | susy | 0 | 0 | 9 | f | f | 0.01639344 | 0.09836065 | 0.027777778 | PRE |
| schiarita4.591 | schiarita | 1 | 4.59 | spot | 0 | 0 | 9 | f | f | 0.03658536 | 0.20731707 | 0.020576132 | PRE |
| schiarita4.591 | schiarita | 1 | 4.59 | sine | 0 | 0 | 9 | f | f | 0.04494387 | 0.23595505 | 0.050682261 | PRE |
| schiarita4.591 | schiarita | 1 | 4.59 | secco | 0 | 0 | 9 | f | m | 0 | 0.1 | 0.123076923 | PRE |
| schiarita4.591 | schiarita | 1 | 4.59 | spiga | 0 | 0 | 9 | f | f | 0.08641975 | 0.13580246 | 0.018372703 | PRE |
| schiarita11.223 | schiarita | 3 | 11.22 | secco | only seen | 1 | 10 | f | m | 0 | 0.1 | 0.123076923 | PRE |
| schiarita11.223 | schiarita | 3 | 11.22 | sonia | 0 | 0 | 10 | f | f | 0.10714285 | 0.14285714 | 0.041666667 | PRE |
| schiarita11.223 | schiarita | 3 | 11.22 | sguercia | 0 | 0 | 10 | f | f | 0 | 0 | 0 | PRE |
| schiarita11.223 | schiarita | 3 | 11.22 | spiga | 0 | 0 | 10 | f | f | 0.08641975 | 0.13580246 | 0.018372703 | PRE |
| schiarita11.223 | schiarita | 3 | 11.22 | stacy | 0 | 0 | 10 | f | f | 0.25490196 | 0.15686274 | 0.021317829 | PRE |
| schiarita11.223 | schiarita | 3 | 11.22 | scapola | 0 | 0 | 10 | f | f | 0 | 0.08620689 | 0.003787879 | PRE |
| schiarita11.223 | schiarita | 3 | 11.22 | rosa | 0 | 0 | 10 | f | f | 0.06122448 | 0.08163265 | 0.027131783 | PRE |
| schiarita11.223 | schiarita | 3 | 11.22 | neomamma | 0 | 0 | 10 | f | f | 0 | 0.10091743 | 0.032840722 | PRE |
| schiarita11.223 | schiarita | 3 | 11.22 | strega | 0 | 0 | 10 | f | f | 0.04411764 | 0.07352941 | 0.016025641 | PRE |
| schiarita11.223 | schiarita | 3 | 11.22 | small | 0 | 0 | 10 | f | f | 0 | 0.1372549 | 0.044117647 | PRE |
| sciura1.131 | sciura | 1 | 1.13 | sonia | 0 | 0 | 2 | f | f | 0.26153846 | 0.04615384 | 0.041666667 | POST |
| sciura1.131 | sciura | 1 | 1.13 | roll | 0 | 0 | 2 | f | f | 0.25139664 | 0.24581005 | 0.036781609 | POST |
| sciura1.211 | sciura | 1 | 1.21 | ruga | 0 | 0 | 1 | f | f | 0.36496335 | 0.19708029 | 0.004608295 | POST |
| sciura1.323 | sciura | 3 | 1.32 | ruga | 0 | 0 | 1 | f | f | 0.36496335 | 0.19708029 | 0.004608295 | POST |
| sciura1.351 | sciura | 1 | 1.35 | ruga | only seen | 0 | 3 | f | f | 0.36496335 | 0.19708029 | 0.004608295 | POST |
| sciura1.351 | sciura | 1 | 1.35 | sfasciato | 0 | 0 | 3 | f | m | 0.05882352 | 0.05882352 | 0.024390244 | POST |
| sciura1.351 | sciura | 1 | 1.35 | roll | 0 | 0 | 3 | f | f | 0.25139664 | 0.24581005 | 0.036781609 | POST |
| sciura1.413 | sciura | 3 | 1.41 | sine | only heard | 0 | 1 | f | f | 0.43103448 | 0.06896551 | 0.050682261 | POST |
| sciura1.443 | sciura | 3 | 1.44 | sine | seen and heard | 0 | 1 | f | f | 0.43103448 | 0.06896551 | 0.050682261 | POST |
| sciura1.441 | sciura | 1 | 1.44 | roll | only seen | 0 | 4 | f | f | 0.25139664 | 0.24581005 | 0.036781609 | POST |
| sciura1.441 | sciura | 1 | 1.44 | sguercia | only seen | 1 | 4 | f | f | 0 | 0 | 0 | POST |
| sciura1.441 | sciura | 1 | 1.44 | sonia | 0 | 0 | 4 | f | f | 0.26153846 | 0.04615384 | 0.041666667 | POST |
| sciura1.441 | sciura | 1 | 1.44 | neomamma | 0 | 0 | 4 | f | f | 0.16 | 0.21333333 | 0.032840722 | POST |
| sciura1.461 | sciura | 1 | 1.46 | ruga | seen and heard | 0 | 2 | f | f | 0.36496335 | 0.19708029 | 0.004608295 | POST |
| sciura1.461 | sciura | 1 | 1.46 | spilla | only heard | 0 | 2 | f | f | 0.21875 | 0.109375 | 0.043678161 | POST |
| sciura1.512 | sciura | 2 | 1.51 | ruga | only heard | 0 | 1 | f | f | 0.36496335 | 0.19708029 | 0.004608295 | POST |
| sciura1.511 | sciura | 1 | 1.51 | spilla | 0 | 0 | 1 | f | f | 0.21875 | 0.109375 | 0.043678161 | POST |
| sciura1.653 | sciura | 3 | 1.65 | roll | only seen | 0 | 2 | f | f | 0.25139664 | 0.24581005 | 0.036781609 | POST |
| sciura1.653 | sciura | 3 | 1.65 | ruga | 0 | 0 | 2 | f | f | 0.36496335 | 0.19708029 | 0.004608295 | POST |
| sciura1.821 | sciura | 1 | 1.82 | spilla | only heard | 0 | 2 | f | f | 0.21875 | 0.109375 | 0.043678161 | POST |
| sciura1.821 | sciura | 1 | 1.82 | roll | only heard | 1 | 2 | f | f | 0.25139664 | 0.24581005 | 0.036781609 | POST |
| sciura1.842 | sciura | 2 | 1.84 | sonia | 0 | 0 | 1 | f | f | 0.26153846 | 0.04615384 | 0.041666667 | POST |
| sciura1.91 | sciura | 1 | 1.9 | ruga | 0 | 0 | 2 | f | f | 0.36496335 | 0.19708029 | 0.004608295 | POST |
| sciura1.91 | sciura | 1 | 1.9 | roll | 0 | 0 | 2 | f | f | 0.25139664 | 0.24581005 | 0.036781609 | POST |
| sciura1.9261 | sciura | 1 | 1.926 | neomamma | only seen | 0 | 7 | f | f | 0.16 | 0.21333333 | 0.032840722 | POST |
| sciura1.9261 | sciura | 1 | 1.926 | sine | only seen | 0 | 7 | f | f | 0.43103448 | 0.06896551 | 0.050682261 | POST |
| sciura1.9261 | sciura | 1 | 1.926 | tino | 0 | 0 | 7 | f | m | 0 | 0 | 0.111111111 | POST |
| sciura1.9261 | sciura | 1 | 1.926 | strega | 0 | 0 | 7 | f | f | 0 | 0.23529411 | 0.016025641 | POST |
| sciura1.9261 | sciura | 1 | 1.926 | sumo | 0 | 0 | 7 | f | m | 0.04 | 0.08 | 0.203125 | POST |
| sciura1.9261 | sciura | 1 | 1.926 | spilla | 0 | 0 | 7 | f | f | 0.21875 | 0.109375 | 0.043678161 | POST |
| sciura1.9261 | sciura | 1 | 1.926 | schiarita | 0 | 0 | 7 | f | f | 0 | 0.23073923 | 0.041420118 | POST |
| sciura1.931 | sciura | 1 | 1.93 | stella | seen and heard | 0 | 7 | f | f | 0 | 0.14285714 | 0.020080321 | POST |
| sciura1.931 | sciura | 1 | 1.93 | osso | only heard | 0 | 7 | f | f | 0.15384615 | 0.01846153 | 0.03030303 | POST |
| sciura1.931 | sciura | 1 | 1.93 | roll | only heard | 0 | 7 | f | f | 0.25139664 | 0.24581005 | 0.036781609 | POST |
| sciura1.931 | sciura | 1 | 1.93 | sfasciato | only heard | 0 | 7 | f | m | 0.05882352 | 0.05882352 | 0.024390244 | POST |
| sciura1.931 | sciura | 1 | 1.93 | secco | only heard | 0 | 7 | f | m | 0 | 0 | 0.123076923 | POST |
| sciura1.931 | sciura | 1 | 1.93 | ruga | only heard | 0 | 7 | f | f | 0.36496335 | 0.19708029 | 0.004608295 | POST |
| sciura1.931 | sciura | 1 | 1.93 | sonia | only heard | 0 | 7 | f | f | 0.26153846 | 0.04615384 | 0.041666667 | POST |
| sciura1.941 | sciura | 1 | 1.94 | ruga | only seen | 0 | 2 | f | f | 0.36496335 | 0.19708029 | 0.004608295 | POST |
| sciura1.942 | sciura | 2 | 1.94 | secco | 0 | 0 | 1 | f | m | 0 | 0 | 0.123076923 | POST |
| sciura1.941 | sciura | 1 | 1.94 | roll | 0 | 0 | 2 | f | f | 0.25139664 | 0.24581005 | 0.036781609 | POST |
| sciura2.121 | sciura | 1 | 2.12 | ruga | 0 | 0 | 1 | f | f | 0.36496335 | 0.19708029 | 0.004608295 | POST |
| sciura2.183 | sciura | 3 | 2.18 | sonia | only heard | 0 | 3 | f | f | 0.26153846 | 0.04615384 | 0.041666667 | POST |
| sciura2.183 | sciura | 3 | 2.18 | roll | only heard | 0 | 3 | f | f | 0.25139664 | 0.24581005 | 0.036781609 | POST |
| sciura2.183 | sciura | 3 | 2.18 | neomamma | only heard | 0 | 3 | f | f | 0.16 | 0.21333333 | 0.032840722 | POST |
| sciura2.331 | sciura | 1 | 2.33 | ruga | only seen | 0 | 2 | f | f | 0.36496335 | 0.19708029 | 0.004608295 | POST |
| sciura2.331 | sciura | 1 | 2.33 | roll | 0 | 0 | 2 | f | f | 0.25139664 | 0.24581005 | 0.036781609 | POST |
| sciura2.481 | sciura | 1 | 2.48 | tris | seen and heard | 1 | 5 | f | f | 0.1617647 | 0.1617647 | 0.010449321 | POST |
| sciura2.481 | sciura | 1 | 2.48 | spilla | seen and heard | 1 | 5 | f | f | 0.21875 | 0.109375 | 0.043678161 | POST |
| sciura2.481 | sciura | 1 | 2.48 | roll | seen and heard | 0 | 5 | f | f | 0.25139664 | 0.24581005 | 0.036781609 | POST |
| sciura2.481 | sciura | 1 | 2.48 | severa | seen and heard | 0 | 5 | f | f | 0.02380952 | 0 | 0.066176471 | POST |
| sciura2.481 | sciura | 1 | 2.48 | osso | only heard | 0 | 5 | f | f | 0.15384615 | 0.03846153 | 0.03030303 | POST |
| sciura2.48* | sciura | * | 2.48 | roll | 0 | 0 | 2 | f | f | 0.25139664 | 0.24581005 | 0.036781609 | POST |
| sciura2.491 | sciura | 1 | 2.49 | ruga | only seen | 0 | 2 | f | f | 0.36496335 | 0.19708029 | 0.004608295 | POST |
| sciura2.491 | sciura | 1 | 2.49 | roll | 0 | 0 | 2 | f | f | 0.25139664 | 0.24581005 | 0.036781609 | POST |
| sciura2.531 | sciura | 1 | 2.53 | roll | only seen | 0 | 4 | f | f | 0.25139664 | 0.24581005 | 0.036781609 | POST |
| sciura2.531 | sciura | 1 | 2.53 | ruga | 0 | 0 | 4 | f | f | 0.36496335 | 0.19708029 | 0.004608295 | POST |
| sciura2.531 | sciura | 1 | 2.53 | sally | 0 | 0 | 4 | f | f | 0.04347826 | 0.08695652 | 0.002949853 | POST |
| sciura2.531 | sciura | 1 | 2.53 | schiarita | 0 | 1 | 4 | f | f | 0 | 0.23073923 | 0.041420118 | POST |
| sciura2.661 | sciura | 1 | 2.66 | sonia | only seen | 0 | 3 | f | f | 0.26153846 | 0.04615384 | 0.041666667 | POST |
| sciura2.661 | sciura | 1 | 2.66 | ruga | 0 | 0 | 3 | f | f | 0.36496335 | 0.19708029 | 0.004608295 | POST |
| sciura2.661 | sciura | 1 | 2.66 | roll | 0 | 0 | 3 | f | f | 0.25139664 | 0.24581005 | 0.036781609 | POST |
| sciura2.681 | sciura | 1 | 2.68 | roll | only heard | 0 | 2 | f | f | 0.25139664 | 0.24581005 | 0.036781609 | PRE |
| sciura2.681 | sciura | 1 | 2.68 | ruga | only heard | 0 | 2 | f | f | 0.36496335 | 0.19708029 | 0.004608295 | PRE |
| sciura2.711 | sciura | 1 | 2.71 | ruga | only seen | 0 | 1 | f | f | 0.36496335 | 0.19708029 | 0.004608295 | PRE |
| sciura2.723 | sciura | 3 | 2.72 | roll | only seen | 0 | 2 | f | f | 0.25139664 | 0.24581005 | 0.036781609 | PRE |
| sciura2.723 | sciura | 3 | 2.72 | ruga | 0 | 0 | 2 | f | f | 0.36496335 | 0.19708029 | 0.004608295 | PRE |
| sciura2.761 | sciura | 1 | 2.76 | spot | 0 | 0 | 3 | f | f | 0.21568627 | 0.1372549 | 0.020576132 | PRE |
| sciura2.761 | sciura | 1 | 2.76 | sine | 0 | 0 | 3 | f | f | 0.43103448 | 0.06896551 | 0.050682261 | PRE |
| sciura2.761 | sciura | 1 | 2.76 | stacy | 0 | 0 | 3 | f | f | 0.02857142 | 0.14285714 | 0.021317829 | PRE |
| sciura2.882 | sciura | 2 | 2.88 | ruga | seen and heard | 0 | 2 | f | f | 0.36496335 | 0.19708029 | 0.004608295 | PRE |
| sciura2.882 | sciura | 2 | 2.88 | roll | only heard | 1 | 2 | f | f | 0.25139664 | 0.24581005 | 0.036781609 | PRE |
| sciura3.011 | sciura | 1 | 3.01 | scapola | 0 | 0 | 1 | f | f | 0 | 0.28571428 | 0.003787879 | PRE |
| sciura3.022 | sciura | 2 | 3.02 | ruga | 0 | 0 | 1 | f | f | 0.36496335 | 0.19708029 | 0.004608295 | PRE |
| sciura3.411 | sciura | 1 | 3.41 | sine | seen and heard | 0 | 3 | f | f | 0.43103448 | 0.06896551 | 0.050682261 | PRE |
| sciura3.411 | sciura | 1 | 3.41 | spot | 0 | 0 | 3 | f | f | 0.21568627 | 0.1372549 | 0.020576132 | PRE |
| sciura3.411 | sciura | 1 | 3.41 | stacy | 0 | 0 | 3 | f | f | 0.02857142 | 0.14285714 | 0.021317829 | PRE |
| sciura3.592 | sciura | 2 | 3.59 | tris | 0 | 0 | 2 | f | f | 0.1617647 | 0.1617647 | 0.010449321 | PRE |
| sciura3.592 | sciura | 2 | 3.59 | ruga | 0 | 0 | 2 | f | f | 0.36496335 | 0.19708029 | 0.004608295 | PRE |
| sciura3.973 | sciura | 3 | 3.97 | ruga | only seen | 0 | 2 | f | f | 0.36496335 | 0.19708029 | 0.004608295 | PRE |
| sciura3.973 | sciura | 3 | 3.97 | roll | 0 | 0 | 2 | f | f | 0.25139664 | 0.24581005 | 0.036781609 | PRE |
| sciura4.043 | sciura | 3 | 4.04 | roll | only seen | 0 | 2 | f | f | 0.25139664 | 0.24581005 | 0.036781609 | PRE |
| sciura4.043 | sciura | 3 | 4.04 | ruga | only seen | 0 | 2 | f | f | 0.36496335 | 0.19708029 | 0.004608295 | PRE |
| sciura4.083 | sciura | 3 | 4.08 | sonia | only seen | 0 | 2 | f | f | 0.26153846 | 0.04615384 | 0.041666667 | PRE |
| sciura4.083 | sciura | 3 | 4.08 | roll | 0 | 0 | 2 | f | f | 0.25139664 | 0.24581005 | 0.036781609 | PRE |
| sciura5.141 | sciura | 1 | 5.14 | roll | only seen | 0 | 2 | f | f | 0.25139664 | 0.24581005 | 0.036781609 | PRE |
| sciura5.141 | sciura | 1 | 5.14 | ruga | only seen | 0 | 2 | f | f | 0.36496335 | 0.19708029 | 0.004608295 | PRE |
| sciura5.181 | sciura | 1 | 5.18 | ruga | only seen | 0 | 8 | f | f | 0.36496335 | 0.19708029 | 0.004608295 | PRE |
| sciura5.181 | sciura | 1 | 5.18 | sorcia | 0 | 0 | 8 | f | f | 0 | 0.02272727 | 0.022727273 | PRE |
| sciura5.181 | sciura | 1 | 5.18 | spilla | 0 | 0 | 8 | f | f | 0.21875 | 0.109375 | 0.043678161 | PRE |
| sciura5.181 | sciura | 1 | 5.18 | severa | 0 | 0 | 8 | f | f | 0.02380952 | 0 | 0.066176471 | PRE |
| sciura5.181 | sciura | 1 | 5.18 | roll | 0 | 0 | 8 | f | f | 0.25139664 | 0.24581005 | 0.036781609 | PRE |
| sciura5.181 | sciura | 1 | 5.18 | tris | 0 | 0 | 8 | f | f | 0.1617647 | 0.1617647 | 0.010449321 | PRE |
| sciura5.181 | sciura | 1 | 5.18 | sonia | 0 | 1 | 8 | f | f | 0.26153846 | 0.04615384 | 0.041666667 | PRE |
| sciura5.181 | sciura | 1 | 5.18 | stella | 0 | 0 | 8 | f | f | 0 | 0.14285714 | 0.020080321 | PRE |
| secco0.842 | secco | 2 | 0.84 | scapola | only seen | 0 | 7 | m | f | 0.08888889 | 0.06666667 | 0.003787879 | POST |
| secco0.842 | secco | 2 | 0.84 | sally | only seen | 0 | 7 | m | f | 0 | 0 | 0.002949853 | POST |
| secco0.842 | secco | 2 | 0.84 | stacy | only seen | 0 | 7 | m | f | 0.01960784 | 0.07843137 | 0.021317829 | POST |
| secco0.842 | secco | 2 | 0.84 | susy | only seen | 0 | 7 | m | f | 0.02631578 | 0.02631578 | 0.027777778 | POST |
| secco0.842 | secco | 2 | 0.84 | spot | only seen | 0 | 7 | m | f | 0.02564102 | 0.15384615 | 0.020576132 | POST |
| secco0.842 | secco | 2 | 0.84 | sine | 0 | 0 | 7 | m | f | 0.125 | 0.08333333 | 0.050682261 | POST |
| secco0.842 | secco | 2 | 0.84 | neomamma | 0 | 0 | 7 | m | f | 0 | 0.11111111 | 0.032840722 | POST |
| secco1.192 | secco | 2 | 1.19 | strega | only seen | 0 | 1 | m | f | 0.09302325 | 0.04651162 | 0.016025641 | POST |
| secco1.21 | secco | 1 | 1.2 | stacy | only seen | 0 | 8 | m | f | 0.01960784 | 0.07843137 | 0.021317829 | POST |
| secco1.21 | secco | 1 | 1.2 | sonia | 0 | 0 | 8 | m | f | 0 | 0.27272727 | 0.041666667 | POST |
| secco1.21 | secco | 1 | 1.2 | spiga | 0 | 0 | 8 | m | f | 0 | 0.08108108 | 0.018372703 | POST |
| secco1.21 | secco | 1 | 1.2 | scapola | 0 | 0 | 8 | m | f | 0.08888889 | 0.06666667 | 0.003787879 | POST |
| secco1.21 | secco | 1 | 1.2 | rosa | 0 | 0 | 8 | m | f | 0.10344827 | 0 | 0.027131783 | POST |
| secco1.21 | secco | 1 | 1.2 | neomamma | 0 | 0 | 8 | m | f | 0 | 0.11111111 | 0.032840722 | POST |
| secco1.21 | secco | 1 | 1.2 | strega | 0 | 0 | 8 | m | f | 0.09302325 | 0.04651162 | 0.016025641 | POST |
| secco1.21 | secco | 1 | 1.2 | small | 0 | 0 | 8 | m | f | 0 | 0.20833333 | 0.044117647 | POST |
| secco1.313 | secco | 3 | 1.31 | sally | only seen | 0 | 4 | m | f | 0 | 0 | 0.002949853 | POST |
| secco1.313 | secco | 3 | 1.31 | sfasciato | only seen | 0 | 4 | m | m | 0 | 0.15384615 | 0.024390244 | POST |
| secco1.313 | secco | 3 | 1.31 | sine | only seen | 0 | 4 | m | f | 0.125 | 0.08333333 | 0.050682261 | POST |
| secco1.313 | secco | 3 | 1.31 | neomamma | 0 | 0 | 4 | m | f | 0 | 0.11111111 | 0.032840722 | POST |
| secco1.423 | secco | 3 | 1.42 | neomamma | only seen | 0 | 7 | m | f | 0 | 0.11111111 | 0.032840722 | POST |
| secco1.423 | secco | 3 | 1.42 | susy | only seen | 1 | 7 | m | f | 0.02631578 | 0.02631578 | 0.027777778 | POST |
| secco1.423 | secco | 3 | 1.42 | spot | only seen | 0 | 7 | m | f | 0.02564102 | 0.15384615 | 0.020576132 | POST |
| secco1.423 | secco | 3 | 1.42 | scapola | only seen | 0 | 7 | m | f | 0.08888889 | 0.06666667 | 0.003787879 | POST |
| secco1.423 | secco | 3 | 1.42 | spiga | only seen | 0 | 7 | m | f | 0 | 0.08108108 | 0.018372703 | POST |
| secco1.423 | secco | 3 | 1.42 | sally | only seen | 0 | 7 | m | f | 0 | 0 | 0.002949853 | POST |
| secco1.423 | secco | 3 | 1.42 | rosa | only seen | 0 | 7 | m | f | 0.10344827 | 0 | 0.027131783 | POST |
| secco1.433 | secco | 3 | 1.43 | spot | only seen | 0 | 9 | m | f | 0.02564102 | 0.15384615 | 0.020576132 | POST |
| secco1.433 | secco | 3 | 1.43 | rosa | only seen | 0 | 9 | m | f | 0.10344827 | 0 | 0.027131783 | POST |
| secco1.433 | secco | 3 | 1.43 | susy | only seen | 0 | 11 | m | f | 0.02631578 | 0.02631578 | 0.027777778 | POST |
| secco1.433 | secco | 3 | 1.43 | strega | only seen | 0 | 11 | m | f | 0.09302325 | 0.04651162 | 0.016025641 | POST |
| secco1.433 | secco | 3 | 1.43 | schiarita | 0 | 0 | 9 | m | f | 0 | 0.1 | 0.041420118 | POST |
| secco1.433 | secco | 3 | 1.43 | spiga | 0 | 0 | 9 | m | f | 0 | 0.08108108 | 0.018372703 | PRE |
| secco1.433 | secco | 3 | 1.43 | scapola | 0 | 0 | 9 | m | f | 0.08888889 | 0.06666667 | 0.003787879 | PRE |
| secco1.433 | secco | 3 | 1.43 | neomamma | 0 | 0 | 9 | m | f | 0 | 0.11111111 | 0.032840722 | PRE |
| secco1.433 | secco | 3 | 1.43 | strega | 0 | 0 | 9 | m | f | 0.09302325 | 0.04651162 | 0.016025641 | PRE |
| secco1.433 | secco | 3 | 1.43 | small | 0 | 0 | 9 | m | f | 0 | 0.20833333 | 0.044117647 | PRE |
| secco1.433 | secco | 3 | 1.43 | sonia | 0 | 0 | 9 | m | f | 0 | 0.27272727 | 0.041666667 | PRE |
| secco1.433 | secco | 3 | 1.43 | stacy | 0 | 0 | 11 | m | f | 0.01960784 | 0.07843137 | 0.021317829 | PRE |
| secco1.433 | secco | 3 | 1.43 | scapola | 0 | 0 | 11 | m | f | 0.08888889 | 0.06666667 | 0.003787879 | PRE |
| secco1.433 | secco | 3 | 1.43 | sfasciato | 0 | 0 | 11 | m | m | 0 | 0.15384615 | 0.024390244 | PRE |
| secco1.433 | secco | 3 | 1.43 | sally | 0 | 0 | 11 | m | f | 0 | 0 | 0.002949853 | PRE |
| secco1.433 | secco | 3 | 1.43 | schiarita | 0 | 0 | 11 | m | f | 0 | 0.1 | 0.041420118 | PRE |
| secco1.433 | secco | 3 | 1.43 | sine | 0 | 0 | 11 | m | f | 0.125 | 0.08333333 | 0.050682261 | PRE |
| secco1.433 | secco | 3 | 1.43 | susy | 0 | 0 | 11 | m | f | 0.02631578 | 0.02631578 | 0.027777778 | PRE |
| secco1.433 | secco | 3 | 1.43 | spot | 0 | 0 | 11 | m | f | 0.02564102 | 0.15384615 | 0.020576132 | PRE |
| secco1.433 | secco | 3 | 1.43 | small | 0 | 0 | 11 | m | f | 0 | 0.20833333 | 0.044117647 | PRE |
| secco1.461 | secco | 1 | 1.46 | strega | 0 | 0 | 1 | m | f | 0.09302325 | 0.04651162 | 0.016025641 | PRE |
| secco1.483 | secco | 3 | 1.48 | spiga | only seen | 0 | 2 | m | f | 0 | 0.08108108 | 0.018372703 | PRE |
| secco1.483 | secco | 3 | 1.48 | rosa | 0 | 0 | 2 | m | f | 0.10344827 | 0 | 0.027131783 | PRE |
| secco1.51 | secco | 1 | 1.5 | sine | only seen | 0 | 8 | m | f | 0.125 | 0.08333333 | 0.050682261 | PRE |
| secco1.51 | secco | 1 | 1.5 | scapola | 0 | 0 | 8 | m | f | 0.08888889 | 0.06666667 | 0.003787879 | PRE |
| secco1.51 | secco | 1 | 1.5 | sfasciato | 0 | 0 | 8 | m | m | 0 | 0.15384615 | 0.024390244 | PRE |
| secco1.51 | secco | 1 | 1.5 | spot | 0 | 0 | 8 | m | f | 0.02564102 | 0.15384615 | 0.020576132 | PRE |
| secco1.51 | secco | 1 | 1.5 | schiarita | 0 | 0 | 8 | m | f | 0 | 0.1 | 0.041420118 | PRE |
| secco1.51 | secco | 1 | 1.5 | strega | 0 | 0 | 8 | m | f | 0.09302325 | 0.04651162 | 0.016025641 | PRE |
| secco1.51 | secco | 1 | 1.5 | neomamma | 0 | 0 | 8 | m | f | 0 | 0.11111111 | 0.032840722 | PRE |
| secco1.51 | secco | 1 | 1.5 | stacy | 0 | 0 | 8 | m | f | 0.01960784 | 0.07843137 | 0.021317829 | PRE |
| secco1.883 | secco | 3 | 1.88 | small | only seen | 0 | 5 | m | f | 0 | 0.20833333 | 0.044117647 | PRE |
| secco1.883 | secco | 3 | 1.88 | spot | only seen | 0 | 5 | m | f | 0.02564102 | 0.15384615 | 0.020576132 | PRE |
| secco1.883 | secco | 3 | 1.88 | spiga | 0 | 0 | 5 | m | f | 0 | 0.08108108 | 0.018372703 | PRE |
| secco1.883 | secco | 3 | 1.88 | sfasciato | 0 | 0 | 5 | m | m | 0 | 0.15384615 | 0.024390244 | PRE |
| secco1.883 | secco | 3 | 1.88 | strega | 0 | 0 | 5 | m | f | 0.09302325 | 0.04651162 | 0.016025641 | PRE |
| secco1.973 | secco | 3 | 1.97 | strega | 0 | 0 | 1 | m | f | 0.09302325 | 0.04651162 | 0.016025641 | PRE |
| secco2.193 | secco | 3 | 2.19 | sine | seen and heard | 0 | 6 | m | f | 0.125 | 0.08333333 | 0.050682261 | PRE |
| secco2.193 | secco | 3 | 2.19 | susy | seen and heard | 0 | 6 | m | f | 0.02631578 | 0.02631578 | 0.027777778 | PRE |
| secco2.193 | secco | 3 | 2.19 | stacy | seen and heard | 0 | 6 | m | f | 0.01960784 | 0.07843137 | 0.021317829 | PRE |
| secco2.193 | secco | 3 | 2.19 | scapola | only heard | 0 | 6 | m | f | 0.08888889 | 0.06666667 | 0.003787879 | PRE |
| secco2.193 | secco | 3 | 2.19 | sfasciato | only heard | 0 | 6 | m | m | 0 | 0.15384615 | 0.024390244 | PRE |
| secco2.193 | secco | 3 | 2.19 | strega | only heard | 0 | 6 | m | f | 0.09302325 | 0.04651162 | 0.016025641 | PRE |
| secco2.533 | secco | 3 | 2.53 | susy | only seen | 0 | 2 | m | f | 0.02631578 | 0.02631578 | 0.027777778 | PRE |
| secco3.023 | secco | 3 | 3.02 | spot | only seen | 0 | 7 | m | f | 0.02564102 | 0.15384615 | 0.020576132 | PRE |
| secco3.023 | secco | 3 | 3.02 | sine | 0 | 0 | 7 | m | f | 0.125 | 0.08333333 | 0.050682261 | PRE |
| secco3.023 | secco | 3 | 3.02 | scapola | 0 | 0 | 7 | m | f | 0.08888889 | 0.06666667 | 0.003787879 | PRE |
| secco3.023 | secco | 3 | 3.02 | sally | 0 | 0 | 7 | m | f | 0 | 0 | 0.002949853 | PRE |
| secco3.023 | secco | 3 | 3.02 | susy | 0 | 0 | 7 | m | f | 0.02631578 | 0.02631578 | 0.027777778 | PRE |
| secco3.023 | secco | 3 | 3.02 | stacy | 0 | 0 | 7 | m | f | 0.01960784 | 0.07843137 | 0.021317829 | PRE |
| secco3.023 | secco | 3 | 3.02 | neomamma | 0 | 0 | 7 | m | f | 0 | 0.11111111 | 0.032840722 | PRE |
| severa0.98* | severa | * | 0.98 | roll | 0 | 0 | 3 | f | f | 0.02083333 | 0 | 0.036781609 | POST |
| severa0.98* | severa | * | 0.98 | osso | 0 | 0 | 3 | f | f | 0.27536231 | 0.11594202 | 0.03030303 | POST |
| severa0.98* | severa | * | 0.98 | tris | 0 | 0 | 3 | f | f | 0.12820512 | 0.14102564 | 0.010449321 | POST |
| severa1.241 | severa | 1 | 1.24 | osso | seen and heard | 0 | 2 | f | f | 0.27536231 | 0.11594202 | 0.03030303 | POST |
| severa1.241 | severa | 1 | 1.24 | sguercia | only heard | 1 | 2 | f | f | 0 | 0.21212121 | 0 | POST |
| severa1.281 | severa | 1 | 1.28 | tris | only heard | 0 | 3 | f | f | 0.12820512 | 0.14102564 | 0.010449321 | POST |
| severa1.281 | severa | 1 | 1.28 | sciura | only heard | 0 | 3 | f | f | 0.02380952 | 0 | 0.06377858 | POST |
| severa1.281 | severa | 1 | 1.28 | roll | only heard | 0 | 3 | f | f | 0.02083333 | 0 | 0.036781609 | POST |
| severa1.362 | severa | 2 | 1.36 | sorcia | 0 | 0 | 2 | f | f | 0 | 0.05714285 | 0.022727273 | POST |
| severa1.362 | severa | 2 | 1.36 | sguercia | 0 | 0 | 2 | f | f | 0 | 0.21212121 | 0 | POST |
| severa1.441 | severa | 1 | 1.44 | spilla | only heard | 0 | 1 | f | f | 0.15463917 | 0.16494845 | 0.043678161 | POST |
| severa1.51 | severa | 1 | 1.5 | spilla | 0 | 0 | 1 | f | f | 0.15463917 | 0.16494845 | 0.043678161 | POST |
| severa1.531 | severa | 1 | 1.53 | spilla | only seen | 0 | 2 | f | f | 0.15463917 | 0.16494845 | 0.043678161 | POST |
| severa1.531 | severa | 1 | 1.53 | sonia | only seen | 0 | 2 | f | f | 0 | 0.26315789 | 0.041666667 | POST |
| severa1.651 | severa | 1 | 1.65 | sonia | only seen | 1 | 5 | f | f | 0 | 0.26315789 | 0.041666667 | POST |
| severa1.651 | severa | 1 | 1.65 | osso | only seen | 0 | 5 | f | f | 0.27536231 | 0.11594202 | 0.03030303 | POST |
| severa1.651 | severa | 1 | 1.65 | spilla | 0 | 0 | 5 | f | f | 0.15463917 | 0.16494845 | 0.043678161 | POST |
| severa1.651 | severa | 1 | 1.65 | stella | 0 | 0 | 5 | f | f | 0 | 0 | 0.020080321 | POST |
| severa1.651 | severa | 1 | 1.65 | tris | 0 | 0 | 5 | f | f | 0.12820512 | 0.14102564 | 0.010449321 | POST |
| severa1.661 | severa | 1 | 1.66 | tris | only seen | 0 | 5 | f | f | 0.12820512 | 0.14102564 | 0.010449321 | POST |
| severa1.661 | severa | 1 | 1.66 | sonia | 0 | 0 | 5 | f | f | 0 | 0.26315789 | 0.041666667 | POST |
| severa1.661 | severa | 1 | 1.66 | spilla | 0 | 1 | 5 | f | f | 0.15463917 | 0.16494845 | 0.043678161 | POST |
| severa1.661 | severa | 1 | 1.66 | osso | 0 | 0 | 5 | f | f | 0.27536231 | 0.11594202 | 0.03030303 | POST |
| severa1.661 | severa | 1 | 1.66 | stella | 0 | 0 | 5 | f | f | 0 | 0 | 0.020080321 | POST |
| severa1.681 | severa | 1 | 1.68 | spilla | only seen | 0 | 4 | f | f | 0.15463917 | 0.16494845 | 0.043678161 | POST |
| severa1.681 | severa | 1 | 1.68 | sonia | 0 | 0 | 4 | f | f | 0 | 0.26315789 | 0.041666667 | POST |
| severa1.681 | severa | 1 | 1.68 | osso | 0 | 0 | 4 | f | f | 0.27536231 | 0.11594202 | 0.03030303 | POST |
| severa1.681 | severa | 1 | 1.68 | tris | 0 | 0 | 4 | f | f | 0.12820512 | 0.14102564 | 0.010449321 | POST |
| severa1.841 | severa | 1 | 1.84 | tris | only seen | 0 | 3 | f | f | 0.12820512 | 0.14102564 | 0.010449321 | POST |
| severa1.841 | severa | 1 | 1.84 | osso | 0 | 0 | 3 | f | f | 0.27536231 | 0.11594202 | 0.03030303 | PRE |
| severa1.841 | severa | 1 | 1.84 | spilla | 0 | 0 | 3 | f | f | 0.15463917 | 0.16494845 | 0.043678161 | PRE |
| severa1.851 | severa | 1 | 1.85 | tris | only seen | 0 | 2 | f | f | 0.12820512 | 0.14102564 | 0.010449321 | PRE |
| severa1.851 | severa | 1 | 1.85 | osso | 0 | 0 | 2 | f | f | 0.27536231 | 0.11594202 | 0.03030303 | PRE |
| severa1.871 | severa | 1 | 1.87 | tris | only seen | 0 | 3 | f | f | 0.12820512 | 0.14102564 | 0.010449321 | PRE |
| severa1.871 | severa | 1 | 1.87 | spilla | only seen | 1 | 3 | f | f | 0.15463917 | 0.16494845 | 0.043678161 | PRE |
| severa1.871 | severa | 1 | 1.87 | stella | 0 | 0 | 3 | f | f | 0 | 0 | 0.020080321 | PRE |
| severa1.911 | severa | 1 | 1.91 | tris | 0 | 0 | 2 | f | f | 0.12820512 | 0.14102564 | 0.010449321 | PRE |
| severa1.911 | severa | 1 | 1.91 | osso | 0 | 0 | 2 | f | f | 0.27536231 | 0.11594202 | 0.03030303 | PRE |
| severa1.971 | severa | 1 | 1.97 | osso | 0 | 0 | 3 | f | f | 0.27536231 | 0.11594202 | 0.03030303 | PRE |
| severa1.971 | severa | 1 | 1.97 | tris | 0 | 0 | 3 | f | f | 0.12820512 | 0.14102564 | 0.010449321 | PRE |
| severa1.971 | severa | 1 | 1.97 | stella | 0 | 0 | 3 | f | f | 0 | 0 | 0.020080321 | PRE |
| severa2.1* | severa | * | 2.1 | stella | 0 | 0 | 5 | f | f | 0 | 0 | 0.020080321 | PRE |
| severa2.1* | severa | * | 2.1 | osso | 0 | 0 | 5 | f | f | 0.27536231 | 0.11594202 | 0.03030303 | PRE |
| severa2.151 | severa | 1 | 2.15 | osso | 0 | 0 | 4 | f | f | 0.27536231 | 0.11594202 | 0.03030303 | PRE |
| severa2.151 | severa | 1 | 2.15 | tris | 0 | 0 | 4 | f | f | 0.12820512 | 0.14102564 | 0.010449321 | PRE |
| severa2.191 | severa | 1 | 2.19 | spilla | 0 | 0 | 1 | f | f | 0.15463917 | 0.16494845 | 0.043678161 | PRE |
| severa2.311 | severa | 1 | 2.31 | spilla | only heard | 0 | 3 | f | f | 0.15463917 | 0.16494845 | 0.043678161 | PRE |
| severa2.311 | severa | 1 | 2.31 | osso | only heard | 0 | 3 | f | f | 0.27536231 | 0.11594202 | 0.03030303 | PRE |
| severa2.311 | severa | 1 | 2.31 | tris | only heard | 0 | 3 | f | f | 0.12820512 | 0.14102564 | 0.010449321 | PRE |
| severa2.311 | severa | 1 | 2.31 | tris | only seen | 0 | 5 | f | f | 0.12820512 | 0.14102564 | 0.010449321 | PRE |
| severa2.311 | severa | 1 | 2.31 | sonia | only seen | 0 | 5 | f | f | 0 | 0.26315789 | 0.041666667 | PRE |
| severa2.311 | severa | 1 | 2.31 | osso | 0 | 0 | 5 | f | f | 0.27536231 | 0.11594202 | 0.03030303 | PRE |
| severa2.311 | severa | 1 | 2.31 | spilla | 0 | 0 | 5 | f | f | 0.15463917 | 0.16494845 | 0.043678161 | PRE |
| severa2.311 | severa | 1 | 2.31 | stella | 0 | 0 | 5 | f | f | 0 | 0 | 0.020080321 | PRE |
| severa2.431 | severa | 1 | 2.43 | roll | only seen | 0 | 4 | f | f | 0.02083333 | 0 | 0.036781609 | PRE |
| severa2.431 | severa | 1 | 2.43 | ruga | only seen | 0 | 4 | f | f | 0.07692307 | 0 | 0.004608295 | PRE |
| severa2.431 | severa | 1 | 2.43 | tris | 0 | 0 | 4 | f | f | 0.12820512 | 0.14102564 | 0.010449321 | PRE |
| severa2.431 | severa | 1 | 2.43 | spilla | 0 | 0 | 4 | f | f | 0.15463917 | 0.16494845 | 0.043678161 | PRE |
| severa2.472 | severa | 2 | 2.47 | osso | 0 | 0 | 3 | f | f | 0.27536231 | 0.11594202 | 0.03030303 | PRE |
| severa2.472 | severa | 2 | 2.47 | tris | 0 | 0 | 3 | f | f | 0.12820512 | 0.14102564 | 0.010449321 | PRE |
| severa2.472 | severa | 2 | 2.47 | stella | 0 | 0 | 3 | f | f | 0 | 0 | 0.020080321 | PRE |
| severa2.472 | severa | 2 | 2.47 | osso | 0 | 0 | 3 | f | f | 0.27536231 | 0.11594202 | 0.03030303 | PRE |
| severa2.472 | severa | 2 | 2.47 | tris | 0 | 0 | 3 | f | f | 0.12820512 | 0.14102564 | 0.010449321 | PRE |
| severa2.472 | severa | 2 | 2.47 | stella | 0 | 0 | 3 | f | f | 0 | 0 | 0.020080321 | PRE |
| severa3.061 | severa | 1 | 3.06 | tris | 0 | 0 | 2 | f | f | 0.12820512 | 0.14102564 | 0.010449321 | PRE |
| severa3.061 | severa | 1 | 3.06 | sorcia | 0 | 0 | 2 | f | f | 0 | 0.05714285 | 0.022727273 | PRE |
| severa3.23* | severa | * | 3.23 | roll | 0 | 0 | 1 | f | f | 0.02083333 | 0 | 0.036781609 | PRE |
| severa3.372 | severa | 2 | 3.37 | spilla | 0 | 0 | 1 | f | f | 0.15463917 | 0.16494845 | 0.043678161 | PRE |
| severa4.251 | severa | 1 | 4.25 | roll | only heard | 1 | 3 | f | f | 0.02083333 | 0 | 0.036781609 | PRE |
| severa4.251 | severa | 1 | 4.25 | ruga | only heard | 0 | 3 | f | f | 0.07692307 | 0 | 0.004608295 | PRE |
| severa4.251 | severa | 1 | 4.25 | sciura | only heard | 0 | 3 | f | f | 0.02380952 | 0 | 0.06377858 | PRE |
| severa4.251 | severa | 1 | 4.25 | sonia | 0 | 0 | 3 | f | f | 0 | 0.26315789 | 0.041666667 | PRE |
| severa4.251 | severa | 1 | 4.25 | osso | 0 | 0 | 3 | f | f | 0.27536231 | 0.11594202 | 0.03030303 | PRE |
| severa4.251 | severa | 1 | 4.25 | tris | 0 | 0 | 3 | f | f | 0.12820512 | 0.14102564 | 0.010449321 | PRE |
| severa5.061 | severa | 1 | 5.06 | osso | 0 | 0 | 3 | f | f | 0.27536231 | 0.11594202 | 0.03030303 | PRE |
| severa5.061 | severa | 1 | 5.06 | tris | 0 | 0 | 3 | f | f | 0.12820512 | 0.14102564 | 0.010449321 | PRE |
| severa5.061 | severa | 1 | 5.06 | roll | 0 | 0 | 3 | f | f | 0.02083333 | 0 | 0.036781609 | PRE |
| sfasciato1.913 | sfasciato | 3 | 1.91 | sine | only seen | 0 | 12 | m | f | 0.25925926 | 0.05555556 | 0.050682261 | POST |
| sfasciato1.913 | sfasciato | 3 | 1.91 | rosa | 0 | 0 | 12 | m | f | 0 | 0.10344827 | 0.027131783 | POST |
| sfasciato1.913 | sfasciato | 3 | 1.91 | strega | 0 | 0 | 12 | m | f | 0 | 0.05405405 | 0.016025641 | POST |
| sfasciato1.913 | sfasciato | 3 | 1.91 | sorcia | 0 | 0 | 12 | m | f | 0 | 0.07692307 | 0.022727273 | POST |
| sfasciato1.913 | sfasciato | 3 | 1.91 | sguercia | 0 | 0 | 12 | m | f | 0 | 0 | 0 | POST |
| sfasciato1.913 | sfasciato | 3 | 1.91 | neomamma | 0 | 0 | 12 | m | f | 0 | 0.1 | 0.032840722 | POST |
| sfasciato1.913 | sfasciato | 3 | 1.91 | schiarita | 0 | 0 | 12 | m | f | 0 | 0.16666667 | 0.041420118 | POST |
| sfasciato1.913 | sfasciato | 3 | 1.91 | stacy | 0 | 0 | 12 | m | f | 0.13461538 | 0.07692307 | 0.021317829 | POST |
| sfasciato1.913 | sfasciato | 3 | 1.91 | sally | 0 | 0 | 12 | m | f | 0 | 0.07894736 | 0.002949853 | PRE |
| sfasciato1.913 | sfasciato | 3 | 1.91 | scapola | 0 | 0 | 12 | m | f | 0 | 0.13333333 | 0.003787879 | PRE |
| sfasciato1.913 | sfasciato | 3 | 1.91 | spilla | 0 | 0 | 12 | m | f | 0 | 0 | 0.043678161 | PRE |
| sfasciato1.913 | sfasciato | 3 | 1.91 | spot | 0 | 0 | 12 | m | f | 0 | 0.12244897 | 0.020576132 | PRE |
| sfasciato2.243 | sfasciato | 3 | 2.24 | scapola | 0 | 0 | 3 | m | f | 0 | 0.13333333 | 0.003787879 | PRE |
| sfasciato2.243 | sfasciato | 3 | 2.24 | susy | 0 | 0 | 3 | m | f | 0 | 0.1707317 | 0.027777778 | PRE |
| sfasciato2.243 | sfasciato | 3 | 2.24 | rosa | 0 | 0 | 3 | m | f | 0 | 0.10344827 | 0.027131783 | PRE |
| sfasciato2.592 | sfasciato | 2 | 2.59 | stacy | only seen | 0 | 1 | m | f | 0.13461538 | 0.07692307 | 0.021317829 | PRE |
| sfasciato5.681 | sfasciato | 1 | 5.68 | sally | only seen | 0 | 2 | m | f | 0 | 0.07894736 | 0.002949853 | PRE |
| sfasciato5.681 | sfasciato | 1 | 5.68 | stella | only seen | 0 | 2 | m | f | 0 | 0.083333333 | 0.020080321 | PRE |
| sguercia0.831 | sguercia | 1 | 0.83 | osso | 0 | 0 | 2 | f | f | 0 | 0.125 | 0.03030303 | POST |
| sguercia0.831 | sguercia | 1 | 0.83 | severa | 0 | 0 | 2 | f | f | 0 | 0.21212121 | 0.066176471 | POST |
| sguercia2.451 | sguercia | 1 | 2.45 | roll | 0 | 0 | 4 | f | f | 0 | 0 | 0.036781609 | POST |
| sguercia2.451 | sguercia | 1 | 2.45 | sonia | 0 | 0 | 4 | f | f | 0 | 0 | 0.041666667 | POST |
| sguercia2.451 | sguercia | 1 | 2.45 | neomamma | 0 | 0 | 4 | f | f | 0 | 0 | 0.032840722 | POST |
| sguercia2.451 | sguercia | 1 | 2.45 | spot | 0 | 0 | 4 | f | f | 0 | 0.07142857 | 0.020576132 | POST |
| sguercia3.673 | sguercia | 3 | 3.67 | schiarita | 0 | 1 | 9 | f | f | 0 | 0 | 0.041420118 | POST |
| sguercia3.673 | sguercia | 3 | 3.67 | spiga | 0 | 0 | 9 | f | f | 0 | 0 | 0.018372703 | POST |
| sguercia3.673 | sguercia | 3 | 3.67 | sonia | 0 | 0 | 9 | f | f | 0 | 0 | 0.041666667 | PRE |
| sguercia3.673 | sguercia | 3 | 3.67 | scapola | 0 | 0 | 9 | f | f | 0 | 0 | 0.003787879 | PRE |
| sguercia3.673 | sguercia | 3 | 3.67 | rosa | 0 | 0 | 9 | f | f | 0 | 0 | 0.027131783 | PRE |
| sguercia3.673 | sguercia | 3 | 3.67 | spot | 0 | 0 | 9 | f | f | 0 | 0.07142857 | 0.020576132 | PRE |
| sguercia3.673 | sguercia | 3 | 3.67 | neomamma | 0 | 0 | 9 | f | f | 0 | 0 | 0.032840722 | PRE |
| sguercia3.673 | sguercia | 3 | 3.67 | strega | 0 | 0 | 9 | f | f | 0.04761904 | 0 | 0.016025641 | PRE |
| sguercia3.673 | sguercia | 3 | 3.67 | small | 0 | 0 | 9 | f | f | 0 | 0 | 0.044117647 | PRE |
| sine0.661 | sine | 1 | 0.66 | stacy | seen and heard | 0 | 2 | f | f | 0.03 | 0.07 | 0.021317829 | POST |
| sine0.661 | sine | 1 | 0.66 | spot | only heard | 0 | 2 | f | f | 0.207207207 | 0.198198198 | 0.020576132 | POST |
| sine1.261 | sine | 1 | 1.26 | susy | 0 | 0 | 6 | f | f | 0.04938271 | 0.08641975 | 0.027777778 | POST |
| sine1.261 | sine | 1 | 1.26 | secco | 0 | 0 | 6 | f | m | 0.125 | 0.08333333 | 0.123076923 | POST |
| sine1.261 | sine | 1 | 1.26 | sfasciato | 0 | 0 | 6 | f | m | 0.25925926 | 0.05555556 | 0.024390244 | POST |
| sine1.261 | sine | 1 | 1.26 | spot | 0 | 0 | 6 | f | f | 0.207207207 | 0.198198198 | 0.020576132 | POST |
| sine1.261 | sine | 1 | 1.26 | strega | 0 | 0 | 6 | f | f | 0 | 0.08064516 | 0.016025641 | POST |
| sine1.261 | sine | 1 | 1.26 | scapola | 0 | 0 | 6 | f | f | 0 | 0.20833333 | 0.003787879 | POST |
| sine1.2661 | sine | 1 | 1.266 | sciura | only seen | 0 | 6 | f | f | 0.43103448 | 0.06896551 | 0.06377858 | POST |
| sine1.2661 | sine | 1 | 1.266 | spot | only seen | 0 | 6 | f | f | 0.207207207 | 0.198198198 | 0.020576132 | POST |
| sine1.2661 | sine | 1 | 1.266 | schiarita | only seen | 1 | 6 | f | f | 0.04494387 | 0.23595505 | 0.041420118 | POST |
| sine1.2661 | sine | 1 | 1.266 | sumo | 0 | 0 | 6 | f | m | 0.11764705 | 0 | 0.203125 | POST |
| sine1.2661 | sine | 1 | 1.266 | tino | 0 | 0 | 6 | f | m | 0 | 0 | 0.111111111 | POST |
| sine1.2661 | sine | 1 | 1.266 | neomamma | 0 | 0 | 6 | f | f | 0.0420168 | 0.20168067 | 0.032840722 | POST |
| sine1.331 | sine | 1 | 1.33 | spot | only seen | 0 | 7 | f | f | 0.207207207 | 0.198198198 | 0.020576132 | POST |
| sine1.331 | sine | 1 | 1.33 | susy | only seen | 0 | 7 | f | f | 0.04938271 | 0.08641975 | 0.027777778 | POST |
| sine1.331 | sine | 1 | 1.33 | neomamma | 0 | 0 | 7 | f | f | 0.0420168 | 0.20168067 | 0.032840722 | POST |
| sine1.331 | sine | 1 | 1.33 | schiarita | 0 | 1 | 7 | f | f | 0.04494387 | 0.23595505 | 0.041420118 | POST |
| sine1.331 | sine | 1 | 1.33 | rosa | 0 | 0 | 7 | f | f | 0 | 0.108108108 | 0.027131783 | POST |
| sine1.331 | sine | 1 | 1.33 | sally | 0 | 0 | 7 | f | f | 0 | 0.09090909 | 0.002949853 | POST |
| sine1.331 | sine | 1 | 1.33 | scapola | 0 | 0 | 7 | f | f | 0 | 0.20833333 | 0.003787879 | POST |
| sine1.491 | sine | 1 | 1.49 | scapola | 0 | 0 | 11 | f | f | 0 | 0.20833333 | 0.003787879 | POST |
| sine1.491 | sine | 1 | 1.49 | neomamma | 0 | 0 | 11 | f | f | 0.0420168 | 0.20168067 | 0.032840722 | POST |
| sine1.491 | sine | 1 | 1.49 | schiarita | 0 | 0 | 11 | f | f | 0.04494387 | 0.23595505 | 0.041420118 | POST |
| sine1.491 | sine | 1 | 1.49 | sally | 0 | 0 | 11 | f | f | 0 | 0.09090909 | 0.002949853 | POST |
| sine1.491 | sine | 1 | 1.49 | stacy | 0 | 0 | 11 | f | f | 0.03 | 0.07 | 0.021317829 | POST |
| sine1.491 | sine | 1 | 1.49 | stella | 0 | 0 | 11 | f | f | 0 | 0.125 | 0.020080321 | POST |
| sine1.491 | sine | 1 | 1.49 | small | 0 | 0 | 11 | f | f | 0 | 0.07317073 | 0.044117647 | POST |
| sine1.491 | sine | 1 | 1.49 | secco | 0 | 0 | 11 | f | m | 0.125 | 0.08333333 | 0.123076923 | POST |
| sine1.491 | sine | 1 | 1.49 | rosa | 0 | 0 | 11 | f | f | 0 | 0.108108108 | 0.027131783 | POST |
| sine1.491 | sine | 1 | 1.49 | strega | 0 | 0 | 11 | f | f | 0 | 0.08064516 | 0.016025641 | POST |
| sine1.491 | sine | 1 | 1.49 | spiga | 0 | 0 | 11 | f | f | 0.06666667 | 0.08 | 0.018372703 | POST |
| sine1.671 | sine | 1 | 1.67 | schiarita | seen and heard | 0 | 12 | f | f | 0.04494387 | 0.23595505 | 0.041420118 | POST |
| sine1.671 | sine | 1 | 1.67 | sfasciato | seen and heard | 0 | 12 | f | m | 0.25925926 | 0.05555556 | 0.024390244 | POST |
| sine1.671 | sine | 1 | 1.67 | scapola | seen and heard | 0 | 12 | f | f | 0 | 0.20833333 | 0.003787879 | POST |
| sine1.671 | sine | 1 | 1.67 | stacy | only heard | 0 | 12 | f | f | 0.03 | 0.07 | 0.021317829 | POST |
| sine1.671 | sine | 1 | 1.67 | strega | only heard | 0 | 12 | f | f | 0 | 0.08064516 | 0.016025641 | POST |
| sine1.671 | sine | 1 | 1.67 | spot | only heard | 0 | 12 | f | f | 0.207207207 | 0.198198198 | 0.020576132 | POST |
| sine1.671 | sine | 1 | 1.67 | small | only heard | 0 | 12 | f | f | 0 | 0.07317073 | 0.044117647 | POST |
| sine1.671 | sine | 1 | 1.67 | rosa | only heard | 0 | 12 | f | f | 0 | 0.108108108 | 0.027131783 | POST |
| sine1.671 | sine | 1 | 1.67 | neomamma | only heard | 0 | 12 | f | f | 0.0420168 | 0.20168067 | 0.032840722 | POST |
| sine1.671 | sine | 1 | 1.67 | secco | only heard | 0 | 12 | f | m | 0.125 | 0.08333333 | 0.123076923 | PRE |
| sine1.671 | sine | 1 | 1.67 | spiga | only heard | 0 | 12 | f | f | 0.06666667 | 0.08 | 0.018372703 | PRE |
| sine1.671 | sine | 1 | 1.67 | sally | only heard | 0 | 12 | f | f | 0 | 0.09090909 | 0.002949853 | PRE |
| sine1.71 | sine | 1 | 1.7 | stacy | only seen | 0 | 6 | f | f | 0.03 | 0.07 | 0.021317829 | PRE |
| sine1.71 | sine | 1 | 1.7 | neomamma | only seen | 0 | 6 | f | f | 0.0420168 | 0.20168067 | 0.032840722 | PRE |
| sine1.71 | sine | 1 | 1.7 | spiga | only seen | 1 | 6 | f | f | 0.06666667 | 0.08 | 0.018372703 | PRE |
| sine1.71 | sine | 1 | 1.7 | sfasciato | only seen | 0 | 7 | f | m | 0.25925926 | 0.05555556 | 0.024390244 | PRE |
| sine1.71 | sine | 1 | 1.7 | rosa | 0 | 0 | 6 | f | f | 0 | 0.108108108 | 0.027131783 | PRE |
| sine1.71 | sine | 1 | 1.7 | spot | 0 | 0 | 6 | f | f | 0.207207207 | 0.198198198 | 0.020576132 | PRE |
| sine1.71 | sine | 1 | 1.7 | susy | 0 | 0 | 6 | f | f | 0.04938271 | 0.08641975 | 0.027777778 | PRE |
| sine1.71 | sine | 1 | 1.7 | spot | 0 | 1 | 7 | f | f | 0.207207207 | 0.198198198 | 0.020576132 | PRE |
| sine1.71 | sine | 1 | 1.7 | stacy | 0 | 0 | 7 | f | f | 0.03 | 0.07 | 0.021317829 | PRE |
| sine1.71 | sine | 1 | 1.7 | secco | 0 | 1 | 7 | f | m | 0.125 | 0.08333333 | 0.123076923 | PRE |
| sine1.71 | sine | 1 | 1.7 | susy | 0 | 0 | 7 | f | f | 0.04938271 | 0.08641975 | 0.027777778 | PRE |
| sine1.71 | sine | 1 | 1.7 | scapola | 0 | 0 | 7 | f | f | 0 | 0.20833333 | 0.003787879 | PRE |
| sine1.71 | sine | 1 | 1.7 | spiga | 0 | 0 | 7 | f | f | 0.06666667 | 0.08 | 0.018372703 | PRE |
| sine1.721 | sine | 1 | 1.72 | scapola | only seen | 0 | 5 | f | f | 0 | 0.20833333 | 0.003787879 | PRE |
| sine1.721 | sine | 1 | 1.72 | sally | only seen | 0 | 5 | f | f | 0 | 0.09090909 | 0.002949853 | PRE |
| sine1.721 | sine | 1 | 1.72 | susy | 0 | 0 | 5 | f | f | 0.04938271 | 0.08641975 | 0.027777778 | PRE |
| sine1.721 | sine | 1 | 1.72 | sorcia | 0 | 0 | 5 | f | f | 0 | 0.037037037 | 0.022727273 | PRE |
| sine1.721 | sine | 1 | 1.72 | stella | 0 | 0 | 5 | f | f | 0 | 0.125 | 0.020080321 | PRE |
| sine1.831 | sine | 1 | 1.83 | secco | only seen | 1 | 3 | f | m | 0.125 | 0.08333333 | 0.123076923 | PRE |
| sine1.831 | sine | 1 | 1.83 | rosa | 0 | 0 | 3 | f | f | 0 | 0.108108108 | 0.027131783 | PRE |
| sine1.831 | sine | 1 | 1.83 | spiga | 0 | 0 | 3 | f | f | 0.06666667 | 0.08 | 0.018372703 | PRE |
| sine2.0341 | sine | 1 | 2.034 | spot | only seen | 0 | 7 | f | f | 0.207207207 | 0.198198198 | 0.020576132 | PRE |
| sine2.0341 | sine | 1 | 2.034 | neomamma | 0 | 0 | 7 | f | f | 0.0420168 | 0.20168067 | 0.032840722 | PRE |
| sine2.0341 | sine | 1 | 2.034 | sumo | 0 | 0 | 7 | f | m | 0.11764705 | 0 | 0.203125 | PRE |
| sine2.0341 | sine | 1 | 2.034 | sciura | 0 | 0 | 7 | f | f | 0.43103448 | 0.06896551 | 0.06377858 | PRE |
| sine2.0341 | sine | 1 | 2.034 | tino | 0 | 0 | 7 | f | m | 0 | 0 | 0.111111111 | PRE |
| sine2.0341 | sine | 1 | 2.034 | strega | 0 | 0 | 7 | f | f | 0 | 0.08064516 | 0.016025641 | PRE |
| sine2.0341 | sine | 1 | 2.034 | schiarita | 0 | 0 | 7 | f | f | 0.04494387 | 0.23595505 | 0.041420118 | PRE |
| sine2.11 | sine | 1 | 2.1 | sumo | only seen | 0 | 9 | f | m | 0.11764705 | 0 | 0.203125 | PRE |
| sine2.11 | sine | 1 | 2.1 | schiarita | only seen | 0 | 9 | f | f | 0.04494387 | 0.23595505 | 0.041420118 | PRE |
| sine2.11 | sine | 1 | 2.1 | sguercia | only seen | 0 | 9 | f | f | 0 | 0.083333333 | 0 | PRE |
| sine2.11 | sine | 1 | 2.1 | strega | 0 | 0 | 9 | f | f | 0 | 0.08064516 | 0.016025641 | PRE |
| sine2.11 | sine | 1 | 2.1 | sfasciato | 0 | 0 | 9 | f | m | 0.25925926 | 0.05555556 | 0.024390244 | PRE |
| sine2.11 | sine | 1 | 2.1 | susy | 0 | 0 | 9 | f | f | 0.04938271 | 0.08641975 | 0.027777778 | PRE |
| sine2.11 | sine | 1 | 2.1 | sciura | 0 | 0 | 9 | f | f | 0.43103448 | 0.06896551 | 0.06377858 | PRE |
| sine2.11 | sine | 1 | 2.1 | secco | 0 | 0 | 9 | f | m | 0.125 | 0.08333333 | 0.123076923 | PRE |
| sine2.11 | sine | 1 | 2.1 | stacy | 0 | 0 | 9 | f | f | 0.03 | 0.07 | 0.021317829 | PRE |
| sine2.221 | sine | 1 | 2.22 | sciura | only seen | 1 | 2 | f | f | 0.43103448 | 0.06896551 | 0.06377858 | PRE |
| sine2.221 | sine | 1 | 2.22 | schiarita | 0 | 0 | 2 | f | f | 0.04494387 | 0.23595505 | 0.041420118 | PRE |
| sine2.281 | sine | 1 | 2.28 | stacy | only seen | 1 | 1 | f | f | 0.03 | 0.07 | 0.021317829 | PRE |
| sine2.651 | sine | 1 | 2.65 | stacy | 0 | 0 | 1 | f | f | 0.03 | 0.07 | 0.021317829 | PRE |
| sine2.681 | sine | 1 | 2.68 | sfasciato | 0 | 0 | 1 | f | m | 0.25925926 | 0.05555556 | 0.024390244 | PRE |
| sine2.823 | sine | 3 | 2.82 | sciura | only seen | 0 | 5 | f | f | 0.43103448 | 0.06896551 | 0.06377858 | PRE |
| sine2.823 | sine | 3 | 2.82 | schiarita | 0 | 0 | 5 | f | f | 0.04494387 | 0.23595505 | 0.041420118 | PRE |
| sine2.823 | sine | 3 | 2.82 | spot | 0 | 0 | 5 | f | f | 0.207207207 | 0.198198198 | 0.020576132 | PRE |
| sine2.823 | sine | 3 | 2.82 | susy | 0 | 0 | 5 | f | f | 0.04938271 | 0.08641975 | 0.027777778 | PRE |
| sine2.823 | sine | 3 | 2.82 | scapola | 0 | 0 | 5 | f | f | 0 | 0.20833333 | 0.003787879 | PRE |
| sine2.883 | sine | 3 | 2.88 | sciura | 0 | 0 | 3 | f | f | 0.43103448 | 0.06896551 | 0.06377858 | PRE |
| sine2.883 | sine | 3 | 2.88 | susy | 0 | 0 | 3 | f | f | 0.04938271 | 0.08641975 | 0.027777778 | PRE |
| sine2.883 | sine | 3 | 2.88 | scapola | 0 | 0 | 3 | f | f | 0 | 0.20833333 | 0.003787879 | PRE |
| small1.241 | small | 1 | 1.24 | strega | only seen | 0 | 2 | f | f | 0.21428571 | 0 | 0.016025641 | POST |
| small1.241 | small | 1 | 1.24 | rosa | 0 | 0 | 2 | f | f | 0.204545455 | 0.285714286 | 0.027131783 | POST |
| small1.9971 | small | 1 | 1.997 | sine | 0 | 0 | 9 | f | f | 0 | 0.07317073 | 0.050682261 | POST |
| small1.9971 | small | 1 | 1.997 | tino | 0 | 0 | 9 | f | m | 0 | 0 | 0.111111111 | POST |
| small1.9971 | small | 1 | 1.997 | stacy | 0 | 0 | 9 | f | f | 0.10869565 | 0.04347826 | 0.021317829 | PRE |
| small1.9971 | small | 1 | 1.997 | sumo | 0 | 0 | 9 | f | m | 0 | 0 | 0.203125 | PRE |
| small1.9971 | small | 1 | 1.997 | schiarita | 0 | 0 | 9 | f | f | 0 | 0.1372549 | 0.041420118 | PRE |
| small1.9971 | small | 1 | 1.997 | neomamma | 0 | 0 | 9 | f | f | 0.03816153 | 0.13461538 | 0.032840722 | PRE |
| small1.9971 | small | 1 | 1.997 | spiga | 0 | 0 | 9 | f | f | 0 | 0.081081081 | 0.018372703 | PRE |
| small2.291 | small | 1 | 2.29 | stacy | 0 | 0 | 6 | f | f | 0.10869565 | 0.04347826 | 0.021317829 | PRE |
| small2.291 | small | 1 | 2.29 | strega | 0 | 0 | 6 | f | f | 0.21428571 | 0 | 0.016025641 | PRE |
| small2.291 | small | 1 | 2.29 | spot | 0 | 0 | 6 | f | f | 0 | 0.12765957 | 0.020576132 | PRE |
| small2.291 | small | 1 | 2.29 | schiarita | 0 | 0 | 6 | f | f | 0 | 0.1372549 | 0.041420118 | PRE |
| small2.291 | small | 1 | 2.29 | neomamma | 0 | 0 | 6 | f | f | 0.03846153 | 0.13461538 | 0.032840722 | PRE |
| small2.291 | small | 1 | 2.29 | spiga | 0 | 0 | 6 | f | f | 0 | 0.081081081 | 0.018372703 | PRE |
| small3.11 | small | 1 | 3.1 | stacy | only seen | 0 | 1 | f | f | 0.10869565 | 0.04347826 | 0.021317829 | PRE |
| small3.451 | small | 1 | 3.45 | stacy | only seen | 0 | 4 | f | f | 0.10869565 | 0.04347826 | 0.021317829 | PRE |
| small3.451 | small | 1 | 3.45 | strega | only seen | 0 | 4 | f | f | 0.21428571 | 0 | 0.016025641 | PRE |
| small3.451 | small | 1 | 3.45 | secco | 0 | 0 | 4 | f | m | 0 | 0.20833333 | 0.123076923 | PRE |
| small3.451 | small | 1 | 3.45 | sine | 0 | 0 | 4 | f | f | 0 | 0.07317073 | 0.050682261 | PRE |
| sonia1.063 | sonia | 3 | 1.06 | severa | 0 | 0 | 3 | f | f | 0 | 0.26315789 | 0.066176471 | POST |
| sonia1.063 | sonia | 3 | 1.06 | stella | 0 | 0 | 3 | f | f | 0.13513514 | 0.10810811 | 0.020080321 | POST |
| sonia1.071 | sonia | 1 | 1.07 | stella | only seen | 1 | 3 | f | f | 0.13513514 | 0.10810811 | 0.020080321 | POST |
| sonia1.351 | sonia | 1 | 1.35 | ruga | only seen | 0 | 8 | f | f | 0.16129032 | 0.12903225 | 0.004608295 | POST |
| sonia1.351 | sonia | 1 | 1.35 | tris | only seen | 0 | 8 | f | f | 0 | 0.15909091 | 0.010449321 | POST |
| sonia1.351 | sonia | 1 | 1.35 | sorcia | 0 | 0 | 8 | f | f | 0.09375 | 0.0625 | 0.022727273 | POST |
| sonia1.351 | sonia | 1 | 1.35 | spilla | 0 | 0 | 8 | f | f | 0.44642857 | 0.19642857 | 0.043678161 | POST |
| sonia1.351 | sonia | 1 | 1.35 | severa | 0 | 0 | 8 | f | f | 0 | 0.26315789 | 0.066176471 | POST |
| sonia1.351 | sonia | 1 | 1.35 | roll | 0 | 0 | 8 | f | f | 0.1 | 0.1 | 0.036781609 | POST |
| sonia1.351 | sonia | 1 | 1.35 | stella | 0 | 0 | 8 | f | f | 0.13513514 | 0.10810811 | 0.020080321 | POST |
| sonia1.351 | sonia | 1 | 1.35 | sciura | 0 | 0 | 8 | f | f | 0.26153846 | 0.04615384 | 0.06377858 | POST |
| sonia1.381 | sonia | 1 | 1.38 | spilla | only seen | 1 | 1 | f | f | 0.44642857 | 0.19642857 | 0.043678161 | POST |
| sonia1.591 | sonia | 1 | 1.59 | osso | 0 | 0 | 3 | f | f | 0 | 0.19047619 | 0.03030303 | POST |
| sonia1.591 | sonia | 1 | 1.59 | tris | 0 | 0 | 3 | f | f | 0 | 0.15909091 | 0.010449321 | POST |
| sonia1.591 | sonia | 1 | 1.59 | stella | 0 | 0 | 3 | f | f | 0.13513514 | 0.10810811 | 0.020080321 | POST |
| sonia1.631 | sonia | 1 | 1.63 | stacy | only seen | 1 | 10 | f | f | 0.04761904 | 0.04761904 | 0.021317829 | POST |
| sonia1.631 | sonia | 1 | 1.63 | spot | only seen | 0 | 10 | f | f | 0.05882352 | 0 | 0.020576132 | POST |
| sonia1.631 | sonia | 1 | 1.63 | strega | 0 | 0 | 10 | f | f | 0 | 0.21052631 | 0.016025641 | POST |
| sonia1.631 | sonia | 1 | 1.63 | sally | 0 | 0 | 10 | f | f | 0 | 0.26315789 | 0.002949853 | POST |
| sonia1.631 | sonia | 1 | 1.63 | neomamma | 0 | 0 | 10 | f | f | 0 | 0.155555556 | 0.032840722 | POST |
| sonia1.631 | sonia | 1 | 1.63 | secco | 0 | 0 | 10 | f | m | 0 | 0.27272727 | 0.123076923 | POST |
| sonia1.631 | sonia | 1 | 1.63 | spiga | 0 | 0 | 10 | f | f | 0.07692307 | 0.03846153 | 0.018372703 | POST |
| sonia1.631 | sonia | 1 | 1.63 | rosa | 0 | 0 | 10 | f | f | 0.204545455 | 0.113636364 | 0.027131783 | POST |
| sonia1.631 | sonia | 1 | 1.63 | sfasciato | 0 | 0 | 10 | f | m | 0.07142857 | 0.21428571 | 0.024390244 | POST |
| sonia1.631 | sonia | 1 | 1.63 | small | 0 | 0 | 10 | f | f | 0 | 0.18181818 | 0.044117647 | POST |
| sonia1.891 | sonia | 1 | 1.89 | roll | only seen | 0 | 1 | f | f | 0.1 | 0.1 | 0.036781609 | POST |
| sonia1.91 | sonia | 1 | 1.9 | stella | only seen | 0 | 8 | f | f | 0.13513514 | 0.10810811 | 0.020080321 | POST |
| sonia1.91 | sonia | 1 | 1.9 | tris | 0 | 0 | 8 | f | f | 0 | 0.15909091 | 0.010449321 | POST |
| sonia1.91 | sonia | 1 | 1.9 | sciura | 0 | 0 | 8 | f | f | 0.26153846 | 0.04615384 | 0.06377858 | POST |
| sonia1.91 | sonia | 1 | 1.9 | roll | 0 | 0 | 8 | f | f | 0.1 | 0.1 | 0.036781609 | POST |
| sonia1.91 | sonia | 1 | 1.9 | severa | 0 | 0 | 8 | f | f | 0 | 0.26315789 | 0.066176471 | POST |
| sonia1.91 | sonia | 1 | 1.9 | spilla | 0 | 0 | 8 | f | f | 0.44642857 | 0.19642857 | 0.043678161 | POST |
| sonia1.91 | sonia | 1 | 1.9 | osso | 0 | 0 | 8 | f | f | 0 | 0.19047619 | 0.03030303 | POST |
| sonia1.91 | sonia | 1 | 1.9 | sguercia | 0 | 0 | 8 | f | f | 0 | 0 | 0 | POST |
| sonia1.971 | sonia | 1 | 1.97 | small | only seen | 0 | 5 | f | f | 0 | 0.18181818 | 0.044117647 | POST |
| sonia1.971 | sonia | 1 | 1.97 | stella | 0 | 0 | 3 | f | f | 0.13513514 | 0.10810811 | 0.020080321 | POST |
| sonia1.971 | sonia | 1 | 1.97 | osso | 0 | 0 | 3 | f | f | 0 | 0.19047619 | 0.03030303 | POST |
| sonia1.971 | sonia | 1 | 1.97 | tris | 0 | 0 | 3 | f | f | 0 | 0.15909091 | 0.010449321 | POST |
| sonia1.971 | sonia | 1 | 1.97 | sine | 0 | 0 | 5 | f | f | 0 | 0.12 | 0.050682261 | POST |
| sonia1.971 | sonia | 1 | 1.97 | neomamma | 0 | 0 | 5 | f | f | 0 | 0.155555556 | 0.032840722 | POST |
| sonia1.971 | sonia | 1 | 1.97 | spiga | 0 | 0 | 5 | f | f | 0.07692307 | 0.03846153 | 0.018372703 | POST |
| sonia1.971 | sonia | 1 | 1.97 | spot | 0 | 0 | 5 | f | f | 0.05882352 | 0 | 0.020576132 | POST |
| sonia1.991 | sonia | 1 | 1.99 | osso | only heard | 0 | 6 | f | f | 0 | 0.19047619 | 0.03030303 | POST |
| sonia1.991 | sonia | 1 | 1.99 | tris | only heard | 0 | 6 | f | f | 0 | 0.15909091 | 0.010449321 | POST |
| sonia1.991 | sonia | 1 | 1.99 | stella | only heard | 0 | 6 | f | f | 0.13513514 | 0.10810811 | 0.020080321 | POST |
| sonia2.111 | sonia | 1 | 2.11 | stella | only seen | 0 | 7 | f | f | 0.13513514 | 0.10810811 | 0.020080321 | POST |
| sonia2.111 | sonia | 1 | 2.11 | roll | 0 | 0 | 7 | f | f | 0.1 | 0.1 | 0.036781609 | POST |
| sonia2.111 | sonia | 1 | 2.11 | sciura | 0 | 1 | 7 | f | f | 0.26153846 | 0.04615384 | 0.06377858 | PRE |
| sonia2.111 | sonia | 1 | 2.11 | sine | 0 | 0 | 7 | f | f | 0 | 0.12 | 0.050682261 | PRE |
| sonia2.111 | sonia | 1 | 2.11 | sfasciato | 0 | 0 | 7 | f | m | 0.01742857 | 0.21428571 | 0.024390244 | PRE |
| sonia2.111 | sonia | 1 | 2.11 | secco | 0 | 0 | 7 | f | m | 0 | 0.27272727 | 0.123076923 | PRE |
| sonia2.111 | sonia | 1 | 2.11 | ruga | 0 | 0 | 7 | f | f | 0.16129032 | 0.12903225 | 0.004608295 | PRE |
| sonia2.131 | sonia | 1 | 2.13 | severa | only heard | 0 | 5 | f | f | 0 | 0.26315789 | 0.066176471 | PRE |
| sonia2.131 | sonia | 1 | 2.13 | tris | only heard | 0 | 5 | f | f | 0 | 0.15909091 | 0.010449321 | PRE |
| sonia2.131 | sonia | 1 | 2.13 | osso | only heard | 0 | 5 | f | f | 0 | 0.19047619 | 0.03030303 | PRE |
| sonia2.131 | sonia | 1 | 2.13 | roll | only heard | 0 | 5 | f | f | 0.1 | 0.1 | 0.036781609 | PRE |
| sonia2.131 | sonia | 1 | 2.13 | spilla | only heard | 0 | 5 | f | f | 0.44642857 | 0.19642857 | 0.043678161 | PRE |
| sonia2.473 | sonia | 3 | 2.47 | osso | 0 | 0 | 3 | f | f | 0 | 0.19047619 | 0.03030303 | PRE |
| sonia2.473 | sonia | 3 | 2.47 | tris | 0 | 0 | 3 | f | f | 0 | 0.15909091 | 0.010449321 | PRE |
| sonia2.473 | sonia | 3 | 2.47 | stella | 0 | 0 | 3 | f | f | 0.13513514 | 0.10810811 | 0.020080321 | PRE |
| sonia2.512 | sonia | 2 | 2.51 | roll | 0 | 1 | 1 | f | f | 0.1 | 0.1 | 0.036781609 | PRE |
| sonia2.71 | sonia | 1 | 2.7 | ruga | only seen | 0 | 3 | f | f | 0.16129032 | 0.12903225 | 0.004608295 | PRE |
| sonia2.71 | sonia | 1 | 2.7 | roll | 0 | 0 | 3 | f | f | 0.1 | 0.1 | 0.036781609 | PRE |
| sonia2.71 | sonia | 1 | 2.7 | neomamma | 0 | 0 | 3 | f | f | 0 | 0.155555556 | 0.032840722 | PRE |
| sonia3.073 | sonia | 3 | 3.07 | roll | 0 | 0 | 4 | f | f | 0.1 | 0.1 | 0.036781609 | PRE |
| sonia3.073 | sonia | 3 | 3.07 | osso | 0 | 0 | 4 | f | f | 0 | 0.19047619 | 0.03030303 | PRE |
| sonia3.073 | sonia | 3 | 3.07 | tris | 0 | 0 | 4 | f | f | 0 | 0.15909091 | 0.010449321 | PRE |
| sonia3.073 | sonia | 3 | 3.07 | spilla | 0 | 1 | 4 | f | f | 0.44642857 | 0.19642857 | 0.043678161 | PRE |
| sonia3.0723 | sonia | 3 | 3.072 | severa | only seen | 0 | 4 | f | f | 0 | 0.26315789 | 0.066176471 | PRE |
| sonia3.0723 | sonia | 3 | 3.072 | spilla | 0 | 0 | 4 | f | f | 0.44642857 | 0.19642857 | 0.043678161 | PRE |
| sonia3.0723 | sonia | 3 | 3.072 | tris | 0 | 0 | 4 | f | f | 0 | 0.15909091 | 0.010449321 | PRE |
| sonia3.0723 | sonia | 3 | 3.072 | roll | 0 | 0 | 4 | f | f | 0.1 | 0.1 | 0.036781609 | PRE |
| sonia3.231 | sonia | 1 | 3.23 | strega | only heard | 0 | 16 | f | f | 0 | 0.21052631 | 0.016025641 | PRE |
| sonia3.231 | sonia | 1 | 3.23 | rosa | only heard | 0 | 16 | f | f | 0.204545455 | 0.285714286 | 0.027131783 | PRE |
| sonia3.231 | sonia | 1 | 3.23 | sfasciato | only heard | 0 | 16 | f | m | 0.01742857 | 0.21428571 | 0.024390244 | PRE |
| sonia3.231 | sonia | 1 | 3.23 | scapola | only heard | 0 | 16 | f | f | 0 | 0.133333333 | 0.003787879 | PRE |
| sonia3.231 | sonia | 1 | 3.23 | sally | only heard | 0 | 16 | f | f | 0 | 0.23615789 | 0.002949853 | PRE |
| sonia3.231 | sonia | 1 | 3.23 | neomamma | only heard | 0 | 16 | f | f | 0 | 0.155555556 | 0.032840722 | PRE |
| sonia3.231 | sonia | 1 | 3.23 | schiarita | only heard | 0 | 16 | f | f | 0.10714285 | 0.14285714 | 0.041420118 | PRE |
| sonia3.231 | sonia | 1 | 3.23 | severa | only heard | 0 | 16 | f | f | 0 | 0.26315789 | 0.066176471 | PRE |
| sonia3.231 | sonia | 1 | 3.23 | stacy | only heard | 0 | 16 | f | f | 0.04761904 | 0.04761904 | 0.021317829 | PRE |
| sonia3.231 | sonia | 1 | 3.23 | spiga | only heard | 0 | 16 | f | f | 0.07692307 | 0.03846153 | 0.018372703 | PRE |
| sonia3.231 | sonia | 1 | 3.23 | sorcia | only heard | 0 | 16 | f | f | 0.09375 | 0.0625 | 0.022727273 | PRE |
| sonia3.231 | sonia | 1 | 3.23 | sine | only heard | 0 | 16 | f | f | 0 | 0.12 | 0.050682261 | PRE |
| sonia3.231 | sonia | 1 | 3.23 | sguercia | only heard | 0 | 16 | f | f | 0 | 0 | 0 | PRE |
| sonia3.231 | sonia | 1 | 3.23 | secco | only heard | 0 | 16 | f | m | 0 | 0.27272727 | 0.123076923 | PRE |
| sonia3.231 | sonia | 1 | 3.23 | stella | only heard | 0 | 16 | f | f | 0.13513514 | 0.10810811 | 0.020080321 | PRE |
| sonia3.231 | sonia | 1 | 3.23 | spiga | only heard | 1 | 16 | f | f | 0.07692307 | 0.03846153 | 0.018372703 | PRE |
| sonia3.381 | sonia | 1 | 3.38 | sciura | 0 | 0 | 4 | f | f | 0.26153846 | 0.04615384 | 0.06377858 | PRE |
| sonia3.381 | sonia | 1 | 3.38 | neomamma | 0 | 0 | 4 | f | f | 0 | 0.155555556 | 0.032840722 | PRE |
| sonia3.381 | sonia | 1 | 3.38 | stella | 0 | 0 | 4 | f | f | 0.13513514 | 0.10810811 | 0.020080321 | PRE |
| sonia3.381 | sonia | 1 | 3.38 | roll | 0 | 0 | 4 | f | f | 0.1 | 0.1 | 0.036781609 | PRE |
| sorcia1.1911 | sorcia | 1 | 1.191 | strega | seen and heard | 0 | 9 | f | f | 0.321428571 | 0.0357143 | 0.016025641 | POST |
| sorcia1.1911 | sorcia | 1 | 1.191 | spot | only heard | 0 | 9 | f | f | 0 | 0.15384615 | 0.020576132 | POST |
| sorcia1.1911 | sorcia | 1 | 1.191 | tino | only heard | 0 | 9 | f | m | 0 | 0 | 0.111111111 | POST |
| sorcia1.1911 | sorcia | 1 | 1.191 | sine | only heard | 0 | 9 | f | f | 0 | 0.037037037 | 0.050682261 | POST |
| sorcia1.1911 | sorcia | 1 | 1.191 | neomamma | only heard | 0 | 9 | f | f | 0 | 0 | 0.032840722 | POST |
| sorcia1.1911 | sorcia | 1 | 1.191 | sciura | only heard | 0 | 9 | f | f | 0 | 0.02272727 | 0.06377858 | POST |
| sorcia1.1911 | sorcia | 1 | 1.191 | sumo | only heard | 1 | 9 | f | m | 0 | 0.05 | 0.203125 | POST |
| sorcia1.1911 | sorcia | 1 | 1.191 | spilla | only heard | 0 | 9 | f | f | 0.03225806 | 0 | 0.043678161 | POST |
| sorcia1.1911 | sorcia | 1 | 1.191 | schiarita | only heard | 0 | 9 | f | f | 0 | 0.027027027 | 0.041420118 | POST |
| sorcia1.1931 | sorcia | 1 | 1.193 | strega | only seen | 0 | 8 | f | f | 0.321428571 | 0.0357143 | 0.016025641 | POST |
| sorcia1.1931 | sorcia | 1 | 1.193 | tino | 0 | 0 | 8 | f | m | 0 | 0 | 0.111111111 | PRE |
| sorcia1.1931 | sorcia | 1 | 1.193 | neomamma | 0 | 0 | 8 | f | f | 0 | 0 | 0.032840722 | PRE |
| sorcia1.1931 | sorcia | 1 | 1.193 | sumo | 0 | 0 | 8 | f | m | 0 | 0.05 | 0.203125 | PRE |
| sorcia1.1931 | sorcia | 1 | 1.193 | spilla | 0 | 0 | 8 | f | f | 0.03225806 | 0 | 0.043678161 | PRE |
| sorcia1.1931 | sorcia | 1 | 1.193 | schiarita | 0 | 0 | 8 | f | f | 0 | 0.027027027 | 0.041420118 | PRE |
| sorcia1.1931 | sorcia | 1 | 1.193 | sine | 0 | 0 | 8 | f | f | 0 | 0.037037037 | 0.050682261 | PRE |
| sorcia1.1931 | sorcia | 1 | 1.193 | sciura | 0 | 0 | 8 | f | f | 0 | 0.02272727 | 0.06377858 | PRE |
| sorcia1.421 | sorcia | 1 | 1.42 | spilla | seen and heard | 1 | 1 | f | f | 0.03225806 | 0 | 0.043678161 | PRE |
| sorcia1.661 | sorcia | 1 | 1.66 | sfasciato | seen and heard | 0 | 14 | f | m | 0 | 0.07692307 | 0.024390244 | PRE |
| sorcia1.661 | sorcia | 1 | 1.66 | sguercia | only heard | 0 | 14 | f | f | 0.03030303 | 0.333333333 | 0 | PRE |
| sorcia1.661 | sorcia | 1 | 1.66 | sonia | only heard | 1 | 14 | f | f | 0.09375 | 0.0625 | 0.041666667 | PRE |
| sorcia1.661 | sorcia | 1 | 1.66 | spilla | only heard | 1 | 14 | f | f | 0.03225806 | 0 | 0.043678161 | PRE |
| sorcia1.661 | sorcia | 1 | 1.66 | rosa | only heard | 0 | 14 | f | f | 0 | 0.04347826 | 0.027131783 | PRE |
| sorcia1.661 | sorcia | 1 | 1.66 | strega | only heard | 0 | 14 | f | f | 0.204545455 | 0.285714286 | 0.016025641 | PRE |
| sorcia1.661 | sorcia | 1 | 1.66 | sally | only heard | 0 | 14 | f | f | 0 | 0.05263157 | 0.002949853 | PRE |
| sorcia1.661 | sorcia | 1 | 1.66 | schiarita | only heard | 0 | 14 | f | f | 0 | 0.027027027 | 0.041420118 | PRE |
| sorcia1.661 | sorcia | 1 | 1.66 | neomamma | only heard | 0 | 14 | f | f | 0 | 0 | 0.032840722 | PRE |
| sorcia1.661 | sorcia | 1 | 1.66 | scapola | only heard | 0 | 14 | f | f | 0 | 0.1 | 0.003787879 | PRE |
| sorcia1.661 | sorcia | 1 | 1.66 | sine | only heard | 0 | 14 | f | f | 0 | 0.037037037 | 0.050682261 | PRE |
| sorcia1.661 | sorcia | 1 | 1.66 | spot | only heard | 0 | 14 | f | f | 0 | 0.15384615 | 0.020576132 | PRE |
| sorcia1.661 | sorcia | 1 | 1.66 | stella | only heard | 0 | 14 | f | f | 0.136363636 | 0 | 0.020080321 | PRE |
| sorcia1.661 | sorcia | 1 | 1.66 | stacy | only heard | 0 | 14 | f | f | 0.05555556 | 0.25 | 0.021317829 | PRE |
| sorcia1.7171 | sorcia | 1 | 1.717 | strega | only seen | 0 | 9 | f | f | 0.321428571 | 0.0357143 | 0.016025641 | PRE |
| sorcia1.7171 | sorcia | 1 | 1.717 | spot | 0 | 0 | 9 | f | f | 0 | 0.15384615 | 0.020576132 | PRE |
| sorcia1.7171 | sorcia | 1 | 1.717 | tino | 0 | 0 | 9 | f | m | 0 | 0 | 0.111111111 | PRE |
| sorcia1.7171 | sorcia | 1 | 1.717 | sine | 0 | 0 | 9 | f | f | 0 | 0.037037037 | 0.050682261 | PRE |
| sorcia1.7171 | sorcia | 1 | 1.717 | neomamma | 0 | 0 | 9 | f | f | 0 | 0 | 0.032840722 | PRE |
| sorcia1.7171 | sorcia | 1 | 1.717 | sciura | 0 | 0 | 9 | f | f | 0 | 0.02272727 | 0.06377858 | PRE |
| sorcia1.7171 | sorcia | 1 | 1.717 | sumo | 0 | 0 | 9 | f | m | 0 | 0.05 | 0.203125 | PRE |
| sorcia1.7171 | sorcia | 1 | 1.717 | spilla | 0 | 0 | 9 | f | f | 0.03225806 | 0 | 0.043678161 | PRE |
| sorcia1.7171 | sorcia | 1 | 1.717 | schiarita | 0 | 0 | 9 | f | f | 0 | 0.027027027 | 0.041420118 | PRE |
| sorcia2.1021 | sorcia | 1 | 2.102 | sine | 0 | 0 | 10 | f | f | 0 | 0.037037037 | 0.050682261 | PRE |
| sorcia2.1021 | sorcia | 1 | 2.102 | tino | 0 | 0 | 10 | f | m | 0 | 0 | 0.111111111 | PRE |
| sorcia2.1021 | sorcia | 1 | 2.102 | stacy | 0 | 0 | 10 | f | f | 0.05555556 | 0.25 | 0.021317829 | PRE |
| sorcia2.1021 | sorcia | 1 | 2.102 | small | 0 | 0 | 10 | f | f | 0 | 0.14285714 | 0.044117647 | PRE |
| sorcia2.1021 | sorcia | 1 | 2.102 | schiarita | 0 | 0 | 10 | f | f | 0 | 0.027027027 | 0.041420118 | PRE |
| sorcia2.1021 | sorcia | 1 | 2.102 | neomamma | 0 | 0 | 10 | f | f | 0 | 0 | 0.032840722 | PRE |
| sorcia2.1021 | sorcia | 1 | 2.102 | strega | 0 | 0 | 10 | f | f | 0.152777778 | 0.191489362 | 0.016025641 | PRE |
| sorcia2.1021 | sorcia | 1 | 2.102 | susy | 0 | 0 | 10 | f | f | 0 | 0 | 0.027777778 | PRE |
| spiga1.951 | spiga | 1 | 1.95 | scapola | only seen | 1 | 7 | f | f | 0 | 0.14285714 | 0.003787879 | POST |
| spiga1.951 | spiga | 1 | 1.95 | susy | 0 | 0 | 7 | f | f | 0 | 0.10909091 | 0.027777778 | POST |
| spiga1.951 | spiga | 1 | 1.95 | spot | 0 | 0 | 7 | f | f | 0.11688312 | 0.16883116 | 0.020576132 | POST |
| spiga1.951 | spiga | 1 | 1.95 | stacy | 0 | 0 | 7 | f | f | 0.063291139 | 0.16455696 | 0.021317829 | POST |
| spiga1.951 | spiga | 1 | 1.95 | secco | 0 | 0 | 7 | f | m | 0 | 0.08108108 | 0.123076923 | POST |
| spiga1.951 | spiga | 1 | 1.95 | rosa | 0 | 0 | 7 | f | f | 0 | 0.08510638 | 0.027131783 | POST |
| spiga1.951 | spiga | 1 | 1.95 | neomamma | 0 | 0 | 7 | f | f | 0.14150943 | 0.16981132 | 0.032840722 | POST |
| spiga2.281 | spiga | 1 | 2.28 | scapola | 0 | 0 | 6 | f | f | 0 | 0.14285714 | 0.003787879 | POST |
| spiga2.281 | spiga | 1 | 2.28 | neomamma | 0 | 0 | 6 | f | f | 0.14150943 | 0.16981132 | 0.032840722 | POST |
| spiga2.281 | spiga | 1 | 2.28 | sorcia | 0 | 0 | 6 | f | f | 0.1 | 0.1 | 0.022727273 | POST |
| spiga2.281 | spiga | 1 | 2.28 | sciura | 0 | 0 | 6 | f | f | 0 | 0.14814814 | 0.06377858 | POST |
| spiga2.281 | spiga | 1 | 2.28 | spilla | 0 | 1 | 6 | f | f | 0 | 0 | 0.043678161 | POST |
| spiga2.281 | spiga | 1 | 2.28 | stella | 0 | 0 | 6 | f | f | 0.08 | 0.16 | 0.020080321 | POST |
| spiga2.421 | spiga | 1 | 2.42 | scapola | only seen | 0 | 8 | f | f | 0 | 0.14285714 | 0.003787879 | POST |
| spiga2.421 | spiga | 1 | 2.42 | neomamma | only seen | 0 | 8 | f | f | 0.14150943 | 0.16981132 | 0.032840722 | POST |
| spiga2.421 | spiga | 1 | 2.42 | sciura | 0 | 0 | 8 | f | f | 0 | 0.14814814 | 0.06377858 | POST |
| spiga2.421 | spiga | 1 | 2.42 | stella | 0 | 0 | 8 | f | f | 0.08 | 0.16 | 0.020080321 | POST |
| spiga2.421 | spiga | 1 | 2.42 | roll | 0 | 0 | 8 | f | f | 0 | 0.17647058 | 0.036781609 | POST |
| spiga2.421 | spiga | 1 | 2.42 | rosa | 0 | 0 | 8 | f | f | 0 | 0.08510638 | 0.027131783 | POST |
| spiga2.421 | spiga | 1 | 2.42 | spot | 0 | 0 | 8 | f | f | 0.11688312 | 0.16883116 | 0.020576132 | POST |
| spiga2.421 | spiga | 1 | 2.42 | ruga | 0 | 0 | 8 | f | f | 0 | 0.111111111 | 0.004608295 | POST |
| spiga3.141 | spiga | 1 | 3.14 | stella | 0 | 0 | 5 | f | f | 0.08 | 0.16 | 0.020080321 | POST |
| spiga3.141 | spiga | 1 | 3.14 | sally | 0 | 0 | 5 | f | f | 0 | 0.21052631 | 0.002949853 | POST |
| spiga3.141 | spiga | 1 | 3.14 | schiarita | 0 | 0 | 5 | f | f | 0.08641975 | 0.13580246 | 0.041420118 | POST |
| spiga3.141 | spiga | 1 | 3.14 | spilla | 0 | 0 | 5 | f | f | 0 | 0 | 0.043678161 | POST |
| spiga3.141 | spiga | 1 | 3.14 | sorcia | 0 | 0 | 5 | f | f | 0.1 | 0.1 | 0.022727273 | PRE |
| spiga4.313 | spiga | 3 | 4.31 | strega | 0 | 0 | 16 | f | f | 0.02083333 | 0.0625 | 0.016025641 | PRE |
| spiga4.313 | spiga | 3 | 4.31 | rosa | 0 | 0 | 16 | f | f | 0 | 0.08510638 | 0.027131783 | PRE |
| spiga4.313 | spiga | 3 | 4.31 | sonia | 0 | 0 | 16 | f | f | 0.07692307 | 0.03846153 | 0.041666667 | PRE |
| spiga4.313 | spiga | 3 | 4.31 | spilla | 0 | 0 | 16 | f | f | 0 | 0 | 0.043678161 | PRE |
| spiga4.313 | spiga | 3 | 4.31 | scapola | 0 | 0 | 16 | f | f | 0 | 0.14285714 | 0.003787879 | PRE |
| spiga4.313 | spiga | 3 | 4.31 | sally | 0 | 0 | 16 | f | f | 0 | 0.21052631 | 0.002949853 | PRE |
| spiga4.313 | spiga | 3 | 4.31 | neomamma | 0 | 0 | 16 | f | f | 0.14150943 | 0.16981132 | 0.032840722 | PRE |
| spiga4.313 | spiga | 3 | 4.31 | schiarita | 0 | 0 | 16 | f | f | 0.08641975 | 0.13580246 | 0.041420118 | PRE |
| spiga4.313 | spiga | 3 | 4.31 | severa | 0 | 0 | 16 | f | f | 0 | 0.23529411 | 0.066176471 | PRE |
| spiga4.313 | spiga | 3 | 4.31 | stacy | 0 | 0 | 16 | f | f | 0.063291139 | 0.16455696 | 0.021317829 | PRE |
| spiga4.313 | spiga | 3 | 4.31 | sorcia | 0 | 0 | 16 | f | f | 0.1 | 0.1 | 0.022727273 | PRE |
| spiga4.313 | spiga | 3 | 4.31 | sine | 0 | 0 | 16 | f | f | 0.06666667 | 0.08 | 0.050682261 | PRE |
| spiga4.313 | spiga | 3 | 4.31 | sguercia | 0 | 0 | 16 | f | f | 0 | 0 | 0 | PRE |
| spiga4.313 | spiga | 3 | 4.31 | secco | 0 | 0 | 16 | f | m | 0 | 0.08108108 | 0.123076923 | PRE |
| spiga4.313 | spiga | 3 | 4.31 | stella | 0 | 0 | 16 | f | f | 0.08 | 0.16 | 0.020080321 | PRE |
| spiga4.313 | spiga | 3 | 4.31 | small | 0 | 0 | 16 | f | f | 0 | 0.081081081 | 0.044117647 | PRE |
| spiga5.591 | spiga | 1 | 5.59 | secco | only seen | 0 | 7 | f | m | 0 | 0.08108108 | 0.123076923 | PRE |
| spiga5.591 | spiga | 1 | 5.59 | sine | 0 | 0 | 7 | f | f | 0.06666667 | 0.08 | 0.050682261 | PRE |
| spiga5.591 | spiga | 1 | 5.59 | rosa | 0 | 0 | 7 | f | f | 0 | 0.08510638 | 0.027131783 | PRE |
| spiga5.591 | spiga | 1 | 5.59 | stacy | 0 | 0 | 7 | f | f | 0.063291139 | 0.16455696 | 0.021317829 | PRE |
| spiga5.591 | spiga | 1 | 5.59 | susy | 0 | 0 | 7 | f | f | 0 | 0.10909091 | 0.027777778 | PRE |
| spiga5.591 | spiga | 1 | 5.59 | neomamma | 0 | 0 | 7 | f | f | 0.14150943 | 0.16981132 | 0.032840722 | PRE |
| spiga5.591 | spiga | 1 | 5.59 | spot | 0 | 0 | 7 | f | f | 0.11688312 | 0.16883116 | 0.020576132 | PRE |
| spilla1.21 | spilla | 1 | 1.2 | severa | only seen | 0 | 3 | f | f | 0.15463917 | 0.16494845 | 0.066176471 | POST |
| spilla1.21 | spilla | 1 | 1.2 | osso | only seen | 0 | 3 | f | f | 0.08064516 | 0.27419354 | 0.03030303 | POST |
| spilla1.21 | spilla | 1 | 1.2 | sonia | 0 | 0 | 3 | f | f | 0.44642857 | 0.19642857 | 0.041666667 | POST |
| spilla1.231 | spilla | 1 | 1.23 | severa | only seen | 1 | 1 | f | f | 0.15463917 | 0.16494845 | 0.066176471 | POST |
| spilla1.391 | spilla | 1 | 1.39 | sonia | 0 | 0 | 1 | f | f | 0.44642857 | 0.19642857 | 0.041666667 | POST |
| spilla1.5* | spilla | * | 1.5 | strega | 0 | 0 | 16 | f | f | 0 | 0.11111111 | 0.016025641 | POST |
| spilla1.5* | spilla | * | 1.5 | rosa | 0 | 0 | 16 | f | f | 0 | 0.16666667 | 0.027131783 | POST |
| spilla1.5* | spilla | * | 1.5 | sfasciato | 0 | 0 | 16 | f | m | 0 | 0 | 0.024390244 | POST |
| spilla1.5* | spilla | * | 1.5 | scapola | 0 | 0 | 16 | f | f | 0 | 0 | 0.003787879 | POST |
| spilla1.5* | spilla | * | 1.5 | sally | 0 | 0 | 16 | f | f | 0 | 0.07142857 | 0.002949853 | POST |
| spilla1.5* | spilla | * | 1.5 | neomamma | 0 | 0 | 16 | f | f | 0 | 0.05555556 | 0.032840722 | POST |
| spilla1.5* | spilla | * | 1.5 | schiarita | 0 | 0 | 16 | f | f | 0 | 0.17857142 | 0.041420118 | POST |
| spilla1.5* | spilla | * | 1.5 | severa | 0 | 0 | 16 | f | f | 0.15463917 | 0.16494845 | 0.066176471 | POST |
| spilla1.5* | spilla | * | 1.5 | stacy | 0 | 0 | 16 | f | f | 0 | 0 | 0.021317829 | POST |
| spilla1.5* | spilla | * | 1.5 | spiga | 0 | 0 | 16 | f | f | 0 | 0 | 0.018372703 | POST |
| spilla1.5* | spilla | * | 1.5 | sorcia | 0 | 0 | 16 | f | f | 0.03225806 | 0 | 0.022727273 | POST |
| spilla1.5* | spilla | * | 1.5 | sine | 0 | 0 | 16 | f | f | 0.07692307 | 0.07692307 | 0.050682261 | POST |
| spilla1.5* | spilla | * | 1.5 | sguercia | 0 | 0 | 16 | f | f | 0.03448275 | 0.03448275 | 0 | POST |
| spilla1.5* | spilla | * | 1.5 | secco | 0 | 0 | 16 | f | m | 0 | 0 | 0.123076923 | POST |
| spilla1.5* | spilla | * | 1.5 | stella | 0 | 0 | 16 | f | f | 0.03448276 | 0 | 0.020080321 | POST |
| spilla1.5* | spilla | * | 1.5 | spiga | 0 | 0 | 16 | f | f | 0 | 0 | 0.018372703 | POST |
| spilla1.641 | spilla | 1 | 1.64 | severa | seen and heard | 1 | 3 | f | f | 0.15463917 | 0.16494845 | 0.066176471 | POST |
| spilla1.641 | spilla | 1 | 1.64 | sorcia | only heard | 0 | 3 | f | f | 0.03225806 | 0 | 0.022727273 | POST |
| spilla1.641 | spilla | 1 | 1.64 | sguercia | only heard | 0 | 3 | f | f | 0.03448275 | 0.03448275 | 0 | POST |
| spilla1.7* | spilla | * | 1.7 | osso | 0 | 0 | 3 | f | f | 0.08064516 | 0.27419354 | 0.03030303 | POST |
| spilla1.7* | spilla | * | 1.7 | tris | 0 | 0 | 3 | f | f | 0.12628558 | 0.25316455 | 0.010449321 | POST |
| spilla1.783 | spilla | 3 | 1.78 | susy | only seen | 0 | 8 | f | f | 0 | 0.33333333 | 0.027777778 | POST |
| spilla1.783 | spilla | 3 | 1.78 | neomamma | 0 | 0 | 8 | f | f | 0 | 0.05555556 | 0.032840722 | POST |
| spilla1.783 | spilla | 3 | 1.78 | scapola | 0 | 0 | 8 | f | f | 0 | 0 | 0.003787879 | POST |
| spilla1.783 | spilla | 3 | 1.78 | spiga | 0 | 0 | 8 | f | f | 0 | 0 | 0.018372703 | POST |
| spilla1.783 | spilla | 3 | 1.78 | spot | 0 | 0 | 8 | f | f | 0.25 | 0 | 0.020576132 | POST |
| spilla1.783 | spilla | 3 | 1.78 | sine | 0 | 0 | 8 | f | f | 0.07692307 | 0.07692307 | 0.050682261 | POST |
| spilla1.783 | spilla | 3 | 1.78 | schiarita | 0 | 0 | 8 | f | f | 0 | 0.17857142 | 0.041420118 | POST |
| spilla1.783 | spilla | 3 | 1.78 | rosa | 0 | 0 | 8 | f | f | 0 | 0.16666667 | 0.027131783 | POST |
| spilla1.99* | spilla | * | 1.99 | osso | 0 | 0 | 6 | f | f | 0.08064516 | 0.27419354 | 0.03030303 | POST |
| spilla1.99* | spilla | * | 1.99 | tris | 0 | 0 | 6 | f | f | 0.12628558 | 0.25316455 | 0.010449321 | POST |
| spilla1.99* | spilla | * | 1.99 | stella | 0 | 0 | 6 | f | f | 0.03448276 | 0 | 0.020080321 | POST |
| spilla2.061 | spilla | 1 | 2.06 | sonia | only seen | 0 | 4 | f | f | 0.44642857 | 0.19642857 | 0.041666667 | POST |
| spilla2.061 | spilla | 1 | 2.06 | severa | 0 | 0 | 4 | f | f | 0.15463917 | 0.16494845 | 0.066176471 | POST |
| spilla2.061 | spilla | 1 | 2.06 | tris | 0 | 0 | 4 | f | f | 0.12628558 | 0.25316455 | 0.010449321 | POST |
| spilla2.061 | spilla | 1 | 2.06 | osso | 0 | 0 | 4 | f | f | 0.08064516 | 0.27419354 | 0.03030303 | POST |
| spilla2.171 | spilla | 1 | 2.17 | sonia | 0 | 0 | 1 | f | f | 0.44642857 | 0.19642857 | 0.041666667 | POST |
| spilla2.261 | spilla | 1 | 2.26 | roll | 0 | 0 | 4 | f | f | 0 | 0.11475409 | 0.036781609 | POST |
| spilla2.261 | spilla | 1 | 2.26 | sonia | 0 | 0 | 4 | f | f | 0.44642857 | 0.19642857 | 0.041666667 | POST |
| spilla2.261 | spilla | 1 | 2.26 | osso | 0 | 0 | 4 | f | f | 0.08064516 | 0.27419354 | 0.03030303 | POST |
| spilla2.261 | spilla | 1 | 2.26 | tris | 0 | 0 | 4 | f | f | 0.12628558 | 0.25316455 | 0.010449321 | POST |
| spilla2.391 | spilla | 1 | 2.39 | sonia | only seen | 0 | 4 | f | f | 0.44642857 | 0.19642857 | 0.041666667 | POST |
| spilla2.391 | spilla | 1 | 2.39 | tris | only seen | 0 | 4 | f | f | 0.12628558 | 0.25316455 | 0.010449321 | POST |
| spilla2.391 | spilla | 1 | 2.39 | severa | 0 | 0 | 4 | f | f | 0.15463917 | 0.16494845 | 0.066176471 | POST |
| spilla2.391 | spilla | 1 | 2.39 | osso | 0 | 0 | 4 | f | f | 0.08064516 | 0.27419354 | 0.03030303 | POST |
| spilla2.411 | spilla | 1 | 2.41 | sonia | only seen | 0 | 1 | f | f | 0.44642857 | 0.19642857 | 0.041666667 | POST |
| spilla2.413 | spilla | 3 | 2.41 | severa | 0 | 0 | 1 | f | f | 0.15463917 | 0.16494845 | 0.066176471 | PRE |
| spilla2.411 | spilla | 1 | 2.41 | osso | 0 | 1 | 1 | f | f | 0.08064516 | 0.27419354 | 0.03030303 | PRE |
| spilla2.411 | spilla | 1 | 2.41 | severa | 0 | 0 | 1 | f | f | 0.15463917 | 0.16494845 | 0.066176471 | PRE |
| spilla2.411 | spilla | 1 | 2.41 | tris | 0 | 0 | 1 | f | f | 0.12628558 | 0.25316455 | 0.010449321 | PRE |
| spilla2.47* | spilla | * | 2.47 | osso | 0 | 0 | 3 | f | f | 0.08064516 | 0.27419354 | 0.03030303 | PRE |
| spilla2.47* | spilla | * | 2.47 | tris | 0 | 0 | 3 | f | f | 0.12628558 | 0.25316455 | 0.010449321 | PRE |
| spilla2.47* | spilla | * | 2.47 | stella | 0 | 0 | 3 | f | f | 0.03448276 | 0 | 0.020080321 | PRE |
| spilla2.521 | spilla | 1 | 2.52 | sciura | seen and heard | 0 | 3 | f | f | 0.21875 | 0.109375 | 0.06377858 | PRE |
| spilla2.521 | spilla | 1 | 2.52 | tris | only heard | 0 | 3 | f | f | 0.12628558 | 0.25316455 | 0.010449321 | PRE |
| spilla2.521 | spilla | 1 | 2.52 | severa | only heard | 0 | 3 | f | f | 0.15463917 | 0.16494845 | 0.066176471 | PRE |
| spilla2.543 | spilla | 3 | 2.54 | scapola | only seen | 1 | 7 | f | f | 0 | 0 | 0.003787879 | PRE |
| spilla2.543 | spilla | 3 | 2.54 | spot | only seen | 0 | 7 | f | f | 0.25 | 0 | 0.020576132 | PRE |
| spilla2.543 | spilla | 3 | 2.54 | susy | only seen | 0 | 7 | f | f | 0 | 0.33333333 | 0.027777778 | PRE |
| spilla2.543 | spilla | 3 | 2.54 | schiarita | only seen | 0 | 7 | f | f | 0 | 0.17857142 | 0.041420118 | PRE |
| spilla2.543 | spilla | 3 | 2.54 | neomamma | 0 | 0 | 7 | f | f | 0 | 0.05555556 | 0.032840722 | PRE |
| spilla2.543 | spilla | 3 | 2.54 | rosa | 0 | 0 | 7 | f | f | 0 | 0.16666667 | 0.027131783 | PRE |
| spilla2.543 | spilla | 3 | 2.54 | spiga | 0 | 0 | 7 | f | f | 0 | 0 | 0.018372703 | PRE |
| spilla2.653 | spilla | 3 | 2.65 | sonia | only seen | 0 | 2 | f | f | 0.44642857 | 0.19642857 | 0.041666667 | PRE |
| spilla2.653 | spilla | 3 | 2.65 | severa | only seen | 1 | 2 | f | f | 0.15463917 | 0.16494845 | 0.066176471 | PRE |
| spilla2.823 | spilla | 3 | 2.82 | scapola | only heard | 1 | 5 | f | f | 0 | 0 | 0.003787879 | PRE |
| spilla2.823 | spilla | 3 | 2.82 | stella | only heard | 0 | 5 | f | f | 0.03448276 | 0 | 0.020080321 | PRE |
| spilla2.823 | spilla | 3 | 2.82 | sally | only heard | 0 | 5 | f | f | 0 | 0.07142857 | 0.002949853 | PRE |
| spilla2.823 | spilla | 3 | 2.82 | schiarita | only heard | 1 | 5 | f | f | 0 | 0.17857142 | 0.041420118 | PRE |
| spilla2.823 | spilla | 3 | 2.82 | spot | only heard | 1 | 5 | f | f | 0.25 | 0 | 0.020576132 | PRE |
| spilla3.071 | spilla | 1 | 3.07 | sonia | only seen | 0 | 3 | f | f | 0.44642857 | 0.19642857 | 0.041666667 | PRE |
| spilla3.071 | spilla | 1 | 3.07 | severa | only seen | 0 | 3 | f | f | 0.15463917 | 0.16494845 | 0.066176471 | PRE |
| spilla3.071 | spilla | 1 | 3.07 | osso | only seen | 0 | 3 | f | f | 0.08064516 | 0.27419354 | 0.03030303 | PRE |
| spilla3.38* | spilla | * | 3.38 | tris | 0 | 0 | 4 | f | f | 0.12628558 | 0.25316455 | 0.010449321 | PRE |
| spilla3.38* | spilla | * | 3.38 | osso | 0 | 0 | 4 | f | f | 0.08064516 | 0.27419354 | 0.03030303 | PRE |
| spilla3.38* | spilla | * | 3.38 | severa | 0 | 0 | 4 | f | f | 0.15463917 | 0.16494845 | 0.066176471 | PRE |
| spilla3.7281 | spilla | 1 | 3.728 | schiarita | only seen | 0 | 4 | f | f | 0 | 0.17857142 | 0.041420118 | PRE |
| spilla3.7281 | spilla | 1 | 3.728 | strega | only seen | 0 | 4 | f | f | 0 | 0.11111111 | 0.016025641 | PRE |
| spilla3.7281 | spilla | 1 | 3.728 | sally | only seen | 1 | 4 | f | f | 0 | 0.07142857 | 0.002949853 | PRE |
| spilla3.7281 | spilla | 1 | 3.728 | sumo | 0 | 1 | 4 | f | m | 0.133333333 | 0 | 0.203125 | PRE |
| spilla3.781 | spilla | 1 | 3.78 | stella | only seen | 0 | 5 | f | f | 0.03448276 | 0 | 0.020080321 | PRE |
| spilla3.781 | spilla | 1 | 3.78 | sonia | only seen | 0 | 5 | f | f | 0.44642857 | 0.19642857 | 0.041666667 | PRE |
| spilla3.781 | spilla | 1 | 3.78 | severa | 0 | 0 | 5 | f | f | 0.15463917 | 0.16494845 | 0.066176471 | PRE |
| spilla3.781 | spilla | 1 | 3.78 | tris | 0 | 0 | 5 | f | f | 0.12658228 | 0.25316455 | 0.010449321 | PRE |
| spilla3.781 | spilla | 1 | 3.78 | osso | 0 | 0 | 5 | f | f | 0.08064516 | 0.27419354 | 0.03030303 | PRE |
| spilla43 | spilla | 3 | 4 | roll | 0 | 0 | 4 | f | f | 0 | 0.11475409 | 0.036781609 | PRE |
| spilla43 | spilla | 3 | 4 | osso | 0 | 0 | 4 | f | f | 0.08064516 | 0.27419354 | 0.03030303 | PRE |
| spilla43 | spilla | 3 | 4 | tris | 0 | 0 | 4 | f | f | 0.12628558 | 0.25316455 | 0.010449321 | PRE |
| spilla6.382 | spilla | 2 | 6.38 | sonia | only seen | 1 | 3 | f | f | 0.44642857 | 0.19642857 | 0.041666667 | PRE |
| spilla6.382 | spilla | 2 | 6.38 | roll | 0 | 0 | 3 | f | f | 0 | 0.11475409 | 0.036781609 | PRE |
| spilla6.382 | spilla | 2 | 6.38 | tris | 0 | 0 | 3 | f | f | 0.12628558 | 0.25316455 | 0.010449321 | PRE |
| spot1.0843 | spot | 3 | 1.084 | sine | only seen | 0 | 7 | f | f | 0.207207207 | 0.198198198 | 0.050682261 | POST |
| spot1.0843 | spot | 3 | 1.084 | secco | only seen | 0 | 7 | f | m | 0.02564102 | 0.15384615 | 0.123076923 | POST |
| spot1.0843 | spot | 3 | 1.084 | scapola | only seen | 0 | 7 | f | f | 0 | 0.15584415 | 0.003787879 | POST |
| spot1.0843 | spot | 3 | 1.084 | susy | only seen | 1 | 7 | f | f | 0.061728395 | 0.22222222 | 0.027777778 | POST |
| spot1.0843 | spot | 3 | 1.084 | strega | 0 | 0 | 7 | f | f | 0.01754386 | 0.01754386 | 0.016025641 | POST |
| spot1.0843 | spot | 3 | 1.084 | schiarita | 0 | 0 | 7 | f | f | 0.03658536 | 0.20731707 | 0.041420118 | POST |
| spot1.0843 | spot | 3 | 1.084 | stacy | 0 | 0 | 7 | f | f | 0.16504854 | 0.1553398 | 0.021317829 | POST |
| spot1.1351 | spot | 1 | 1.135 | sine | seen and heard | 0 | 7 | f | f | 0.207207207 | 0.198198198 | 0.050682261 | POST |
| spot1.1351 | spot | 1 | 1.135 | tino | only heard | 0 | 7 | f | m | 0 | 0 | 0.111111111 | POST |
| spot1.1351 | spot | 1 | 1.135 | neomamma | only heard | 0 | 7 | f | f | 0.16806722 | 0.18487394 | 0.032840722 | POST |
| spot1.1351 | spot | 1 | 1.135 | strega | only heard | 0 | 7 | f | f | 0.01754386 | 0.01754386 | 0.016025641 | POST |
| spot1.1351 | spot | 1 | 1.135 | sumo | only heard | 0 | 7 | f | m | 0 | 0 | 0.203125 | POST |
| spot1.1351 | spot | 1 | 1.135 | spilla | only heard | 0 | 7 | f | f | 0.25 | 0 | 0.043678161 | POST |
| spot1.1351 | spot | 1 | 1.135 | schiarita | only heard | 0 | 7 | f | f | 0.03658536 | 0.20731707 | 0.041420118 | POST |
| spot1.671 | spot | 1 | 1.67 | stacy | only seen | 0 | 1 | f | f | 0.16504854 | 0.1553398 | 0.021317829 | POST |
| spot1.771 | spot | 1 | 1.77 | scapola | only seen | 0 | 5 | f | f | 0 | 0.15584415 | 0.003787879 | POST |
| spot1.771 | spot | 1 | 1.77 | stacy | only seen | 0 | 5 | f | f | 0.16504854 | 0.1553398 | 0.021317829 | POST |
| spot1.771 | spot | 1 | 1.77 | susy | only seen | 0 | 5 | f | f | 0.061728395 | 0.22222222 | 0.027777778 | POST |
| spot1.771 | spot | 1 | 1.77 | sfasciato | 0 | 0 | 5 | f | m | 0 | 0.12244897 | 0.024390244 | POST |
| spot1.771 | spot | 1 | 1.77 | strega | 0 | 0 | 5 | f | f | 0.01754386 | 0.01754386 | 0.016025641 | PRE |
| spot1.891 | spot | 1 | 1.89 | sciura | only heard | 0 | 1 | f | f | 0.21568627 | 0.1372549 | 0.06377858 | PRE |
| spot21 | spot | 1 | 2 | sally | only seen | 0 | 4 | f | f | 0.054545454 | 0.145454545 | 0.002949853 | PRE |
| spot21 | spot | 1 | 2 | neomamma | 0 | 0 | 4 | f | f | 0.16806722 | 0.18487394 | 0.032840722 | PRE |
| spot21 | spot | 1 | 2 | sine | 0 | 0 | 4 | f | f | 0.207207207 | 0.198198198 | 0.050682261 | PRE |
| spot21 | spot | 1 | 2 | susy | 0 | 0 | 4 | f | f | 0.061728395 | 0.22222222 | 0.027777778 | PRE |
| spot2.21 | spot | 1 | 2.2 | scapola | only seen | 0 | 6 | f | f | 0 | 0.15584415 | 0.003787879 | PRE |
| spot2.21 | spot | 1 | 2.2 | sine | only seen | 0 | 6 | f | f | 0.207207207 | 0.198198198 | 0.050682261 | PRE |
| spot2.21 | spot | 1 | 2.2 | secco | only seen | 0 | 6 | f | m | 0.02564102 | 0.15384615 | 0.123076923 | PRE |
| spot2.21 | spot | 1 | 2.2 | schiarita | 0 | 0 | 6 | f | f | 0.03658536 | 0.20731707 | 0.041420118 | PRE |
| spot2.21 | spot | 1 | 2.2 | sfasciato | 0 | 0 | 6 | f | m | 0 | 0.12244897 | 0.024390244 | PRE |
| spot2.21 | spot | 1 | 2.2 | strega | 0 | 0 | 6 | f | f | 0.01754386 | 0.01754386 | 0.016025641 | PRE |
| spot2.4461 | spot | 1 | 2.446 | sine | only seen | 0 | 4 | f | f | 0.207207207 | 0.198198198 | 0.050682261 | PRE |
| spot2.4461 | spot | 1 | 2.446 | stacy | only seen | 0 | 4 | f | f | 0.16504854 | 0.1553398 | 0.021317829 | PRE |
| spot2.4461 | spot | 1 | 2.446 | sciura | 0 | 1 | 4 | f | f | 0.21568627 | 0.1372549 | 0.06377858 | PRE |
| spot2.4461 | spot | 1 | 2.446 | neomamma | 0 | 0 | 4 | f | f | 0.16806722 | 0.18487394 | 0.032840722 | PRE |
| spot2.612 | spot | 2 | 2.61 | small | only seen | 0 | 8 | f | f | 0 | 0.12765957 | 0.044117647 | PRE |
| spot2.612 | spot | 2 | 2.61 | schiarita | only seen | 1 | 8 | f | f | 0.03658536 | 0.20731707 | 0.041420118 | PRE |
| spot2.612 | spot | 2 | 2.61 | scapola | 0 | 0 | 8 | f | f | 0 | 0.15584415 | 0.003787879 | PRE |
| spot2.612 | spot | 2 | 2.61 | ruga | 0 | 0 | 8 | f | f | 0.1 | 0.2 | 0.004608295 | PRE |
| spot2.612 | spot | 2 | 2.61 | sciura | 0 | 0 | 8 | f | f | 0.21568627 | 0.1372549 | 0.06377858 | PRE |
| spot2.612 | spot | 2 | 2.61 | stella | 0 | 0 | 8 | f | f | 0 | 0.15789473 | 0.020080321 | PRE |
| spot2.612 | spot | 2 | 2.61 | spiga | 0 | 0 | 8 | f | f | 0.11688312 | 0.16883116 | 0.018372703 | PRE |
| spot2.612 | spot | 2 | 2.61 | sally | 0 | 0 | 8 | f | f | 0.054545454 | 0.145454545 | 0.002949853 | PRE |
| stacy1.661 | stacy | 1 | 1.66 | sine | only seen | 1 | 1 | f | f | 0.03 | 0.07 | 0.050682261 | POST |
| stacy1.731 | stacy | 1 | 1.73 | sine | only seen | 0 | 7 | f | f | 0.03 | 0.07 | 0.050682261 | POST |
| stacy1.731 | stacy | 1 | 1.73 | secco | only seen | 0 | 7 | f | m | 0.01960784 | 0.07843137 | 0.123076923 | POST |
| stacy1.731 | stacy | 1 | 1.73 | scapola | only seen | 1 | 7 | f | f | 0.01428571 | 0.15714285 | 0.003787879 | POST |
| stacy1.731 | stacy | 1 | 1.73 | spot | only seen | 0 | 7 | f | f | 0.16504854 | 0.1553398 | 0.020576132 | POST |
| stacy1.731 | stacy | 1 | 1.73 | sfasciato | 0 | 0 | 7 | f | m | 0.13461538 | 0.07692307 | 0.024390244 | POST |
| stacy1.731 | stacy | 1 | 1.73 | neomamma | 0 | 0 | 7 | f | f | 0.01904761 | 0.19047619 | 0.032840722 | POST |
| stacy1.731 | stacy | 1 | 1.73 | small | 0 | 0 | 7 | f | f | 0.10869565 | 0.04347826 | 0.044117647 | POST |
| stacy1.841 | stacy | 1 | 1.84 | sciura | seen and heard | 1 | 2 | f | f | 0.02857142 | 0.14285714 | 0.06377858 | POST |
| stacy1.841 | stacy | 1 | 1.84 | spot | only heard | 0 | 2 | f | f | 0.16504854 | 0.1553398 | 0.020576132 | POST |
| stacy1.931 | stacy | 1 | 1.93 | sally | only heard | 0 | 4 | f | f | 0.02985074 | 0.05970149 | 0.002949853 | POST |
| stacy1.931 | stacy | 1 | 1.93 | rosa | only heard | 0 | 4 | f | f | 0.06382978 | 0 | 0.027131783 | POST |
| stacy1.931 | stacy | 1 | 1.93 | spot | only heard | 0 | 4 | f | f | 0.16504854 | 0.1553398 | 0.020576132 | POST |
| stacy1.931 | stacy | 1 | 1.93 | neomamma | only heard | 0 | 4 | f | f | 0.01904761 | 0.19047619 | 0.032840722 | POST |
| stacy2.111 | stacy | 1 | 2.11 | strega | only seen | 0 | 2 | f | f | 0 | 0.0140845 | 0.016025641 | POST |
| stacy2.111 | stacy | 1 | 2.11 | sorcia | 0 | 0 | 2 | f | f | 0.05555556 | 0.25 | 0.022727273 | POST |
| stacy2.31 | stacy | 1 | 2.3 | schiarita | seen and heard | 0 | 11 | f | f | 0.25490196 | 0.15686274 | 0.041420118 | POST |
| stacy2.31 | stacy | 1 | 2.3 | sine | seen and heard | 0 | 11 | f | f | 0.03 | 0.07 | 0.050682261 | POST |
| stacy2.31 | stacy | 1 | 2.3 | sguercia | seen and heard | 0 | 11 | f | f | 0 | 0.045454545 | 0 | POST |
| stacy2.31 | stacy | 1 | 2.3 | spilla | only heard | 0 | 11 | f | f | 0 | 0 | 0.043678161 | POST |
| stacy2.31 | stacy | 1 | 2.3 | rosa | only heard | 0 | 11 | f | f | 0.06382978 | 0 | 0.027131783 | POST |
| stacy2.31 | stacy | 1 | 2.3 | strega | only heard | 0 | 11 | f | f | 0 | 0.0140845 | 0.016025641 | POST |
| stacy2.31 | stacy | 1 | 2.3 | sfasciato | only heard | 0 | 11 | f | m | 0.13461538 | 0.07692307 | 0.024390244 | POST |
| stacy2.31 | stacy | 1 | 2.3 | sally | only heard | 0 | 11 | f | f | 0.02985074 | 0.05970149 | 0.002949853 | POST |
| stacy2.31 | stacy | 1 | 2.3 | scapola | only heard | 0 | 11 | f | f | 0.01428571 | 0.15714285 | 0.003787879 | POST |
| stacy2.31 | stacy | 1 | 2.3 | spot | only heard | 0 | 11 | f | f | 0.16504854 | 0.1553398 | 0.020576132 | POST |
| stacy2.31 | stacy | 1 | 2.3 | neomamma | only heard | 0 | 11 | f | f | 0.01904761 | 0.19047619 | 0.032840722 | POST |
| stacy2.351 | stacy | 1 | 2.35 | rosa | only seen | 0 | 9 | f | f | 0.06382978 | 0 | 0.027131783 | POST |
| stacy2.351 | stacy | 1 | 2.35 | schiarita | 0 | 0 | 9 | f | f | 0.25490196 | 0.15686274 | 0.041420118 | POST |
| stacy2.351 | stacy | 1 | 2.35 | neomamma | 0 | 0 | 9 | f | f | 0.01904761 | 0.19047619 | 0.032840722 | POST |
| stacy2.351 | stacy | 1 | 2.35 | spiga | 0 | 0 | 9 | f | f | 0.063291139 | 0.16455696 | 0.018372703 | POST |
| stacy2.351 | stacy | 1 | 2.35 | sfasciato | 0 | 0 | 9 | f | m | 0.13461538 | 0.07692307 | 0.024390244 | POST |
| stacy2.351 | stacy | 1 | 2.35 | scapola | 0 | 0 | 9 | f | f | 0.01428571 | 0.15714285 | 0.003787879 | POST |
| stacy2.351 | stacy | 1 | 2.35 | spot | 0 | 0 | 9 | f | f | 0.16504854 | 0.1553398 | 0.020576132 | POST |
| stacy2.351 | stacy | 1 | 2.35 | sally | 0 | 0 | 9 | f | f | 0.02985074 | 0.05970149 | 0.002949853 | POST |
| stacy2.351 | stacy | 1 | 2.35 | strega | 0 | 0 | 9 | f | f | 0 | 0.0140845 | 0.016025641 | POST |
| stacy2.471 | stacy | 1 | 2.47 | neomamma | only seen | 0 | 4 | f | f | 0.01904761 | 0.19047619 | 0.032840722 | POST |
| stacy2.471 | stacy | 1 | 2.47 | spot | 0 | 0 | 4 | f | f | 0.16504854 | 0.1553398 | 0.020576132 | PRE |
| stacy2.471 | stacy | 1 | 2.47 | schiarita | 0 | 0 | 4 | f | f | 0.25490196 | 0.15686274 | 0.041420118 | PRE |
| stacy2.471 | stacy | 1 | 2.47 | sally | 0 | 0 | 4 | f | f | 0.02985074 | 0.05970149 | 0.002949853 | PRE |
| stacy2.813 | stacy | 3 | 2.81 | sine | only seen | 0 | 8 | f | f | 0.03 | 0.07 | 0.050682261 | PRE |
| stacy2.813 | stacy | 3 | 2.81 | secco | only seen | 1 | 8 | f | m | 0.01960784 | 0.07843137 | 0.123076923 | PRE |
| stacy2.813 | stacy | 3 | 2.81 | scapola | 0 | 0 | 8 | f | f | 0.01428571 | 0.15714285 | 0.003787879 | PRE |
| stacy2.813 | stacy | 3 | 2.81 | sfasciato | 0 | 0 | 8 | f | m | 0.13461538 | 0.07692307 | 0.024390244 | PRE |
| stacy2.813 | stacy | 3 | 2.81 | spot | 0 | 0 | 8 | f | f | 0.16504854 | 0.1553398 | 0.020576132 | PRE |
| stacy2.813 | stacy | 3 | 2.81 | schiarita | 0 | 0 | 8 | f | f | 0.25490196 | 0.15686274 | 0.041420118 | PRE |
| stacy2.813 | stacy | 3 | 2.81 | strega | 0 | 0 | 8 | f | f | 0 | 0.0140845 | 0.016025641 | PRE |
| stacy2.813 | stacy | 3 | 2.81 | neomamma | 0 | 0 | 8 | f | f | 0.01904761 | 0.19047619 | 0.032840722 | PRE |
| stacy3.183 | stacy | 3 | 3.18 | schiarita | only seen | 0 | 12 | f | f | 0.25490196 | 0.15686274 | 0.041420118 | PRE |
| stacy3.183 | stacy | 3 | 3.18 | susy | only seen | 0 | 12 | f | f | 0.140625 | 0.09375 | 0.027777778 | PRE |
| stacy3.183 | stacy | 3 | 3.18 | spot | only seen | 0 | 12 | f | f | 0.16504854 | 0.1553398 | 0.020576132 | PRE |
| stacy3.183 | stacy | 3 | 3.18 | rosa | 0 | 0 | 12 | f | f | 0.06382978 | 0 | 0.027131783 | PRE |
| stacy3.183 | stacy | 3 | 3.18 | strega | 0 | 0 | 12 | f | f | 0 | 0.0140845 | 0.016025641 | PRE |
| stacy3.183 | stacy | 3 | 3.18 | small | 0 | 0 | 12 | f | f | 0.10869565 | 0.04347826 | 0.044117647 | PRE |
| stacy3.183 | stacy | 3 | 3.18 | scapola | 0 | 0 | 12 | f | f | 0.01428571 | 0.15714285 | 0.003787879 | PRE |
| stacy3.183 | stacy | 3 | 3.18 | neomamma | 0 | 0 | 12 | f | f | 0.01904761 | 0.19047619 | 0.032840722 | PRE |
| stacy3.183 | stacy | 3 | 3.18 | secco | 0 | 0 | 12 | f | m | 0.01960784 | 0.07843137 | 0.123076923 | PRE |
| stacy3.183 | stacy | 3 | 3.18 | sfasciato | 0 | 0 | 12 | f | m | 0.13461538 | 0.07692307 | 0.024390244 | PRE |
| stacy3.183 | stacy | 3 | 3.18 | sine | 0 | 1 | 12 | f | f | 0.03 | 0.07 | 0.050682261 | PRE |
| stacy3.183 | stacy | 3 | 3.18 | spiga | 0 | 0 | 12 | f | f | 0.063291139 | 0.16455696 | 0.018372703 | PRE |
| stacy3.763 | stacy | 3 | 3.76 | scapola | only seen | 0 | 8 | f | f | 0.01428571 | 0.15714285 | 0.003787879 | PRE |
| stacy3.763 | stacy | 3 | 3.76 | sonia | only seen | 0 | 8 | f | f | 0.04761904 | 0.04761904 | 0.041666667 | PRE |
| stacy3.763 | stacy | 3 | 3.76 | spiga | only seen | 0 | 8 | f | f | 0.063291139 | 0.16455696 | 0.018372703 | PRE |
| stacy3.763 | stacy | 3 | 3.76 | rosa | 0 | 0 | 8 | f | f | 0.06382978 | 0 | 0.027131783 | PRE |
| stacy3.763 | stacy | 3 | 3.76 | spot | 0 | 0 | 8 | f | f | 0.16504854 | 0.1553398 | 0.020576132 | PRE |
| stacy3.763 | stacy | 3 | 3.76 | neomamma | 0 | 0 | 8 | f | f | 0.01904761 | 0.19047619 | 0.032840722 | PRE |
| stacy3.763 | stacy | 3 | 3.76 | strega | 0 | 0 | 8 | f | f | 0 | 0.0140845 | 0.016025641 | PRE |
| stacy3.763 | stacy | 3 | 3.76 | small | 0 | 0 | 8 | f | f | 0.10869565 | 0.04347826 | 0.044117647 | PRE |
| stella2.072 | stella | 2 | 2.07 | osso | only seen | 0 | 4 | f | f | 0 | 0 | 0.03030303 | POST |
| stella2.072 | stella | 2 | 2.07 | spilla | 0 | 0 | 4 | f | f | 0.03448276 | 0 | 0.043678161 | POST |
| stella2.072 | stella | 2 | 2.07 | severa | 0 | 0 | 4 | f | f | 0 | 0 | 0.066176471 | POST |
| stella2.072 | stella | 2 | 2.07 | tris | 0 | 0 | 4 | f | f | 0 | 0.03846153 | 0.010449321 | POST |
| stella2.221 | stella | 1 | 2.22 | spilla | 0 | 0 | 4 | f | f | 0.03448276 | 0 | 0.043678161 | PRE |
| stella2.221 | stella | 1 | 2.22 | severa | 0 | 0 | 4 | f | f | 0 | 0 | 0.066176471 | PRE |
| stella2.221 | stella | 1 | 2.22 | tris | 0 | 0 | 4 | f | f | 0 | 0.03846153 | 0.010449321 | PRE |
| stella2.221 | stella | 1 | 2.22 | osso | 0 | 0 | 4 | f | f | 0 | 0 | 0.03030303 | PRE |
| stella2.561 | stella | 1 | 2.56 | spilla | only seen | 1 | 1 | f | f | 0.03448276 | 0 | 0.043678161 | PRE |
| stella5.471 | stella | 1 | 5.47 | tris | seen and heard | 0 | 2 | f | f | 0 | 0.03846153 | 0.010449321 | PRE |
| stella5.471 | stella | 1 | 5.47 | sorcia | only heard | 0 | 2 | f | f | 0.136363636 | 0 | 0.022727273 | PRE |
| strega1.61 | strega | 1 | 1.6 | rosa | only seen | 0 | 11 | f | f | 0.1707317 | 0.04878048 | 0.027131783 | POST |
| strega1.61 | strega | 1 | 1.6 | strega | 0 | 0 | 11 | f | f | 0 | 0 | 0.016025641 | POST |
| strega1.61 | strega | 1 | 1.6 | sine | 0 | 0 | 11 | f | f | 0 | 0.08064516 | 0.050682261 | POST |
| strega1.61 | strega | 1 | 1.6 | sfasciato | 0 | 0 | 11 | f | m | 0 | 0.05405405 | 0.024390244 | POST |
| strega1.61 | strega | 1 | 1.6 | neomamma | 0 | 0 | 11 | f | f | 0 | 0.14285714 | 0.032840722 | POST |
| strega1.61 | strega | 1 | 1.6 | spot | 0 | 0 | 11 | f | f | 0.01754386 | 0.01754386 | 0.020576132 | POST |
| strega1.61 | strega | 1 | 1.6 | stacy | 0 | 0 | 11 | f | f | 0 | 0.0140845 | 0.021317829 | POST |
| strega1.61 | strega | 1 | 1.6 | secco | 0 | 0 | 11 | f | m | 0.09302325 | 0.04651162 | 0.123076923 | POST |
| strega1.61 | strega | 1 | 1.6 | susy | 0 | 0 | 11 | f | f | 0.08163265 | 0.08163265 | 0.027777778 | POST |
| strega1.61 | strega | 1 | 1.6 | scapola | 0 | 0 | 11 | f | f | 0 | 0.06 | 0.003787879 | POST |
| strega1.61 | strega | 1 | 1.6 | spiga | 0 | 0 | 11 | f | f | 0.02083333 | 0.0625 | 0.018372703 | POST |
| strega1.671 | strega | 1 | 1.67 | spot | only seen | 0 | 13 | f | f | 0.01754386 | 0.01754386 | 0.020576132 | POST |
| strega1.671 | strega | 1 | 1.67 | susy | 0 | 0 | 13 | f | f | 0.08163265 | 0.08163265 | 0.027777778 | POST |
| strega1.671 | strega | 1 | 1.67 | rosa | 0 | 0 | 13 | f | f | 0.1707317 | 0.04878048 | 0.027131783 | POST |
| strega1.671 | strega | 1 | 1.67 | sine | 0 | 0 | 13 | f | f | 0 | 0.08064516 | 0.050682261 | POST |
| strega1.671 | strega | 1 | 1.67 | scapola | 0 | 0 | 13 | f | f | 0 | 0.06 | 0.003787879 | POST |
| strega1.671 | strega | 1 | 1.67 | stacy | 0 | 0 | 13 | f | f | 0 | 0.0140845 | 0.021317829 | POST |
| strega1.671 | strega | 1 | 1.67 | small | 0 | 0 | 13 | f | f | 0.21428571 | 0 | 0.044117647 | POST |
| strega1.671 | strega | 1 | 1.67 | stella | 0 | 0 | 13 | f | f | 0.28571429 | 0 | 0.020080321 | POST |
| strega1.671 | strega | 1 | 1.67 | spiga | 0 | 0 | 13 | f | f | 0.02083333 | 0.0625 | 0.018372703 | PRE |
| strega1.671 | strega | 1 | 1.67 | sally | 0 | 0 | 13 | f | f | 0.07142857 | 0 | 0.002949853 | PRE |
| strega1.671 | strega | 1 | 1.67 | schiarita | 0 | 0 | 13 | f | f | 0.04411764 | 0.07352941 | 0.041420118 | PRE |
| strega1.671 | strega | 1 | 1.67 | secco | 0 | 0 | 13 | f | m | 0.09302325 | 0.04651162 | 0.123076923 | PRE |
| strega1.671 | strega | 1 | 1.67 | neomamma | 0 | 0 | 13 | f | f | 0 | 0.14285714 | 0.032840722 | PRE |
| strega1.741 | strega | 1 | 1.74 | stacy | 0 | 0 | 4 | f | f | 0 | 0.0140845 | 0.021317829 | PRE |
| strega1.741 | strega | 1 | 1.74 | sguercia | 0 | 0 | 4 | f | f | 0.04761904 | 0 | 0 | PRE |
| strega1.741 | strega | 1 | 1.74 | secco | 0 | 0 | 4 | f | m | 0.09302325 | 0.04651162 | 0.123076923 | PRE |
| strega1.741 | strega | 1 | 1.74 | scapola | 0 | 0 | 4 | f | f | 0 | 0.06 | 0.003787879 | PRE |
| strega1.771 | strega | 1 | 1.77 | sine | only seen | 0 | 7 | f | f | 0 | 0.08064516 | 0.050682261 | PRE |
| strega1.771 | strega | 1 | 1.77 | secco | only seen | 0 | 7 | f | m | 0.09302325 | 0.04651162 | 0.123076923 | PRE |
| strega1.771 | strega | 1 | 1.77 | spot | only seen | 1 | 7 | f | f | 0.01754386 | 0.01754386 | 0.020576132 | PRE |
| strega1.771 | strega | 1 | 1.77 | scapola | 0 | 0 | 7 | f | f | 0 | 0.06 | 0.003787879 | PRE |
| strega1.771 | strega | 1 | 1.77 | sfasciato | 0 | 0 | 7 | f | m | 0 | 0.05405405 | 0.024390244 | PRE |
| strega1.771 | strega | 1 | 1.77 | schiarita | 0 | 0 | 7 | f | f | 0.04411764 | 0.07352941 | 0.041420118 | PRE |
| strega1.771 | strega | 1 | 1.77 | neomamma | 0 | 0 | 7 | f | f | 0 | 0.14285714 | 0.032840722 | PRE |
| strip1.1671 | strip | 1 | 1.167 | evasa | only seen | 1 | 2 | f | f | 0 | 0.07216494 | 0.024024024 | POST |
| strip1.3531 | strip | 1 | 1.353 | evasa | only seen | 0 | 2 | f | f | 0 | 0.07216494 | 0.024024024 | POST |
| strip1.3531 | strip | 1 | 1.353 | striscia | 0 | 0 | 2 | f | f | 0 | 0.09 | 0.00862069 | POST |
| strip1.3661 | strip | 1 | 1.366 | striscia | seen and heard | 0 | 2 | f | f | 0 | 0.09 | 0.00862069 | POST |
| strip1.3661 | strip | 1 | 1.366 | evasa | only heard | 0 | 2 | f | f | 0 | 0.07216494 | 0.024024024 | POST |
| strip2.7083 | strip | 3 | 2.708 | enzo | 0 | 0 | 3 | f | m | 0.04672897 | 0.18691588 | 0.181102362 | PRE |
| strip2.7083 | strip | 3 | 2.708 | striscia | 0 | 0 | 3 | f | f | 0 | 0.09 | 0.00862069 | PRE |
| strip2.7083 | strip | 3 | 2.708 | elly | 0 | 0 | 3 | f | f | 0 | 0.0625 | 0 | PRE |
| strip4.2883 | strip | 3 | 4.288 | striscia | only seen | 0 | 2 | f | f | 0 | 0.09 | 0.00862069 | PRE |
| strip4.2883 | strip | 3 | 4.288 | elly | only seen | 0 | 2 | f | f | 0 | 0.0625 | 0 | PRE |
| strip5.7673 | strip | 3 | 5.767 | evasa | only seen | 0 | 6 | f | f | 0 | 0.07216494 | 0.024024024 | PRE |
| strip5.7673 | strip | 3 | 5.767 | enzo | 0 | 0 | 6 | f | m | 0.04672897 | 0.18691588 | 0.181102362 | PRE |
| strip5.7673 | strip | 3 | 5.767 | edera | 0 | 0 | 6 | f | f | 0 | 0.11392405 | 0 | PRE |
| strip5.7673 | strip | 3 | 5.767 | e1m6 | 0 | 0 | 6 | f | f | 0.01234567 | 0.13580246 | 0.007092199 | PRE |
| strip5.7673 | strip | 3 | 5.767 | e2m6 | 0 | 0 | 6 | f | f | 0 | 0.20588235 | 0 | PRE |
| strip5.7673 | strip | 3 | 5.767 | striscia | 0 | 0 | 6 | f | f | 0 | 0.09 | 0.00862069 | PRE |
| striscia1.8391 | striscia | 1 | 1.839 | enzo | only seen | 1 | 6 | f | m | 0.06862745 | 0.14705882 | 0.181102362 | POST |
| striscia1.8391 | striscia | 1 | 1.839 | e1m6 | 0 | 0 | 6 | f | f | 0 | 0.13095238 | 0.007092199 | PRE |
| striscia1.8391 | striscia | 1 | 1.839 | strip | 0 | 0 | 6 | f | f | 0 | 0.09 | 0.020114943 | PRE |
| striscia1.8391 | striscia | 1 | 1.839 | evasa | 0 | 0 | 6 | f | f | 0.06185567 | 0.14432989 | 0.024024024 | PRE |
| striscia1.8391 | striscia | 1 | 1.839 | e2m6 | 0 | 1 | 6 | f | f | 0.041666667 | 0.166666667 | 0 | PRE |
| striscia1.8391 | striscia | 1 | 1.839 | edera | 0 | 0 | 6 | f | f | 0.01265822 | 0.1392405 | 0 | PRE |
| sumo1.013 | sumo | 3 | 1.01 | schiarita | only seen | 0 | 4 | m | f | 0 | 0.23255813 | 0.041420118 | POST |
| sumo1.013 | sumo | 3 | 1.01 | severa | 0 | 0 | 4 | m | f | 0.066666667 | 0.133333333 | 0.066176471 | POST |
| sumo1.013 | sumo | 3 | 1.01 | sguercia | 0 | 0 | 4 | m | f | 0 | 0 | 0 | POST |
| sumo1.013 | sumo | 3 | 1.01 | spilla | 0 | 0 | 4 | m | f | 0.133333333 | 0 | 0.043678161 | POST |
| sumo1.1173 | sumo | 3 | 1.117 | tino | only seen | 1 | 9 | m | m | 0 | 0.02857142 | 0.111111111 | POST |
| sumo1.1173 | sumo | 3 | 1.117 | sciura | only seen | 0 | 9 | m | f | 0.04 | 0.08 | 0.06377858 | POST |
| sumo1.1173 | sumo | 3 | 1.117 | schiarita | only seen | 0 | 9 | m | f | 0 | 0.23255813 | 0.041420118 | POST |
| sumo1.1173 | sumo | 3 | 1.117 | spilla | 0 | 0 | 9 | m | f | 0.133333333 | 0 | 0.043678161 | POST |
| sumo1.1173 | sumo | 3 | 1.117 | sine | 0 | 0 | 9 | m | f | 0.11764705 | 0 | 0.050682261 | POST |
| sumo1.1173 | sumo | 3 | 1.117 | spot | 0 | 0 | 9 | m | f | 0 | 0 | 0.020576132 | POST |
| sumo1.1173 | sumo | 3 | 1.117 | neomamma | 0 | 0 | 9 | m | f | 0 | 0.033333333 | 0.032840722 | POST |
| sumo1.1173 | sumo | 3 | 1.117 | strega | 0 | 0 | 9 | m | f | 0.04 | 0.04 | 0.016025641 | POST |
| sumo1.1173 | sumo | 3 | 1.117 | sorcia | 0 | 0 | 9 | m | f | 0 | 0.05 | 0.022727273 | POST |
| sumo1.1423 | sumo | 3 | 1.142 | susy | seen and heard | 0 | 5 | m | f | 0.20689655 | 0.03448275 | 0.027777778 | POST |
| sumo1.1423 | sumo | 3 | 1.142 | tino | seen and heard | 1 | 5 | m | m | 0 | 0.02857142 | 0.111111111 | POST |
| sumo1.1423 | sumo | 3 | 1.142 | spilla | only heard | 0 | 5 | m | f | 0.133333333 | 0 | 0.043678161 | POST |
| sumo1.1423 | sumo | 3 | 1.142 | schiarita | only heard | 0 | 5 | m | f | 0 | 0.23255813 | 0.041420118 | POST |
| sumo1.1423 | sumo | 3 | 1.142 | strega | only heard | 0 | 5 | m | f | 0.04 | 0.04 | 0.016025641 | POST |
| sumo1.1553 | sumo | 3 | 1.155 | spilla | seen and heard | 0 | 4 | m | f | 0.133333333 | 0 | 0.043678161 | POST |
| sumo1.1553 | sumo | 3 | 1.155 | schiarita | seen and heard | 0 | 4 | m | f | 0 | 0.23255813 | 0.041420118 | POST |
| sumo1.1553 | sumo | 3 | 1.155 | susy | seen and heard | 0 | 4 | m | f | 0.20689655 | 0.03448275 | 0.027777778 | POST |
| sumo1.1553 | sumo | 3 | 1.155 | tino | only heard | 0 | 4 | m | m | 0 | 0.02857142 | 0.111111111 | POST |
| sumo1.1711 | sumo | 1 | 1.171 | sciura | seen and heard | 0 | 3 | m | f | 0.04 | 0.08 | 0.06377858 | POST |
| sumo1.1711 | sumo | 1 | 1.171 | spot | seen and heard | 0 | 3 | m | f | 0 | 0 | 0.020576132 | POST |
| sumo1.1711 | sumo | 1 | 1.171 | sine | only heard | 0 | 3 | m | f | 0.11764705 | 0 | 0.050682261 | POST |
| sumo1.1853 | sumo | 3 | 1.185 | strega | only seen | 0 | 7 | m | f | 0.04 | 0.04 | 0.016025641 | POST |
| sumo1.1853 | sumo | 3 | 1.185 | schiarita | only seen | 0 | 7 | m | f | 0 | 0.23255813 | 0.041420118 | POST |
| sumo1.1853 | sumo | 3 | 1.185 | sine | only seen | 1 | 7 | m | f | 0.11764705 | 0 | 0.050682261 | POST |
| sumo1.1853 | sumo | 3 | 1.185 | tino | only seen | 0 | 7 | m | m | 0 | 0.02857142 | 0.111111111 | POST |
| sumo1.1853 | sumo | 3 | 1.185 | sciura | 0 | 0 | 7 | m | f | 0.04 | 0.08 | 0.06377858 | POST |
| sumo1.1853 | sumo | 3 | 1.185 | neomamma | 0 | 0 | 7 | m | f | 0 | 0.033333333 | 0.032840722 | POST |
| sumo1.1853 | sumo | 3 | 1.185 | spot | 0 | 0 | 7 | m | f | 0 | 0 | 0.020576132 | POST |
| sumo1.243 | sumo | 3 | 1.24 | schiarita | only seen | 0 | 5 | m | f | 0 | 0.23255813 | 0.041420118 | POST |
| sumo1.243 | sumo | 3 | 1.24 | severa | only seen | 0 | 5 | m | f | 0.066666667 | 0.133333333 | 0.066176471 | POST |
| sumo1.243 | sumo | 3 | 1.24 | strega | 0 | 0 | 5 | m | f | 0.04 | 0.04 | 0.016025641 | POST |
| sumo1.243 | sumo | 3 | 1.24 | sguercia | 0 | 0 | 5 | m | f | 0 | 0 | 0 | POST |
| sumo1.243 | sumo | 3 | 1.24 | sorcia | 0 | 0 | 5 | m | f | 0 | 0.05 | 0.022727273 | POST |
| sumo1.2613 | sumo | 3 | 1.261 | schiarita | seen and heard | 0 | 4 | m | f | 0 | 0.23255813 | 0.041420118 | POST |
| sumo1.2613 | sumo | 3 | 1.261 | strega | seen and heard | 0 | 4 | m | f | 0.04 | 0.04 | 0.016025641 | POST |
| sumo1.2613 | sumo | 3 | 1.261 | spilla | only heard | 1 | 4 | m | f | 0.133333333 | 0 | 0.043678161 | POST |
| sumo1.2613 | sumo | 3 | 1.261 | sally | only heard | 0 | 4 | m | f | 0 | 0 | 0.002949853 | POST |
| sumo1.3023 | sumo | 3 | 1.302 | tino | only heard | 0 | 2 | m | m | 0 | 0.02857142 | 0.111111111 | POST |
| sumo1.3023 | sumo | 3 | 1.302 | sguercia | only heard | 0 | 2 | m | f | 0 | 0 | 0 | POST |
| sumo1.43 | sumo | 3 | 1.4 | neomamma | only seen | 0 | 11 | m | f | 0 | 0.033333333 | 0.032840722 | POST |
| sumo1.43 | sumo | 3 | 1.4 | evasa | only seen | 0 | 11 | m | f | 0 | 0 | 0.024024024 | POST |
| sumo1.43 | sumo | 3 | 1.4 | severa | only seen | 0 | 11 | m | f | 0.066666667 | 0.133333333 | 0.066176471 | POST |
| sumo1.43 | sumo | 3 | 1.4 | strega | only seen | 0 | 11 | m | f | 0.04 | 0.04 | 0.016025641 | POST |
| sumo1.43 | sumo | 3 | 1.4 | enzo | only seen | 0 | 11 | m | m | 0 | 0 | 0.181102362 | PRE |
| sumo1.43 | sumo | 3 | 1.4 | strip | only seen | 0 | 11 | m | f | 0 | 0 | 0.020114943 | PRE |
| sumo1.43 | sumo | 3 | 1.4 | spot | 0 | 0 | 11 | m | f | 0 | 0 | 0.020576132 | PRE |
| sumo1.43 | sumo | 3 | 1.4 | stacy | 0 | 0 | 11 | m | f | 0.03125 | 0.09375 | 0.021317829 | PRE |
| sumo1.43 | sumo | 3 | 1.4 | sfasciato | 0 | 0 | 11 | m | m | 0 | 0 | 0.024390244 | PRE |
| sumo1.43 | sumo | 3 | 1.4 | susy | 0 | 0 | 11 | m | f | 0.20689655 | 0.03448275 | 0.027777778 | PRE |
| sumo1.43 | sumo | 3 | 1.4 | spiga | 0 | 0 | 11 | m | f | 0 | 0 | 0.018372703 | PRE |
| sumo1.5113 | sumo | 3 | 1.511 | sine | seen and heard | 0 | 8 | m | f | 0.11764705 | 0 | 0.050682261 | PRE |
| sumo1.5113 | sumo | 3 | 1.511 | stacy | seen and heard | 0 | 8 | m | f | 0.03125 | 0.09375 | 0.021317829 | PRE |
| sumo1.5113 | sumo | 3 | 1.511 | spot | seen and heard | 1 | 8 | m | f | 0 | 0 | 0.020576132 | PRE |
| sumo1.5113 | sumo | 3 | 1.511 | sorcia | only heard | 1 | 8 | m | f | 0 | 0.05 | 0.022727273 | PRE |
| sumo1.5113 | sumo | 3 | 1.511 | sciura | only heard | 0 | 8 | m | f | 0.04 | 0.08 | 0.06377858 | PRE |
| sumo1.5113 | sumo | 3 | 1.511 | spiga | only heard | 0 | 8 | m | f | 0 | 0 | 0.018372703 | PRE |
| sumo1.5113 | sumo | 3 | 1.511 | sguercia | only heard | 0 | 8 | m | f | 0 | 0 | 0 | PRE |
| sumo1.5113 | sumo | 3 | 1.511 | susy | 0 | 0 | 8 | m | f | 0.20689655 | 0.03448275 | 0.027777778 | PRE |
| sumo1.5272 | sumo | 2 | 1.527 | tino | seen and heard | 0 | 6 | m | m | 0 | 0.02857142 | 0.111111111 | PRE |
| sumo1.5272 | sumo | 2 | 1.527 | sine | seen and heard | 0 | 6 | m | f | 0.11764705 | 0 | 0.050682261 | PRE |
| sumo1.5272 | sumo | 2 | 1.527 | stacy | 0 | 0 | 6 | m | f | 0.03125 | 0.09375 | 0.021317829 | PRE |
| sumo1.5272 | sumo | 2 | 1.527 | strega | 0 | 0 | 6 | m | f | 0.04 | 0.04 | 0.016025641 | PRE |
| sumo1.5272 | sumo | 2 | 1.527 | schiarita | 0 | 0 | 6 | m | f | 0 | 0.23255813 | 0.041420118 | PRE |
| sumo1.5272 | sumo | 2 | 1.527 | neomamma | 0 | 0 | 6 | m | f | 0 | 0.033333333 | 0.032840722 | PRE |
| sumo1.563 | sumo | 3 | 1.56 | spot | only heard | 0 | 4 | m | f | 0 | 0 | 0.020576132 | PRE |
| sumo1.563 | sumo | 3 | 1.56 | strega | only heard | 0 | 4 | m | f | 0.04 | 0.04 | 0.016025641 | PRE |
| sumo1.563 | sumo | 3 | 1.56 | schiarita | only heard | 0 | 4 | m | f | 0 | 0.23255813 | 0.041420118 | PRE |
| sumo1.563 | sumo | 3 | 1.56 | neomamma | only heard | 1 | 4 | m | f | 0 | 0.033333333 | 0.032840722 | PRE |
| sumo1.5723 | sumo | 3 | 1.572 | spilla | only heard | 0 | 3 | m | f | 0.133333333 | 0 | 0.043678161 | PRE |
| sumo1.5723 | sumo | 3 | 1.572 | schiarita | only heard | 0 | 3 | m | f | 0 | 0.23255813 | 0.041420118 | PRE |
| sumo1.5723 | sumo | 3 | 1.572 | susy | only heard | 0 | 3 | m | f | 0.20689655 | 0.03448275 | 0.027777778 | PRE |
| sumo1.5983 | sumo | 3 | 1.598 | spot | seen and heard | 0 | 8 | m | f | 0 | 0 | 0.020576132 | PRE |
| sumo1.5983 | sumo | 3 | 1.598 | sine | seen and heard | 0 | 8 | m | f | 0.11764705 | 0 | 0.050682261 | PRE |
| sumo1.5983 | sumo | 3 | 1.598 | sciura | seen and heard | 0 | 8 | m | f | 0.04 | 0.08 | 0.06377858 | PRE |
| sumo1.5983 | sumo | 3 | 1.598 | sorcia | only heard | 0 | 8 | m | f | 0 | 0.05 | 0.022727273 | PRE |
| sumo1.5983 | sumo | 3 | 1.598 | neomamma | only heard | 0 | 8 | m | f | 0 | 0.033333333 | 0.032840722 | PRE |
| sumo1.5983 | sumo | 3 | 1.598 | spiga | only heard | 0 | 8 | m | f | 0 | 0 | 0.018372703 | PRE |
| sumo1.5983 | sumo | 3 | 1.598 | sguercia | only heard | 0 | 8 | m | f | 0 | 0 | 0 | PRE |
| sumo1.5983 | sumo | 3 | 1.598 | schiarita | only heard | 0 | 8 | m | f | 0 | 0.23255813 | 0.041420118 | PRE |
| sumo1.673 | sumo | 3 | 1.67 | sguercia | 0 | 0 | 12 | m | f | 0 | 0 | 0 | PRE |
| sumo1.673 | sumo | 3 | 1.67 | severa | 0 | 0 | 12 | m | f | 0.066666667 | 0.133333333 | 0.066176471 | PRE |
| sumo1.673 | sumo | 3 | 1.67 | schiarita | 0 | 0 | 12 | m | f | 0 | 0.23255813 | 0.041420118 | PRE |
| sumo1.673 | sumo | 3 | 1.67 | spilla | 0 | 0 | 12 | m | f | 0.133333333 | 0 | 0.043678161 | PRE |
| sumo1.673 | sumo | 3 | 1.67 | susy | 0 | 0 | 12 | m | f | 0.20689655 | 0.03448275 | 0.027777778 | PRE |
| sumo1.673 | sumo | 3 | 1.67 | neomamma | 0 | 0 | 12 | m | f | 0 | 0.033333333 | 0.032840722 | PRE |
| sumo1.673 | sumo | 3 | 1.67 | strip | 0 | 0 | 12 | m | f | 0 | 0 | 0.020114943 | PRE |
| sumo1.673 | sumo | 3 | 1.67 | sonia | 0 | 0 | 12 | m | f | 0 | 0 | 0.041666667 | PRE |
| sumo1.673 | sumo | 3 | 1.67 | spiga | 0 | 0 | 12 | m | f | 0 | 0 | 0.018372703 | PRE |
| sumo1.673 | sumo | 3 | 1.67 | sfasciato | 0 | 0 | 12 | m | m | 0 | 0 | 0.024390244 | PRE |
| sumo1.673 | sumo | 3 | 1.67 | evasa | 0 | 0 | 12 | m | f | 0 | 0 | 0.024024024 | PRE |
| sumo1.673 | sumo | 3 | 1.67 | sine | 0 | 0 | 12 | m | f | 0.11764705 | 0 | 0.050682261 | PRE |
| sumo1.7373 | sumo | 3 | 1.737 | sciura | seen and heard | 0 | 7 | m | f | 0.04 | 0.08 | 0.06377858 | PRE |
| sumo1.7373 | sumo | 3 | 1.737 | sine | seen and heard | 0 | 7 | m | f | 0.11764705 | 0 | 0.050682261 | PRE |
| sumo1.7373 | sumo | 3 | 1.737 | stacy | seen and heard | 0 | 7 | m | f | 0.03125 | 0.09375 | 0.021317829 | PRE |
| sumo1.7373 | sumo | 3 | 1.737 | sorcia | only heard | 0 | 7 | m | f | 0 | 0.05 | 0.022727273 | PRE |
| sumo1.7373 | sumo | 3 | 1.737 | neomamma | only heard | 0 | 7 | m | f | 0 | 0.033333333 | 0.032840722 | PRE |
| sumo1.7373 | sumo | 3 | 1.737 | spiga | only heard | 0 | 7 | m | f | 0 | 0 | 0.018372703 | PRE |
| sumo1.7373 | sumo | 3 | 1.737 | schiarita | only heard | 0 | 7 | m | f | 0 | 0.23255813 | 0.041420118 | PRE |
| sumo1.763 | sumo | 3 | 1.76 | spot | seen and heard | 0 | 11 | m | f | 0 | 0 | 0.020576132 | PRE |
| sumo1.763 | sumo | 3 | 1.76 | neomamma | seen and heard | 0 | 11 | m | f | 0 | 0.033333333 | 0.032840722 | PRE |
| sumo1.763 | sumo | 3 | 1.76 | strega | seen and heard | 0 | 11 | m | f | 0.04 | 0.04 | 0.016025641 | PRE |
| sumo1.763 | sumo | 3 | 1.76 | enzo | only heard | 1 | 11 | m | m | 0 | 0 | 0.181102362 | PRE |
| sumo1.763 | sumo | 3 | 1.76 | strip | only heard | 0 | 11 | m | f | 0 | 0 | 0.020114943 | PRE |
| sumo1.763 | sumo | 3 | 1.76 | stacy | only heard | 0 | 11 | m | f | 0.03125 | 0.09375 | 0.021317829 | PRE |
| sumo1.763 | sumo | 3 | 1.76 | sfasciato | only heard | 0 | 11 | m | m | 0 | 0 | 0.024390244 | PRE |
| sumo1.763 | sumo | 3 | 1.76 | tino | only heard | 0 | 11 | m | m | 0 | 0.02857142 | 0.111111111 | PRE |
| sumo1.763 | sumo | 3 | 1.76 | spiga | only heard | 0 | 11 | m | f | 0 | 0 | 0.018372703 | PRE |
| sumo1.763 | sumo | 3 | 1.76 | sine | only heard | 0 | 11 | m | f | 0.11764705 | 0 | 0.050682261 | PRE |
| sumo1.763 | sumo | 3 | 1.76 | severa | only heard | 0 | 11 | m | f | 0.066666667 | 0.133333333 | 0.066176471 | PRE |
| sumo1.8151 | sumo | 1 | 1.815 | sine | seen and heard | 0 | 9 | m | f | 0.11764705 | 0 | 0.050682261 | PRE |
| sumo1.8151 | sumo | 1 | 1.815 | strega | seen and heard | 0 | 9 | m | f | 0.04 | 0.04 | 0.016025641 | PRE |
| sumo1.8151 | sumo | 1 | 1.815 | stacy | only heard | 0 | 9 | m | f | 0.03125 | 0.09375 | 0.021317829 | PRE |
| sumo1.8151 | sumo | 1 | 1.815 | schiarita | only heard | 0 | 9 | m | f | 0 | 0.23255813 | 0.041420118 | PRE |
| sumo1.8151 | sumo | 1 | 1.815 | neomamma | only heard | 0 | 9 | m | f | 0 | 0.033333333 | 0.032840722 | PRE |
| sumo1.8151 | sumo | 1 | 1.815 | tino | only heard | 0 | 9 | m | m | 0 | 0.02857142 | 0.111111111 | PRE |
| sumo1.8673 | sumo | 3 | 1.867 | spilla | seen and heard | 0 | 6 | m | f | 0.133333333 | 0 | 0.043678161 | PRE |
| sumo1.8673 | sumo | 3 | 1.867 | schiarita | seen and heard | 0 | 6 | m | f | 0 | 0.23255813 | 0.041420118 | PRE |
| sumo1.8673 | sumo | 3 | 1.867 | tino | only heard | 0 | 6 | m | m | 0 | 0.02857142 | 0.111111111 | PRE |
| sumo1.8673 | sumo | 3 | 1.867 | sine | only heard | 0 | 6 | m | f | 0.11764705 | 0 | 0.050682261 | PRE |
| sumo1.8673 | sumo | 3 | 1.867 | neomamma | only heard | 0 | 6 | m | f | 0 | 0.033333333 | 0.032840722 | PRE |
| sumo1.8673 | sumo | 3 | 1.867 | strega | only heard | 0 | 6 | m | f | 0.04 | 0.04 | 0.016025641 | PRE |
| sumo1.873 | sumo | 3 | 1.87 | schiarita | seen and heard | 0 | 7 | m | f | 0 | 0.23255813 | 0.041420118 | PRE |
| sumo1.873 | sumo | 3 | 1.87 | spot | seen and heard | 0 | 7 | m | f | 0 | 0 | 0.020576132 | PRE |
| sumo1.873 | sumo | 3 | 1.87 | sine | seen and heard | 1 | 7 | m | f | 0.11764705 | 0 | 0.050682261 | PRE |
| sumo1.873 | sumo | 3 | 1.87 | tino | seen and heard | 0 | 7 | m | m | 0 | 0.02857142 | 0.111111111 | PRE |
| sumo1.873 | sumo | 3 | 1.87 | strega | only heard | 0 | 7 | m | f | 0.04 | 0.04 | 0.016025641 | PRE |
| sumo1.873 | sumo | 3 | 1.87 | sciura | only heard | 0 | 7 | m | f | 0.04 | 0.08 | 0.06377858 | PRE |
| sumo1.873 | sumo | 3 | 1.87 | neomamma | only heard | 0 | 7 | m | f | 0 | 0.033333333 | 0.032840722 | PRE |
| sumo1.93 | sumo | 3 | 1.9 | stacy | seen and heard | 0 | 11 | m | f | 0.03125 | 0.09375 | 0.021317829 | PRE |
| sumo1.93 | sumo | 3 | 1.9 | strega | seen and heard | 0 | 11 | m | f | 0.04 | 0.04 | 0.016025641 | PRE |
| sumo1.93 | sumo | 3 | 1.9 | tino | seen and heard | 1 | 11 | m | m | 0 | 0.02857142 | 0.111111111 | PRE |
| sumo1.93 | sumo | 3 | 1.9 | sine | seen and heard | 0 | 11 | m | f | 0.11764705 | 0 | 0.050682261 | PRE |
| sumo1.93 | sumo | 3 | 1.9 | spot | only heard | 0 | 11 | m | f | 0 | 0 | 0.020576132 | PRE |
| sumo1.93 | sumo | 3 | 1.9 | susy | only heard | 0 | 11 | m | f | 0.20689655 | 0.03448275 | 0.027777778 | PRE |
| sumo1.93 | sumo | 3 | 1.9 | neomamma | only heard | 0 | 11 | m | f | 0 | 0.033333333 | 0.032840722 | PRE |
| sumo1.93 | sumo | 3 | 1.9 | sfasciato | only heard | 0 | 11 | m | m | 0 | 0 | 0.024390244 | PRE |
| sumo1.93 | sumo | 3 | 1.9 | sonia | only heard | 0 | 11 | m | f | 0 | 0 | 0.041666667 | PRE |
| sumo1.93 | sumo | 3 | 1.9 | strip | only heard | 0 | 11 | m | f | 0 | 0 | 0.020114943 | PRE |
| sumo1.93 | sumo | 3 | 1.9 | enzo | only heard | 1 | 11 | m | m | 0 | 0 | 0.181102362 | PRE |
| sumo1.973 | sumo | 3 | 1.97 | sciura | only seen | 0 | 3 | m | f | 0.04 | 0.08 | 0.06377858 | PRE |
| sumo1.973 | sumo | 3 | 1.97 | spot | only seen | 0 | 3 | m | f | 0 | 0 | 0.020576132 | PRE |
| sumo1.973 | sumo | 3 | 1.97 | sine | 0 | 0 | 3 | m | f | 0.11764705 | 0 | 0.050682261 | PRE |
| sumo1.9991 | sumo | 1 | 1.999 | spilla | seen and heard | 0 | 9 | m | f | 0.133333333 | 0 | 0.043678161 | PRE |
| sumo1.9991 | sumo | 1 | 1.999 | tino | only heard | 0 | 9 | m | m | 0 | 0.02857142 | 0.111111111 | PRE |
| sumo1.9991 | sumo | 1 | 1.999 | schiarita | only heard | 0 | 9 | m | f | 0 | 0.23255813 | 0.041420118 | PRE |
| sumo1.9991 | sumo | 1 | 1.999 | sine | only heard | 0 | 9 | m | f | 0.11764705 | 0 | 0.050682261 | PRE |
| sumo1.9991 | sumo | 1 | 1.999 | spot | only heard | 1 | 9 | m | f | 0 | 0 | 0.020576132 | PRE |
| sumo1.9991 | sumo | 1 | 1.999 | sciura | only heard | 0 | 9 | m | f | 0.04 | 0.08 | 0.06377858 | PRE |
| sumo1.9991 | sumo | 1 | 1.999 | neomamma | only heard | 0 | 9 | m | f | 0 | 0.033333333 | 0.032840722 | PRE |
| sumo1.9991 | sumo | 1 | 1.999 | strega | only heard | 0 | 9 | m | f | 0.04 | 0.04 | 0.016025641 | PRE |
| sumo2.5081 | sumo | 1 | 2.508 | tino | seen and heard | 0 | 4 | m | m | 0 | 0.02857142 | 0.111111111 | PRE |
| sumo2.5081 | sumo | 1 | 2.508 | spilla | only heard | 0 | 4 | m | f | 0.133333333 | 0 | 0.043678161 | PRE |
| sumo2.5081 | sumo | 1 | 2.508 | schiarita | only heard | 0 | 4 | m | f | 0 | 0.23255813 | 0.041420118 | PRE |
| sumo2.5081 | sumo | 1 | 2.508 | susy | only heard | 0 | 4 | m | f | 0.20689655 | 0.03448275 | 0.027777778 | PRE |
| susy1.151 | susy | 1 | 1.15 | scapola | only seen | 0 | 6 | f | f | 0.26388889 | 0.22222222 | 0.003787879 | POST |
| susy1.151 | susy | 1 | 1.15 | stacy | only seen | 1 | 6 | f | f | 0.140625 | 0.09375 | 0.021317829 | POST |
| susy1.151 | susy | 1 | 1.15 | sine | 0 | 0 | 6 | f | f | 0.04938271 | 0.08641975 | 0.050682261 | POST |
| susy1.151 | susy | 1 | 1.15 | spot | 0 | 0 | 6 | f | f | 0.061728395 | 0.22222222 | 0.020576132 | POST |
| susy1.151 | susy | 1 | 1.15 | neomamma | 0 | 0 | 6 | f | f | 0 | 0.08450704 | 0.032840722 | POST |
| susy1.151 | susy | 1 | 1.15 | sfasciato | 0 | 0 | 6 | f | m | 0 | 0.1707317 | 0.024390244 | POST |
| susy1.191 | susy | 1 | 1.19 | neomamma | only seen | 0 | 10 | f | f | 0 | 0.08450704 | 0.032840722 | POST |
| susy1.191 | susy | 1 | 1.19 | spot | only seen | 0 | 10 | f | f | 0.061728395 | 0.22222222 | 0.020576132 | POST |
| susy1.191 | susy | 1 | 1.19 | sine | only seen | 0 | 10 | f | f | 0.04938271 | 0.08641975 | 0.050682261 | POST |
| susy1.191 | susy | 1 | 1.19 | rosa | only seen | 1 | 10 | f | f | 0.03703703 | 0 | 0.027131783 | POST |
| susy1.191 | susy | 1 | 1.19 | secco | 0 | 0 | 10 | f | m | 0.02631578 | 0.02631578 | 0.123076923 | POST |
| susy1.191 | susy | 1 | 1.19 | sfasciato | 0 | 0 | 10 | f | m | 0 | 0.1707317 | 0.024390244 | POST |
| susy1.191 | susy | 1 | 1.19 | schiarita | 0 | 0 | 10 | f | f | 0.01639344 | 0.09836065 | 0.041420118 | POST |
| susy1.191 | susy | 1 | 1.19 | strega | 0 | 0 | 10 | f | f | 0.08163265 | 0.08163265 | 0.016025641 | POST |
| susy1.191 | susy | 1 | 1.19 | scapola | 0 | 0 | 10 | f | f | 0.26388889 | 0.22222222 | 0.003787879 | POST |
| susy1.4291 | susy | 1 | 1.429 | strega | only seen | 0 | 7 | f | f | 0.08163265 | 0.08163265 | 0.016025641 | POST |
| susy1.4291 | susy | 1 | 1.429 | sine | 0 | 0 | 7 | f | f | 0.04938271 | 0.08641975 | 0.050682261 | POST |
| susy1.4291 | susy | 1 | 1.429 | tino | 0 | 0 | 7 | f | m | 0 | 0 | 0.111111111 | POST |
| susy1.4291 | susy | 1 | 1.429 | stacy | 0 | 0 | 7 | f | f | 0.140625 | 0.09375 | 0.021317829 | POST |
| susy1.4291 | susy | 1 | 1.429 | schiarita | 0 | 0 | 7 | f | f | 0.01639344 | 0.09836065 | 0.041420118 | POST |
| susy1.4291 | susy | 1 | 1.429 | neomamma | 0 | 0 | 7 | f | f | 0 | 0.08450704 | 0.032840722 | POST |
| susy1.4291 | susy | 1 | 1.429 | sumo | 0 | 0 | 7 | f | m | 0.20689655 | 0.03448275 | 0.203125 | POST |
| susy1.5381 | susy | 1 | 1.538 | stacy | only seen | 0 | 7 | f | f | 0.140625 | 0.09375 | 0.021317829 | POST |
| susy1.5381 | susy | 1 | 1.538 | sine | 0 | 0 | 7 | f | f | 0.04938271 | 0.08641975 | 0.050682261 | POST |
| susy1.5381 | susy | 1 | 1.538 | tino | 0 | 0 | 7 | f | m | 0 | 0 | 0.111111111 | POST |
| susy1.5381 | susy | 1 | 1.538 | strega | 0 | 0 | 7 | f | f | 0.08163265 | 0.08163265 | 0.016025641 | POST |
| susy1.5381 | susy | 1 | 1.538 | sumo | 0 | 0 | 7 | f | m | 0.20689655 | 0.03448275 | 0.203125 | POST |
| susy1.5381 | susy | 1 | 1.538 | schiarita | 0 | 0 | 7 | f | f | 0.01639344 | 0.09836065 | 0.041420118 | PRE |
| susy1.5381 | susy | 1 | 1.538 | neomamma | 0 | 0 | 7 | f | f | 0 | 0.08450704 | 0.032840722 | PRE |
| susy1.591 | susy | 1 | 1.59 | schiarita | only seen | 0 | 9 | f | f | 0.01639344 | 0.09836065 | 0.041420118 | PRE |
| susy1.591 | susy | 1 | 1.59 | sally | only seen | 0 | 9 | f | f | 0.26 | 0.02 | 0.002949853 | PRE |
| susy1.591 | susy | 1 | 1.59 | sfasciato | 0 | 0 | 9 | f | m | 0 | 0.1707317 | 0.024390244 | PRE |
| susy1.591 | susy | 1 | 1.59 | sine | 0 | 0 | 9 | f | f | 0.04938271 | 0.08641975 | 0.050682261 | PRE |
| susy1.591 | susy | 1 | 1.59 | scapola | 0 | 0 | 9 | f | f | 0.26388889 | 0.22222222 | 0.003787879 | PRE |
| susy1.591 | susy | 1 | 1.59 | spot | 0 | 0 | 9 | f | f | 0.061728395 | 0.22222222 | 0.020576132 | PRE |
| susy1.591 | susy | 1 | 1.59 | stacy | 0 | 0 | 9 | f | f | 0.140625 | 0.09375 | 0.021317829 | PRE |
| susy1.591 | susy | 1 | 1.59 | neomamma | 0 | 0 | 9 | f | f | 0 | 0.08450704 | 0.032840722 | PRE |
| susy1.591 | susy | 1 | 1.59 | spiga | 0 | 0 | 9 | f | f | 0 | 0.10909091 | 0.018372703 | PRE |
| susy1.861 | susy | 1 | 1.86 | sine | only seen | 0 | 8 | f | f | 0.04938271 | 0.08641975 | 0.050682261 | PRE |
| susy1.861 | susy | 1 | 1.86 | stacy | only seen | 0 | 8 | f | f | 0.140625 | 0.09375 | 0.021317829 | PRE |
| susy1.861 | susy | 1 | 1.86 | sfasciato | 0 | 0 | 8 | f | m | 0 | 0.1707317 | 0.024390244 | PRE |
| susy1.861 | susy | 1 | 1.86 | scapola | 0 | 0 | 8 | f | f | 0.26388889 | 0.22222222 | 0.003787879 | PRE |
| susy1.861 | susy | 1 | 1.86 | spot | 0 | 0 | 8 | f | f | 0.061728395 | 0.22222222 | 0.020576132 | PRE |
| susy1.861 | susy | 1 | 1.86 | strega | 0 | 0 | 8 | f | f | 0.08163265 | 0.08163265 | 0.016025641 | PRE |
| susy1.861 | susy | 1 | 1.86 | sally | 0 | 0 | 8 | f | f | 0.26 | 0.02 | 0.002949853 | PRE |
| susy1.861 | susy | 1 | 1.86 | small | 0 | 0 | 8 | f | f | 0.20588235 | 0.08823529 | 0.044117647 | PRE |
| susy2.43 | susy | 3 | 2.4 | stacy | only seen | 1 | 9 | f | f | 0.140625 | 0.09375 | 0.021317829 | PRE |
| susy2.43 | susy | 3 | 2.4 | sorcia | 0 | 0 | 9 | f | f | 0 | 0 | 0.022727273 | PRE |
| susy2.43 | susy | 3 | 2.4 | small | 0 | 0 | 9 | f | f | 0.20588235 | 0.08823529 | 0.044117647 | PRE |
| susy2.43 | susy | 3 | 2.4 | scapola | 0 | 0 | 9 | f | f | 0.26388889 | 0.22222222 | 0.003787879 | PRE |
| susy2.43 | susy | 3 | 2.4 | ruga | 0 | 0 | 9 | f | f | 0 | 0.2 | 0.004608295 | PRE |
| susy2.43 | susy | 3 | 2.4 | sciura | 0 | 0 | 9 | f | f | 0 | 0.08333333 | 0.06377858 | PRE |
| susy2.43 | susy | 3 | 2.4 | stella | 0 | 0 | 9 | f | f | 0 | 0 | 0.020080321 | PRE |
| susy2.43 | susy | 3 | 2.4 | spiga | 0 | 0 | 9 | f | f | 0 | 0.10909091 | 0.018372703 | PRE |
| susy2.43 | susy | 3 | 2.4 | sally | 0 | 0 | 9 | f | f | 0.26 | 0.02 | 0.002949853 | PRE |
| susy4.253 | susy | 3 | 4.25 | scapola | only seen | 0 | 4 | f | f | 0.26388889 | 0.22222222 | 0.003787879 | PRE |
| susy4.253 | susy | 3 | 4.25 | sfasciato | 0 | 0 | 4 | f | m | 0 | 0.1707317 | 0.024390244 | PRE |
| susy4.253 | susy | 3 | 4.25 | schiarita | 0 | 0 | 4 | f | f | 0.01639344 | 0.09836065 | 0.041420118 | PRE |
| susy4.253 | susy | 3 | 4.25 | sally | 0 | 0 | 4 | f | f | 0.26 | 0.02 | 0.002949853 | PRE |
| susy5.851 | susy | 1 | 5.85 | schiarita | only seen | 0 | 3 | f | f | 0.01639344 | 0.09836065 | 0.041420118 | PRE |
| susy5.851 | susy | 1 | 5.85 | scapola | 0 | 0 | 3 | f | f | 0.26388889 | 0.22222222 | 0.003787879 | PRE |
| susy5.851 | susy | 1 | 5.85 | stella | 0 | 0 | 3 | f | f | 0 | 0 | 0.020080321 | PRE |
| tinino11.381 | tinino1 | 1 | 1.38 | sandro | seen and heard | 0 | 8 | m | m | 0.34482759 | 0.03448275 | 0.089430894 | POST |
| tinino11.381 | tinino1 | 1 | 1.38 | tinino2 | seen and heard | 0 | 8 | m | m | 0.09090909 | 0.45454545 | 0.037037037 | POST |
| tinino11.381 | tinino1 | 1 | 1.38 | ruga | only heard | 0 | 8 | m | f | 0 | 0 | 0.004608295 | POST |
| tinino11.381 | tinino1 | 1 | 1.38 | roll | only heard | 0 | 8 | m | f | 0 | 0.037037037 | 0.036781609 | POST |
| tinino11.381 | tinino1 | 1 | 1.38 | tris | only heard | 0 | 8 | m | f | 0 | 0 | 0.010449321 | POST |
| tinino11.381 | tinino1 | 1 | 1.38 | scapola | only heard | 0 | 8 | m | f | 0 | 0 | 0.003787879 | POST |
| tinino11.381 | tinino1 | 1 | 1.38 | osso | only heard | 0 | 8 | m | f | 0 | 0 | 0.03030303 | POST |
| tinino11.381 | tinino1 | 1 | 1.38 | rocco | only heard | 0 | 8 | m | m | 0 | 0 | 0.25 | POST |
| tinino11.6911 | tinino1 | 1 | 1.691 | sandro | seen and heard | 0 | 7 | m | m | 0.34482759 | 0.03448275 | 0.089430894 | POST |
| tinino11.6911 | tinino1 | 1 | 1.691 | osso | seen and heard | 0 | 7 | m | f | 0 | 0 | 0.03030303 | POST |
| tinino11.6911 | tinino1 | 1 | 1.691 | roll | only heard | 0 | 7 | m | f | 0 | 0.037037037 | 0.036781609 | POST |
| tinino11.6911 | tinino1 | 1 | 1.691 | rocco | only heard | 0 | 7 | m | m | 0 | 0 | 0.25 | POST |
| tinino11.6911 | tinino1 | 1 | 1.691 | tris | only heard | 0 | 7 | m | f | 0 | 0 | 0.010449321 | PRE |
| tinino11.6911 | tinino1 | 1 | 1.691 | scapola | only heard | 0 | 7 | m | f | 0 | 0 | 0.003787879 | PRE |
| tinino11.6911 | tinino1 | 1 | 1.691 | ruga | only heard | 0 | 7 | m | f | 0 | 0 | 0.004608295 | PRE |
| tinino12.0661 | tinino1 | 1 | 2.066 | sandro | 0 | 0 | 4 | m | m | 0.34482759 | 0.03448275 | 0.089430894 | PRE |
| tinino12.0661 | tinino1 | 1 | 2.066 | roll | 0 | 0 | 4 | m | f | 0 | 0.037037037 | 0.036781609 | PRE |
| tinino12.0661 | tinino1 | 1 | 2.066 | osso | 0 | 0 | 4 | m | f | 0 | 0 | 0.03030303 | PRE |
| tinino12.0661 | tinino1 | 1 | 2.066 | ruga | 0 | 0 | 4 | m | f | 0 | 0 | 0.004608295 | PRE |
| tinino21.2271 | tinino2 | 1 | 1.227 | roll | 0 | 0 | 5 | m | f | 0 | 0.04347826 | 0.036781609 | POST |
| tinino21.2271 | tinino2 | 1 | 1.227 | scapola | 0 | 0 | 5 | m | f | 0 | 0 | 0.003787879 | POST |
| tinino21.2271 | tinino2 | 1 | 1.227 | osso | 0 | 0 | 5 | m | f | 0 | 0 | 0.03030303 | POST |
| tinino21.2271 | tinino2 | 1 | 1.227 | sandro | 0 | 0 | 5 | m | m | 0.44 | 0.12 | 0.089430894 | POST |
| tinino21.2271 | tinino2 | 1 | 1.227 | tris | 0 | 0 | 5 | m | f | 0 | 0 | 0.010449321 | PRE |
| tinino21.5381 | tinino2 | 1 | 1.538 | sandro | seen and heard | 1 | 5 | m | m | 0.44 | 0.12 | 0.089430894 | PRE |
| tinino21.5381 | tinino2 | 1 | 1.538 | roll | only heard | 0 | 5 | m | f | 0 | 0.04347826 | 0.036781609 | PRE |
| tinino21.5381 | tinino2 | 1 | 1.538 | scapola | only heard | 0 | 5 | m | f | 0 | 0 | 0.003787879 | PRE |
| tinino21.5381 | tinino2 | 1 | 1.538 | osso | only heard | 0 | 5 | m | f | 0 | 0 | 0.03030303 | PRE |
| tinino21.5381 | tinino2 | 1 | 1.538 | tris | only heard | 0 | 5 | m | f | 0 | 0 | 0.010449321 | PRE |
| tinino22.21 | tinino2 | 1 | 2.2 | tinino1 | 0 | 0 | 7 | m | m | 0.09090909 | 0.45454545 | 0.021505376 | PRE |
| tinino22.21 | tinino2 | 1 | 2.2 | sandro | 0 | 0 | 7 | m | m | 0.44 | 0.12 | 0.089430894 | PRE |
| tinino22.21 | tinino2 | 1 | 2.2 | rocco | 0 | 1 | 7 | m | m | 0 | 0 | 0.25 | PRE |
| tinino22.21 | tinino2 | 1 | 2.2 | roll | 0 | 0 | 7 | m | f | 0 | 0.04347826 | 0.036781609 | PRE |
| tinino22.21 | tinino2 | 1 | 2.2 | tris | 0 | 0 | 7 | m | f | 0 | 0 | 0.010449321 | PRE |
| tinino22.21 | tinino2 | 1 | 2.2 | ruga | 0 | 0 | 7 | m | f | 0 | 0 | 0.004608295 | PRE |
| tinino22.21 | tinino2 | 1 | 2.2 | scapola | 0 | 0 | 7 | m | f | 0 | 0 | 0.003787879 | PRE |
| tino1.0841 | tino | 1 | 1.084 | sumo | only seen | 1 | 4 | m | m | 0 | 0.02857142 | 0.203125 | POST |
| tino1.0841 | tino | 1 | 1.084 | spilla | 0 | 0 | 4 | m | f | 0 | 0 | 0.043678161 | POST |
| tino1.0841 | tino | 1 | 1.084 | schiarita | 0 | 0 | 4 | m | f | 0 | 0.04 | 0.041420118 | POST |
| tino1.0841 | tino | 1 | 1.084 | susy | 0 | 0 | 4 | m | f | 0 | 0 | 0.027777778 | POST |
| tino1.5241 | tino | 1 | 1.524 | sciura | only seen | 0 | 3 | m | f | 0 | 0 | 0.06377858 | POST |
| tino1.5241 | tino | 1 | 1.524 | sine | 0 | 0 | 3 | m | f | 0 | 0 | 0.050682261 | POST |
| tino1.5241 | tino | 1 | 1.524 | neomamma | 0 | 0 | 3 | m | f | 0 | 0.191489362 | 0.032840722 | POST |
| tino1.6053 | tino | 3 | 1.605 | spilla | 0 | 0 | 5 | m | f | 0 | 0 | 0.043678161 | PRE |
| tino1.6053 | tino | 3 | 1.605 | schiarita | 0 | 0 | 5 | m | f | 0 | 0.04 | 0.041420118 | PRE |
| tino1.6053 | tino | 3 | 1.605 | sally | 0 | 0 | 5 | m | f | 0.1 | 0.1 | 0.002949853 | PRE |
| tino1.6053 | tino | 3 | 1.605 | strega | 0 | 0 | 5 | m | f | 0 | 0 | 0.016025641 | PRE |
| tino1.6053 | tino | 3 | 1.605 | susy | 0 | 0 | 5 | m | f | 0 | 0 | 0.027777778 | PRE |
| tino1.6193 | tino | 3 | 1.619 | sumo | seen and heard | 1 | 7 | m | m | 0 | 0.02857142 | 0.203125 | PRE |
| tino1.6193 | tino | 3 | 1.619 | sine | seen and heard | 0 | 7 | m | f | 0 | 0 | 0.050682261 | PRE |
| tino1.6193 | tino | 3 | 1.619 | spot | seen and heard | 0 | 7 | m | f | 0 | 0 | 0.020576132 | PRE |
| tino1.6193 | tino | 3 | 1.619 | strega | only heard | 0 | 7 | m | f | 0 | 0 | 0.016025641 | PRE |
| tino1.6193 | tino | 3 | 1.619 | schiarita | only heard | 0 | 7 | m | f | 0 | 0.04 | 0.041420118 | PRE |
| tino1.6193 | tino | 3 | 1.619 | sciura | only heard | 0 | 7 | m | f | 0 | 0 | 0.06377858 | PRE |
| tino1.6193 | tino | 3 | 1.619 | neomamma | only heard | 0 | 7 | m | f | 0 | 0 | 0.032840722 | PRE |
| tino1.683 | tino | 3 | 1.68 | spilla | 0 | 0 | 3 | m | f | 0 | 0 | 0.043678161 | PRE |
| tino1.683 | tino | 3 | 1.68 | schiarita | 0 | 0 | 3 | m | f | 0 | 0.04 | 0.041420118 | PRE |
[truncated: 5,814 more chars]
